# Supplementary material for: Cyclic stretch induces autophagy-mediated focal adhesion remodeling and activates mitochondria
Source: Life Sci Alliance. 2026 Feb 5;9(4):e202503347. doi: 10.26508/lsa.202503347 (PMC12877405; doi:10.26508/lsa.202503347)
Supplement: Supplementary file 1 [file LSA-2025-03347_SdataF1.pdf]

Source data Figure 1D for ML cells

Main orientation of actin fibers per cell equivalent in [°]

Unstr.	30 min str. -	1 h str. -	4 h str.-
0,26136506	16,9448389	16,8146797	61,3728815
0,30258308	19,0426335	30,4409024	61,9897484
0,43036745	22,9974346	31,9189895	65,6023898
0,44093709	30,2315022	42,4727451	66,2850979
0,44671637	35,3991069	46,7110225	66,9125524
0,57414791	35,7319365	47,1065997	66,9265867
0,73134456	37,2543288	47,1303674	67,4831103
0,77450462	38,1706543	48,4059243	67,5316416
0,80239939	39,0409138	49,0499385	67,6388457
0,84068034	39,3990256	50,7789028	67,6526496
0,9122809	40,5686815	50,7843	67,7287061
0,92279761	41,5418664	50,9444396	68,0721741
0,9262669	42,5606021	51,068951	69,3724964
0,96367912	42,9608917	51,5785812	69,4638023
0,99966968	44,1882133	51,6052715	69,5599335
1,0573679	44,386695	52,0118405	69,7943066
1,12402335	45,5374243	53,0594227	69,8982798
1,13675218	46,0109314	53,1256044	70,0240172
1,27684498	46,2245171	53,2087445	70,0895239
1,50427469	46,5181328	54,3167717	70,3097131
1,55586303	46,8107032	54,6299516	70,9425886
1,62403947	46,8692916	54,6417011	71,0263128
1,65406802	46,9071435	54,8155431	71,3221695
1,67058309	46,9535693	55,117609	71,6738944
1,70262421	46,9933519	55,3143414	71,7120791
1,71963262	47,7458324	55,5188204	71,7538444
1,73923617	48,0498914	55,6220494	71,7843794
1,89819974	48,1149393	55,6780138	71,8488231
2,00127838	48,6885709	56,0033471	72,0159669
2,01001919	48,7844636	56,0054948	72,143018
2,11388949	48,9231941	56,1132639	72,148059
2,18413063	49,3824111	56,1565541	72,1921981
2,18553768	49,4088711	56,2452086	72,305879
2,19080016	49,4238569	56,4344654	72,3984565
2,19905451	49,8138678	56,5223878	72,4048218
2,30062276	49,8725244	56,5382761	72,4096185
2,33160009	50,2119424	56,6299738	72,5968314
2,39444743	50,4833775	56,7667354	72,7676054
2,44992604	50,6325897	57,1835732	72,7764626
2,57883148	50,7127319	57,1944151	72,781141
2,59128609	50,7640827	57,5805803	72,7860551
2,64800629	50,909239	57,8401582	72,8814041
2,69341676	50,9595489	57,8411286	72,9381824
2,74697559	51,1898451	58,0199618	72,9391582

2,75958635	51,2981719	58,1557702	72,942734
2,78054407	51,3125051	58,2470934	72,9944054
2,98456718	51,5866917	58,2620326	73,0761189
2,99980781	51,6126589	58,3027038	73,271449
3,04333159	51,7774977	58,5589532	73,4266265
3,07852344	51,8122218	58,6440925	73,5173512
3,12405636	51,8589166	58,7474872	73,529634
3,14889073	51,9019544	58,8214927	73,5673881
3,28961541	51,9449616	58,8815738	73,6442275
3,31996405	52,0311211	58,8862448	73,6736968
3,34868143	52,1035197	58,897092	73,6834793
3,40990078	52,1271368	58,900376	73,686798
3,41496649	52,2312061	58,9389897	73,7411287
3,77319416	52,3681085	59,1106569	73,7779119
3,90097114	52,5903866	59,2632533	73,8017604
3,95121894	52,756799	59,3174622	73,8640327
4,06540283	52,9658676	59,3786497	73,8857664
4,08420631	52,9727909	59,4778932	73,9631365
4,26547313	53,2016712	59,5107473	74,058378
4,30697516	53,2360383	59,5501899	74,1030092
4,4317697	53,3263575	59,618962	74,1987709
4,70359978	53,3495802	59,6319645	74,2210465
4,71663365	53,3603198	59,6952416	74,2792621
4,8062323	53,3889569	59,7207834	74,2862138
4,9263349	53,4549478	59,757545	74,3154724
5,13225359	53,4706153	59,7872273	74,3216182
5,1741002	53,576313	59,8233263	74,3589496
5,18969815	53,5968571	60,1962577	74,373536
5,20280997	53,6079439	60,3380413	74,3956496
5,21213731	53,6183351	60,5326521	74,4589081
5,23037896	53,6187951	60,5562466	74,4666043
5,30901955	53,7203208	60,6229069	74,4757928
5,34571022	53,7514872	60,7024351	74,6504618
5,35631523	53,7530663	60,7711979	74,6679385
5,36319889	53,8133589	60,8385943	74,8612052
5,39243905	53,8271673	61,1853349	75,0406077
5,442175	54,0245901	61,2036004	75,0812386
5,49743171	54,0404987	61,2187277	75,0946459
5,80754359	54,1239554	61,3005926	75,1460665
5,90915828	54,1334805	61,3319981	75,2197544
5,95157776	54,320962	61,4932755	75,2728807
6,10613838	54,4886587	61,542384	75,3677714
6,17123742	54,4951992	61,5644059	75,3894845
6,25064156	54,5443496	61,585271	75,4465505
6,27353933	54,5562181	61,5994813	75,4655724
6,30446407	54,6426252	61,6098842	75,5125664
6,36396639	55,0054682	61,6200889	75,5477646

6,45526256	55,1586365	61,6382043	75,5501009
6,48477621	55,2510296	61,7122764	75,5767292
6,50503184	55,2513044	61,9448055	75,5882402
6,64042638	55,2812584	62,1368384	75,6513785
6,6985411	55,3059512	62,2384878	75,6614064
6,82964177	55,437117	62,2602823	75,7417058
6,85951877	55,7767876	62,3641731	75,8234203
6,90163335	55,9250058	62,3748826	75,8273991
6,90447193	55,9497132	62,4028941	75,8534603
7,01214969	56,0785994	62,5475014	75,87439
7,12352105	56,0789817	62,5737458	75,8757995
7,18497929	56,2429529	62,76781	75,8825131
7,34010145	56,3258275	62,7684205	75,8895157
7,56450962	56,4333798	62,7784765	75,937894
8,05718859	56,4436265	62,8218145	75,9755087
8,08042803	56,449491	62,8721961	75,9829014
8,14704674	56,4552616	62,8784658	76,0684004
8,20585609	56,4573997	62,8975363	76,0853624
8,22727769	56,6444728	62,9488688	76,1422669
8,2514028	56,6529571	62,9694185	76,1470046
8,52691323	56,6623088	63,0628339	76,411398
8,54085165	56,7147751	63,086025	76,5159487
8,58671467	56,8221274	63,096461	76,5569262
8,67826407	56,8773999	63,1650921	76,5921221
8,83304355	56,978193	63,1861234	76,6103006
8,95687411	56,984799	63,2407061	76,6263159
9,17391653	57,0767263	63,286124	76,6746425
9,20091204	57,113067	63,3201932	76,7156332
9,30085313	57,1263744	63,4741855	76,8506674
9,41910954	57,1279957	63,4913485	76,8615881
9,59930841	57,1308115	63,5445344	76,878981
9,70855734	57,147648	63,5823929	76,8836199
9,79195362	57,2311172	63,6035292	76,9120393
9,81849408	57,2311317	63,6382295	76,9231794
9,87466408	57,2742437	63,6733547	76,9367938
10,1267967	57,3132522	63,6918557	76,9568807
10,1318749	57,3227065	63,6928439	76,9840177
10,181591	57,3283439	63,7263861	77,0784754
10,3245027	57,4145544	63,7765984	77,0896522
10,3510991	57,4148375	63,7883294	77,0910933
10,3525548	57,4173743	63,8086557	77,1292173
10,6463799	57,5759202	63,8329113	77,185467
10,7390663	57,5876436	63,8576865	77,2003948
10,7403714	57,5893269	63,8661406	77,2099932
10,7712153	57,6250576	63,866348	77,2512678
10,8262054	57,7694439	63,9128283	77,2515522
10,9124898	57,7936253	64,0275254	77,3146561

11,038762	57,8002483	64,0616928	77,3290355
11,1535726	57,9703256	64,1157385	77,4085541
11,1733109	57,9826948	64,1609588	77,5274497
11,2368433	57,9959297	64,2137863	77,5776296
11,2632565	58,0525095	64,2865191	77,5876147
11,5256935	58,0727231	64,2901181	77,647102
11,6047654	58,0856529	64,3533785	77,6695517
11,8282142	58,1473918	64,3639052	77,711905
12,0648426	58,1564539	64,3845913	77,7766718
12,1493181	58,2186605	64,4121502	77,8477994
12,2603703	58,2198756	64,4638299	77,8668044
12,2620156	58,2329426	64,478169	77,8933531
12,3829091	58,2627461	64,5191163	77,907283
12,42463	58,2990923	64,5464457	77,9541605
12,5407495	58,3664915	64,5850118	77,9722256
12,714703	58,3756826	64,613204	77,9851532
12,9162411	58,3996272	64,6331493	77,9911045
12,9548392	58,431002	64,6351833	78,0473664
13,0434895	58,445095	64,6391601	78,1368258
13,1327417	58,5430807	64,6670037	78,1459973
13,1699519	58,5542801	64,7413169	78,1637633
13,3329315	58,5954286	64,7473039	78,1941467
13,9158779	58,6485041	64,8298357	78,1967885
14,0903299	58,6711919	64,8396978	78,2072348
14,3981219	58,6785767	64,8812962	78,2092187
14,5145869	58,6862832	64,9417229	78,2097936
14,9547843	58,6932586	64,9419703	78,2226642
14,9926198	58,8334871	64,9442353	78,2349316
15,2344836	58,8688103	64,9554471	78,2694738
15,2741462	58,9011059	64,9662136	78,3148153
15,5190808	58,9233697	64,9939608	78,3437141
15,5844511	59,038115	65,0105442	78,3628339
15,6532285	59,0415251	65,0159245	78,3911258
16,0245605	59,0699942	65,0191167	78,4047771
16,0666592	59,1002141	65,0799932	78,4107643
16,194509	59,1424991	65,1065005	78,5311762
16,5513375	59,1737006	65,122442	78,549349
16,6505691	59,2010933	65,1393174	78,639193
16,6771697	59,2040024	65,1948008	78,6637801
16,7067264	59,2167965	65,3403471	78,6729575
16,7792026	59,2282692	65,3495452	78,6879506
16,9165747	59,2408315	65,4518206	78,7200145
17,2816839	59,2463931	65,4738053	78,7218082
17,3926812	59,292122	65,558924	78,7386705
17,4342137	59,503524	65,5777591	78,7503845
17,4441672	59,5372322	65,6411596	78,7541918
17,475563	59,5509154	65,6565748	78,7560118

17,6937765	59,584883	65,7144594	78,7811135
17,9265613	59,6371168	65,7297704	78,8041828
18,0157637	59,6452664	65,7581829	78,8352734
18,5327914	59,6749859	65,9338395	78,8818378
18,6553756	59,6982431	65,9626784	78,8904646
18,6829088	59,701934	65,9754629	78,9545987
18,7877283	59,7189691	65,9992174	78,9768331
19,0193966	59,7526043	66,0027782	78,9826764
19,1042984	59,8305254	66,0302533	79,0113246
19,1935477	59,8745436	66,0437403	79,0572375
19,2171902	59,8900313	66,1009826	79,0574893
19,2190385	59,9175526	66,1246775	79,067811
19,374559	59,9342418	66,1429916	79,1598916
19,4303862	59,9367961	66,1430525	79,1637772
19,5112599	59,95536	66,2055895	79,1697963
19,5832608	60,1202728	66,2819368	79,1799096
19,5905151	60,1660641	66,3026702	79,1916175
19,7059539	60,1783292	66,3316476	79,2347597
19,8759634	60,1937347	66,3943559	79,2420218
20,1851296	60,2457472	66,4459057	79,2527491
20,3875927	60,2987353	66,4550015	79,2575308
20,4991475	60,3251811	66,4667218	79,2763462
20,5705868	60,3585542	66,6071952	79,3214093
20,7252156	60,4557576	66,6131854	79,3601577
20,7279497	60,5262752	66,6272034	79,3712823
20,7898031	60,5475991	66,6518034	79,4284599
21,0080765	60,5653631	66,7483634	79,4603987
21,0117815	60,7054474	66,7566856	79,4830771
21,0847076	60,7238153	66,8033724	79,5143388
21,2135283	60,7282984	66,8448123	79,5231122
21,2829904	60,7358976	66,9294066	79,5667066
21,2949882	60,7684276	66,9572541	79,6013942
21,3863662	60,7837971	66,9761859	79,6181595
21,4832035	60,8117593	67,0048319	79,6531391
21,6309471	60,8397751	67,0106598	79,6578512
21,6383935	60,8504565	67,0150302	79,6818083
21,7080607	60,9007204	67,0527771	79,6961293
21,7135499	60,9221996	67,0698515	79,7322971
21,7917548	60,9708018	67,0745126	79,7520643
21,8134233	60,9732966	67,1635123	79,7654287
21,8880751	60,9796113	67,1725884	79,8097186
21,9065936	61,0278278	67,256817	79,8696571
21,9451507	61,060656	67,261214	79,8820287
22,0514378	61,075442	67,2794897	79,940238
22,1390267	61,0930683	67,365227	80,0098242
22,1834757	61,10688	67,4261967	80,0279626
22,2946896	61,1237623	67,4341176	80,0583603

22,3858985	61,1828691	67,4449349	80,1113728
22,3977422	61,1925173	67,4550185	80,1173462
22,5903353	61,2045038	67,4578196	80,1331393
22,696323	61,2200205	67,459243	80,1529813
22,8393042	61,2758984	67,4718248	80,189321
22,917313	61,3055583	67,4871408	80,2106774
22,924822	61,3352217	67,4877416	80,2743668
22,9785198	61,4670644	67,5223849	80,3140109
23,068141	61,4861758	67,5605508	80,3416086
23,0682105	61,5242622	67,5690361	80,3668709
23,13705	61,541912	67,5930621	80,4148872
23,1843144	61,6154137	67,5994253	80,4333502
23,2050489	61,6404097	67,6435193	80,4955866
23,4186898	61,6676869	67,659196	80,5327601
23,4770211	61,7156461	67,8012747	80,556628
23,6662561	61,7404567	67,8521797	80,5787353
23,8865804	61,7452632	67,9361328	80,6005176
23,932783	61,7644985	67,9582531	80,6260749
23,9452609	61,8021013	67,9808001	80,6311498
24,0539473	61,8134398	68,0795293	80,64029
24,1017971	61,8313645	68,1690188	80,7027139
24,119107	61,8452047	68,1860352	80,7098122
24,1311594	61,8455908	68,2086191	80,7178212
24,1357368	61,8652666	68,2278025	80,7529156
24,1631773	61,8906675	68,243365	80,7680956
24,1639029	61,9052481	68,2543717	80,7775097
24,2056103	61,9576233	68,3489923	80,7924401
24,2307688	62,0009616	68,356198	80,8356768
24,2394228	62,0317532	68,3747641	80,8850029
24,3209902	62,0483539	68,3794831	80,9135602
24,4149221	62,0953772	68,5334467	80,9487263
24,4371695	62,1020227	68,6326516	80,9718262
24,4586894	62,1091288	68,6803468	80,9837053
24,4648549	62,1108779	68,7446063	81,0213435
24,5452843	62,1228832	68,7846096	81,0334695
24,6275169	62,1320262	68,7956714	81,0339481
24,7628128	62,1476319	68,8210407	81,0403859
24,7861418	62,159982	68,8296127	81,0522281
25,2042976	62,1662113	68,8555822	81,070894
25,2517595	62,1994337	68,8865389	81,0908222
25,279161	62,2377838	68,891791	81,1622502
25,3734014	62,2437085	68,9028697	81,1694607
25,9174661	62,2601938	68,9302165	81,1982605
25,9587421	62,299762	68,9597194	81,2192082
26,0635876	62,3160059	69,0174701	81,2664718
26,0738671	62,3354917	69,0235106	81,2664794
26,1025245	62,4732743	69,0491189	81,2757996

26,6008874	62,5137485	69,0842638	81,2872525
27,1193296	62,5384552	69,0938402	81,2949098
27,1209324	62,5683459	69,1163879	81,3422848
27,1667191	62,5762071	69,1222451	81,4393561
27,3347617	62,5923322	69,1277456	81,4826752
27,3493151	62,6629732	69,1328337	81,5523089
27,5814734	62,6848132	69,1493853	81,565631
27,6570363	62,6891108	69,1531315	81,5661408
27,6597986	62,7092379	69,1608807	81,5698607
27,6781759	62,7402706	69,1884171	81,6639748
27,7527871	62,7696096	69,2008218	81,7276066
27,7655553	62,807236	69,214206	81,7798582
27,8147474	62,812239	69,2806813	81,7919742
27,8999626	62,8411613	69,3375591	81,7983783
27,927904	62,8882162	69,3431534	81,8663557
27,934423	62,9278261	69,362066	81,895355
27,9925149	62,9298403	69,3992406	81,8999438
28,1907243	63,0127175	69,4032623	81,913855
28,2969658	63,0148703	69,4439784	81,9441988
28,3095429	63,0403148	69,5639923	81,9470191
28,8035358	63,0588251	69,5787625	81,9829005
28,8385559	63,1446181	69,5890438	81,9858495
29,1034645	63,2892257	69,6600762	81,9915554
29,1704649	63,3573614	69,6942224	82,0032732
29,1777936	63,3734709	69,7072471	82,0207384
29,2454626	63,3766269	69,7092224	82,0373936
29,3289312	63,3871431	69,7125597	82,1223371
29,3650664	63,406349	69,7284411	82,1374753
29,5218891	63,451351	69,743526	82,1471203
29,8688927	63,4550397	69,7506225	82,1758188
29,8911529	63,492928	69,7507662	82,221135
29,9375171	63,5592333	69,7809209	82,2911962
30,1001059	63,5614473	69,8164608	82,3038132
30,2013131	63,5874433	69,8202745	82,3079285
30,5861669	63,5890318	69,8377225	82,3185943
30,6040721	63,5919638	69,9048279	82,3686408
30,6400939	63,6513035	69,9225383	82,3769152
30,6598978	63,6918933	69,9247557	82,3895146
30,9322145	63,7215979	69,9377062	82,399862
31,2642921	63,7245573	69,9710338	82,4080151
31,2849026	63,736436	69,982965	82,4152834
31,2859538	63,7700636	69,9975144	82,4746754
31,3258585	63,7874103	70,0007973	82,5029801
31,343869	63,8002982	70,0224058	82,5032276
31,4490001	63,8625919	70,1299807	82,5161703
31,4856405	63,8645014	70,1452276	82,5491881
31,5134141	63,9542884	70,1471358	82,5494373

31,6137092	63,9667692	70,1502828	82,5634286
31,7312074	63,9794377	70,1514793	82,5769657
31,7328554	64,0144577	70,2090412	82,6172206
31,7911032	64,0235905	70,2117587	82,623239
31,8121184	64,0285473	70,2625729	82,6274764
31,8978186	64,0344379	70,2691956	82,6337072
31,9337386	64,0476941	70,2713422	82,6607657
31,9576164	64,0572957	70,2730196	82,7354283
32,0224351	64,0823457	70,3107382	82,7379232
32,1042242	64,1611402	70,3401285	82,7438213
32,4690108	64,1891019	70,3457814	82,7695231
32,5750932	64,1989365	70,3522782	82,8455173
32,7447601	64,2526196	70,3562074	82,8471632
32,8988227	64,2603195	70,3733655	82,9005282
33,0945936	64,2646739	70,4178617	82,9033026
33,1286227	64,2664024	70,4208098	82,9059594
33,2028511	64,2929549	70,4214074	82,9081756
33,3398791	64,3111338	70,4368493	82,9247374
33,4105872	64,3371036	70,4899945	82,936082
33,7217357	64,3452453	70,5033799	82,9829606
33,797226	64,3654667	70,5685832	82,9960957
33,9101416	64,3752497	70,5726638	82,9982028
34,0442072	64,381783	70,5980984	83,0284781
34,0715051	64,4109781	70,607461	83,0445514
34,0905843	64,4142443	70,6295523	83,0637101
34,0932479	64,492863	70,6589897	83,0691305
34,1317484	64,4928818	70,7255025	83,1100922
34,2081945	64,5014064	70,784647	83,1123918
34,2595437	64,5397816	70,8060955	83,2006169
34,371918	64,5523244	70,8067417	83,2014578
34,5076729	64,5689964	70,8426803	83,2183832
34,5840762	64,5808446	70,8559686	83,249263
34,5980416	64,6187564	70,8993927	83,2794723
34,6987246	64,6264789	70,9157587	83,307844
34,8565322	64,6304637	70,9237473	83,323048
34,8813262	64,677347	70,9440355	83,3623078
35,1302404	64,708557	70,9824025	83,3643232
35,1552589	64,7192402	70,9855211	83,3979528
35,1646062	64,7401679	71,0006095	83,4272106
35,1716649	64,7745317	71,0169382	83,435416
35,248041	64,7870499	71,0589777	83,4964809
35,2564322	64,7950485	71,0943899	83,5489214
35,295638	64,8039817	71,114688	83,54914
35,2981842	64,8136968	71,1178881	83,5501735
35,3716023	64,8242177	71,1318838	83,6069916
35,4024987	64,8588007	71,1404571	83,6186588
35,4602347	64,9047769	71,1556058	83,6241684

35,5278739	64,9187647	71,2499125	83,6438478
35,6311255	65,0083631	71,2539007	83,6694168
35,7149728	65,0292983	71,2787532	83,6763246
35,8017719	65,0608342	71,3446877	83,6879982
35,875292	65,1567964	71,4202198	83,6908728
35,8755051	65,1637033	71,4609358	83,7842186
35,9055853	65,1851856	71,4705632	83,8145511
36,2975554	65,2095279	71,6388867	83,8766499
36,8351323	65,2119329	71,643649	83,9075176
36,8401206	65,2515029	71,6668782	83,9137266
36,92494	65,252462	71,6703897	83,9296758
36,9392822	65,256366	71,6807816	83,9311469
37,1002749	65,2858088	71,6900207	83,938929
37,1556824	65,2905317	71,7066475	83,9735255
37,1944601	65,3055116	71,7118508	83,9928091
37,2820277	65,3086838	71,7340932	84,0559702
37,2999841	65,390256	71,735111	84,0737119
37,4704844	65,3960653	71,7781677	84,0939434
37,4978395	65,4489069	71,8371464	84,0979443
37,5180232	65,457904	71,8730142	84,1163327
37,5363696	65,462802	71,9148619	84,1271061
37,7853883	65,4713807	71,919338	84,1515164
37,8114863	65,4909084	71,9230258	84,1624367
37,8146753	65,4966056	71,9240834	84,1645399
37,8225877	65,5173779	71,9944345	84,1685212
37,9303267	65,5498889	72,0037884	84,1727949
38,0403094	65,5628898	72,0119665	84,1891622
38,0532888	65,585271	72,0398205	84,22079
38,2150384	65,6125915	72,0818862	84,2233861
38,3645052	65,6170796	72,1013005	84,2301696
38,6062539	65,6299052	72,1039903	84,2464265
38,8651095	65,6335908	72,1923595	84,2638697
39,0513024	65,6508416	72,1987264	84,2979235
39,0540164	65,6511701	72,2066356	84,3189439
39,0904282	65,6628565	72,2157482	84,3220639
39,1519556	65,6734463	72,2330304	84,3297405
39,2304981	65,6790054	72,2728715	84,3413534
39,2442407	65,6896007	72,2939472	84,3569677
39,2950176	65,7508089	72,2969199	84,3639029
39,3583939	65,7805366	72,3597714	84,3749833
39,5964033	65,7895002	72,3662161	84,3864922
39,6455634	65,8525399	72,3776268	84,3929961
39,6713897	65,8541088	72,386221	84,4074071
39,7766558	65,8800741	72,3973061	84,4442442
39,8369903	65,887829	72,461675	84,4537058
39,8735642	65,9628041	72,4778015	84,5189109
39,9141809	66,0103343	72,5119826	84,5277492

39,9875402	66,0128655	72,5274304	84,5761456
40,173487	66,0411902	72,5404611	84,6038535
40,1812996	66,0419105	72,5493642	84,6049299
40,2288509	66,0421691	72,5947252	84,607859
40,2601	66,0611129	72,6031047	84,6154998
40,3254971	66,0735644	72,60974	84,6439579
40,4227087	66,1123862	72,6226912	84,6758028
40,4259519	66,1181954	72,653166	84,6787465
40,4644246	66,1328823	72,7026548	84,7144237
40,4757818	66,1595975	72,7244232	84,7291522
40,5188472	66,1856828	72,7428085	84,7391476
40,5388664	66,2214405	72,7737436	84,7429997
40,5476899	66,2514797	72,7771305	84,7627142
40,5515701	66,2589556	72,7883156	84,8462028
40,7219991	66,2762639	72,8113952	84,8581732
40,7696755	66,2779171	72,8873327	84,8765361
40,7891358	66,3266919	72,904345	84,9036902
40,8215367	66,3288581	72,9093961	84,9202278
40,8278884	66,3358414	72,9474421	84,9229526
40,8957544	66,3395817	72,9717593	84,926606
41,0523512	66,3674378	72,9730681	84,9700305
41,1065626	66,3820784	72,9827253	84,9726281
41,3325928	66,3931385	73,0181428	84,9909472
41,409589	66,4103582	73,0197747	84,9931609
41,5209138	66,4265281	73,0461146	85,0185805
41,6943257	66,4694363	73,0636034	85,0211188
41,8347567	66,4716959	73,0665072	85,0269966
41,8872425	66,5103602	73,0694739	85,036278
41,8909842	66,5243968	73,0788237	85,037934
42,2469954	66,5277155	73,0966595	85,0441799
42,2792967	66,5644641	73,1367599	85,0539757
42,2917558	66,5754453	73,204327	85,0875317
42,3334586	66,5810147	73,2361521	85,0984712
42,3835975	66,5999429	73,2517925	85,1303668
42,4183886	66,6163582	73,2902437	85,136463
42,5045794	66,6300365	73,2913896	85,1513305
42,5147091	66,6351406	73,3183063	85,1537473
42,5169196	66,6599087	73,4024971	85,1925597
42,7332034	66,7317205	73,4299995	85,1997983
42,8063616	66,7625058	73,4363695	85,2103067
42,9758699	66,7724808	73,4811062	85,2230181
43,0033276	66,799072	73,5354206	85,2427091
43,1577263	66,8253348	73,5408	85,279751
43,2335975	66,8817347	73,5527657	85,2858662
43,2687001	66,909423	73,5635177	85,2975531
43,3468736	66,9242467	73,5914146	85,3268435
43,3506317	66,9819759	73,6355844	85,330803

43,440125	67,0119334	73,6415415	85,3775715
43,4768983	67,0181422	73,650281	85,3790745
43,5937021	67,0501312	73,7368874	85,3877128
43,6562171	67,0592222	73,7392419	85,3899151
43,8360928	67,074421	73,8417308	85,3960916
43,8990296	67,0921746	73,8717393	85,4525814
43,9663937	67,1063876	73,9496363	85,4900386
43,9892181	67,1474247	73,9739325	85,4908636
43,9970617	67,1508335	73,9808935	85,5040398
44,0698506	67,1870014	73,9848435	85,5247141
44,1471077	67,1878324	74,0895476	85,5429926
44,1687572	67,1924271	74,1130599	85,5562801
44,326809	67,2136678	74,1136924	85,5675048
44,3616791	67,2183671	74,1167796	85,6055731
44,4710442	67,2766228	74,1460408	85,6293015
44,5568783	67,2791334	74,169127	85,6307037
44,6148662	67,3173586	74,1934136	85,6353268
44,7103609	67,3226633	74,2213495	85,6740861
44,7197028	67,3364844	74,2395253	85,7012525
44,7198719	67,357344	74,263296	85,7483739
44,8997783	67,3632447	74,2706489	85,7492231
45,0509443	67,3706048	74,2908333	85,7642277
45,0775712	67,371149	74,3280968	85,7659455
45,2361301	67,3773271	74,3604194	85,7960519
45,2734913	67,4223659	74,366661	85,8015541
45,332375	67,4251265	74,3751743	85,8132059
45,4340284	67,4370903	74,3757006	85,826961
45,4366631	67,4782631	74,4666861	85,8531933
45,4872027	67,4942824	74,501424	85,8681831
45,6612564	67,5145047	74,552889	85,8686003
45,6866732	67,515707	74,5545376	85,8718952
45,7717979	67,5781186	74,6032457	85,8831811
45,7730491	67,5914969	74,6372311	85,8979871
45,7924077	67,6094581	74,6392743	85,9064191
45,8019369	67,6115567	74,6477319	85,9163511
45,8215369	67,6203603	74,6824338	85,9396081
45,8231842	67,6352199	74,771836	85,9408934
45,9382648	67,6375747	74,7745613	85,9617171
46,1230624	67,6508291	74,8423331	86,0053418
46,1339496	67,664854	74,8469496	86,0667616
46,2336983	67,6869653	74,8830043	86,068088
46,2930664	67,6905662	74,9925708	86,0716524
46,4013442	67,6990393	74,9977421	86,0808037
46,520108	67,7076454	75,0038644	86,1052789
46,5918735	67,7389156	75,0168963	86,1141142
46,6366584	67,7527929	75,0227415	86,2400867
46,7006289	67,8471923	75,0229019	86,2463969

46,7008446	67,8572834	75,0299761	86,2486712
46,7327969	67,8704454	75,0328285	86,2500476
46,7450964	67,8824621	75,0391578	86,3178287
46,7628356	67,911196	75,097057	86,3243544
46,8214696	67,9299719	75,0985652	86,4097139
46,8504621	68,0178954	75,1511156	86,4112744
46,8593993	68,0218384	75,1561119	86,4570732
46,8661918	68,0240769	75,1694619	86,5009372
46,8695069	68,0474089	75,1698645	86,5488024
46,9137952	68,125307	75,2385989	86,5641307
46,9674748	68,1630908	75,2419557	86,5840604
47,0055658	68,1845957	75,253987	86,599915
47,0495951	68,1885186	75,2583277	86,6023425
47,0841307	68,2041899	75,2766418	86,61199
47,3034062	68,2775976	75,2893925	86,6185536
47,3757439	68,2835187	75,3102577	86,6258159
47,3933366	68,3460075	75,3108116	86,6306199
47,5222587	68,3556716	75,3159455	86,6531545
47,569663	68,4397825	75,3163866	86,6550334
47,6472764	68,4435231	75,3553078	86,689708
47,6685194	68,5256821	75,3964118	86,6904737
47,725726	68,5387361	75,4125801	86,6948423
47,8133314	68,5593242	75,4264028	86,7140883
47,8869122	68,5691669	75,4386503	86,717063
47,8921838	68,612419	75,4416701	86,8276109
47,8994165	68,65607	75,6271445	86,8395467
47,9083426	68,6639716	75,6483574	86,8675641
47,9330623	68,6842442	75,6918263	86,8708681
47,956847	68,7751294	75,6979994	86,9167903
47,9823893	68,7798279	75,7211332	86,9432934
48,0354309	68,7859133	75,7404243	86,9842285
48,0504854	68,7985406	75,8152985	86,9865376
48,0906393	68,8398499	75,8196361	86,9925026
48,1041203	68,8675851	75,8199935	87,0059696
48,1871361	68,9408836	75,8335009	87,0354925
48,2023841	69,0152792	75,8361042	87,0936273
48,2703207	69,0166549	75,8545985	87,1274289
48,2892332	69,0233624	75,9143253	87,1356008
48,2915146	69,0424073	75,9423765	87,1375979
48,3035958	69,0546638	75,9532136	87,1653326
48,3093704	69,0878606	76,0001915	87,1782903
48,3366432	69,0890258	76,0304878	87,1873637
48,3971584	69,0924889	76,0455921	87,1984941
48,4111275	69,1311304	76,0502547	87,231745
48,4526438	69,1538789	76,1026007	87,2349104
48,4863602	69,1573164	76,1161664	87,255126
48,5082639	69,1764883	76,1178146	87,2651401

48,6416478	69,1792101	76,1251715	87,2824766
48,64754	69,1973093	76,1659377	87,2899379
48,685247	69,2038944	76,1873363	87,2918163
48,742625	69,2387322	76,1952834	87,325393
48,7560528	69,2810877	76,2047463	87,3429578
48,786864	69,2820811	76,220896	87,3842191
48,8173455	69,3005843	76,25611	87,390382
48,9169231	69,3008592	76,287985	87,3904416
48,9988284	69,3241968	76,3280439	87,3960993
49,0729732	69,3289801	76,3313188	87,4097864
49,1250546	69,3335502	76,3546278	87,4194307
49,1297429	69,3560504	76,3734946	87,4577012
49,1933986	69,3912762	76,423306	87,462005
49,2199731	69,4237794	76,4453386	87,46471
49,290324	69,4386615	76,469509	87,4895201
49,4232923	69,4449365	76,4778908	87,5032452
49,6233644	69,4449402	76,5063487	87,5529772
49,7015522	69,4895126	76,5726948	87,584677
49,8264753	69,4934318	76,5986314	87,5923089
49,8355603	69,5147731	76,599355	87,5980093
49,9841939	69,5257551	76,6135734	87,6268499
50,0027174	69,5365307	76,6567237	87,6290186
50,146818	69,5473902	76,657302	87,63251
50,2120895	69,5715021	76,6582487	87,6713647
50,419131	69,5716588	76,6607912	87,6745158
50,4994938	69,6032329	76,7136702	87,6821612
50,8417535	69,605608	76,7399596	87,6986996
50,8969558	69,6187258	76,8402448	87,7030862
50,9555201	69,6264591	76,9187263	87,7122569
50,9748886	69,6303311	76,928399	87,7139434
51,1652484	69,6394111	76,9875503	87,7196057
51,358833	69,6514948	77,0249864	87,7220948
51,4009182	69,7506424	77,0285386	87,7684388
51,4932659	69,7742739	77,0773968	87,7837061
51,52935	69,8432322	77,0854463	87,8080844
51,6004547	69,927714	77,0883563	87,8117816
51,6647269	69,9432696	77,3352834	87,8258229
51,690351	69,9702544	77,4240085	87,8436815
51,7313316	69,9892746	77,4305984	87,8722143
51,7542272	70,0554209	77,4662144	87,8796493
51,7622032	70,200264	77,5017546	87,8948502
51,7726155	70,2395008	77,5055342	87,9003324
52,0017753	70,2534043	77,5263407	87,9093337
52,0081955	70,2573313	77,5266907	87,9155903
52,2611698	70,2925824	77,5536375	87,9273333
52,2926299	70,3022428	77,5563723	87,93048
52,4389008	70,3259148	77,5665769	87,9352008

52,4535764	70,3319303	77,6131262	87,9356153
52,5353897	70,3366728	77,7168808	87,9772514
52,6640574	70,3446701	77,7864678	87,9835376
52,7429226	70,3500511	77,8810249	87,9970415
52,7494042	70,3840568	77,9637336	87,9982285
52,7584895	70,5219381	77,9748836	87,9988983
52,8355521	70,6289571	78,0266119	88,0013757
52,9715201	70,6542108	78,0949224	88,0453805
52,9995077	70,6736865	78,0971005	88,0733693
53,0784135	70,6874106	78,1259637	88,0861262
53,1124702	70,6888442	78,2106053	88,0914742
53,1828388	70,7054685	78,3049696	88,106521
53,3177188	70,7507281	78,3197115	88,1185266
53,3351976	70,7908973	78,3378437	88,1484112
53,4709372	70,8814858	78,3727225	88,1653775
53,4950105	70,8952037	78,3779327	88,1760661
53,5141742	70,9200196	78,3993539	88,1986819
53,5485568	71,0051231	78,4358153	88,2159964
53,5580889	71,009836	78,486128	88,2199539
53,6074521	71,0909449	78,5103637	88,2333301
53,9924625	71,1047097	78,5183931	88,2687636
54,0201427	71,1204424	78,5539651	88,2940206
54,0594976	71,1249715	78,5603594	88,2993339
54,0643202	71,1536208	78,5783478	88,3183986
54,1142751	71,2542239	78,6143969	88,332052
54,1808615	71,3287949	78,6241424	88,384409
54,273937	71,336157	78,6302376	88,3873184
54,3602191	71,3431475	78,631035	88,3887325
54,6871251	71,3864174	78,6694686	88,4395472
54,819856	71,4591832	78,6696726	88,4485068
54,8959716	71,5261954	78,684583	88,4774524
55,3363686	71,5740681	78,7665519	88,506099
55,3834439	71,5843619	78,7974751	88,5308482
55,5592408	71,6386471	78,8371665	88,5352015
55,722752	71,6481208	78,8826271	88,549122
55,8745206	71,7100161	78,9343015	88,550531
56,0166502	71,7261179	78,9652256	88,5538974
56,0172031	71,7441161	78,9755946	88,5547919
56,1570128	71,7503791	79,015417	88,5953659
56,4366786	71,773208	79,1028358	88,5987738
56,5270101	71,7750288	79,1701151	88,6002291
56,5627867	71,8084623	79,1786907	88,6256165
56,5813184	71,8642725	79,2066659	88,6333844
56,6007756	71,8658912	79,2123657	88,6357847
56,7399002	71,9350361	79,2171319	88,6451087
56,9983113	71,9518574	79,242802	88,6473617
57,2204475	71,9635023	79,2692979	88,649392

57,2359183	72,0716211	79,2965679	88,6505536
57,2935675	72,1181211	79,3023267	88,7324009
57,3005554	72,1400777	79,3410943	88,7581341
57,3796585	72,1519534	79,3538843	88,7678215
57,489685	72,1805286	79,3691626	88,7838462
57,5898059	72,2064774	79,3728809	88,7991401
57,6617486	72,261885	79,3849406	88,8002964
57,7479533	72,2767166	79,4087622	88,8100051
57,7890505	72,3126658	79,4156666	88,8108665
57,7949381	72,3441537	79,4185118	88,8198354
57,8185039	72,4185355	79,4473717	88,8597007
57,865288	72,4300234	79,5237923	88,8781976
57,9535705	72,5039188	79,6163964	88,8979425
58,1344603	72,5106477	79,6388861	88,9422413
58,7870219	72,5750571	79,7576103	88,9489771
58,8564997	72,5852126	79,79729	88,9691647
59,2048564	72,6750943	79,8654413	88,9700584
59,2954443	72,6751867	79,8872976	88,9857343
59,4220575	72,7189634	79,9435712	89,0008134
59,5081116	72,7593647	79,9606014	89,0029643
59,5425818	72,8115168	79,9916682	89,0299777
59,6148034	72,8887182	80,0153973	89,0711217
59,6425355	72,8909088	80,0349285	89,0759345
59,6857969	72,8953649	80,0572785	89,0965928
59,711547	72,933711	80,0629999	89,1005516
59,7319554	72,9878982	80,150526	89,1166864
59,7414933	73,0013728	80,1615402	89,146499
59,7448213	73,023836	80,1962455	89,1541887
59,7682487	73,0719998	80,2260233	89,1713523
59,7772638	73,1160204	80,3119873	89,2141741
59,8221623	73,1841044	80,3290007	89,2290483
59,8595753	73,2016466	80,340349	89,2318927
60,0005956	73,2862708	80,3443567	89,2331769
60,0421168	73,3075071	80,409704	89,2424607
60,2402846	73,3475207	80,4432523	89,2673952
60,2867964	73,3593554	80,5969603	89,3167325
60,4249296	73,3711432	80,637214	89,3288343
60,5030836	73,4539526	80,6650339	89,3377528
60,6190663	73,4810357	80,7290923	89,3887426
60,6290148	73,5171379	80,7388495	89,3949725
60,8052329	73,6316609	80,7669364	89,4020793
60,8146381	73,7064249	80,8127832	89,4394081
60,8544242	73,7143448	80,9284717	89,4569053
60,9189314	73,7163358	80,9311168	89,4679841
60,9230542	73,7318089	80,9744211	89,4860755
61,0359569	73,7629832	80,9814885	89,4873706
61,1395373	73,782829	81,0214526	89,5005665

61,5598315	73,7879643	81,0270162	89,5024134
61,6810195	73,8222637	81,0510301	89,5385231
61,8442736	73,8605799	81,0576122	89,5557727
62,0282145	73,9029121	81,0724881	89,5574699
62,0354134	73,9273665	81,1119285	89,5780231
62,0458441	73,9777012	81,1131171	89,5959175
62,1394946	74,0156095	81,1416876	89,6336713
62,4283557	74,0500838	81,1590929	89,6477122
62,5976701	74,1030526	81,1596028	89,6995961
62,6751795	74,1040624	81,171043	89,726307
62,8480684	74,2588452	81,1882023	89,7307728
63,1664705	74,2930804	81,1946765	89,7546125
63,3584718	74,6334239	81,2403467	89,7679632
63,43954	74,6440055	81,3491221	89,7828413
63,4856788	74,6617902	81,3499953	89,8011471
63,5381027	74,713201	81,3607033	89,8056492
63,6024223	74,762826	81,3992277	89,8111249
63,7692054	74,762904	81,4826216	89,8117292
63,8007301	74,7812994	81,5017569	89,8130959
63,9317072	74,8293591	81,5663664	89,8148587
63,9543286	74,8851076	81,5707655	89,8268873
63,969338	74,890568	81,5711137	89,8546954
64,0926212	74,9908006	81,7300153	89,8676088
64,2634058	74,9910706	81,7598015	89,8687826
64,4480498	75,0234237	81,7672413	89,8798855
64,6651446	75,1049444	81,8318425	89,9213271
64,8426355	75,1084777	81,8936023	89,9302059
64,851739	75,1418934	82,0988411	89,9379007
64,8877545	75,1487579	82,1032816	89,9604271
64,9096798	75,1627417	82,1134839	89,9608714
64,937962	75,1781264	82,1167536	89,9775895
65,0363867	75,1923228	82,1387433	89,9778545
65,0403029	75,2641289	82,1521208	89,982745
65,0969156	75,2917302	82,1526096	89,9917969
65,145918	75,3160394	82,2044659	90
65,1808869	75,3448015	82,2452496	90
65,3038967	75,3659956	82,2587291	90
65,3617727	75,4005086	82,2612139	90
65,5519987	75,4382804	82,2673291	90
65,5849114	75,4922872	82,2762458	90
65,7987485	75,5070991	82,2856065	90
65,9301615	75,5247126	82,2933386	90
65,993344	75,5939902	82,4351844	90
66,1209946	75,5954933	82,5232734	90
66,1682182	75,6536222	82,5280469	90
66,1805799	75,6842937	82,572015	90
66,2649953	75,7791603	82,6025426	90

66,2756399	75,841692	82,6126535	90
66,3613437	75,9291953	82,6208895	90
66,4511042	75,9349453	82,6250817	90
66,4868651	76,1151377	82,6267162	90
66,5012158	76,1322403	82,64024	90
66,7268513	76,1743288	82,6599746	90
67,0044662	76,1959993	82,696156	90
67,0411973	76,2059561	82,7004534	90
67,0986171	76,2112294	82,8457157	90
67,1947894	76,253699	82,8468815	90
67,3768968	76,3337371	82,8553974	90
67,381803	76,375324	82,871423	90
67,4681685	76,3833944	82,9047758	90
67,6019809	76,3838756	82,9248303	90
67,7182876	76,450408	83,0768787	90
67,8020548	76,4665995	83,083731	90
67,8952006	76,4835899	83,0853626	90
68,0446541	76,5024596	83,1023158	90
68,1115888	76,5779355	83,1030498	90
68,3035995	76,6799956	83,1807856	90
68,3182207	76,6848522	83,2552558	90
68,3193786	76,7815261	83,2930063	
68,6460745	76,785433	83,3060931	
68,6797512	76,8903223	83,3377787	
68,6986043	76,9633997	83,3404268	
68,8930876	76,9774146	83,3791872	
69,0753229	77,0234938	83,3827112	
69,442171	77,1016757	83,3836635	
69,5683385	77,1215964	83,4250874	
69,7574437	77,1561704	83,4549076	
69,809039	77,1825528	83,6135794	
69,8565681	77,3268429	83,6684213	
69,9787961	77,3380423	83,7270117	
70,0737049	77,4141091	83,7381405	
70,1232961	77,4468116	83,7503942	
70,155906	77,7511621	83,7655017	
70,1823611	77,9212189	83,8061858	
70,2925797	77,9276105	83,8791317	
70,3102315	77,9994775	83,8796003	
70,3976641	78,0736567	83,8823201	
70,4921368	78,1052553	83,9070418	
71,0369724	78,1567331	83,9156938	
71,0523577	78,157502	83,9847145	
71,685021	78,1638079	83,9867304	
71,7169333	78,1837465	83,9961941	
71,857567	78,3527807	84,0711253	
71,9006961	78,387566	84,0858115	

71,9778944	78,4133902	84,14083
71,9968042	78,5098342	84,16314
72,0040849	78,5300978	84,1760403
72,0552587	78,5360266	84,2139345
72,2163647	78,5680178	84,2820611
72,2932343	78,5722925	84,301474
72,3098865	78,5748371	84,3335526
72,3399024	78,6051521	84,3517466
72,4042862	78,6576013	84,4327317
72,4483667	78,7100198	84,7053233
72,5709495	78,9277796	84,7959022
72,6950185	78,9983428	84,7971215
72,8436441	79,0771367	84,8387655
73,0493224	79,1556539	84,8465391
73,2949439	79,3021952	84,8844717
73,4341724	79,4824352	84,8954658
73,4480818	79,4873424	84,9124176
73,5485021	79,4937006	84,9163738
73,5487548	79,5756493	85,0350983
73,641209	79,5889115	85,035215
73,6578005	79,6808477	85,0778964
73,6778685	79,7831602	85,1110941
73,715115	79,8644954	85,1945122
73,8135234	79,8860713	85,1967968
74,058967	80,0034693	85,2952289
74,3347094	80,0213873	85,3444765
74,4228076	80,1163662	85,3591893
74,5756938	80,1235215	85,3768119
74,6739959	80,2021268	85,383918
74,7070818	80,2381221	85,3906516
74,7071541	80,3081117	85,400263
74,7152309	80,3212348	85,4040427
74,776452	80,3483825	85,4388111
74,8506503	80,3984998	85,4951813
74,9086118	80,4465399	85,5163052
74,9420792	80,5162927	85,5178971
75,0257578	80,5178802	85,6480484
75,0311279	80,6320094	85,6601511
75,0904684	80,6356398	85,6892105
75,1540333	80,6926743	85,7368907
75,2166785	80,7311697	85,7595741
75,2488522	80,8053896	85,7791655
75,661085	80,9657215	85,8283783
75,8166649	81,0180192	85,8769608
75,8876823	81,0663712	85,9605254
75,9188619	81,0679343	86,035449
75,9931117	81,1528726	86,0586454

76,2119743	81,367932	86,0840422
76,2486466	81,4272406	86,1044775
76,2951508	81,4977921	86,1712267
76,3032044	81,4981676	86,2266445
76,3189508	81,5864111	86,3032828
76,3481495	81,6366545	86,3038455
76,4973113	81,6663924	86,3346232
77,0617753	81,7116783	86,3962862
77,2519064	81,7296023	86,4459325
77,2528741	81,8274599	86,5166492
77,3734043	82,2262868	86,5425033
77,4453907	82,426443	86,5504223
77,4987553	82,636555	86,5840164
77,5883729	82,6406206	86,5854738
77,6587626	82,7451748	86,6734349
77,7422058	82,8169816	86,6996472
77,7985148	82,8288642	86,720357
77,920023	82,8406678	86,7207561
77,9899271	82,8614121	86,7211804
78,0503379	83,0935254	86,756244
78,0799435	83,2085683	86,7692262
78,0969801	83,4815374	86,7805999
78,3098134	83,4862246	86,7860428
78,3387089	83,5313447	86,876808
78,3627968	83,6340634	86,8818156
78,4806237	83,6565862	86,928396
78,5060489	83,7254283	86,9562727
78,5402995	83,7743835	87,1335948
78,5538803	83,8865859	87,2226662
78,5725573	83,8914964	87,2896694
78,5755408	83,9090252	87,2943104
78,648761	83,9302978	87,3212516
78,7013421	83,9490093	87,3230347
78,8154223	83,9643783	87,3653581
78,8566995	83,9721799	87,3808052
78,9832094	83,9819973	87,3829095
79,0111576	84,0603017	87,4009403
79,1320191	84,2477122	87,4201705
79,1529655	84,4404957	87,4419321
79,2415289	84,4807732	87,4694583
79,4591097	84,4856881	87,5038741
79,5675522	84,6211079	87,7953224
79,5897594	84,6477705	87,8651536
79,7425704	84,7290244	87,8810261
80,0014844	84,7984236	87,9352587
80,0410413	84,8416402	87,979513
80,2224369	84,851976	87,9966295

80,3201596	84,875696	88,0652018
80,3851491	85,2129621	88,0800136
80,4682711	85,3086944	88,1822709
80,4806967	85,3284848	88,1920932
80,8452967	85,3689264	88,2439441
81,1142153	85,4916556	88,2584288
81,2302385	85,5119689	88,2667807
81,2371815	85,6115444	88,3808657
81,411332	85,6295073	88,3909099
81,4745538	85,6413973	88,4841784
81,5483276	85,7024807	88,4950112
81,6824161	85,7051175	88,5182245
82,014104	85,7269666	88,5426518
82,1298211	85,7343012	88,5909872
82,3047718	85,7732721	88,6125218
82,3784152	85,8456196	88,6144504
82,4909832	86,0012051	88,6373519
82,6139767	86,0454127	88,6490701
82,6174648	86,0668683	88,6743426
82,6572026	86,2556423	88,6760842
82,6833899	86,3061844	88,706947
82,917073	86,341725	88,7358114
83,0856275	86,3955057	88,7960708
83,3140676	86,404222	88,8036549
83,3501824	86,4787381	88,8121581
83,4345706	86,4993214	88,8950921
83,553071	86,5779446	88,9334007
83,6747898	86,5781672	88,9510014
83,6889981	86,5897365	88,9805532
83,6955007	86,7725869	89,0044898
83,7303254	86,7862598	89,0239042
83,8130169	86,8061968	89,0393731
83,8326999	86,8139389	89,0423537
84,1896815	86,8468692	89,0428363
84,2431603	86,8517091	89,112288
84,5561239	86,88554	89,1384877
84,5965499	86,9109703	89,1569937
84,6622713	87,1262648	89,160792
84,9031649	87,3360113	89,1865762
85,1168963	87,3853634	89,2550464
85,239944	87,5455608	89,2711209
85,4183257	87,5522627	89,2954121
85,4522995	87,5949834	89,3266519
85,5673191	87,6143201	89,3521919
85,6866075	87,775084	89,3592354
85,739215	87,7804395	89,3719181
85,7573861	87,8262982	89,3816699

85,7808525	87,9355582	89,4322314
85,8175213	87,9546838	89,4856799
85,8537414	88,0890559	89,4857298
86,1454596	88,1919177	89,5044648
86,1620743	88,2304865	89,5122266
86,391619	88,3853038	89,5904792
86,440025	88,4923879	89,6614466
86,488491	88,512657	89,7052239
86,8427256	88,5264445	89,7362798
86,8946224	88,5802639	89,7695929
86,9055339	88,6620523	89,780806
87,049811	88,6764943	89,7925449
87,0764418	88,7492775	89,8006844
87,1470172	88,8253517	89,8010808
87,1997885	89,0147838	89,8038455
87,2920827	89,1227198	89,8169975
87,3032459	89,1417952	89,9406753
87,3436629	89,1841583	89,9534657
87,3453637	89,1960912	90
87,3978863	89,2283196	90
87,4870757	89,2506932	90
87,5439874	89,319361	90
87,81932	89,3904878	90
87,8443105	89,4223187	90
87,8597636	89,4795339	90
88,0662864	89,6045514	90
88,1431317	89,6636969	90
88,2904268	89,7221242	90
88,3610398	89,7534157	90
88,5636679	89,7953532	90
88,6610757	89,948532	90
88,6636016	90	90
88,868185	90	90
88,9948371	90	90
89,0777066	90	90
89,248605	90	90
89,3226058	90	90
89,3950216	90	90
89,4986975		
89,6993435		
89,7104315		
89,7333526		
89,7357427		
89,769775		
89,8087061		
89,8661127		
89,942505		

89,9944843
90
90
90
90
90
