# Supplementary material for: Cyclic stretch induces autophagy-mediated focal adhesion remodeling and activates mitochondria
Source: Life Sci Alliance. 2026 Feb 5;9(4):e202503347. doi: 10.26508/lsa.202503347 (PMC12877405; doi:10.26508/lsa.202503347)
Supplement: Supplementary file 2 [file LSA-2025-03347_SdataF2.pdf]

Source data Figure 2B

Absolute numbers of LC3B-spots/cell

Unstr. - (a)	Unstr.+CQ (b)	10 min str. - (c)	10 min str. +CQ (d)	30 min str. - €	30 min str. +CQ (f)
1,17	23,40	2,11	71,91	1,25	36,86
1,17	23,40	2,11	71,91	0,71	36,86
1,17	23,40	2,11	71,91	0,71	36,86
1,33	23,40	2,11	71,91	0,71	36,86
1,33	23,40	2,11	71,91	0,71	74,40
1,33	23,40	2,11	71,91	0,71	74,40
1,33	23,40	9,14	71,91	0,71	74,40
1,33	50,20	9,14	71,91	0,71	74,40
1,33	50,20	9,14	63,86	4,33	74,40
1,33	50,20	9,14	63,86	4,33	55,40
1,33	50,20	9,14	63,86	4,33	55,40
1,33	50,20	9,14	63,86	4,33	55,40
1,33	121,33	9,14	63,86	4,33	55,40
1,33	121,33	5,50	63,86	4,33	55,40
1,33	121,33	5,50	63,86	3,17	37,22
0,56	55,67	5,50	42,13	3,17	37,22
0,56	55,67	5,50	42,13	3,17	37,22
0,56	55,67	4,80	42,13	3,17	37,22
0,56	55,67	4,80	42,13	3,17	37,22
0,56	55,67	4,80	42,13	3,17	37,22
0,56	55,67	4,80	42,13	1,80	37,22
0,56	55,00	4,80	42,13	1,80	37,22
0,56	55,00	3,20	42,13	1,80	37,22
0,56	55,00	3,20	57,57	1,80	65,25
0,60	55,00	3,20	57,57	1,80	65,25
0,60	55,00	3,20	57,57	2,38	65,25
0,60	55,00	3,20	57,57	2,38	65,25
0,60	55,00	3,00	57,57	2,38	30,50
0,60	28,38	3,00	57,57	2,38	30,50
0,50	28,38	3,00	57,57	2,38	30,50
0,50	28,38	3,00	47,75	2,38	30,50
0,50	28,38	1,00	47,75	2,38	30,50
0,50	28,38	1,00	47,75	2,38	30,50
0,50	28,38	1,00	47,75	3,43	47,14
0,50	28,38	1,00	47,75	3,43	47,14
0,50	28,38	1,60	47,75	3,43	47,14
0,50	47,29	1,60	47,75	3,43	47,14
0,33	47,29	1,60	47,75	3,43	47,14
0,33	47,29	1,60	45,86	3,43	47,14
0,33	47,29	1,60	45,86	3,43	47,14
0,33	47,29	1,33	45,86	2,60	63,29
0,33	47,29	1,33	45,86	2,60	63,29
0,33	47,29	1,33	45,86	2,60	63,29
0,33	39,75	1,33	45,86	2,60	63,29

0,33	39,75	1,33	45,86	2,60	63,29
0,33	39,75	1,33	82,20	5,75	63,29
1,44	39,75	1,43	82,20	5,75	63,29
1,44	39,75	1,43	82,20	5,75	66,29
1,44	39,75	1,43	82,20	5,75	66,29
1,44	39,75	1,43	82,20	3,75	66,29
1,44	39,75	1,43	75,33	3,75	66,29
1,44	84,11	1,43	75,33	3,75	66,29
1,44	84,11	1,43	75,33	3,75	66,29
1,44	84,11	6,33	75,33	4,75	66,29
1,44	84,11	6,33	75,33	4,80	53,63
0,00	84,11	6,33	75,33	4,80	53,63
0,00	84,11	4,80	34,33	4,80	53,63
0,00	84,11	4,80	34,33	4,80	53,63
0,00	84,11	4,80	34,33	4,80	53,63
0,00	84,11	4,80	34,33	4,80	53,63
1,00	80,60	3,00	34,33	4,80	53,63
1,00	80,60	3,00	34,33	4,80	53,63
1,00	80,60	3,00	54,29	4,80	245,60
1,00	80,60	3,00	54,29	4,80	245,60
1,00	80,60	2,70	54,29	4,80	245,60
2,33	100,75	2,70	54,29	4,80	245,60
2,33	100,75	2,70	54,29	2,80	245,60
2,33	100,75	2,70	54,29	2,80	246,00
2,33	100,75	2,70	54,29	2,80	246,00
2,33	85,40	2,70	225,40	2,80	246,00
2,33	85,40	2,80	225,40	2,80	246,00
0,67	85,40	2,80	225,40	19,50	246,00
0,67	85,40	2,80	225,40	19,50	236,20
0,67	85,40	2,80	225,40	19,50	236,20
0,67	90,67	2,50	129,25	19,50	236,20
0,67	90,67	2,50	129,25	3,30	236,20
0,67	90,67	2,50	129,25	3,30	236,20
0,67	66,00	2,50	129,25	3,30	195,20
0,67	66,00	5,80	129,25	2,50	195,20
0,67	66,00	5,80	129,25	2,50	195,20
1,14	66,00	5,80	129,25	2,50	195,20
1,14	104,17	5,80	129,25	2,50	195,20
1,14	104,17	4,80	259,50	1,20	139,00
1,14	104,17	4,80	259,50	1,20	139,00
1,14	104,17	4,80	259,50	1,20	139,00
1,14	104,17	4,80	259,50	1,20	139,00
1,14	104,17	7,90	73,38	1,20	139,00
0,89	90,80	7,90	73,38	2,50	135,50
0,89	90,80	7,90	73,38	2,50	135,50
0,89	90,80	7,90	73,38	2,50	135,50
0,89	90,80	7,90	73,38	2,50	135,50

0,89	90,80	7,90	73,38	2,50	135,50
0,89	260,33	7,90	73,38	2,50	135,50
0,89	260,33	13,70	73,38	2,50	432,00
0,89	260,33	13,70	564,33	2,50	432,00
0,89	105,60	13,70	564,33	0,70	123,50
1,50	105,60	3,25	564,33	0,70	123,50
1,50	105,60	3,25	152,00	0,70	123,50
1,50	105,60	3,25	152,00	0,70	123,50
1,50	105,60	5,33	152,00	0,70	123,50
1,50	115,25	5,33	152,00	0,70	123,50
1,50	115,25	5,33	152,00	1,50	362,33
1,50	115,25	6,33	293,67	1,50	362,33
1,50	115,25	6,33	293,67	1,50	362,33
2,13	120,00	6,33	293,67	1,50	121,00
2,13	120,00	6,25	352,50	1,50	121,00
2,13	120,00	6,25	352,50	1,50	121,00
2,13	120,00	6,25	352,50	1,50	121,00
2,13	120,00	6,25	352,50	1,50	135,00
2,13	58,20	7,00	111,20	2,00	135,00
2,13	58,20	7,00	111,20	2,00	135,00
2,13	58,20	7,00	111,20	2,00	83,25
1,00	58,20	1,25	111,20	3,67	83,25
1,00	58,20	1,25	111,20	3,67	83,25
1,00	47,71	1,25	210,80	3,67	83,25
1,00	47,71	1,25	210,80	2,00	75,75
1,00	47,71	7,67	210,80	2,00	75,75
1,00	47,71	7,67	210,80	2,00	75,75
1,00	47,71	7,67	210,80	2,00	75,75
1,10	47,71	3,20	153,25	2,40	72,50
1,10	47,71	3,20	153,25	2,40	72,50
1,10	50,50	3,20	153,25	2,40	72,50
1,10	50,50	3,20	153,25	2,40	72,50
1,10	50,50	3,20	523,00	2,40	232,50
1,10	50,50	6,83	523,00	0,80	232,50
1,10	55,00	6,83	96,00	0,80	94,00
1,10	55,00	6,83	96,00	0,80	94,00
1,10	55,00	6,83	96,00	0,80	94,00
1,10	55,00	6,83	96,00	0,80	94,00
1,50	55,00	6,83	96,00	1,50	135,67
1,50	55,00	4,67	150,20	1,50	135,67
1,50	34,67	4,67	150,20	1,50	135,67
1,50	34,67	4,67	150,20	1,50	83,80
1,50	34,67		150,20	1,50	83,80
1,50	55,80		150,20	1,50	83,80
0,00	55,80		143,29	1,25	83,80
0,00	55,80		143,29	1,25	83,80
0,00	55,80		143,29	1,25	86,57

0,00	55,80	143,29	1,25	86,57
0,00	53,00	143,29	0,60	86,57
2,00	53,00	143,29	0,60	86,57
2,00	53,00	143,29	0,60	86,57
2,00	53,00	166,80	0,60	86,57
2,00	53,00	166,80	0,60	86,57
2,00	43,40	166,80	4,33	
0,40	43,40	166,80	4,33	
0,40	43,40	166,80	4,33	
0,40	43,40	293,75	2,75	
0,40	43,40	293,75	2,75	
0,40	91,00	293,75	2,75	
1,00	91,00	293,75	2,75	
1,00	91,00	194,00		
1,00	80,40	194,00		
1,00	80,40	194,00		
1,00	80,40	194,00		
1,00	80,40	130,80		
1,00	80,40	130,80		
1,00		130,80		
1,00		130,80		
5,33		130,80		
5,33		188,50		
5,33		188,50		
1,20		188,50		
1,20		188,50		
1,20				
1,20				
1,20				
4,67				
4,67				
4,67				
1,75				
1,75				
1,75				
1,75				
1,33				
1,33				
1,33				
1,50				
1,50				
1,50				
1,50				
4,50				
4,50				
4,50				
4,50				

1,33
1,33
1,33
1,50
1,50
0,80
0,80
0,80
0,80
0,80
0,80
1,33
1,33
1,33
4,75
4,75
4,75
4,75
1,75
1,75
1,75
1,75
2,50
2,50
2,50
2,50
3,75
3,75
3,75
3,75
3,75

1 h str. - (g)	1 h str. +CQ (h)	4 h str. - (i)	4 h str. +CQ (j)
6,17	98,88	0,80	49,33
6,17	98,88	0,80	49,33
6,17	98,88	1,50	49,33
0,57	98,88	1,50	49,33
0,57	98,88	1,50	49,33
0,57	121,33	1,50	49,33
0,57	121,33	1,50	39,57
0,57	121,33	1,50	39,57
0,57	121,33	0,83	39,57
0,57	121,33	0,83	39,57
0,67	121,33	0,83	39,57
0,67	79,29	0,83	39,57
0,67	79,29	0,83	39,57
0,67	79,29	0,83	54,83
0,67	79,29	0,25	54,83
0,67	79,29	0,25	54,83
1,25	79,29	0,25	54,83
1,25	79,29	0,25	54,83
1,25	75,67	0,43	54,83
1,25	75,67	0,43	201,00
0,25	75,67	0,43	201,00
0,25	75,67	0,43	201,00
0,25	75,67	0,43	201,00
0,25	75,67	0,43	201,00
0,25	71,00	0,43	95,50
0,25	71,00	1,25	95,50
0,25	71,00	1,25	95,50
0,25	71,00	1,25	95,50
1,67	71,00	1,25	95,50
1,67	107,50	0,00	95,50
1,67	107,50	0,00	103,38
0,00	107,50	0,00	103,38
0,00	107,50	0,00	103,38
0,00	107,50	0,00	103,38
0,00	107,50	0,00	103,38
0,00	72,57	0,00	103,38
0,40	72,57	0,00	103,38
0,40	72,57	0,00	103,38
0,40	72,57	0,00	188,60
0,40	72,57	0,00	188,60
0,40	72,57	0,40	188,60
2,14	72,57	0,40	188,60
2,14	83,33	0,40	188,60
2,14	83,33	0,40	110,43

2,14	83,33	0,40	110,43
2,14	83,33	0,00	110,43
2,14	83,33	0,00	110,43
2,14	83,33	0,00	110,43
4,00	108,33	0,00	110,43
4,00	108,33	0,00	110,43
4,00	108,33	0,00	558,50
4,00	108,33	2,00	558,50
3,75	108,33	2,00	558,50
3,75	108,33	2,00	558,50
3,75	59,67	2,00	240,00
3,75	59,67	0,70	240,00
1,00	59,67	0,70	240,00
1,00	59,67	0,70	240,00
1,00	59,67	2,00	240,00
1,00	59,67	2,00	240,00
1,00	118,33	2,00	240,00
8,00	118,33	2,00	407,67
8,00	118,33	2,00	407,67
8,00	118,33	0,80	407,67
8,00	118,33	0,80	123,50
3,25	118,33	0,80	123,50
3,25	165,60	0,80	123,50
3,25	165,60	0,80	123,50
3,25	165,60	0,80	120,00
4,75	165,60	1,00	120,00
4,75	165,60	1,00	120,00
4,75	113,14	1,00	120,00
4,75	113,14	1,00	412,00
3,60	113,14	1,00	412,00
3,60	113,14	1,00	412,00
3,60	113,14	0,30	412,00
3,60	113,14	0,30	412,00
3,60	113,14	0,30	111,60
5,00	209,40	0,30	111,60
5,00	209,40	0,30	111,60
5,00	209,40	0,30	111,60
5,00	209,40	0,30	111,60
2,25	209,40	0,30	81,00
2,25	715,00	0,30	81,00
2,25	715,00	0,30	81,00
2,25	220,80	0,30	81,00
4,25	220,80	1,00	81,00
4,25	220,80	1,00	81,00
4,25	220,80	1,00	304,67
4,25	220,80	1,00	304,67
8,50	167,50	1,00	304,67

8,50	167,50	0,70	150,00
8,50	167,50	0,70	150,00
8,50	167,50	0,70	150,00
0,33	167,50	0,70	150,00
0,33	167,50	0,70	201,50
0,33	467,00	0,70	201,50
0,00	467,00	3,20	201,50
0,00	467,00	3,20	201,50
0,00	467,00	3,20	334,75
0,00	203,00	3,20	334,75
0,00	203,00	3,20	334,75
0,00	203,00	1,28	334,75
0,00	203,00	1,28	206,67
0,00	203,00	1,28	206,36
0,00	203,00	1,29	206,05
2,67	241,80	1,29	205,74
2,67	241,80	1,30	205,43
2,67	241,80	1,30	205,12
2,00	241,80	1,31	204,81
2,00	241,80	1,31	204,50
2,00	304,71	1,31	204,19
2,00	307,97	1,32	203,88
1,50	311,23	1,32	203,56
1,50	314,50	1,33	203,25
1,50	317,76	1,33	202,94
1,50	321,03	1,34	202,63
3,50	324,29	1,34	202,32
3,50	327,56	1,34	202,01
2,50	330,82	1,35	201,70
2,50	334,09	1,35	201,39
2,67	337,35	1,36	201,08
2,67	340,62	1,36	200,77
2,67	343,88	1,37	200,46
	347,15	1,37	200,15
	350,41	1,37	199,84
	353,68	1,38	199,53
	356,94	1,38	199,22
	360,21	1,39	198,91
	363,47	1,39	198,60
	366,74	1,40	198,29
	370,00	1,40	197,98
	373,26	1,40	197,67
	376,53	1,41	197,36
	379,79	1,41	197,05
	383,06	1,42	196,74
	386,32	1,42	196,43
	389,59	1,43	196,12

392,85	1,43	195,81
396,12	1,43	195,50
399,38	1,44	195,19
402,65	1,44	194,88
405,91	1,45	194,57
409,18	1,45	194,26
412,44		193,95
415,71		
418,97		
422,24		
425,50		
428,76		
432,03		
435,29		
438,56		

Source data Figure 2D

Normalized LC3B-II/LC3B-I ratio

unstr. - (k)	10 min str. - (l)	30 min str. - (m)	1 h str. - (n)	4 h str. - (o)
1	1,339	6,185	6,346	0,784
1	1,233	1,929	0,996	2,705
1	0,979	1,463	1,680	2,114
1	4,786	5,506	2,665	3,884
1	1,439	2,374	0,919	2,903
1	6,770	7,360	7,610	3,220

Source data Figure 2D western blot membranes uncropped

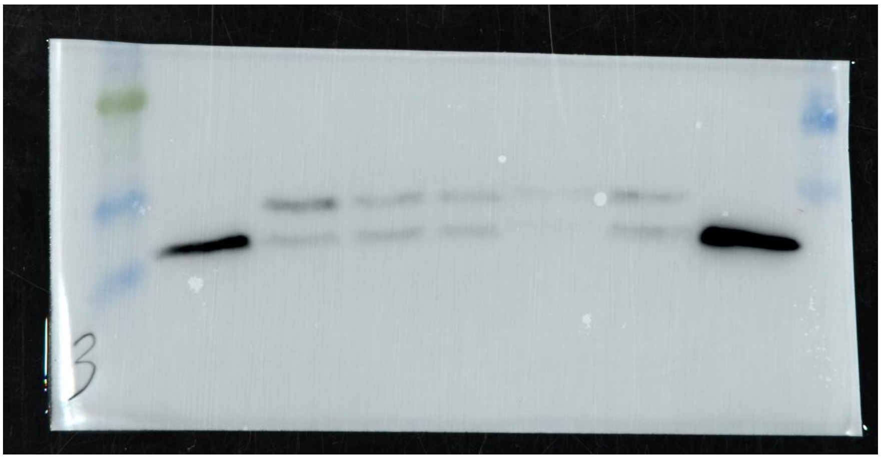

Lane 1: ladder
Lane 2: 1 h +CQ unstr.
Lane 3: unstr. – (k)
Lane 4: 10 min str. – (l)
Lane 5: 30 min str. – (m)
Lane 6: 1 h str. – (n)
Lane 7: 4 h str. – (o)
Lane 8: 4 h +CQ unstr.
Lane 9: ladder

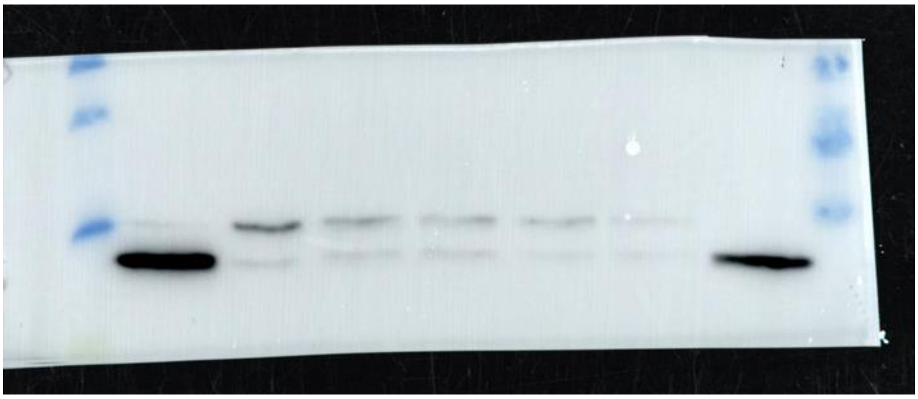

Lane 1: ladder
Lane 2: 1 h +CQ unstr.
Lane 3: unstr. – (k)
Lane 4: 10 min str. – (l)
Lane 5: 30 min str. – (m)
Lane 6: 1 h str. – (n)
Lane 7: 4 h str. – (o)
Lane 8: 4 h +CQ unstr.
Lane 9: ladder

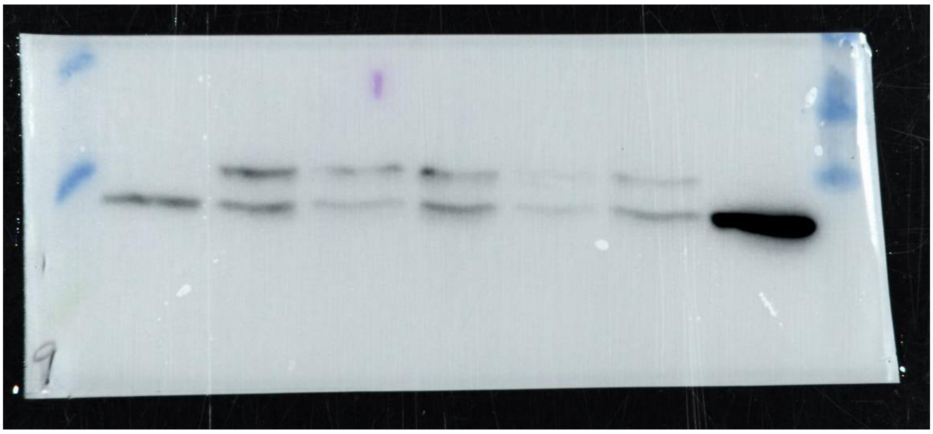

Lane 1: ladder
Lane 2: 1 h +CQ unstr.
Lane 3: unstr. – (k)
Lane 4: 10 min str. – (l)
Lane 5: 30 min str. – (m)
Lane 6: 1 h str. – (n)
Lane 7: 4 h str. – (o)
Lane 8: 4 h +CQ unstr.
Lane 9: ladder

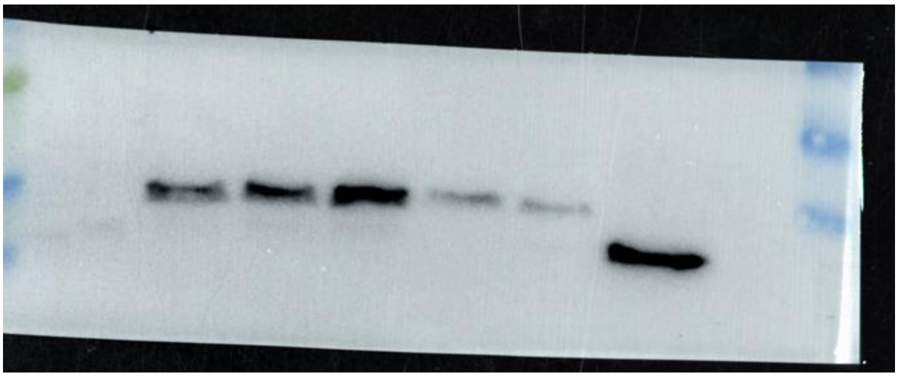

Lane 1: ladder
Lane 2: 1 h +CQ unstr.
Lane 3: unstr. – (k)
Lane 4: 10 min str. – (l)
Lane 5: 30 min str. – (m)
Lane 6: 1 h str. – (n)
Lane 7: 4 h str. – (o)
Lane 8: 4 h +CQ unstr.
Lane 9: ladder

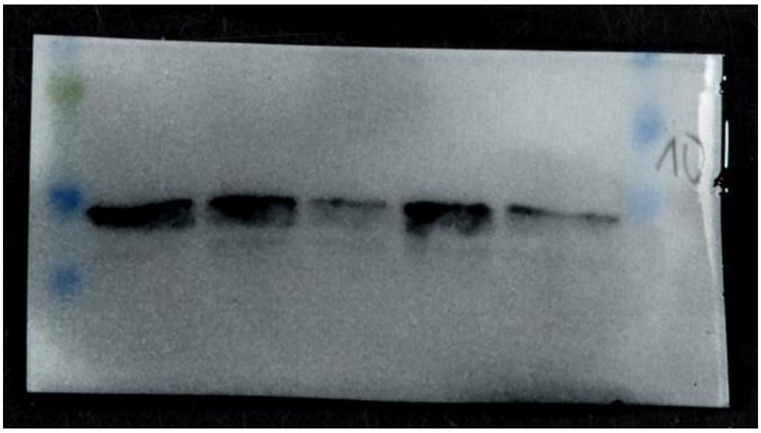

Lane 1: ladder
Lane 2: 1 h +CQ unstr.
Lane 3: unstr. – (k)
Lane 4: 10 min str. – (l)
Lane 5: 30 min str. – (m)
Lane 6: 1 h str. – (n)
Lane 7: 4 h str. – (o)
Lane 8: 4 h +CQ unstr.
Lane 9: ladder

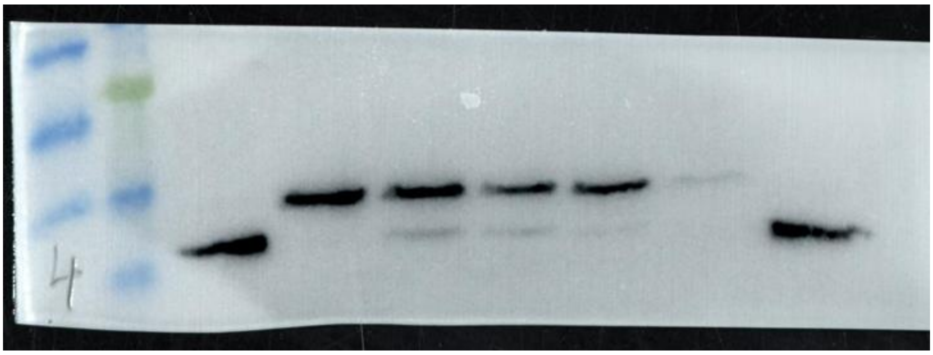

Lane 1: ladder
Lane 2: 1 h +CQ unstr.
Lane 3: unstr. – (k)
Lane 4: 10 min str. – (l)
Lane 5: 30 min str. – (m)
Lane 6: 1 h str. – (n)
Lane 7: 4 h str. – (o)
Lane 8: 4 h +CQ unstr.
Lane 9: ladder
