# Supplementary material for: Cyclic stretch induces autophagy-mediated focal adhesion remodeling and activates mitochondria
Source: Life Sci Alliance. 2026 Feb 5;9(4):e202503347. doi: 10.26508/lsa.202503347 (PMC12877405; doi:10.26508/lsa.202503347)
Supplement: Supplementary file 3 [file LSA-2025-03347_SdataF3.pdf]

**Source data Figure 3B-C**

**Main orientation of actin fibers per cell equivalent in [°]**

| unstr. (a) | unstr. +CQ (e) | 30 min str. - (b) | 30 min str. +CQ (f) | 1 h str. - (c) | 1 h str. +CQ (g) |
|---|---|---|---|---|---|
| 0,261365 | 0,00480085 | 16,9448389 | 0,69680389 | 16,8146797 | 1,29979907 |
| 0,302583 | 0,08539248 | 19,0426335 | 0,91563987 | 30,4409024 | 4,81795422 |
| 0,430367 | 0,39712558 | 22,9974346 | 1,11471182 | 31,9189895 | 6,03303036 |
| 0,440937 | 0,71807679 | 30,2315022 | 1,72603531 | 42,4727451 | 6,66748493 |
| 0,446716 | 0,73428883 | 35,3991069 | 2,34369974 | 46,7110225 | 6,83904654 |
| 0,574148 | 0,74349108 | 35,7319365 | 3,18485086 | 47,1065997 | 6,88696344 |
| 0,731345 | 0,76131412 | 37,2543288 | 3,26518005 | 47,1303674 | 7,38955106 |
| 0,774505 | 0,76895634 | 38,1706543 | 4,00804377 | 48,4059243 | 8,76172927 |
| 0,802399 | 0,90905035 | 39,0409138 | 4,828688 | 49,0499385 | 11,0344059 |
| 0,84068 | 1,06351868 | 39,3990256 | 5,60714967 | 50,7789028 | 13,9263659 |
| 0,912281 | 1,08465711 | 40,5686815 | 5,74247715 | 50,7843 | 15,8706632 |
| 0,922798 | 1,11596671 | 41,5418664 | 8,76633143 | 50,9444396 | 17,9689178 |
| 0,926267 | 1,39995548 | 42,5606021 | 9,83196085 | 51,068951 | 22,4056713 |
| 0,963679 | 1,51193112 | 42,9608917 | 10,65835 | 51,5785812 | 23,1983144 |
| 0,99967 | 1,64124872 | 44,1882133 | 11,0510037 | 51,6052715 | 25,3871399 |
| 1,057368 | 1,68301736 | 44,386695 | 11,204292 | 52,0118405 | 28,1594313 |
| 1,124023 | 1,70711215 | 45,5374243 | 11,5803319 | 53,0594227 | 35,1505801 |
| 1,136752 | 1,78260229 | 46,0109314 | 12,5030055 | 53,1256044 | 36,0675911 |
| 1,276845 | 1,78868295 | 46,2245171 | 13,3388497 | 53,2087445 | 36,3618221 |
| 1,504275 | 2,02209611 | 46,5181328 | 13,4743802 | 54,3167717 | 37,1594954 |
| 1,555863 | 2,20467696 | 46,8107032 | 15,3656224 | 54,6299516 | 39,1097294 |
| 1,624039 | 2,33582217 | 46,8692916 | 15,493821 | 54,6417011 | 40,218673 |
| 1,654068 | 2,36138136 | 46,9071435 | 16,4013887 | 54,8155431 | 40,3337683 |
| 1,670583 | 2,62011986 | 46,9535693 | 18,130017 | 55,117609 | 40,3758561 |
| 1,702624 | 2,97629494 | 46,9933519 | 18,6546054 | 55,3143414 | 40,4393697 |
| 1,719633 | 3,22739885 | 47,7458324 | 19,2165774 | 55,5188204 | 42,1312176 |
| 1,739236 | 3,40115045 | 48,0498914 | 19,4086331 | 55,6220494 | 42,1800436 |
| 1,8982 | 3,40193044 | 48,1149393 | 19,7018181 | 55,6780138 | 42,3470316 |
| 2,001278 | 3,48792121 | 48,6885709 | 21,1621941 | 56,0033471 | 42,9543251 |
| 2,010019 | 3,53791896 | 48,7844636 | 21,4908106 | 56,0054948 | 43,0971582 |
| 2,113889 | 3,8217933 | 48,9231941 | 22,6982681 | 56,1132639 | 43,5280422 |
| 2,184131 | 3,97240765 | 49,3824111 | 22,9988944 | 56,1565541 | 44,0903572 |
| 2,185538 | 4,11443369 | 49,4088711 | 23,091326 | 56,2452086 | 44,1029088 |
| 2,1908 | 4,3038328 | 49,4238569 | 23,706736 | 56,4344654 | 44,2193419 |
| 2,199055 | 4,37445434 | 49,8138678 | 25,4422482 | 56,5223878 | 44,2504577 |
| 2,300623 | 4,38386681 | 49,8725244 | 26,1843975 | 56,5382761 | 44,9764099 |
| 2,3316 | 4,43165803 | 50,2119424 | 26,9728775 | 56,6299738 | 45,0373877 |
| 2,394447 | 4,49511543 | 50,4833775 | 27,0928424 | 56,7667354 | 45,1630612 |
| 2,449926 | 4,57253306 | 50,6325897 | 27,6437264 | 57,1835732 | 45,3784555 |
| 2,578831 | 4,59815607 | 50,7127319 | 28,8673932 | 57,1944151 | 45,3827874 |
| 2,591286 | 4,64921716 | 50,7640827 | 28,9795173 | 57,5805803 | 46,3518461 |
| 2,648006 | 4,66993218 | 50,909239 | 29,8826275 | 57,8401582 | 46,5288612 |
| 2,693417 | 4,67754966 | 50,9595489 | 29,92383 | 57,8411286 | 46,5705156 |
| 2,746976 | 4,86644844 | 51,1898451 | 29,948636 | 58,0199618 | 46,7437521 |

| | | | | | |
|---|---|---|---|---|---|
| 2,759586 | 4,87832541 | 51,2981719 | 30,0921758 | 58,1557702 | 46,8175522 |
| 2,780544 | 4,89955055 | 51,3125051 | 30,2723581 | 58,2470934 | 46,8281005 |
| 2,984567 | 4,9869854 | 51,5866917 | 30,3957089 | 58,2620326 | 46,9013459 |
| 2,999808 | 5,23198569 | 51,6126589 | 33,7603132 | 58,3027038 | 46,9660082 |
| 3,043332 | 5,23380875 | 51,7774977 | 33,7633857 | 58,5589532 | 47,1816058 |
| 3,078523 | 5,4937505 | 51,8122218 | 33,8671876 | 58,6440925 | 47,573834 |
| 3,124056 | 5,59535385 | 51,8589166 | 33,9627315 | 58,7474872 | 47,6186324 |
| 3,148891 | 5,6918427 | 51,9019544 | 34,0091798 | 58,8214927 | 47,7113237 |
| 3,289615 | 5,72418349 | 51,9449616 | 34,019517 | 58,8815738 | 47,8626678 |
| 3,319964 | 5,7762388 | 52,0311211 | 34,502119 | 58,8862448 | 48,0118837 |
| 3,348681 | 5,80135508 | 52,1035197 | 34,673367 | 58,897092 | 48,0570218 |
| 3,409901 | 6,27767059 | 52,1271368 | 35,7296517 | 58,900376 | 48,0577962 |
| 3,414966 | 6,65940971 | 52,2312061 | 35,7616569 | 58,9389897 | 48,2354962 |
| 3,773194 | 6,72051072 | 52,3681085 | 35,9614303 | 59,1106569 | 48,2549197 |
| 3,900971 | 6,76196532 | 52,5903866 | 36,1801184 | 59,2632533 | 48,2666094 |
| 3,951219 | 6,99143504 | 52,756799 | 36,9230632 | 59,3174622 | 48,4099335 |
| 4,065403 | 7,30570055 | 52,9658676 | 37,1701561 | 59,3786497 | 48,4215564 |
| 4,084206 | 7,3278898 | 52,9727909 | 37,2106776 | 59,4778932 | 48,4977442 |
| 4,265473 | 7,47117312 | 53,2016712 | 38,3037495 | 59,5107473 | 48,6820005 |
| 4,306975 | 7,62516987 | 53,2360383 | 38,3177726 | 59,5501899 | 48,7251981 |
| 4,43177 | 7,70145269 | 53,3263575 | 38,4107763 | 59,618962 | 49,0130222 |
| 4,7036 | 7,72677175 | 53,3495802 | 38,8407781 | 59,6319645 | 49,1895888 |
| 4,716634 | 7,83558432 | 53,3603198 | 38,9787033 | 59,6952416 | 49,1983777 |
| 4,806232 | 7,83781419 | 53,3889569 | 39,2676961 | 59,7207834 | 49,4074098 |
| 4,926335 | 8,56320186 | 53,4549478 | 39,6800839 | 59,757545 | 49,450973 |
| 5,132254 | 8,60182442 | 53,4706153 | 40,0977575 | 59,7872273 | 49,5197214 |
| 5,1741 | 8,63894695 | 53,576313 | 40,9774942 | 59,8233263 | 49,7392027 |
| 5,189698 | 8,91019309 | 53,5968571 | 41,2067171 | 60,1962577 | 49,7761039 |
| 5,20281 | 9,00581887 | 53,6079439 | 41,2080105 | 60,3380413 | 49,8871789 |
| 5,212137 | 9,72471102 | 53,6183351 | 41,2363517 | 60,5326521 | 49,9621036 |
| 5,230379 | 9,90837336 | 53,6187951 | 41,255651 | 60,5562466 | 50,0016077 |
| 5,30902 | 9,94967533 | 53,7203208 | 41,3462113 | 60,6229069 | 50,043159 |
| 5,34571 | 9,98393597 | 53,7514872 | 41,5690957 | 60,7024351 | 50,0582931 |
| 5,356315 | 10,1220593 | 53,7530663 | 41,9281576 | 60,7711979 | 50,2234541 |
| 5,363199 | 10,1924546 | 53,8133589 | 42,0006299 | 60,8385943 | 50,3061094 |
| 5,392439 | 10,3058256 | 53,8271673 | 42,102889 | 61,1853349 | 50,3604622 |
| 5,442175 | 10,5209021 | 54,0245901 | 42,2433002 | 61,2036004 | 50,578713 |
| 5,497432 | 10,7339958 | 54,0404987 | 42,4030932 | 61,2187277 | 50,6849548 |
| 5,807544 | 10,9492149 | 54,1239554 | 42,4127868 | 61,3005926 | 50,8759022 |
| 5,909158 | 10,9931165 | 54,1334805 | 42,9036424 | 61,3319981 | 50,9997301 |
| 5,951578 | 11,2684773 | 54,320962 | 42,9190125 | 61,4932755 | 51,0214918 |
| 6,106138 | 11,28394 | 54,4886587 | 42,9379627 | 61,542384 | 51,065358 |
| 6,171237 | 11,4194929 | 54,4951992 | 42,9616557 | 61,5644059 | 51,0957022 |
| 6,250642 | 11,5566866 | 54,5443496 | 42,9979606 | 61,585271 | 51,1047524 |
| 6,273539 | 11,5690523 | 54,5562181 | 43,0204049 | 61,5994813 | 51,3471715 |
| 6,304464 | 11,6625177 | 54,6426252 | 43,1281565 | 61,6098842 | 51,5903042 |
| 6,363966 | 11,7328353 | 55,0054682 | 43,140515 | 61,6200889 | 51,6594454 |

| | | | | | |
|---|---|---|---|---|---|
| 6,455263 | 11,916155 | 55,1586365 | 43,1566309 | 61,6382043 | 51,735088 |
| 6,484776 | 12,5106745 | 55,2510296 | 43,1953217 | 61,7122764 | 51,9339229 |
| 6,505032 | 12,7915907 | 55,2513044 | 43,3934795 | 61,9448055 | 52,028813 |
| 6,640426 | 13,1132459 | 55,2812584 | 43,6106986 | 62,1368384 | 52,4119508 |
| 6,698541 | 13,145239 | 55,3059512 | 43,634614 | 62,2384878 | 52,415219 |
| 6,829642 | 13,4168451 | 55,437117 | 43,750631 | 62,2602823 | 52,4589201 |
| 6,859519 | 13,5814059 | 55,7767876 | 43,7515456 | 62,3641731 | 52,4837746 |
| 6,901633 | 13,6651635 | 55,9250058 | 43,8585558 | 62,3748826 | 52,4963358 |
| 6,904472 | 13,951838 | 55,9497132 | 43,8763633 | 62,4028941 | 52,4982845 |
| 7,01215 | 14,4452744 | 56,0785994 | 43,8771288 | 62,5475014 | 52,519079 |
| 7,123521 | 14,5497407 | 56,0789817 | 44,1750273 | 62,5737458 | 52,5542486 |
| 7,184979 | 15,2547842 | 56,2429529 | 44,2041467 | 62,76781 | 52,5635652 |
| 7,340101 | 15,2783828 | 56,3258275 | 44,2830168 | 62,7684205 | 52,6379363 |
| 7,56451 | 15,3938251 | 56,4333798 | 44,2837364 | 62,7784765 | 52,678679 |
| 8,057189 | 15,5207213 | 56,4436265 | 44,3277356 | 62,8218145 | 52,7040157 |
| 8,080428 | 15,8206853 | 56,449491 | 44,4487898 | 62,8721961 | 52,7153318 |
| 8,147047 | 15,9775132 | 56,4552616 | 44,6576285 | 62,8784658 | 52,8044097 |
| 8,205856 | 16,5339992 | 56,4573997 | 44,6822572 | 62,8975363 | 52,805979 |
| 8,227278 | 16,5506952 | 56,6444728 | 44,7324241 | 62,9488688 | 52,9003649 |
| 8,251403 | 16,8754618 | 56,6529571 | 44,7720612 | 62,9694185 | 53,0134595 |
| 8,526913 | 17,4743259 | 56,6623088 | 45,0115197 | 63,0628339 | 53,1254091 |
| 8,540852 | 17,5049467 | 56,7147751 | 45,0523408 | 63,086025 | 53,2551831 |
| 8,586715 | 17,5129189 | 56,8221274 | 45,1325894 | 63,096461 | 53,3038985 |
| 8,678264 | 17,7038884 | 56,8773999 | 45,1414072 | 63,1650921 | 53,3778623 |
| 8,833044 | 17,7156174 | 56,978193 | 45,243213 | 63,1861234 | 53,3996338 |
| 8,956874 | 17,7830048 | 56,984799 | 45,3823342 | 63,2407061 | 53,6679265 |
| 9,173917 | 17,9541999 | 57,0767263 | 45,6982268 | 63,286124 | 53,7505662 |
| 9,200912 | 18,0899847 | 57,113067 | 45,7193038 | 63,3201932 | 53,8516722 |
| 9,300853 | 18,3172921 | 57,1263744 | 45,7588438 | 63,4741855 | 53,9350326 |
| 9,41911 | 18,4931219 | 57,1279957 | 45,7743804 | 63,4913485 | 53,9982283 |
| 9,599308 | 18,9760963 | 57,1308115 | 45,8002604 | 63,5445344 | 54,0226965 |
| 9,708557 | 19,5208977 | 57,147648 | 45,8150423 | 63,5823929 | 54,0593287 |
| 9,791954 | 19,7635352 | 57,2311172 | 45,8354102 | 63,6035292 | 54,1426231 |
| 9,818494 | 19,9677348 | 57,2311317 | 46,004576 | 63,6382295 | 54,274393 |
| 9,874664 | 20,076163 | 57,2742437 | 46,0906413 | 63,6733547 | 54,3445271 |
| 10,1268 | 20,0958442 | 57,3132522 | 46,2854178 | 63,6918557 | 54,4306863 |
| 10,13187 | 20,2939153 | 57,3227065 | 46,3530292 | 63,6928439 | 54,5069861 |
| 10,18159 | 20,3318479 | 57,3283439 | 46,3635603 | 63,7263861 | 54,5758117 |
| 10,3245 | 20,3904753 | 57,4145544 | 46,5745724 | 63,7765984 | 54,5804744 |
| 10,3511 | 20,4218364 | 57,4148375 | 46,6842716 | 63,7883294 | 54,6541089 |
| 10,35255 | 20,6078339 | 57,4173743 | 46,9475828 | 63,8086557 | 54,6600974 |
| 10,64638 | 20,9078922 | 57,5759202 | 47,0705921 | 63,8329113 | 54,7084064 |
| 10,73907 | 21,0965246 | 57,5876436 | 47,2092934 | 63,8576865 | 54,7307221 |
| 10,74037 | 21,1110614 | 57,5893269 | 47,392509 | 63,8661406 | 54,7971779 |
| 10,77122 | 21,2575414 | 57,6250576 | 47,5871803 | 63,866348 | 54,8202691 |
| 10,82621 | 21,496992 | 57,7694439 | 47,6537405 | 63,9128283 | 54,8323014 |
| 10,91249 | 21,8951431 | 57,7936253 | 47,6650038 | 64,0275254 | 54,8497929 |

| | | | | | |
|---|---|---|---|---|---|
| 11,03876 | 21,9070419 | 57,8002483 | 47,7094441 | 64,0616928 | 54,8823772 |
| 11,15357 | 21,9350825 | 57,9703256 | 47,7316202 | 64,1157385 | 54,8909469 |
| 11,17331 | 22,0646013 | 57,9826948 | 47,7824017 | 64,1609588 | 54,9791679 |
| 11,23684 | 22,2503225 | 57,9959297 | 47,8848536 | 64,2137863 | 55,0902698 |
| 11,26326 | 22,2693308 | 58,0525095 | 47,926015 | 64,2865191 | 55,2479193 |
| 11,52569 | 22,3321776 | 58,0727231 | 47,9875331 | 64,2901181 | 55,2734705 |
| 11,60477 | 22,4462493 | 58,0856529 | 48,0578869 | 64,3533785 | 55,3106744 |
| 11,82821 | 22,6931732 | 58,1473918 | 48,1280036 | 64,3639052 | 55,3711918 |
| 12,06484 | 22,7012938 | 58,1564539 | 48,297237 | 64,3845913 | 55,4299508 |
| 12,14932 | 23,0266453 | 58,2186605 | 48,3406647 | 64,4121502 | 55,4554157 |
| 12,26037 | 23,1031058 | 58,2198756 | 48,4971507 | 64,4638299 | 55,4827808 |
| 12,26202 | 23,1718162 | 58,2329426 | 48,6121747 | 64,478169 | 55,569106 |
| 12,38291 | 23,5144345 | 58,2627461 | 48,638109 | 64,5191163 | 55,572664 |
| 12,42463 | 23,5354601 | 58,2990923 | 48,6652485 | 64,5464457 | 55,762111 |
| 12,54075 | 24,1377304 | 58,3664915 | 48,8276272 | 64,5850118 | 55,7799361 |
| 12,7147 | 24,3740792 | 58,3756826 | 49,0157531 | 64,613204 | 55,9273632 |
| 12,91624 | 24,4332718 | 58,3996272 | 49,2229101 | 64,6331493 | 56,0823355 |
| 12,95484 | 24,7818503 | 58,431002 | 49,2460118 | 64,6351833 | 56,0898799 |
| 13,04349 | 24,9012714 | 58,445095 | 49,3011841 | 64,6391601 | 56,144079 |
| 13,13274 | 25,0526967 | 58,5430807 | 49,3123528 | 64,6670037 | 56,1866327 |
| 13,16995 | 25,2974987 | 58,5542801 | 49,3248817 | 64,7413169 | 56,2504633 |
| 13,33293 | 25,3453486 | 58,5954286 | 49,4312841 | 64,7473039 | 56,2699465 |
| 13,91588 | 25,6232007 | 58,6485041 | 49,4592681 | 64,8298357 | 56,3343779 |
| 14,09033 | 25,6571943 | 58,6711919 | 49,4783222 | 64,8396978 | 56,3637921 |
| 14,39812 | 25,8753444 | 58,6785767 | 49,494907 | 64,8812962 | 56,4729955 |
| 14,51459 | 26,1548051 | 58,6862832 | 49,7097383 | 64,9417229 | 56,5018271 |
| 14,95478 | 26,1888171 | 58,6932586 | 49,7143948 | 64,9419703 | 56,5529884 |
| 14,99262 | 26,2582925 | 58,8334871 | 49,7281042 | 64,9442353 | 56,6350841 |
| 15,23448 | 26,3703087 | 58,8688103 | 49,7963763 | 64,9554471 | 56,6692917 |
| 15,27415 | 26,4968615 | 58,9011059 | 49,8225565 | 64,9662136 | 56,681375 |
| 15,51908 | 26,6341473 | 58,9233697 | 49,9976215 | 64,9939608 | 56,6821601 |
| 15,58445 | 26,8347772 | 59,038115 | 50,0374142 | 65,0105442 | 56,7907352 |
| 15,65323 | 26,875027 | 59,0415251 | 50,0689609 | 65,0159245 | 56,8238949 |
| 16,02456 | 26,8809782 | 59,0699942 | 50,0987482 | 65,0191167 | 56,8815996 |
| 16,06666 | 26,9739851 | 59,1002141 | 50,147171 | 65,0799932 | 57,0379299 |
| 16,19451 | 26,9840113 | 59,1424991 | 50,1662899 | 65,1065005 | 57,0723826 |
| 16,55134 | 27,1584791 | 59,1737006 | 50,2483223 | 65,122442 | 57,1635378 |
| 16,65057 | 27,3592321 | 59,2010933 | 50,2497663 | 65,1393174 | 57,4692193 |
| 16,67717 | 27,3986157 | 59,2040024 | 50,6090968 | 65,1948008 | 57,4739881 |
| 16,70673 | 27,4431082 | 59,2167965 | 50,7214551 | 65,3403471 | 57,4767082 |
| 16,7792 | 27,4996541 | 59,2282692 | 50,7898678 | 65,3495452 | 57,5766313 |
| 16,91657 | 27,5987267 | 59,2408315 | 50,9105485 | 65,4518206 | 57,5808759 |
| 17,28168 | 27,747307 | 59,2463931 | 51,0245611 | 65,4738053 | 57,664978 |
| 17,39268 | 27,7707742 | 59,292122 | 51,070348 | 65,558924 | 57,7826304 |
| 17,43421 | 27,8366433 | 59,503524 | 51,1427652 | 65,5777591 | 57,8011653 |
| 17,44417 | 27,9209086 | 59,5372322 | 51,5286589 | 65,6411596 | 57,8367276 |
| 17,47556 | 28,0185756 | 59,5509154 | 51,6681784 | 65,6565748 | 57,8585599 |

| | | | | | |
|---|---|---|---|---|---|
| 17,69378 | 28,0696238 | 59,584883 | 51,7358694 | 65,7144594 | 58,1435651 |
| 17,92656 | 28,0780954 | 59,6371168 | 51,8072243 | 65,7297704 | 58,2142967 |
| 18,01576 | 28,0923133 | 59,6452664 | 51,8099519 | 65,7581829 | 58,2305265 |
| 18,53279 | 28,3896795 | 59,6749859 | 51,821047 | 65,9338395 | 58,24245 |
| 18,65538 | 28,4909801 | 59,6982431 | 51,8304674 | 65,9626784 | 58,292575 |
| 18,68291 | 28,6028979 | 59,701934 | 51,8820789 | 65,9754629 | 58,4969601 |
| 18,78773 | 28,7174444 | 59,7189691 | 52,0174086 | 65,9992174 | 58,5129055 |
| 19,0194 | 28,7643651 | 59,7526043 | 52,0597049 | 66,0027782 | 58,5333664 |
| 19,1043 | 28,8181448 | 59,8305254 | 52,1280181 | 66,0302533 | 58,5568617 |
| 19,19355 | 28,8506429 | 59,8745436 | 52,210768 | 66,0437403 | 58,6158655 |
| 19,21719 | 29,3565137 | 59,8900313 | 52,2515806 | 66,1009826 | 58,6512782 |
| 19,21904 | 29,570321 | 59,9175526 | 52,3390787 | 66,1246775 | 58,6943674 |
| 19,37456 | 29,7122838 | 59,9342418 | 52,4374382 | 66,1429916 | 58,8111293 |
| 19,43039 | 29,7229416 | 59,9367961 | 52,5405696 | 66,1430525 | 58,93943 |
| 19,51126 | 29,9720518 | 59,95536 | 52,6621648 | 66,2055895 | 58,9540322 |
| 19,58326 | 30,1202169 | 60,1202728 | 52,6713389 | 66,2819368 | 59,0505345 |
| 19,59052 | 30,7056713 | 60,1660641 | 52,7045031 | 66,3026702 | 59,0844509 |
| 19,70595 | 30,8044697 | 60,1783292 | 52,7120753 | 66,3316476 | 59,1515183 |
| 19,87596 | 30,8106013 | 60,1937347 | 52,8541605 | 66,3943559 | 59,1762322 |
| 20,18513 | 30,9623041 | 60,2457472 | 52,8608413 | 66,4459057 | 59,1852042 |
| 20,38759 | 31,0102735 | 60,2987353 | 52,8690142 | 66,4550015 | 59,2541125 |
| 20,49915 | 31,128488 | 60,3251811 | 53,067264 | 66,4667218 | 59,3777187 |
| 20,57059 | 31,1889063 | 60,3585542 | 53,0814599 | 66,6071952 | 59,4103374 |
| 20,72522 | 31,2743563 | 60,4557576 | 53,095744 | 66,6131854 | 59,4369115 |
| 20,72795 | 31,2958574 | 60,5262752 | 53,1596391 | 66,6272034 | 59,4756684 |
| 20,7898 | 31,3140459 | 60,5475991 | 53,2170846 | 66,6518034 | 59,4859475 |
| 21,00808 | 31,4678365 | 60,5653631 | 53,2561312 | 66,7483634 | 59,4894254 |
| 21,01178 | 31,5016039 | 60,7054474 | 53,2576847 | 66,7566856 | 59,5752038 |
| 21,08471 | 31,5451022 | 60,7238153 | 53,2683536 | 66,8033724 | 59,7410038 |
| 21,21353 | 31,9936557 | 60,7282984 | 53,3230724 | 66,8448123 | 59,7727815 |
| 21,28299 | 32,0496607 | 60,7358976 | 53,3412854 | 66,9294066 | 59,7930956 |
| 21,29499 | 32,0939082 | 60,7684276 | 53,4924758 | 66,9572541 | 59,800512 |
| 21,38637 | 32,1842537 | 60,7837971 | 53,5028136 | 66,9761859 | 59,8682293 |
| 21,4832 | 32,3125634 | 60,8117593 | 53,582812 | 67,0048319 | 59,8803071 |
| 21,63095 | 32,3244185 | 60,8397751 | 53,6469682 | 67,0106598 | 59,9315771 |
| 21,63839 | 32,8763334 | 60,8504565 | 53,7060867 | 67,0150302 | 59,978738 |
| 21,70806 | 32,9018234 | 60,9007204 | 53,7161574 | 67,0527771 | 60,0197697 |
| 21,71355 | 32,9234188 | 60,9221996 | 53,7747508 | 67,0698515 | 60,0340474 |
| 21,79175 | 33,1389361 | 60,9708018 | 53,8224954 | 67,0745126 | 60,0501141 |
| 21,81342 | 33,3297493 | 60,9732966 | 53,9426156 | 67,1635123 | 60,051713 |
| 21,88808 | 33,4776409 | 60,9796113 | 53,9676265 | 67,1725884 | 60,0746703 |
| 21,90659 | 33,6225865 | 61,0278278 | 54,0214446 | 67,256817 | 60,0808572 |
| 21,94515 | 34,0343085 | 61,060656 | 54,0786676 | 67,261214 | 60,1025308 |
| 22,05144 | 34,1274933 | 61,075442 | 54,087407 | 67,2794897 | 60,1366039 |
| 22,13903 | 34,2230247 | 61,0930683 | 54,162983 | 67,365227 | 60,1599768 |
| 22,18348 | 34,2442134 | 61,10688 | 54,2163349 | 67,4261967 | 60,2783716 |
| 22,29469 | 34,2721894 | 61,1237623 | 54,2193635 | 67,4341176 | 60,3477821 |

| | | | | | |
|---|---|---|---|---|---|
| 22,3859 | 34,3823249 | 61,1828691 | 54,2960515 | 67,4449349 | 60,3731455 |
| 22,39774 | 34,4413141 | 61,1925173 | 54,3005074 | 67,4550185 | 60,3851546 |
| 22,59034 | 34,5050585 | 61,2045038 | 54,3314142 | 67,4578196 | 60,4103208 |
| 22,69632 | 34,6686509 | 61,2200205 | 54,4093321 | 67,459243 | 60,4106021 |
| 22,8393 | 35,0668051 | 61,2758984 | 54,4501184 | 67,4718248 | 60,4705841 |
| 22,91731 | 35,0803216 | 61,3055583 | 54,5737538 | 67,4871408 | 60,5721319 |
| 22,92482 | 35,080564 | 61,3352217 | 54,6215775 | 67,4877416 | 60,6301394 |
| 22,97852 | 35,1057144 | 61,4670644 | 54,7329842 | 67,5223849 | 60,6384413 |
| 23,06814 | 35,1399151 | 61,4861758 | 54,7405206 | 67,5605508 | 60,6698533 |
| 23,06821 | 35,348814 | 61,5242622 | 54,8120577 | 67,5690361 | 60,7297683 |
| 23,13705 | 35,5644493 | 61,541912 | 54,8401602 | 67,5930621 | 60,7848489 |
| 23,18431 | 35,5818252 | 61,6154137 | 54,8447102 | 67,5994253 | 61,0235226 |
| 23,20505 | 35,8340586 | 61,6404097 | 54,9225978 | 67,6435193 | 61,039853 |
| 23,41869 | 35,8761103 | 61,6676869 | 54,9330459 | 67,659196 | 61,0837994 |
| 23,47702 | 35,9118741 | 61,7156461 | 55,0360712 | 67,8012747 | 61,1206848 |
| 23,66626 | 35,9286469 | 61,7404567 | 55,1218347 | 67,8521797 | 61,1243437 |
| 23,88658 | 36,1112199 | 61,7452632 | 55,1508125 | 67,9361328 | 61,1408853 |
| 23,93278 | 36,4075505 | 61,7644985 | 55,3104539 | 67,9582531 | 61,1546068 |
| 23,94526 | 36,4475955 | 61,8021013 | 55,5539946 | 67,9808001 | 61,198055 |
| 24,05395 | 36,4651817 | 61,8134398 | 55,5600797 | 68,0795293 | 61,2026312 |
| 24,1018 | 36,5595354 | 61,8313645 | 55,5765477 | 68,1690188 | 61,3005653 |
| 24,11911 | 36,8094931 | 61,8452047 | 55,6348939 | 68,1860352 | 61,3518202 |
| 24,13116 | 37,1192501 | 61,8455908 | 55,6720049 | 68,2086191 | 61,3846886 |
| 24,13574 | 37,1755168 | 61,8652666 | 55,7090516 | 68,2278025 | 61,4061599 |
| 24,16318 | 37,2125937 | 61,8906675 | 55,7135871 | 68,243365 | 61,480863 |
| 24,1639 | 37,2180009 | 61,9052481 | 55,7664109 | 68,2543717 | 61,6514956 |
| 24,20561 | 37,241608 | 61,9576233 | 55,8102125 | 68,3489923 | 61,7182226 |
| 24,23077 | 37,3672854 | 62,0009616 | 55,8704765 | 68,356198 | 61,7889801 |
| 24,23942 | 37,7395245 | 62,0317532 | 55,9058975 | 68,3747641 | 61,7982011 |
| 24,32099 | 37,7417404 | 62,0483539 | 55,966572 | 68,3794831 | 61,8112129 |
| 24,41492 | 37,8586449 | 62,0953772 | 55,9856801 | 68,5334467 | 61,8270248 |
| 24,43717 | 37,8994612 | 62,1020227 | 56,2226629 | 68,6326516 | 61,8819051 |
| 24,45869 | 38,2518304 | 62,1091288 | 56,2284102 | 68,6803468 | 62,0212269 |
| 24,46485 | 38,5489863 | 62,1108779 | 56,2416833 | 68,7446063 | 62,0557309 |
| 24,54528 | 38,6222305 | 62,1228832 | 56,2941251 | 68,7846096 | 62,1411229 |
| 24,62752 | 38,6882201 | 62,1320262 | 56,3300161 | 68,7956714 | 62,2119741 |
| 24,76281 | 38,7346967 | 62,1476319 | 56,3714979 | 68,8210407 | 62,2365909 |
| 24,78614 | 38,8427473 | 62,159982 | 56,4704069 | 68,8296127 | 62,242433 |
| 25,2043 | 39,2088728 | 62,1662113 | 56,4814962 | 68,8555822 | 62,2946035 |
| 25,25176 | 39,2751013 | 62,1994337 | 56,5448155 | 68,8865389 | 62,3159201 |
| 25,27916 | 39,3929065 | 62,2377838 | 56,5919086 | 68,891791 | 62,3358202 |
| 25,3734 | 39,4800408 | 62,2437085 | 56,6712452 | 68,9028697 | 62,4024379 |
| 25,91747 | 39,7537468 | 62,2601938 | 56,6769412 | 68,9302165 | 62,4292771 |
| 25,95874 | 39,9249601 | 62,299762 | 56,7600313 | 68,9597194 | 62,5377906 |
| 26,06359 | 39,9757705 | 62,3160059 | 56,8185032 | 69,0174701 | 62,5696422 |
| 26,07387 | 40,0360567 | 62,3354917 | 56,8258685 | 69,0235106 | 62,6140641 |
| 26,10252 | 40,0538592 | 62,4732743 | 56,8748647 | 69,0491189 | 62,8127887 |

| | | | | | |
|---|---|---|---|---|---|
| 26,60089 | 40,1153239 | 62,5137485 | 56,8955862 | 69,0842638 | 62,8497931 |
| 27,11933 | 40,1396837 | 62,5384552 | 57,0547361 | 69,0938402 | 62,9881399 |
| 27,12093 | 40,3718822 | 62,5683459 | 57,0586633 | 69,1163879 | 63,0496755 |
| 27,16672 | 40,4444431 | 62,5762071 | 57,0720506 | 69,1222451 | 63,1866231 |
| 27,33476 | 40,6121436 | 62,5923322 | 57,1244952 | 69,1277456 | 63,2963376 |
| 27,34932 | 40,6402919 | 62,6629732 | 57,1890845 | 69,1328337 | 63,3383692 |
| 27,58147 | 40,6519602 | 62,6848132 | 57,2287422 | 69,1493853 | 63,3480912 |
| 27,65704 | 40,7022722 | 62,6891108 | 57,3761481 | 69,1531315 | 63,3772306 |
| 27,6598 | 40,9563501 | 62,7092379 | 57,3805685 | 69,1608807 | 63,4003503 |
| 27,67818 | 40,9848927 | 62,7402706 | 57,41738 | 69,1884171 | 63,4027633 |
| 27,75279 | 41,0679297 | 62,7696096 | 57,4194107 | 69,2008218 | 63,4262451 |
| 27,76556 | 41,179875 | 62,807236 | 57,4368962 | 69,214206 | 63,4266763 |
| 27,81475 | 41,3077409 | 62,812239 | 57,5090556 | 69,2806813 | 63,4270576 |
| 27,89996 | 41,3216816 | 62,8411613 | 57,5618184 | 69,3375591 | 63,462675 |
| 27,9279 | 41,347454 | 62,8882162 | 57,565578 | 69,3431534 | 63,4843042 |
| 27,93442 | 41,4878587 | 62,9278261 | 57,5984964 | 69,362066 | 63,4850243 |
| 27,99251 | 41,5726838 | 62,9298403 | 57,6235566 | 69,3992406 | 63,5039819 |
| 28,19072 | 41,6456128 | 63,0127175 | 57,7736396 | 69,4032623 | 63,5492277 |
| 28,29697 | 41,7980717 | 63,0148703 | 57,7881319 | 69,4439784 | 63,554234 |
| 28,30954 | 41,8731945 | 63,0403148 | 57,8024999 | 69,5639923 | 63,5626621 |
| 28,80354 | 41,9332654 | 63,0588251 | 57,823259 | 69,5787625 | 63,5639721 |
| 28,83856 | 41,9964562 | 63,1446181 | 57,8262963 | 69,5890438 | 63,6110805 |
| 29,10346 | 42,0910423 | 63,2892257 | 57,9091298 | 69,6600762 | 63,6297551 |
| 29,17046 | 42,1043715 | 63,3573614 | 57,9609114 | 69,6942224 | 63,7201684 |
| 29,17779 | 42,1891262 | 63,3734709 | 57,9626545 | 69,7072471 | 63,7247748 |
| 29,24546 | 42,2126155 | 63,3766269 | 57,9735472 | 69,7092224 | 63,7892239 |
| 29,32893 | 42,2974864 | 63,3871431 | 57,9838212 | 69,7125597 | 63,8474181 |
| 29,36507 | 42,3216206 | 63,406349 | 57,9974387 | 69,7284411 | 63,871569 |
| 29,52189 | 42,3970488 | 63,451351 | 58,0234702 | 69,743526 | 63,8765794 |
| 29,86889 | 42,4917929 | 63,4550397 | 58,0505618 | 69,7506225 | 63,9361839 |
| 29,89115 | 42,5207906 | 63,492928 | 58,0613033 | 69,7507662 | 63,9458627 |
| 29,93752 | 42,6290586 | 63,5592333 | 58,0776225 | 69,7809209 | 63,9576598 |
| 30,10011 | 42,7007795 | 63,5614473 | 58,1422112 | 69,8164608 | 63,972392 |
| 30,20131 | 42,7070463 | 63,5874433 | 58,1584163 | 69,8202745 | 64,0378046 |
| 30,58617 | 42,7263326 | 63,5890318 | 58,1865513 | 69,8377225 | 64,0551934 |
| 30,60407 | 42,7545623 | 63,5919638 | 58,2514874 | 69,9048279 | 64,1059907 |
| 30,64009 | 42,8013662 | 63,6513035 | 58,2664635 | 69,9225383 | 64,1171397 |
| 30,6599 | 42,8494114 | 63,6918933 | 58,2846048 | 69,9247557 | 64,1228716 |
| 30,93221 | 42,9396388 | 63,7215979 | 58,2896851 | 69,9377062 | 64,1396412 |
| 31,26429 | 43,059838 | 63,7245573 | 58,3084782 | 69,9710338 | 64,1464781 |
| 31,2849 | 43,0976427 | 63,736436 | 58,3578936 | 69,982965 | 64,1538839 |
| 31,28595 | 43,1779729 | 63,7700636 | 58,3987056 | 69,9975144 | 64,193173 |
| 31,32586 | 43,2106655 | 63,7874103 | 58,4312631 | 70,0007973 | 64,214266 |
| 31,34387 | 43,30565 | 63,8002982 | 58,5127708 | 70,0224058 | 64,2362525 |
| 31,449 | 43,3158414 | 63,8625919 | 58,516927 | 70,1299807 | 64,3084875 |
| 31,48564 | 43,3778097 | 63,8645014 | 58,5324783 | 70,1452276 | 64,3177661 |
| 31,51341 | 43,4127256 | 63,9542884 | 58,5975322 | 70,1471358 | 64,3493049 |

| | | | | | |
|---|---|---|---|---|---|
| 31,61371 | 43,5847284 | 63,9667692 | 58,6020863 | 70,1502828 | 64,3493528 |
| 31,73121 | 43,6317428 | 63,9794377 | 58,6188277 | 70,1514793 | 64,4076697 |
| 31,73286 | 43,6863016 | 64,0144577 | 58,6400948 | 70,2090412 | 64,4301182 |
| 31,7911 | 43,721003 | 64,0235905 | 58,7707668 | 70,2117587 | 64,6141461 |
| 31,81212 | 43,7246424 | 64,0285473 | 58,8106536 | 70,2625729 | 64,6304503 |
| 31,89782 | 43,8488124 | 64,0344379 | 58,8119974 | 70,2691956 | 64,6375648 |
| 31,93374 | 43,9810014 | 64,0476941 | 58,8398424 | 70,2713422 | 64,6784524 |
| 31,95762 | 43,9958634 | 64,0572957 | 58,9140109 | 70,2730196 | 64,6851413 |
| 32,02244 | 44,0269927 | 64,0823457 | 58,9254112 | 70,3107382 | 64,6953427 |
| 32,10422 | 44,1892257 | 64,1611402 | 58,9353584 | 70,3401285 | 64,7123174 |
| 32,46901 | 44,4308143 | 64,1891019 | 58,9675035 | 70,3457814 | 64,7190288 |
| 32,57509 | 44,4465807 | 64,1989365 | 58,9967604 | 70,3522782 | 64,8142009 |
| 32,74476 | 44,636639 | 64,2526196 | 59,0001342 | 70,3562074 | 64,8495876 |
| 32,89882 | 44,65873 | 64,2603195 | 59,0012017 | 70,3733655 | 64,8828565 |
| 33,09459 | 44,6865709 | 64,2646739 | 59,0064391 | 70,4178617 | 64,8989603 |
| 33,12862 | 44,7888513 | 64,2664024 | 59,0144906 | 70,4208098 | 64,9259642 |
| 33,20285 | 44,8351017 | 64,2929549 | 59,0284905 | 70,4214074 | 64,968677 |
| 33,33988 | 45,0460531 | 64,3111338 | 59,0830744 | 70,4368493 | 64,9730648 |
| 33,41059 | 45,1063189 | 64,3371036 | 59,1142479 | 70,4899945 | 64,98442 |
| 33,72174 | 45,1067114 | 64,3452453 | 59,1835016 | 70,5033799 | 65,009178 |
| 33,79723 | 45,126057 | 64,3654667 | 59,2125072 | 70,5685832 | 65,0250724 |
| 33,91014 | 45,2285971 | 64,3752497 | 59,2388446 | 70,5726638 | 65,0797962 |
| 34,04421 | 45,2878031 | 64,381783 | 59,255904 | 70,5980984 | 65,0904137 |
| 34,07151 | 45,2959461 | 64,4109781 | 59,3391352 | 70,607461 | 65,1028063 |
| 34,09058 | 45,3045371 | 64,4142443 | 59,3435604 | 70,6295523 | 65,151237 |
| 34,09325 | 45,3412552 | 64,492863 | 59,3998284 | 70,6589897 | 65,2958685 |
| 34,13175 | 45,4552965 | 64,4928818 | 59,4974337 | 70,7255025 | 65,2992424 |
| 34,20819 | 45,6830505 | 64,5014064 | 59,5395107 | 70,784647 | 65,2995195 |
| 34,25954 | 45,7899872 | 64,5397816 | 59,5538181 | 70,8060955 | 65,3299984 |
| 34,37192 | 45,8550493 | 64,5523244 | 59,6091396 | 70,8067417 | 65,3486917 |
| 34,50767 | 45,8927314 | 64,5689964 | 59,6805712 | 70,8426803 | 65,4152014 |
| 34,58408 | 45,9109343 | 64,5808446 | 59,6857735 | 70,8559686 | 65,4425926 |
| 34,59804 | 45,9418512 | 64,6187564 | 59,7444965 | 70,8993927 | 65,4495148 |
| 34,69872 | 46,3152387 | 64,6264789 | 59,7636488 | 70,9157587 | 65,4839208 |
| 34,85653 | 46,3435895 | 64,6304637 | 59,787237 | 70,9237473 | 65,5689426 |
| 34,88133 | 46,5430521 | 64,677347 | 59,8029245 | 70,9440355 | 65,5941772 |
| 35,13024 | 46,5541334 | 64,708557 | 59,8188033 | 70,9824025 | 65,5983624 |
| 35,15526 | 46,5887603 | 64,7192402 | 59,9592121 | 70,9855211 | 65,6221005 |
| 35,16461 | 46,6180988 | 64,7401679 | 59,9799679 | 71,0006095 | 65,6307878 |
| 35,17166 | 46,6235404 | 64,7745317 | 60,0140715 | 71,0169382 | 65,6345264 |
| 35,24804 | 46,9671658 | 64,7870499 | 60,0154854 | 71,0589777 | 65,6564878 |
| 35,25643 | 47,2753148 | 64,7950485 | 60,0163644 | 71,0943899 | 65,6608045 |
| 35,29564 | 47,3860901 | 64,8039817 | 60,054121 | 71,114688 | 65,6767881 |
| 35,29818 | 47,4191613 | 64,8136968 | 60,063585 | 71,1178881 | 65,6987399 |
| 35,3716 | 47,5610722 | 64,8242177 | 60,1144451 | 71,1318838 | 65,7079901 |
| 35,4025 | 47,5816541 | 64,8588007 | 60,1838973 | 71,1404571 | 65,7422026 |
| 35,46023 | 47,5824306 | 64,9047769 | 60,1933011 | 71,1556058 | 65,9098155 |

| | | | | | |
|---|---|---|---|---|---|
| 35,52787 | 47,6631379 | 64,9187647 | 60,2334062 | 71,2499125 | 65,9141073 |
| 35,63113 | 47,6928369 | 65,0083631 | 60,2384193 | 71,2539007 | 65,9196998 |
| 35,71497 | 47,8342655 | 65,0292983 | 60,345003 | 71,2787532 | 65,9252699 |
| 35,80177 | 47,8553659 | 65,0608342 | 60,3785739 | 71,3446877 | 65,9326298 |
| 35,87529 | 48,0276204 | 65,1567964 | 60,3983376 | 71,4202198 | 65,9676971 |
| 35,87551 | 48,3553992 | 65,1637033 | 60,3988782 | 71,4609358 | 65,9716548 |
| 35,90559 | 48,5142102 | 65,1851856 | 60,4489899 | 71,4705632 | 65,9775958 |
| 36,29756 | 48,6577386 | 65,2095279 | 60,4560004 | 71,6388867 | 65,9958744 |
| 36,83513 | 48,6848475 | 65,2119329 | 60,4736602 | 71,643649 | 66,0238494 |
| 36,84012 | 48,874488 | 65,2515029 | 60,4789518 | 71,6668782 | 66,1121837 |
| 36,92494 | 48,932389 | 65,252462 | 60,513664 | 71,6703897 | 66,2640406 |
| 36,93928 | 48,9394402 | 65,256366 | 60,5597072 | 71,6807816 | 66,2744327 |
| 37,10027 | 49,0186912 | 65,2858088 | 60,6250581 | 71,6900207 | 66,3039115 |
| 37,15568 | 49,0223209 | 65,2905317 | 60,6900115 | 71,7066475 | 66,333136 |
| 37,19446 | 49,0357046 | 65,3055116 | 60,694483 | 71,7118508 | 66,3487148 |
| 37,28203 | 49,1396166 | 65,3086838 | 60,7304035 | 71,7340932 | 66,3584796 |
| 37,29998 | 49,1509813 | 65,390256 | 60,7478149 | 71,735111 | 66,3797664 |
| 37,47048 | 49,4931143 | 65,3960653 | 60,7576102 | 71,7781677 | 66,4037023 |
| 37,49784 | 49,6186363 | 65,4489069 | 60,7759465 | 71,8371464 | 66,4059492 |
| 37,51802 | 49,6953464 | 65,457904 | 60,8167922 | 71,8730142 | 66,4886741 |
| 37,53637 | 49,8672699 | 65,462802 | 60,8717546 | 71,9148619 | 66,548592 |
| 37,78539 | 49,9751021 | 65,4713807 | 60,9012538 | 71,919338 | 66,581007 |
| 37,81149 | 50,1024472 | 65,4909084 | 60,9067136 | 71,9230258 | 66,6264268 |
| 37,81468 | 50,1566961 | 65,4966056 | 60,9539122 | 71,9240834 | 66,6357742 |
| 37,82259 | 50,4019607 | 65,5173779 | 60,9645208 | 71,9944345 | 66,6603203 |
| 37,93033 | 50,4173397 | 65,5498889 | 61,0174434 | 72,0037884 | 66,6698617 |
| 38,04031 | 50,4533455 | 65,5628898 | 61,1244332 | 72,0119665 | 66,7180251 |
| 38,05329 | 50,6058282 | 65,585271 | 61,1434526 | 72,0398205 | 66,7352525 |
| 38,21504 | 50,8608926 | 65,6125915 | 61,1526752 | 72,0818862 | 66,8181625 |
| 38,36451 | 50,9484649 | 65,6170796 | 61,1562309 | 72,1013005 | 66,8316081 |
| 38,60625 | 51,2327188 | 65,6299052 | 61,1685018 | 72,1039903 | 66,8494396 |
| 38,86511 | 51,2895518 | 65,6335908 | 61,2412739 | 72,1923595 | 66,8739341 |
| 39,0513 | 51,3208828 | 65,6508416 | 61,328472 | 72,1987264 | 66,8877579 |
| 39,05402 | 51,3403135 | 65,6511701 | 61,3867806 | 72,2066356 | 66,9530807 |
| 39,09043 | 51,3845591 | 65,6628565 | 61,43817 | 72,2157482 | 66,9827732 |
| 39,15196 | 51,4633103 | 65,6734463 | 61,4761969 | 72,2330304 | 66,9942042 |
| 39,2305 | 51,5738266 | 65,6790054 | 61,4884498 | 72,2728715 | 67,019145 |
| 39,24424 | 51,6798934 | 65,6896007 | 61,4896513 | 72,2939472 | 67,0597343 |
| 39,29502 | 51,6966635 | 65,7508089 | 61,4931588 | 72,2969199 | 67,0952147 |
| 39,35839 | 51,7161741 | 65,7805366 | 61,5113382 | 72,3597714 | 67,152206 |
| 39,5964 | 51,8165517 | 65,7895002 | 61,5191793 | 72,3662161 | 67,1831366 |
| 39,64556 | 51,828871 | 65,8525399 | 61,5462448 | 72,3776268 | 67,1978754 |
| 39,67139 | 51,8642709 | 65,8541088 | 61,5881461 | 72,386221 | 67,2244398 |
| 39,77666 | 51,8660004 | 65,8800741 | 61,5910547 | 72,3973061 | 67,226737 |
| 39,83699 | 51,9372746 | 65,887829 | 61,6387525 | 72,461675 | 67,2333221 |
| 39,87356 | 51,9740458 | 65,9628041 | 61,6566487 | 72,4778015 | 67,2585019 |
| 39,91418 | 51,9927511 | 66,0103343 | 61,7106844 | 72,5119826 | 67,3048667 |

| | | | | | |
|---|---|---|---|---|---|
| 39,98754 | 52,0022537 | 66,0128655 | 61,7902632 | 72,5274304 | 67,3628986 |
| 40,17349 | 52,0486466 | 66,0411902 | 61,8379992 | 72,5404611 | 67,3931369 |
| 40,1813 | 52,1551047 | 66,0419105 | 61,8579069 | 72,5493642 | 67,3978023 |
| 40,22885 | 52,4840461 | 66,0421691 | 61,8678576 | 72,5947252 | 67,4262125 |
| 40,2601 | 52,6709042 | 66,0611129 | 61,8707735 | 72,6031047 | 67,4867552 |
| 40,3255 | 52,7170506 | 66,0735644 | 61,9463354 | 72,60974 | 67,5310074 |
| 40,42271 | 52,7417316 | 66,1123862 | 61,9567593 | 72,6226912 | 67,549534 |
| 40,42595 | 52,8469302 | 66,1181954 | 61,9815757 | 72,653166 | 67,5741361 |
| 40,46442 | 52,9313474 | 66,1328823 | 61,9966256 | 72,7026548 | 67,6273984 |
| 40,47578 | 53,0058516 | 66,1595975 | 62,0207556 | 72,7244232 | 67,6627633 |
| 40,51885 | 53,043193 | 66,1856828 | 62,058709 | 72,7428085 | 67,7205786 |
| 40,53887 | 53,0995729 | 66,2214405 | 62,0778676 | 72,7737436 | 67,7398676 |
| 40,54769 | 53,250435 | 66,2514797 | 62,0841098 | 72,7771305 | 67,768116 |
| 40,55157 | 53,315501 | 66,2589556 | 62,1436624 | 72,7883156 | 67,7893081 |
| 40,722 | 53,3321731 | 66,2762639 | 62,1973047 | 72,8113952 | 67,8431063 |
| 40,76968 | 53,6839518 | 66,2779171 | 62,2221481 | 72,8873327 | 67,8983639 |
| 40,78914 | 53,9401683 | 66,3266919 | 62,3064722 | 72,904345 | 67,8989774 |
| 40,82154 | 53,942804 | 66,3288581 | 62,3504309 | 72,9093961 | 67,9829182 |
| 40,82789 | 53,9844371 | 66,3358414 | 62,35767 | 72,9474421 | 68,0578859 |
| 40,89575 | 54,0157448 | 66,3395817 | 62,3713477 | 72,9717593 | 68,062167 |
| 41,05235 | 54,0420974 | 66,3674378 | 62,3747183 | 72,9730681 | 68,1585781 |
| 41,10656 | 54,1555638 | 66,3820784 | 62,3945896 | 72,9827253 | 68,1742767 |
| 41,33259 | 54,3919108 | 66,3931385 | 62,4153264 | 73,0181428 | 68,2155608 |
| 41,40959 | 54,6012548 | 66,4103582 | 62,4421989 | 73,0197747 | 68,259115 |
| 41,52091 | 54,8958754 | 66,4265281 | 62,4945206 | 73,0461146 | 68,2699217 |
| 41,69433 | 54,9865582 | 66,4694363 | 62,4969947 | 73,0636034 | 68,2943253 |
| 41,83476 | 55,1492132 | 66,4716959 | 62,5238452 | 73,0665072 | 68,32386 |
| 41,88724 | 55,186342 | 66,5103602 | 62,5339825 | 73,0694739 | 68,4105582 |
| 41,89098 | 55,2228447 | 66,5243968 | 62,5554465 | 73,0788237 | 68,4160052 |
| 42,247 | 55,2989648 | 66,5277155 | 62,5673965 | 73,0966595 | 68,4226847 |
| 42,2793 | 55,3060514 | 66,5644641 | 62,6027965 | 73,1367599 | 68,4331184 |
| 42,29176 | 55,3650724 | 66,5754453 | 62,634071 | 73,204327 | 68,5145069 |
| 42,33346 | 55,4094605 | 66,5810147 | 62,6358814 | 73,2361521 | 68,5609506 |
| 42,3836 | 55,510718 | 66,5999429 | 62,7094539 | 73,2517925 | 68,6229931 |
| 42,41839 | 55,690433 | 66,6163582 | 62,7282388 | 73,2902437 | 68,6237597 |
| 42,50458 | 55,741649 | 66,6300365 | 62,7331818 | 73,2913896 | 68,6362588 |
| 42,51471 | 55,777507 | 66,6351406 | 62,7786211 | 73,3183063 | 68,6490909 |
| 42,51692 | 55,8077324 | 66,6599087 | 62,8195392 | 73,4024971 | 68,6610028 |
| 42,7332 | 55,8518701 | 66,7317205 | 62,8199893 | 73,4299995 | 68,7263626 |
| 42,80636 | 55,9215668 | 66,7625058 | 62,8505912 | 73,4363695 | 68,7737725 |
| 42,97587 | 55,9449441 | 66,7724808 | 62,8850581 | 73,4811062 | 68,8055662 |
| 43,00333 | 55,9780364 | 66,799072 | 62,9220243 | 73,5354206 | 68,8264789 |
| 43,15773 | 56,0679347 | 66,8253348 | 62,9402779 | 73,5408 | 68,8632441 |
| 43,2336 | 56,0912389 | 66,8817347 | 62,9754242 | 73,5527657 | 68,9690955 |
| 43,2687 | 56,3848772 | 66,909423 | 63,0155821 | 73,5635177 | 68,9779702 |
| 43,34687 | 56,470947 | 66,9242467 | 63,0479912 | 73,5914146 | 69,0059297 |
| 43,35063 | 56,5092164 | 66,9819759 | 63,0758956 | 73,6355844 | 69,0465198 |

| | | | | | |
|---|---|---|---|---|---|
| 43,44013 | 56,5189689 | 67,0119334 | 63,0809939 | 73,6415415 | 69,0751782 |
| 43,4769 | 56,593895 | 67,0181422 | 63,109717 | 73,650281 | 69,1180048 |
| 43,5937 | 56,7678365 | 67,0501312 | 63,1985909 | 73,7368874 | 69,1450133 |
| 43,65622 | 56,8945052 | 67,0592222 | 63,2553404 | 73,7392419 | 69,1487897 |
| 43,83609 | 57,1337018 | 67,074421 | 63,2561063 | 73,8417308 | 69,208892 |
| 43,89903 | 57,1764172 | 67,0921746 | 63,2786553 | 73,8717393 | 69,2132679 |
| 43,96639 | 57,2123497 | 67,1063876 | 63,2899966 | 73,9496363 | 69,2366003 |
| 43,98922 | 57,2203584 | 67,1474247 | 63,3168504 | 73,9739325 | 69,2630992 |
| 43,99706 | 57,2448404 | 67,1508335 | 63,3241595 | 73,9808935 | 69,2638024 |
| 44,06985 | 57,4072718 | 67,1870014 | 63,327083 | 73,9848435 | 69,2841505 |
| 44,14711 | 57,4251087 | 67,1878324 | 63,3442211 | 74,0895476 | 69,2896319 |
| 44,16876 | 57,6883809 | 67,1924271 | 63,3751195 | 74,1130599 | 69,3817558 |
| 44,32681 | 57,8699395 | 67,2136678 | 63,3768183 | 74,1136924 | 69,3901118 |
| 44,36168 | 57,8988189 | 67,2183671 | 63,384087 | 74,1167796 | 69,3935551 |
| 44,47104 | 57,9588758 | 67,2766228 | 63,3988781 | 74,1460408 | 69,4552802 |
| 44,55688 | 57,9735687 | 67,2791334 | 63,4541892 | 74,169127 | 69,4637533 |
| 44,61487 | 58,2515263 | 67,3173586 | 63,5520153 | 74,1934136 | 69,4923433 |
| 44,71036 | 58,368398 | 67,3226633 | 63,6586787 | 74,2213495 | 69,5250804 |
| 44,7197 | 58,4523476 | 67,3364844 | 63,679314 | 74,2395253 | 69,5407003 |
| 44,71987 | 58,8954862 | 67,357344 | 63,6945506 | 74,263296 | 69,5440966 |
| 44,89978 | 58,984802 | 67,3632447 | 63,7490486 | 74,2706489 | 69,5686117 |
| 45,05094 | 59,0552771 | 67,3706048 | 63,8257898 | 74,2908333 | 69,6209136 |
| 45,07757 | 59,1501124 | 67,371149 | 63,861361 | 74,3280968 | 69,654572 |
| 45,23613 | 59,1872088 | 67,3773271 | 63,9673785 | 74,3604194 | 69,6966882 |
| 45,27349 | 59,2947301 | 67,4223659 | 63,9683486 | 74,366661 | 69,6988981 |
| 45,33238 | 59,359454 | 67,4251265 | 63,9684019 | 74,3751743 | 69,7394543 |
| 45,43403 | 59,5516758 | 67,4370903 | 64,0097612 | 74,3757006 | 69,741339 |
| 45,43666 | 59,5941933 | 67,4782631 | 64,0146339 | 74,4666861 | 69,7478171 |
| 45,4872 | 59,6850135 | 67,4942824 | 64,0217077 | 74,501424 | 69,7753546 |
| 45,66126 | 59,8063012 | 67,5145047 | 64,0780483 | 74,552889 | 69,7898021 |
| 45,68667 | 60,3471175 | 67,515707 | 64,1222413 | 74,5545376 | 69,8102902 |
| 45,7718 | 60,4078212 | 67,5781186 | 64,1551141 | 74,6032457 | 69,8130949 |
| 45,77305 | 60,4224338 | 67,5914969 | 64,2954919 | 74,6372311 | 69,8296357 |
| 45,79241 | 60,4981455 | 67,6094581 | 64,3014684 | 74,6392743 | 69,8614194 |
| 45,80194 | 60,5165612 | 67,6115567 | 64,313663 | 74,6477319 | 69,8876213 |
| 45,82154 | 60,6082671 | 67,6203603 | 64,3447022 | 74,6824338 | 69,9005896 |
| 45,82318 | 60,6481553 | 67,6352199 | 64,4348301 | 74,771836 | 69,9508156 |
| 45,93826 | 61,0399529 | 67,6375747 | 64,4359687 | 74,7745613 | 69,9624007 |
| 46,12306 | 61,0439489 | 67,6508291 | 64,4368267 | 74,8423331 | 69,9912056 |
| 46,13395 | 61,0577097 | 67,664854 | 64,4466704 | 74,8469496 | 70,0345052 |
| 46,2337 | 61,059588 | 67,6869653 | 64,4607855 | 74,8830043 | 70,0518495 |
| 46,29307 | 61,372791 | 67,6905662 | 64,4729258 | 74,9925708 | 70,0726217 |
| 46,40134 | 61,3950956 | 67,6990393 | 64,5511049 | 74,9977421 | 70,0891089 |
| 46,52011 | 61,4314471 | 67,7076454 | 64,5534386 | 75,0038644 | 70,161444 |
| 46,59187 | 61,525881 | 67,7389156 | 64,6230108 | 75,0168963 | 70,2079378 |
| 46,63666 | 62,0360017 | 67,7527929 | 64,647955 | 75,0227415 | 70,2692423 |
| 46,70063 | 62,0807199 | 67,8471923 | 64,7051684 | 75,0229019 | 70,3246166 |

| | | | | | |
|---|---|---|---|---|---|
| 46,70084 | 62,0859903 | 67,8572834 | 64,8299591 | 75,0299761 | 70,3267939 |
| 46,7328 | 62,1355954 | 67,8704454 | 64,9280082 | 75,0328285 | 70,3513164 |
| 46,7451 | 62,1836781 | 67,8824621 | 64,97029 | 75,0391578 | 70,4199364 |
| 46,76284 | 62,2089447 | 67,911196 | 65,0973546 | 75,097057 | 70,5265209 |
| 46,82147 | 62,6404855 | 67,9299719 | 65,1667337 | 75,0985652 | 70,5352914 |
| 46,85046 | 62,8420061 | 68,0178954 | 65,1950201 | 75,1511156 | 70,5396283 |
| 46,8594 | 62,9752609 | 68,0218384 | 65,2272494 | 75,1561119 | 70,5598007 |
| 46,86619 | 63,047705 | 68,0240769 | 65,229312 | 75,1694619 | 70,5708245 |
| 46,86951 | 63,0904207 | 68,0474089 | 65,2509147 | 75,1698645 | 70,5738118 |
| 46,9138 | 63,1200416 | 68,125307 | 65,2860506 | 75,2385989 | 70,5835566 |
| 46,96747 | 63,3886301 | 68,1630908 | 65,2901427 | 75,2419557 | 70,5864911 |
| 47,00557 | 63,4226232 | 68,1845957 | 65,2916918 | 75,253987 | 70,5895065 |
| 47,0496 | 63,4434382 | 68,1885186 | 65,3441967 | 75,2583277 | 70,5939066 |
| 47,08413 | 63,5192483 | 68,2041899 | 65,3974217 | 75,2766418 | 70,6046075 |
| 47,30341 | 63,6280064 | 68,2775976 | 65,4157477 | 75,2893925 | 70,6123993 |
| 47,37574 | 63,7048294 | 68,2835187 | 65,455796 | 75,3102577 | 70,6379456 |
| 47,39334 | 63,7049852 | 68,3460075 | 65,4903074 | 75,3108116 | 70,65471 |
| 47,52226 | 63,7081083 | 68,3556716 | 65,6668514 | 75,3159455 | 70,6602684 |
| 47,56966 | 64,4768512 | 68,4397825 | 65,6990105 | 75,3163866 | 70,6614049 |
| 47,64728 | 64,6061902 | 68,4435231 | 65,7817914 | 75,3553078 | 70,6615871 |
| 47,66852 | 64,7633577 | 68,5256821 | 65,8313418 | 75,3964118 | 70,6848553 |
| 47,72573 | 64,8408074 | 68,5387361 | 65,8932583 | 75,4125801 | 70,6979302 |
| 47,81333 | 64,9064116 | 68,5593242 | 65,9085828 | 75,4264028 | 70,7169565 |
| 47,88691 | 64,9241088 | 68,5691669 | 65,9528067 | 75,4386503 | 70,7468907 |
| 47,89218 | 65,1368853 | 68,612419 | 66,0083655 | 75,4416701 | 70,7499421 |
| 47,89942 | 65,1677425 | 68,65607 | 66,0316607 | 75,6271445 | 70,8279115 |
| 47,90834 | 65,2426813 | 68,6639716 | 66,0347152 | 75,6483574 | 70,8761288 |
| 47,93306 | 65,3164023 | 68,6842442 | 66,0629015 | 75,6918263 | 70,8869544 |
| 47,95685 | 65,3281717 | 68,7751294 | 66,1215483 | 75,6979994 | 70,9080965 |
| 47,98239 | 65,4287613 | 68,7798279 | 66,1667743 | 75,7211332 | 70,94497 |
| 48,03543 | 65,5359911 | 68,7859133 | 66,2120598 | 75,7404243 | 70,948399 |
| 48,05049 | 65,6299537 | 68,7985406 | 66,2292785 | 75,8152985 | 71,0184224 |
| 48,09064 | 65,6852361 | 68,8398499 | 66,2581791 | 75,8196361 | 71,0538678 |
| 48,10412 | 65,6895996 | 68,8675851 | 66,3482622 | 75,8199935 | 71,1155467 |
| 48,18714 | 65,7460549 | 68,9408836 | 66,3583446 | 75,8335009 | 71,1273647 |
| 48,20238 | 65,7847845 | 69,0152792 | 66,3914964 | 75,8361042 | 71,1352506 |
| 48,27032 | 65,9241041 | 69,0166549 | 66,4067856 | 75,8545985 | 71,141158 |
| 48,28923 | 66,0006286 | 69,0233624 | 66,4606175 | 75,9143253 | 71,2118668 |
| 48,29151 | 66,0674377 | 69,0424073 | 66,4606932 | 75,9423765 | 71,2159181 |
| 48,3036 | 66,210434 | 69,0546638 | 66,5183155 | 75,9532136 | 71,2362934 |
| 48,30937 | 66,2404766 | 69,0878606 | 66,5732442 | 76,0001915 | 71,2726553 |
| 48,33664 | 66,2907261 | 69,0890258 | 66,6181199 | 76,0304878 | 71,3120306 |
| 48,39716 | 66,4242333 | 69,0924889 | 66,6346224 | 76,0455921 | 71,4000332 |
| 48,41113 | 66,4652299 | 69,1311304 | 66,6701968 | 76,0502547 | 71,4105611 |
| 48,45264 | 66,6574922 | 69,1538789 | 66,7280726 | 76,1026007 | 71,5010452 |
| 48,48636 | 66,8261192 | 69,1573164 | 66,7452849 | 76,1161664 | 71,5012319 |
| 48,50826 | 66,9316232 | 69,1764883 | 66,7664546 | 76,1178146 | 71,5993632 |

| | | | | | |
|---|---|---|---|---|---|
| 48,64165 | 66,9666196 | 69,1792101 | 66,7987884 | 76,1251715 | 71,6501568 |
| 48,64754 | 67,2812999 | 69,1973093 | 66,8169185 | 76,1659377 | 71,6516878 |
| 48,68525 | 67,312331 | 69,2038944 | 66,8420666 | 76,1873363 | 71,6574592 |
| 48,74263 | 67,6230576 | 69,2387322 | 66,9448304 | 76,1952834 | 71,6700147 |
| 48,75605 | 67,6327411 | 69,2810877 | 66,9698739 | 76,2047463 | 71,6880671 |
| 48,78686 | 67,6415779 | 69,2820811 | 67,0128174 | 76,220896 | 71,7746679 |
| 48,81735 | 67,6607775 | 69,3005843 | 67,050273 | 76,25611 | 71,7866371 |
| 48,91692 | 67,7389461 | 69,3008592 | 67,0555048 | 76,287985 | 71,811571 |
| 48,99883 | 67,7424565 | 69,3241968 | 67,0897254 | 76,3280439 | 71,8345419 |
| 49,07297 | 67,8088714 | 69,3289801 | 67,1445672 | 76,3313188 | 71,8670688 |
| 49,12505 | 67,8986566 | 69,3335502 | 67,2028068 | 76,3546278 | 71,9554376 |
| 49,12974 | 68,2009498 | 69,3560504 | 67,2222837 | 76,3734946 | 71,9960293 |
| 49,1934 | 68,2182799 | 69,3912762 | 67,3695531 | 76,423306 | 72,0000898 |
| 49,21997 | 68,353908 | 69,4237794 | 67,3951706 | 76,4453386 | 72,0168685 |
| 49,29032 | 68,4156526 | 69,4386615 | 67,4666592 | 76,469509 | 72,034799 |
| 49,42329 | 68,4479424 | 69,4449365 | 67,4886364 | 76,4778908 | 72,1521702 |
| 49,62336 | 68,4646168 | 69,4449402 | 67,5330708 | 76,5063487 | 72,1547786 |
| 49,70155 | 68,6927697 | 69,4895126 | 67,6416771 | 76,5726948 | 72,1724276 |
| 49,82648 | 68,8260577 | 69,4934318 | 67,6586175 | 76,5986314 | 72,1838355 |
| 49,83556 | 68,8320719 | 69,5147731 | 67,6779071 | 76,599355 | 72,2291856 |
| 49,98419 | 68,9478558 | 69,5257551 | 67,7091292 | 76,6135734 | 72,2455721 |
| 50,00272 | 68,9800614 | 69,5365307 | 67,7117699 | 76,6567237 | 72,265254 |
| 50,14682 | 69,156638 | 69,5473902 | 67,7621812 | 76,657302 | 72,2846761 |
| 50,21209 | 69,3801374 | 69,5715021 | 67,7623786 | 76,6582487 | 72,3092164 |
| 50,41913 | 69,4054526 | 69,5716588 | 67,766262 | 76,6607912 | 72,4033459 |
| 50,49949 | 69,4587427 | 69,6032329 | 67,7776732 | 76,7136702 | 72,4325789 |
| 50,84175 | 69,4897712 | 69,605608 | 67,7793217 | 76,7399596 | 72,4335466 |
| 50,89696 | 69,8738475 | 69,6187258 | 67,8572294 | 76,8402448 | 72,4335993 |
| 50,95552 | 69,8851453 | 69,6264591 | 67,979136 | 76,9187263 | 72,4656211 |
| 50,97489 | 70,2216472 | 69,6303311 | 67,9823002 | 76,928399 | 72,4727714 |
| 51,16525 | 70,4264069 | 69,6394111 | 68,0110821 | 76,9875503 | 72,4780979 |
| 51,35883 | 70,7895469 | 69,6514948 | 68,1017837 | 77,0249864 | 72,4798372 |
| 51,40092 | 70,8722765 | 69,7506424 | 68,1106058 | 77,0285386 | 72,5340853 |
| 51,49327 | 70,8898472 | 69,7742739 | 68,1232459 | 77,0773968 | 72,5404166 |
| 51,52935 | 71,143568 | 69,8432322 | 68,1469454 | 77,0854463 | 72,5408178 |
| 51,60045 | 71,2639805 | 69,927714 | 68,15095 | 77,0883563 | 72,5698989 |
| 51,66473 | 71,5927836 | 69,9432696 | 68,1647849 | 77,3352834 | 72,5878716 |
| 51,69035 | 71,6070689 | 69,9702544 | 68,2007242 | 77,4240085 | 72,6303524 |
| 51,73133 | 71,630147 | 69,9892746 | 68,3028931 | 77,4305984 | 72,6579315 |
| 51,75423 | 72,4995984 | 70,0554209 | 68,4046148 | 77,4662144 | 72,6944977 |
| 51,7622 | 72,5669053 | 70,200264 | 68,4071216 | 77,5017546 | 72,700139 |
| 51,77262 | 72,7400315 | 70,2395008 | 68,4353508 | 77,5055342 | 72,7529194 |
| 52,00178 | 72,752124 | 70,2534043 | 68,5031633 | 77,5263407 | 72,8269041 |
| 52,0082 | 72,8825418 | 70,2573313 | 68,5261355 | 77,5266907 | 72,8842444 |
| 52,26117 | 72,9319407 | 70,2925824 | 68,5290511 | 77,5536375 | 72,9102577 |
| 52,29263 | 73,0258378 | 70,3022428 | 68,5310469 | 77,5563723 | 72,9193211 |
| 52,4389 | 73,0611561 | 70,3259148 | 68,5467122 | 77,5665769 | 72,9431522 |

| | | | | | |
|---|---|---|---|---|---|
| 52,45358 | 73,1053392 | 70,3319303 | 68,5740769 | 77,6131262 | 72,9564737 |
| 52,53539 | 73,2133464 | 70,3366728 | 68,5957576 | 77,7168808 | 72,9940317 |
| 52,66406 | 73,3026855 | 70,3446701 | 68,6362536 | 77,7864678 | 73,0005296 |
| 52,74292 | 73,3285578 | 70,3500511 | 68,6722675 | 77,8810249 | 73,0192823 |
| 52,7494 | 73,3557573 | 70,3840568 | 68,6726161 | 77,9637336 | 73,2371333 |
| 52,75849 | 73,3699272 | 70,5219381 | 68,6883475 | 77,9748836 | 73,2425672 |
| 52,83555 | 73,4283069 | 70,6289571 | 68,7054829 | 78,0266119 | 73,2688371 |
| 52,97152 | 73,5682353 | 70,6542108 | 68,7784728 | 78,0949224 | 73,3191214 |
| 52,99951 | 73,6189124 | 70,6736865 | 68,9643797 | 78,0971005 | 73,3387415 |
| 53,07841 | 73,6538464 | 70,6874106 | 69,0033085 | 78,1259637 | 73,3718976 |
| 53,11247 | 73,8016424 | 70,6888442 | 69,0372054 | 78,2106053 | 73,3727407 |
| 53,18284 | 73,8228827 | 70,7054685 | 69,0548285 | 78,3049696 | 73,4123528 |
| 53,31772 | 73,9528893 | 70,7507281 | 69,1030691 | 78,3197115 | 73,4651304 |
| 53,3352 | 74,0440911 | 70,7908973 | 69,1996043 | 78,3378437 | 73,474186 |
| 53,47094 | 74,1951088 | 70,8814858 | 69,2028296 | 78,3727225 | 73,5018944 |
| 53,49501 | 74,2040879 | 70,8952037 | 69,2661747 | 78,3779327 | 73,5022921 |
| 53,51417 | 74,2445429 | 70,9200196 | 69,2973845 | 78,3993539 | 73,5097336 |
| 53,54856 | 74,2754344 | 71,0051231 | 69,3378527 | 78,4358153 | 73,511026 |
| 53,55809 | 74,4471796 | 71,009836 | 69,3664252 | 78,486128 | 73,5298352 |
| 53,60745 | 74,9520261 | 71,0909449 | 69,4068619 | 78,5103637 | 73,559967 |
| 53,99246 | 74,9990386 | 71,1047097 | 69,4381475 | 78,5183931 | 73,5615509 |
| 54,02014 | 75,0757529 | 71,1204424 | 69,4539941 | 78,5539651 | 73,614086 |
| 54,0595 | 75,1952383 | 71,1249715 | 69,4677365 | 78,5603594 | 73,6410358 |
| 54,06432 | 75,1972098 | 71,1536208 | 69,4684187 | 78,5783478 | 73,6874046 |
| 54,11428 | 75,3762399 | 71,2542239 | 69,5185992 | 78,6143969 | 73,7672673 |
| 54,18086 | 75,6045466 | 71,3287949 | 69,5969111 | 78,6241424 | 73,8026045 |
| 54,27394 | 76,1241278 | 71,336157 | 69,6492174 | 78,6302376 | 73,8351387 |
| 54,36022 | 76,3482091 | 71,3431475 | 69,6567153 | 78,631035 | 73,840192 |
| 54,68713 | 76,5310482 | 71,3864174 | 69,6863426 | 78,6694686 | 73,8609398 |
| 54,81986 | 76,6184048 | 71,4591832 | 69,8092912 | 78,6696726 | 73,8684389 |
| 54,89597 | 76,6987972 | 71,5261954 | 69,8203808 | 78,684583 | 73,8698424 |
| 55,33637 | 76,7699832 | 71,5740681 | 69,8463513 | 78,7665519 | 73,9273845 |
| 55,38344 | 76,8300522 | 71,5843619 | 69,9832554 | 78,7974751 | 74,039541 |
| 55,55924 | 76,9156149 | 71,6386471 | 69,9947205 | 78,8371665 | 74,0694878 |
| 55,72275 | 77,1475502 | 71,6481208 | 70,0061961 | 78,8826271 | 74,0769097 |
| 55,87452 | 77,2526381 | 71,7100161 | 70,1265301 | 78,9343015 | 74,101956 |
| 56,01665 | 77,2601163 | 71,7261179 | 70,1852576 | 78,9652256 | 74,1857422 |
| 56,0172 | 77,2705999 | 71,7441161 | 70,2127952 | 78,9755946 | 74,2849879 |
| 56,15701 | 77,4000131 | 71,7503791 | 70,2274004 | 79,015417 | 74,3053416 |
| 56,43668 | 77,5020704 | 71,773208 | 70,2389726 | 79,1028358 | 74,3062103 |
| 56,52701 | 77,6827689 | 71,7750288 | 70,2897758 | 79,1701151 | 74,3320742 |
| 56,56279 | 77,7528747 | 71,8084623 | 70,321852 | 79,1786907 | 74,3738723 |
| 56,58132 | 77,9615787 | 71,8642725 | 70,3633083 | 79,2066659 | 74,4613307 |
| 56,60078 | 78,1387187 | 71,8658912 | 70,3941172 | 79,2123657 | 74,4896987 |
| 56,7399 | 78,202063 | 71,9350361 | 70,487834 | 79,2171319 | 74,4982941 |
| 56,99831 | 78,3615644 | 71,9518574 | 70,5889195 | 79,242802 | 74,514369 |
| 57,22045 | 78,7878516 | 71,9635023 | 70,6344542 | 79,2692979 | 74,5227733 |

| | | | | | |
|---|---|---|---|---|---|
| 57,23592 | 78,895452 | 72,0716211 | 70,6675964 | 79,2965679 | 74,5391286 |
| 57,29357 | 78,9272474 | 72,1181211 | 70,6897964 | 79,3023267 | 74,5661212 |
| 57,30056 | 78,9608226 | 72,1400777 | 70,6907458 | 79,3410943 | 74,5900486 |
| 57,37966 | 79,0081626 | 72,1519534 | 70,7309636 | 79,3538843 | 74,593055 |
| 57,48969 | 79,1144703 | 72,1805286 | 70,7370995 | 79,3691626 | 74,6035212 |
| 57,58981 | 79,2469322 | 72,2064774 | 70,7880831 | 79,3728809 | 74,6070018 |
| 57,66175 | 79,3024209 | 72,261885 | 70,7906134 | 79,3849406 | 74,6353926 |
| 57,74795 | 79,5931613 | 72,2767166 | 70,8511558 | 79,4087622 | 74,6520688 |
| 57,78905 | 79,6397024 | 72,3126658 | 70,8862923 | 79,4156666 | 74,7211945 |
| 57,79494 | 79,9058811 | 72,3441537 | 70,9138026 | 79,4185118 | 74,7260247 |
| 57,8185 | 79,9501934 | 72,4185355 | 70,9866246 | 79,4473717 | 74,8540494 |
| 57,86529 | 80,0113664 | 72,4300234 | 70,9894255 | 79,5237923 | 74,9171898 |
| 57,95357 | 80,0897965 | 72,5039188 | 70,9913769 | 79,6163964 | 74,9655593 |
| 58,13446 | 80,2710691 | 72,5106477 | 71,0078785 | 79,6388861 | 74,9689374 |
| 58,78702 | 80,4035627 | 72,5750571 | 71,0322836 | 79,7576103 | 74,9829197 |
| 58,8565 | 80,4640285 | 72,5852126 | 71,0475445 | 79,79729 | 75,1284041 |
| 59,20486 | 80,5219012 | 72,6750943 | 71,0660857 | 79,8654413 | 75,1442104 |
| 59,29544 | 80,5616888 | 72,6751867 | 71,1002604 | 79,8872976 | 75,2395657 |
| 59,42206 | 80,7043192 | 72,7189634 | 71,1390637 | 79,9435712 | 75,2826035 |
| 59,50811 | 80,9985902 | 72,7593647 | 71,207479 | 79,9606014 | 75,2848255 |
| 59,54258 | 81,0135266 | 72,8115168 | 71,2286057 | 79,9916682 | 75,3194279 |
| 59,6148 | 81,0244647 | 72,8887182 | 71,2629466 | 80,0153973 | 75,4883035 |
| 59,64254 | 81,0415101 | 72,8909088 | 71,2982397 | 80,0349285 | 75,5549391 |
| 59,6858 | 81,1368309 | 72,8953649 | 71,3143051 | 80,0572785 | 75,5779534 |
| 59,71155 | 81,2013945 | 72,933711 | 71,3544393 | 80,0629999 | 75,595151 |
| 59,73196 | 81,2421 | 72,9878982 | 71,3937778 | 80,150526 | 75,6000441 |
| 59,74149 | 81,2631708 | 73,0013728 | 71,406624 | 80,1615402 | 75,7113178 |
| 59,74482 | 81,3937002 | 73,023836 | 71,5425326 | 80,1962455 | 75,834491 |
| 59,76825 | 81,5970309 | 73,0719998 | 71,570171 | 80,2260233 | 75,8558251 |
| 59,77726 | 81,8013019 | 73,1160204 | 71,7047463 | 80,3119873 | 75,9395806 |
| 59,82216 | 81,8105982 | 73,1841044 | 71,7418381 | 80,3290007 | 76,0719684 |
| 59,85958 | 81,8732122 | 73,2016466 | 71,7895318 | 80,340349 | 76,087025 |
| 60,0006 | 81,9222767 | 73,2862708 | 71,7929136 | 80,3443567 | 76,1910227 |
| 60,04212 | 81,9277975 | 73,3075071 | 71,8489452 | 80,409704 | 76,2832924 |
| 60,24028 | 82,1264116 | 73,3475207 | 72,0124077 | 80,4432523 | 76,3409311 |
| 60,2868 | 82,1546093 | 73,3593554 | 72,0285242 | 80,5969603 | 76,3778444 |
| 60,42493 | 82,1549755 | 73,3711432 | 72,0475023 | 80,637214 | 76,3901869 |
| 60,50308 | 82,3183981 | 73,4539526 | 72,0739321 | 80,6650339 | 76,4092819 |
| 60,61907 | 82,3196118 | 73,4810357 | 72,1459604 | 80,7290923 | 76,4371615 |
| 60,62901 | 82,4352683 | 73,5171379 | 72,1999178 | 80,7388495 | 76,4722288 |
| 60,80523 | 82,551356 | 73,6316609 | 72,2109354 | 80,7669364 | 76,4779796 |
| 60,81464 | 82,5995218 | 73,7064249 | 72,2151576 | 80,8127832 | 76,4835401 |
| 60,85442 | 82,8021579 | 73,7143448 | 72,215692 | 80,9284717 | 76,4951977 |
| 60,91893 | 82,8308602 | 73,7163358 | 72,3943961 | 80,9311168 | 76,5283708 |
| 60,92305 | 82,9520063 | 73,7318089 | 72,4316428 | 80,9744211 | 76,6099732 |
| 61,03596 | 83,0892262 | 73,7629832 | 72,4348188 | 80,9814885 | 76,7605366 |
| 61,13954 | 83,1495874 | 73,782829 | 72,4549679 | 81,0214526 | 76,8115503 |

| | | | | | |
|---|---|---|---|---|---|
| 61,55983 | 83,4095659 | 73,7879643 | 72,4579155 | 81,0270162 | 76,8711263 |
| 61,68102 | 83,5014249 | 73,8222637 | 72,5586922 | 81,0510301 | 76,8918073 |
| 61,84427 | 83,52591 | 73,8605799 | 72,6611895 | 81,0576122 | 77,0774113 |
| 62,02821 | 83,6847944 | 73,9029121 | 72,7198141 | 81,0724881 | 77,1812395 |
| 62,03541 | 83,8899222 | 73,9273665 | 72,7272783 | 81,1119285 | 77,2563247 |
| 62,04584 | 83,9455288 | 73,9777012 | 72,7799433 | 81,1131171 | 77,2567392 |
| 62,13949 | 84,1279744 | 74,0156095 | 72,7911388 | 81,1416876 | 77,3110854 |
| 62,42836 | 84,1442229 | 74,0500838 | 72,8587345 | 81,1590929 | 77,3910883 |
| 62,59767 | 84,3616398 | 74,1030526 | 72,8859117 | 81,1596028 | 77,3929155 |
| 62,67518 | 84,4493616 | 74,1040624 | 72,8866886 | 81,171043 | 77,4299079 |
| 62,84807 | 84,6809764 | 74,2588452 | 72,9206711 | 81,1882023 | 77,4683347 |
| 63,16647 | 84,9145077 | 74,2930804 | 72,9549242 | 81,1946765 | 77,5732509 |
| 63,35847 | 85,0438044 | 74,6334239 | 73,1403689 | 81,2403467 | 77,6013187 |
| 63,43954 | 85,4637777 | 74,6440055 | 73,1793622 | 81,3491221 | 77,6142767 |
| 63,48568 | 85,5440444 | 74,6617902 | 73,1948775 | 81,3499953 | 77,6575806 |
| 63,5381 | 85,6321184 | 74,713201 | 73,2822908 | 81,3607033 | 77,7490054 |
| 63,60242 | 85,8388064 | 74,762826 | 73,3007077 | 81,3992277 | 77,7490587 |
| 63,76921 | 85,995663 | 74,762904 | 73,3081413 | 81,4826216 | 77,7578572 |
| 63,80073 | 86,1400934 | 74,7812994 | 73,3250409 | 81,5017569 | 77,8347678 |
| 63,93171 | 86,2162367 | 74,8293591 | 73,3453064 | 81,5663664 | 77,8609215 |
| 63,95433 | 86,2734234 | 74,8851076 | 73,3499036 | 81,5707655 | 77,8653302 |
| 63,96934 | 86,4307657 | 74,890568 | 73,4010489 | 81,5711137 | 77,985241 |
| 64,09262 | 86,4473531 | 74,9908006 | 73,4127939 | 81,7300153 | 78,0646836 |
| 64,26341 | 86,5037552 | 74,9910706 | 73,445107 | 81,7598015 | 78,1093038 |
| 64,44805 | 86,6620655 | 75,0234237 | 73,4729079 | 81,7672413 | 78,1726272 |
| 64,66514 | 86,7691332 | 75,1049444 | 73,4795522 | 81,8318425 | 78,2025259 |
| 64,84264 | 86,8190683 | 75,1084777 | 73,4983197 | 81,8936023 | 78,2038849 |
| 64,85174 | 86,8444475 | 75,1418934 | 73,5632639 | 82,0988411 | 78,205655 |
| 64,88775 | 86,9115852 | 75,1487579 | 73,5687792 | 82,1032816 | 78,6039458 |
| 64,90968 | 86,9669555 | 75,1627417 | 73,5867965 | 82,1134839 | 78,6079969 |
| 64,93796 | 86,9919421 | 75,1781264 | 73,6022645 | 82,1167536 | 78,6326402 |
| 65,03639 | 87,0497683 | 75,1923228 | 73,6690871 | 82,1387433 | 78,653969 |
| 65,0403 | 87,0539241 | 75,2641289 | 73,6731832 | 82,1521208 | 78,6694903 |
| 65,09692 | 87,0983133 | 75,2917302 | 73,7306588 | 82,1526096 | 78,7158648 |
| 65,14592 | 87,1035008 | 75,3160394 | 73,854615 | 82,2044659 | 78,7554167 |
| 65,18089 | 87,1184267 | 75,3448015 | 73,8919612 | 82,2452496 | 78,8468458 |
| 65,3039 | 87,2252874 | 75,3659956 | 73,9056213 | 82,2587291 | 78,9217922 |
| 65,36177 | 87,2791685 | 75,4005086 | 74,0634172 | 82,2612139 | 78,9353613 |
| 65,552 | 87,6362533 | 75,4382804 | 74,1047953 | 82,2673291 | 78,9812671 |
| 65,58491 | 87,7613745 | 75,4922872 | 74,1573207 | 82,2762458 | 78,9883386 |
| 65,79875 | 87,8041941 | 75,5070991 | 74,169732 | 82,2856065 | 79,0584518 |
| 65,93016 | 87,8892894 | 75,5247126 | 74,1881576 | 82,2933386 | 79,0645184 |
| 65,99334 | 87,9389297 | 75,5939902 | 74,1959356 | 82,4351844 | 79,1332868 |
| 66,12099 | 88,0126255 | 75,5954933 | 74,2123509 | 82,5232734 | 79,1381225 |
| 66,16822 | 88,1152963 | 75,6536222 | 74,2266922 | 82,5280469 | 79,1453313 |
| 66,18058 | 88,124866 | 75,6842937 | 74,2874664 | 82,572015 | 79,242223 |
| 66,265 | 88,4314776 | 75,7791603 | 74,4611344 | 82,6025426 | 79,2655565 |

| | | | | | |
|---|---|---|---|---|---|
| 66,27564 | 88,4556604 | 75,841692 | 74,4994363 | 82,6126535 | 79,2706628 |
| 66,36134 | 88,676145 | 75,9291953 | 74,5405085 | 82,6208895 | 79,4189736 |
| 66,4511 | 88,8194388 | 75,9349453 | 74,5730636 | 82,6250817 | 79,4200651 |
| 66,48687 | 88,8888255 | 76,1151377 | 74,6217855 | 82,6267162 | 79,5231558 |
| 66,50122 | 88,9732002 | 76,1322403 | 74,689115 | 82,64024 | 79,792631 |
| 66,72685 | 89,074655 | 76,1743288 | 74,7019255 | 82,6599746 | 79,8581075 |
| 67,00447 | 89,2099611 | 76,1959993 | 74,7297909 | 82,696156 | 79,9597624 |
| 67,0412 | 89,2308294 | 76,2059561 | 74,74517 | 82,7004534 | 80,107461 |
| 67,09862 | 89,232153 | 76,2112294 | 74,7462775 | 82,8457157 | 80,1170626 |
| 67,19479 | 89,2585425 | 76,253699 | 74,768591 | 82,8468815 | 80,1789806 |
| 67,3769 | 89,2790895 | 76,3337371 | 74,8143275 | 82,8553974 | 80,2516628 |
| 67,3818 | 89,4069378 | 76,375324 | 74,8497748 | 82,871423 | 80,2851616 |
| 67,46817 | 89,5111914 | 76,3833944 | 74,8704545 | 82,9047758 | 80,3074967 |
| 67,60198 | 89,5532505 | 76,3838756 | 75,0533221 | 82,9248303 | 80,3084442 |
| 67,71829 | 89,572194 | 76,450408 | 75,1030118 | 83,0768787 | 80,4454869 |
| 67,80205 | 89,7856033 | 76,4665995 | 75,1599492 | 83,083731 | 80,7191893 |
| 67,8952 | 89,9345772 | 76,4835899 | 75,3117319 | 83,0853626 | 80,7222931 |
| 68,04465 | 89,979723 | 76,5024596 | 75,3971338 | 83,1023158 | 80,7756123 |
| 68,11159 | 90 | 76,5779355 | 75,5974751 | 83,1030498 | 80,8767547 |
| 68,3036 | 90 | 76,6799956 | 75,6610591 | 83,1807856 | 80,9803545 |
| 68,31822 | 90 | 76,6848522 | 75,7617791 | 83,2552558 | 81,0076883 |
| 68,31938 | | 76,7815261 | 75,8302903 | 83,2930063 | 81,0539666 |
| 68,64607 | | 76,785433 | 75,9047401 | 83,3060931 | 81,0984984 |
| 68,67975 | | 76,8903223 | 75,9745526 | 83,3377787 | 81,2313831 |
| 68,6986 | | 76,9633997 | 76,0145563 | 83,3404268 | 81,2538879 |
| 68,89309 | | 76,9774146 | 76,0263103 | 83,3791872 | 81,3821173 |
| 69,07532 | | 77,0234938 | 76,0308396 | 83,3827112 | 81,3923786 |
| 69,44217 | | 77,1016757 | 76,154344 | 83,3836635 | 81,4702218 |
| 69,56834 | | 77,1215964 | 76,1631516 | 83,4250874 | 81,4751892 |
| 69,75744 | | 77,1561704 | 76,1665639 | 83,4549076 | 81,4922351 |
| 69,80904 | | 77,1825528 | 76,216522 | 83,6135794 | 81,6538771 |
| 69,85657 | | 77,3268429 | 76,2277505 | 83,6684213 | 81,7628095 |
| 69,9788 | | 77,3380423 | 76,2698671 | 83,7270117 | 81,7830445 |
| 70,0737 | | 77,4141091 | 76,336773 | 83,7381405 | 81,7948611 |
| 70,1233 | | 77,4468116 | 76,5460732 | 83,7503942 | 81,8894703 |
| 70,15591 | | 77,7511621 | 76,5488325 | 83,7655017 | 81,9576833 |
| 70,18236 | | 77,9212189 | 76,5580787 | 83,8061858 | 81,9813939 |
| 70,29258 | | 77,9276105 | 76,5609669 | 83,8791317 | 81,9986985 |
| 70,31023 | | 77,9994775 | 76,6407626 | 83,8796003 | 82,0170894 |
| 70,39766 | | 78,0736567 | 76,7037791 | 83,8823201 | 82,0378723 |
| 70,49214 | | 78,1052553 | 76,7162335 | 83,9070418 | 82,110206 |
| 71,03697 | | 78,1567331 | 76,7282654 | 83,9156938 | 82,1665022 |
| 71,05236 | | 78,157502 | 76,8090576 | 83,9847145 | 82,1696501 |
| 71,68502 | | 78,1638079 | 76,8473607 | 83,9867304 | 82,2230646 |
| 71,71693 | | 78,1837465 | 76,867405 | 83,9961941 | 82,260847 |
| 71,85757 | | 78,3527807 | 76,8724226 | 84,0711253 | 82,3403422 |
| 71,9007 | | 78,387566 | 76,9388136 | 84,0858115 | 82,3775293 |

| | | | | |
|---|---|---|---|---|
| 71,97789 | 78,4133902 | 76,9832147 | 84,14083 | 82,4539829 |
| 71,9968 | 78,5098342 | 77,0083093 | 84,16314 | 82,4565657 |
| 72,00408 | 78,5300978 | 77,043622 | 84,1760403 | 82,4672118 |
| 72,05526 | 78,5360266 | 77,1059622 | 84,2139345 | 82,519123 |
| 72,21636 | 78,5680178 | 77,1866 | 84,2820611 | 82,5632121 |
| 72,29323 | 78,5722925 | 77,2528544 | 84,301474 | 82,6506337 |
| 72,30989 | 78,5748371 | 77,281988 | 84,3335526 | 82,661044 |
| 72,3399 | 78,6051521 | 77,2934367 | 84,3517466 | 82,6965769 |
| 72,40429 | 78,6576013 | 77,325797 | 84,4327317 | 82,7105595 |
| 72,44837 | 78,7100198 | 77,3980758 | 84,7053233 | 82,9380086 |
| 72,57095 | 78,9277796 | 77,5892528 | 84,7959022 | 83,0879653 |
| 72,69502 | 78,9983428 | 77,661797 | 84,7971215 | 83,1880297 |
| 72,84364 | 79,0771367 | 77,7998499 | 84,8387655 | 83,3290131 |
| 73,04932 | 79,1556539 | 77,9749246 | 84,8465391 | 83,3471934 |
| 73,29494 | 79,3021952 | 77,9903005 | 84,8844717 | 83,3732027 |
| 73,43417 | 79,4824352 | 78,0239568 | 84,8954658 | 83,3881937 |
| 73,44808 | 79,4873424 | 78,0337104 | 84,9124176 | 83,3953198 |
| 73,5485 | 79,4937006 | 78,1466816 | 84,9163738 | 83,4266587 |
| 73,54875 | 79,5756493 | 78,2016346 | 85,0350983 | 83,5305137 |
| 73,64121 | 79,5889115 | 78,3353879 | 85,035215 | 83,6024547 |
| 73,6578 | 79,6808477 | 78,4606232 | 85,0778964 | 83,6148998 |
| 73,67787 | 79,7831602 | 78,5048128 | 85,1110941 | 83,724396 |
| 73,71512 | 79,8644954 | 78,5645204 | 85,1945122 | 83,7428031 |
| 73,81352 | 79,8860713 | 78,5931579 | 85,1967968 | 83,7594924 |
| 74,05897 | 80,0034693 | 78,6392394 | 85,2952289 | 83,810625 |
| 74,33471 | 80,0213873 | 78,6941252 | 85,3444765 | 84,1194426 |
| 74,42281 | 80,1163662 | 78,7480376 | 85,3591893 | 84,1427756 |
| 74,57569 | 80,1235215 | 78,9520273 | 85,3768119 | 84,2392035 |
| 74,674 | 80,2021268 | 78,9896874 | 85,383918 | 84,2611789 |
| 74,70708 | 80,2381221 | 79,117767 | 85,3906516 | 84,2747638 |
| 74,70715 | 80,3081117 | 79,2501939 | 85,400263 | 84,370729 |
| 74,71523 | 80,3212348 | 79,250898 | 85,4040427 | 84,3932691 |
| 74,77645 | 80,3483825 | 79,3300942 | 85,4388111 | 84,4446147 |
| 74,85065 | 80,3984998 | 79,4593112 | 85,4951813 | 84,4778923 |
| 74,90861 | 80,4465399 | 79,4669958 | 85,5163052 | 84,5261524 |
| 74,94208 | 80,5162927 | 79,7807424 | 85,5178971 | 84,5781483 |
| 75,02576 | 80,5178802 | 79,8694225 | 85,6480484 | 84,5823406 |
| 75,03113 | 80,6320094 | 79,8892191 | 85,6601511 | 84,6714283 |
| 75,09047 | 80,6356398 | 79,9988305 | 85,6892105 | 84,77246 |
| 75,15403 | 80,6926743 | 80,1158509 | 85,7368907 | 84,8095654 |
| 75,21668 | 80,7311697 | 80,1315593 | 85,7595741 | 84,9262866 |
| 75,24885 | 80,8053896 | 80,1489844 | 85,7791655 | 84,9765336 |
| 75,66109 | 80,9657215 | 80,2068776 | 85,8283783 | 85,0624463 |
| 75,81666 | 81,0180192 | 80,3151178 | 85,8769608 | 85,0687468 |
| 75,88768 | 81,0663712 | 80,3531449 | 85,9605254 | 85,1000824 |
| 75,91886 | 81,0679343 | 80,3547876 | 86,035449 | 85,1357241 |
| 75,99311 | 81,1528726 | 80,3664057 | 86,0586454 | 85,2801114 |

| | | | | |
|---|---|---|---|---|
| 76,21197 | 81,367932 | 80,3941202 | 86,0840422 | 85,2833321 |
| 76,24865 | 81,4272406 | 80,510327 | 86,1044775 | 85,2877991 |
| 76,29515 | 81,4977921 | 80,5299626 | 86,1712267 | 85,3159132 |
| 76,3032 | 81,4981676 | 80,6634369 | 86,2266445 | 85,4309411 |
| 76,31895 | 81,5864111 | 80,6915089 | 86,3032828 | 85,501941 |
| 76,34815 | 81,6366545 | 80,7224493 | 86,3038455 | 85,5869234 |
| 76,49731 | 81,6663924 | 80,7514486 | 86,3346232 | 85,6020746 |
| 77,06178 | 81,7116783 | 80,8527719 | 86,3962862 | 85,6280106 |
| 77,25191 | 81,7296023 | 80,8596891 | 86,4459325 | 85,6438907 |
| 77,25287 | 81,8274599 | 81,0436264 | 86,5166492 | 85,7837353 |
| 77,3734 | 82,2262868 | 81,0699911 | 86,5425033 | 85,8793611 |
| 77,44539 | 82,426443 | 81,1069474 | 86,5504223 | 85,937185 |
| 77,49876 | 82,636555 | 81,1934713 | 86,5840164 | 85,9696798 |
| 77,58837 | 82,6406206 | 81,1978162 | 86,5854738 | 85,9932908 |
| 77,65876 | 82,7451748 | 81,214934 | 86,6734349 | 86,02589 |
| 77,74221 | 82,8169816 | 81,2841777 | 86,6996472 | 86,0609262 |
| 77,79851 | 82,8288642 | 81,3131046 | 86,720357 | 86,0791547 |
| 77,92002 | 82,8406678 | 81,4699293 | 86,7207561 | 86,1543477 |
| 77,98993 | 82,8614121 | 81,4707116 | 86,7211804 | 86,2097361 |
| 78,05034 | 83,0935254 | 81,4860685 | 86,756244 | 86,462369 |
| 78,07994 | 83,2085683 | 81,5189241 | 86,7692262 | 86,5131803 |
| 78,09698 | 83,4815374 | 81,6744689 | 86,7805999 | 86,5173248 |
| 78,30981 | 83,4862246 | 81,7974895 | 86,7860428 | 86,5535913 |
| 78,33871 | 83,5313447 | 81,9149843 | 86,876808 | 86,5882895 |
| 78,3628 | 83,6340634 | 81,9350937 | 86,8818156 | 86,8144914 |
| 78,48062 | 83,6565862 | 82,0422981 | 86,928396 | 86,9449751 |
| 78,50605 | 83,7254283 | 82,0565519 | 86,9562727 | 86,9801484 |
| 78,5403 | 83,7743835 | 82,0572834 | 87,1335948 | 87,0832066 |
| 78,55388 | 83,8865859 | 82,1633566 | 87,2226662 | 87,0896741 |
| 78,57256 | 83,8914964 | 82,2094396 | 87,2896694 | 87,1658809 |
| 78,57554 | 83,9090252 | 82,233821 | 87,2943104 | 87,2093607 |
| 78,64876 | 83,9302978 | 82,3035146 | 87,3212516 | 87,2298892 |
| 78,70134 | 83,9490093 | 82,3290948 | 87,3230347 | 87,2629824 |
| 78,81542 | 83,9643783 | 82,4374938 | 87,3653581 | 87,3511424 |
| 78,8567 | 83,9721799 | 82,4416677 | 87,3808052 | 87,3884382 |
| 78,98321 | 83,9819973 | 82,7461693 | 87,3829095 | 87,4218759 |
| 79,01116 | 84,0603017 | 82,7869557 | 87,4009403 | 87,4877999 |
| 79,13202 | 84,2477122 | 82,860168 | 87,4201705 | 87,4979043 |
| 79,15297 | 84,4404957 | 82,8977641 | 87,4419321 | 87,5611558 |
| 79,24153 | 84,4807732 | 82,9142586 | 87,4694583 | 87,6762697 |
| 79,45911 | 84,4856881 | 82,9959382 | 87,5038741 | 87,7877685 |
| 79,56755 | 84,6211079 | 83,0096222 | 87,7953224 | 87,8691196 |
| 79,58976 | 84,6477705 | 83,0118542 | 87,8651536 | 87,9803191 |
| 79,74257 | 84,7290244 | 83,0222578 | 87,8810261 | 87,995707 |
| 80,00148 | 84,7984236 | 83,0653343 | 87,9352587 | 88,0598414 |
| 80,04104 | 84,8416402 | 83,0805507 | 87,979513 | 88,0739163 |
| 80,22244 | 84,851976 | 83,0845575 | 87,9966295 | 88,0810429 |

| | | | | |
|---|---|---|---|---|
| 80,32016 | 84,875696 | 83,5729659 | 88,0652018 | 88,0824867 |
| 80,38515 | 85,2129621 | 84,3533297 | 88,0800136 | 88,0850267 |
| 80,46827 | 85,3086944 | 84,3962546 | 88,1822709 | 88,0929181 |
| 80,4807 | 85,3284848 | 84,5368515 | 88,1920932 | 88,1073703 |
| 80,8453 | 85,3689264 | 84,5389742 | 88,2439441 | 88,122211 |
| 81,11422 | 85,4916556 | 84,6291157 | 88,2584288 | 88,1940958 |
| 81,23024 | 85,5119689 | 84,694988 | 88,2667807 | 88,2457801 |
| 81,23718 | 85,6115444 | 84,7102819 | 88,3808657 | 88,2467593 |
| 81,41133 | 85,6295073 | 84,7432595 | 88,3909099 | 88,2935859 |
| 81,47455 | 85,6413973 | 84,7662862 | 88,4841784 | 88,3705387 |
| 81,54833 | 85,7024807 | 84,8150869 | 88,4950112 | 88,5445073 |
| 81,68242 | 85,7051175 | 84,956284 | 88,5182245 | 88,5591808 |
| 82,0141 | 85,7269666 | 85,0073612 | 88,5426518 | 88,6463642 |
| 82,12982 | 85,7343012 | 85,1412427 | 88,5909872 | 88,6862712 |
| 82,30477 | 85,7732721 | 85,2025219 | 88,6125218 | 88,769087 |
| 82,37842 | 85,8456196 | 85,2290759 | 88,6144504 | 88,7765323 |
| 82,49098 | 86,0012051 | 85,2586162 | 88,6373519 | 88,8261269 |
| 82,61398 | 86,0454127 | 85,2798904 | 88,6490701 | 88,8425677 |
| 82,61746 | 86,0668683 | 85,4182828 | 88,6743426 | 88,88706 |
| 82,6572 | 86,2556423 | 85,46521 | 88,6760842 | 88,9764037 |
| 82,68339 | 86,3061844 | 85,4862241 | 88,706947 | 88,9945155 |
| 82,91707 | 86,341725 | 85,5522418 | 88,7358114 | 89,0006573 |
| 83,08563 | 86,3955057 | 85,5546647 | 88,7960708 | 89,0511356 |
| 83,31407 | 86,404222 | 85,5603474 | 88,8036549 | 89,141497 |
| 83,35018 | 86,4787381 | 85,6181965 | 88,8121581 | 89,1689866 |
| 83,43457 | 86,4993214 | 85,6271229 | 88,8950921 | 89,1877993 |
| 83,55307 | 86,5779446 | 85,6293995 | 88,9334007 | 89,3013997 |
| 83,67479 | 86,5781672 | 85,6832735 | 88,9510014 | 89,4009664 |
| 83,689 | 86,5897365 | 85,7427843 | 88,9805532 | 89,4917678 |
| 83,6955 | 86,7725869 | 85,8234494 | 89,0044898 | 89,5659941 |
| 83,73033 | 86,7862598 | 85,8244339 | 89,0239042 | 89,5748704 |
| 83,81302 | 86,8061968 | 85,9197207 | 89,0393731 | 89,5969886 |
| 83,8327 | 86,8139389 | 85,9609357 | 89,0423537 | 89,6231019 |
| 84,18968 | 86,8468692 | 86,2762389 | 89,0428363 | 89,6398779 |
| 84,24316 | 86,8517091 | 86,3411504 | 89,112288 | 89,6638491 |
| 84,55612 | 86,88554 | 86,3684184 | 89,1384877 | 89,7518503 |
| 84,59655 | 86,9109703 | 86,4765471 | 89,1569937 | 89,7872283 |
| 84,66227 | 87,1262648 | 86,5048387 | 89,160792 | 89,8346437 |
| 84,90316 | 87,3360113 | 86,5682326 | 89,1865762 | 89,8502447 |
| 85,1169 | 87,3853634 | 86,6024393 | 89,2550464 | 89,9008212 |
| 85,23994 | 87,5455608 | 86,6766064 | 89,2711209 | 89,9841764 |
| 85,41833 | 87,5522627 | 86,8331571 | 89,2954121 | 89,9874361 |
| 85,4523 | 87,5949834 | 86,902515 | 89,3266519 | 89,990727 |
| 85,56732 | 87,6143201 | 86,9138395 | 89,3521919 | 90 |
| 85,68661 | 87,775084 | 87,1484884 | 89,3592354 | 90 |
| 85,73922 | 87,7804395 | 87,1830339 | 89,3719181 | 90 |
| 85,75739 | 87,8262982 | 87,2595492 | 89,3816699 | 90 |

| | | | | |
|---|---|---|---|---|
| 85,78085 | 87,9355582 | 87,4015267 | 89,4322314 | 90 |
| 85,81752 | 87,9546838 | 87,537131 | 89,4856799 | 90 |
| 85,85374 | 88,0890559 | 87,5962785 | 89,4857298 | 90 |
| 86,14546 | 88,1919177 | 87,6082959 | 89,5044648 | 90 |
| 86,16207 | 88,2304865 | 87,6295667 | 89,5122266 | 90 |
| 86,39162 | 88,3853038 | 87,8124581 | 89,5904792 | 90 |
| 86,44003 | 88,4923879 | 87,8680403 | 89,6614466 | 90 |
| 86,48849 | 88,512657 | 87,8926627 | 89,7052239 | 90 |
| 86,84273 | 88,5264445 | 87,919773 | 89,7362798 | |
| 86,89462 | 88,5802639 | 87,969908 | 89,7695929 | |
| 86,90553 | 88,6620523 | 88,0582078 | 89,780806 | |
| 87,04981 | 88,6764943 | 88,1697177 | 89,7925449 | |
| 87,07644 | 88,7492775 | 88,1760368 | 89,8006844 | |
| 87,14702 | 88,8253517 | 88,1810591 | 89,8010808 | |
| 87,19979 | 89,0147838 | 88,3136206 | 89,8038455 | |
| 87,29208 | 89,1227198 | 88,3541587 | 89,8169975 | |
| 87,30325 | 89,1417952 | 88,392193 | 89,9406753 | |
| 87,34366 | 89,1841583 | 88,4334301 | 89,9534657 | |
| 87,34536 | 89,1960912 | 88,4758702 | 90 | |
| 87,39789 | 89,2283196 | 88,4800726 | 90 | |
| 87,48708 | 89,2506932 | 88,5100349 | 90 | |
| 87,54399 | 89,319361 | 88,5121753 | 90 | |
| 87,81932 | 89,3904878 | 88,5424099 | 90 | |
| 87,84431 | 89,4223187 | 88,689928 | 90 | |
| 87,85976 | 89,4795339 | 88,7066343 | 90 | |
| 88,06629 | 89,6045514 | 88,7349821 | 90 | |
| 88,14313 | 89,6636969 | 88,7823633 | 90 | |
| 88,29043 | 89,7221242 | 88,8458757 | 90 | |
| 88,36104 | 89,7534157 | 88,8609542 | 90 | |
| 88,56367 | 89,7953532 | 88,8677164 | 90 | |
| 88,66108 | 89,948532 | 88,8738528 | 90 | |
| 88,6636 | 90 | 88,9729562 | 90 | |
| 88,86819 | 90 | 88,9755022 | 90 | |
| 88,99484 | 90 | 89,132077 | 90 | |
| 89,07771 | 90 | 89,3485036 | 90 | |
| 89,24861 | 90 | 89,394657 | 90 | |
| 89,32261 | 90 | 89,4357119 | 90 | |
| 89,39502 | 90 | 89,5905382 | 90 | |
| 89,4987 | | 89,7624392 | | |
| 89,69934 | | 89,777904 | | |
| 89,71043 | | 89,9142768 | | |
| 89,73335 | | 89,9962884 | | |
| 89,73574 | | 90 | | |
| 89,76978 | | 90 | | |
| 89,80871 | | 90 | | |
| 89,86611 | | 90 | | |
| 89,94251 | | 90 | | |

| | |
|---|---|
| 89,99448 | 90 |
| 90 | 90 |
| 90 | 90 |
| 90 | 90 |
| 90 | 90 |
| 90 | 90 |

| 4 h str.- (d) | 4 h str. +CQ (h) |
|---|---|
| 61,3728815 | 0,51359123 |
| 61,9897484 | 1,94667983 |
| 65,6023898 | 2,41628337 |
| 66,2850979 | 2,85528352 |
| 66,9125524 | 3,22074913 |
| 66,9265867 | 3,51147759 |
| 67,4831103 | 4,55719961 |
| 67,5316416 | 4,66359147 |
| 67,6388457 | 5,11535317 |
| 67,6526496 | 8,19891335 |
| 67,7287061 | 8,35993541 |
| 68,0721741 | 10,423797 |
| 69,3724964 | 11,1307317 |
| 69,4638023 | 11,1416847 |
| 69,5599335 | 11,1936187 |
| 69,7943066 | 11,5196742 |
| 69,8982798 | 12,7683372 |
| 70,0240172 | 13,6848753 |
| 70,0895239 | 17,3989145 |
| 70,3097131 | 18,0374521 |
| 70,9425886 | 18,276986 |
| 71,0263128 | 18,8052397 |
| 71,3221695 | 21,7211359 |
| 71,6738944 | 23,6956898 |
| 71,7120791 | 26,5579558 |
| 71,7538444 | 29,8024937 |
| 71,7843794 | 30,4064062 |
| 71,8488231 | 31,1885155 |
| 72,0159669 | 31,3192381 |
| 72,143018 | 31,8589414 |
| 72,148059 | 35,0613483 |
| 72,1921981 | 38,5404105 |
| 72,305879 | 38,9257689 |
| 72,3984565 | 38,9671658 |
| 72,4048218 | 39,1506533 |
| 72,4096185 | 40,2832288 |
| 72,5968314 | 40,3488701 |
| 72,7676054 | 40,4954796 |
| 72,7764626 | 41,2485345 |
| 72,781141 | 41,2900003 |
| 72,7860551 | 42,0548718 |
| 72,8814041 | 43,2838252 |
| 72,9381824 | 44,1888536 |
| 72,9391582 | 44,828573 |

| | |
|---|---|
| 72,942734 | 45,0166012 |
| 72,9944054 | 45,3559734 |
| 73,0761189 | 45,5179248 |
| 73,271449 | 46,5954312 |
| 73,4266265 | 46,6528517 |
| 73,5173512 | 46,7599414 |
| 73,529634 | 47,0414158 |
| 73,5673881 | 47,5803689 |
| 73,6442275 | 47,7633586 |
| 73,6736968 | 47,830146 |
| 73,6834793 | 48,1692276 |
| 73,686798 | 48,4968181 |
| 73,7411287 | 48,6898518 |
| 73,7779119 | 48,9810023 |
| 73,8017604 | 49,0788881 |
| 73,8640327 | 49,1427267 |
| 73,8857664 | 49,2306667 |
| 73,9631365 | 49,2953232 |
| 74,058378 | 49,7409142 |
| 74,1030092 | 49,9441496 |
| 74,1987709 | 50,9462507 |
| 74,2210465 | 51,1811341 |
| 74,2792621 | 51,2264128 |
| 74,2862138 | 51,2297499 |
| 74,3154724 | 51,3696911 |
| 74,3216182 | 51,3768739 |
| 74,3589496 | 51,4954099 |
| 74,373536 | 51,4990957 |
| 74,3956496 | 51,7941666 |
| 74,4589081 | 51,8483405 |
| 74,4666043 | 51,9492325 |
| 74,4757928 | 51,9760512 |
| 74,6504618 | 51,9778809 |
| 74,6679385 | 52,1270849 |
| 74,8612052 | 52,3452142 |
| 75,0406077 | 52,7108194 |
| 75,0812386 | 52,9154758 |
| 75,0946459 | 52,9157613 |
| 75,1460665 | 53,230548 |
| 75,2197544 | 53,3666595 |
| 75,2728807 | 53,8497746 |
| 75,3677714 | 54,3304509 |
| 75,3894845 | 54,4992173 |
| 75,4465505 | 54,7892315 |
| 75,4655724 | 55,0132924 |
| 75,5125664 | 55,0217624 |
| 75,5477646 | 55,2311497 |

| | |
|---|---|
| 75,5501009 | 55,3056698 |
| 75,5767292 | 55,7930876 |
| 75,5882402 | 55,9167112 |
| 75,6513785 | 55,9543597 |
| 75,6614064 | 55,987717 |
| 75,7417058 | 56,2778115 |
| 75,8234203 | 56,6507285 |
| 75,8273991 | 56,7919213 |
| 75,8534603 | 56,7952418 |
| 75,87439 | 57,0436778 |
| 75,8757995 | 57,1696657 |
| 75,8825131 | 57,2044976 |
| 75,8895157 | 57,3994656 |
| 75,937894 | 58,065292 |
| 75,9755087 | 58,3019983 |
| 75,9829014 | 58,4322179 |
| 76,0684004 | 58,5933539 |
| 76,0853624 | 58,6716832 |
| 76,1422669 | 58,6971691 |
| 76,1470046 | 58,7055117 |
| 76,411398 | 59,0140018 |
| 76,5159487 | 59,1937663 |
| 76,5569262 | 59,2068635 |
| 76,5921221 | 59,5157969 |
| 76,6103006 | 59,5798988 |
| 76,6263159 | 59,7284792 |
| 76,6746425 | 59,7776872 |
| 76,7156332 | 60,0111059 |
| 76,8506674 | 60,2153379 |
| 76,8615881 | 60,2331492 |
| 76,878981 | 60,4814631 |
| 76,8836199 | 60,7637285 |
| 76,9120393 | 60,8875494 |
| 76,9231794 | 60,9221227 |
| 76,9367938 | 61,2330807 |
| 76,9568807 | 61,5319851 |
| 76,9840177 | 61,6391756 |
| 77,0784754 | 61,7081572 |
| 77,0896522 | 61,7815879 |
| 77,0910933 | 61,9162388 |
| 77,1292173 | 62,0122348 |
| 77,185467 | 62,1608265 |
| 77,2003948 | 62,1843846 |
| 77,2099932 | 62,507046 |
| 77,2512678 | 62,9393785 |
| 77,2515522 | 63,5839752 |
| 77,3146561 | 63,6958379 |

| | |
|---|---|
| 77,3290355 | 63,8082194 |
| 77,4085541 | 63,8559 |
| 77,5274497 | 64,0277417 |
| 77,5776296 | 64,2208114 |
| 77,5876147 | 64,2362293 |
| 77,647102 | 64,241243 |
| 77,6695517 | 64,4621352 |
| 77,711905 | 64,670408 |
| 77,7766718 | 64,9270028 |
| 77,8477994 | 64,9293289 |
| 77,8668044 | 65,0141455 |
| 77,8933531 | 65,2719809 |
| 77,907283 | 65,437074 |
| 77,9541605 | 65,6511447 |
| 77,9722256 | 65,6610503 |
| 77,9851532 | 65,6715135 |
| 77,9911045 | 65,7049002 |
| 78,0473664 | 65,7733223 |
| 78,1368258 | 65,9442761 |
| 78,1459973 | 66,0754325 |
| 78,1637633 | 66,2928644 |
| 78,1941467 | 66,9017804 |
| 78,1967885 | 67,0159777 |
| 78,2072348 | 67,0497692 |
| 78,2092187 | 67,1158712 |
| 78,2097936 | 67,172794 |
| 78,2226642 | 67,1781847 |
| 78,2349316 | 67,4074063 |
| 78,2694738 | 67,4261551 |
| 78,3148153 | 67,4970282 |
| 78,3437141 | 67,5550357 |
| 78,3628339 | 67,6390896 |
| 78,3911258 | 67,7440146 |
| 78,4047771 | 67,7994938 |
| 78,4107643 | 67,8888778 |
| 78,5311762 | 68,2271155 |
| 78,549349 | 68,3340321 |
| 78,639193 | 68,3742899 |
| 78,6637801 | 68,4811065 |
| 78,6729575 | 68,5912075 |
| 78,6879506 | 68,7118525 |
| 78,7200145 | 68,7286896 |
| 78,7218082 | 68,8001674 |
| 78,7386705 | 68,8525138 |
| 78,7503845 | 69,146759 |
| 78,7541918 | 69,1614057 |
| 78,7560118 | 69,1706895 |

| | |
|---|---|
| 78,7811135 | 69,1781458 |
| 78,8041828 | 69,291796 |
| 78,8352734 | 69,4678948 |
| 78,8818378 | 69,477824 |
| 78,8904646 | 69,5028256 |
| 78,9545987 | 69,5262446 |
| 78,9768331 | 69,6299924 |
| 78,9826764 | 69,824213 |
| 79,0113246 | 70,0937822 |
| 79,0572375 | 70,2756856 |
| 79,0574893 | 70,291645 |
| 79,067811 | 70,3957052 |
| 79,1598916 | 70,4205248 |
| 79,1637772 | 70,4641677 |
| 79,1697963 | 70,5303831 |
| 79,1799096 | 70,5461894 |
| 79,1916175 | 70,5724086 |
| 79,2347597 | 70,6971362 |
| 79,2420218 | 70,9690777 |
| 79,2527491 | 71,0936242 |
| 79,2575308 | 71,1722387 |
| 79,2763462 | 71,2071825 |
| 79,3214093 | 71,3187755 |
| 79,3601577 | 71,3194161 |
| 79,3712823 | 71,3653086 |
| 79,4284599 | 71,4510824 |
| 79,4603987 | 71,4565076 |
| 79,4830771 | 71,482634 |
| 79,5143388 | 71,55234 |
| 79,5231122 | 71,7111265 |
| 79,5667066 | 71,7371683 |
| 79,6013942 | 71,7840538 |
| 79,6181595 | 72,1796565 |
| 79,6531391 | 72,1905923 |
| 79,6578512 | 72,2136119 |
| 79,6818083 | 72,4435129 |
| 79,6961293 | 72,520002 |
| 79,7322971 | 72,5329803 |
| 79,7520643 | 72,5349179 |
| 79,7654287 | 72,5361491 |
| 79,8097186 | 72,5815133 |
| 79,8696571 | 72,6218716 |
| 79,8820287 | 72,6711783 |
| 79,940238 | 72,6927748 |
| 80,0098242 | 72,8397692 |
| 80,0279626 | 72,8706733 |
| 80,0583603 | 72,8822683 |

| | |
|---|---|
| 80,1113728 | 72,9194619 |
| 80,1173462 | 72,9498732 |
| 80,1331393 | 73,0145191 |
| 80,1529813 | 73,0272821 |
| 80,189321 | 73,2364421 |
| 80,2106774 | 73,3026289 |
| 80,2743668 | 73,3464036 |
| 80,3140109 | 73,4195941 |
| 80,3416086 | 73,4284924 |
| 80,3668709 | 73,5449955 |
| 80,4148872 | 73,5824793 |
| 80,4333502 | 73,5970599 |
| 80,4955866 | 73,8095214 |
| 80,5327601 | 73,8205421 |
| 80,556628 | 73,8511052 |
| 80,5787353 | 73,9683987 |
| 80,6005176 | 74,0124604 |
| 80,6260749 | 74,0366907 |
| 80,6311498 | 74,0944664 |
| 80,64029 | 74,1294105 |
| 80,7027139 | 74,2523672 |
| 80,7098122 | 74,2761097 |
| 80,7178212 | 74,301221 |
| 80,7529156 | 74,3214448 |
| 80,7680956 | 74,3441353 |
| 80,7775097 | 74,4590083 |
| 80,7924401 | 74,5870875 |
| 80,8356768 | 74,7089255 |
| 80,8850029 | 74,8386218 |
| 80,9135602 | 74,840938 |
| 80,9487263 | 74,867693 |
| 80,9718262 | 74,9438958 |
| 80,9837053 | 74,9971471 |
| 81,0213435 | 75,0575269 |
| 81,0334695 | 75,0601167 |
| 81,0339481 | 75,0897426 |
| 81,0403859 | 75,1557756 |
| 81,0522281 | 75,1985132 |
| 81,070894 | 75,2104666 |
| 81,0908222 | 75,2720453 |
| 81,1622502 | 75,3390651 |
| 81,1694607 | 75,3689981 |
| 81,1982605 | 75,3698586 |
| 81,2192082 | 75,3926685 |
| 81,2664718 | 75,4414154 |
| 81,2664794 | 75,4460935 |
| 81,2757996 | 75,4695128 |

| | |
|---|---|
| 81,2872525 | 75,483807 |
| 81,2949098 | 75,6716074 |
| 81,3422848 | 75,7058388 |
| 81,4393561 | 75,7543749 |
| 81,4826752 | 75,7707709 |
| 81,5523089 | 75,8436887 |
| 81,565631 | 75,8616784 |
| 81,5661408 | 75,947149 |
| 81,5698607 | 75,9479244 |
| 81,6639748 | 75,9517613 |
| 81,7276066 | 76,0711037 |
| 81,7798582 | 76,099178 |
| 81,7919742 | 76,2281503 |
| 81,7983783 | 76,2743781 |
| 81,8663557 | 76,3980923 |
| 81,895355 | 76,4889256 |
| 81,8999438 | 76,4911108 |
| 81,913855 | 76,493849 |
| 81,9441988 | 76,5004592 |
| 81,9470191 | 76,5410394 |
| 81,9829005 | 76,6123302 |
| 81,9858495 | 76,7203125 |
| 81,9915554 | 76,8059941 |
| 82,0032732 | 76,8101447 |
| 82,0207384 | 76,8206729 |
| 82,0373936 | 76,8472908 |
| 82,1223371 | 76,8878215 |
| 82,1374753 | 76,9133711 |
| 82,1471203 | 76,954256 |
| 82,1758188 | 77,0349771 |
| 82,221135 | 77,1014541 |
| 82,2911962 | 77,135317 |
| 82,3038132 | 77,1533242 |
| 82,3079285 | 77,2001071 |
| 82,3185943 | 77,2385476 |
| 82,3686408 | 77,2948973 |
| 82,3769152 | 77,35877 |
| 82,3895146 | 77,4199578 |
| 82,399862 | 77,6026218 |
| 82,4080151 | 77,6141166 |
| 82,4152834 | 77,629636 |
| 82,4746754 | 77,6372216 |
| 82,5029801 | 77,6876834 |
| 82,5032276 | 77,7212876 |
| 82,5161703 | 77,7417675 |
| 82,5491881 | 77,7490247 |
| 82,5494373 | 77,7781797 |

| | |
|---|---|
| 82,5634286 | 77,78831 |
| 82,5769657 | 77,7995216 |
| 82,6172206 | 77,8591919 |
| 82,623239 | 77,8978444 |
| 82,6274764 | 77,9138845 |
| 82,6337072 | 78,0356177 |
| 82,6607657 | 78,043085 |
| 82,7354283 | 78,0439254 |
| 82,7379232 | 78,0990197 |
| 82,7438213 | 78,1036295 |
| 82,7695231 | 78,1386978 |
| 82,8455173 | 78,1665889 |
| 82,8471632 | 78,1727565 |
| 82,9005282 | 78,2248517 |
| 82,9033026 | 78,2316765 |
| 82,9059594 | 78,3071146 |
| 82,9081756 | 78,3768963 |
| 82,9247374 | 78,3781155 |
| 82,936082 | 78,6478693 |
| 82,9829606 | 78,6903907 |
| 82,9960957 | 78,753836 |
| 82,9982028 | 78,7764236 |
| 83,0284781 | 78,7852421 |
| 83,0445514 | 78,8186857 |
| 83,0637101 | 78,8664496 |
| 83,0691305 | 78,888057 |
| 83,1100922 | 78,927755 |
| 83,1123918 | 79,0108084 |
| 83,2006169 | 79,0185235 |
| 83,2014578 | 79,0272111 |
| 83,2183832 | 79,0328543 |
| 83,249263 | 79,0351189 |
| 83,2794723 | 79,0528244 |
| 83,307844 | 79,1183052 |
| 83,323048 | 79,1573734 |
| 83,3623078 | 79,1842869 |
| 83,3643232 | 79,2042277 |
| 83,3979528 | 79,4154329 |
| 83,4272106 | 79,4286881 |
| 83,435416 | 79,59965 |
| 83,4964809 | 79,7939756 |
| 83,5489214 | 79,7981486 |
| 83,54914 | 79,8068588 |
| 83,5501735 | 79,9162452 |
| 83,6069916 | 79,928976 |
| 83,6186588 | 79,9469538 |
| 83,6241684 | 79,9891095 |

| | |
|---|---|
| 83,6438478 | 80,0438142 |
| 83,6694168 | 80,0715544 |
| 83,6763246 | 80,0782711 |
| 83,6879982 | 80,1161922 |
| 83,6908728 | 80,1663638 |
| 83,7842186 | 80,1989459 |
| 83,8145511 | 80,202466 |
| 83,8766499 | 80,3251303 |
| 83,9075176 | 80,379932 |
| 83,9137266 | 80,4159476 |
| 83,9296758 | 80,6297069 |
| 83,9311469 | 80,6572373 |
| 83,938929 | 80,699745 |
| 83,9735255 | 80,7625989 |
| 83,9928091 | 80,8140685 |
| 84,0559702 | 80,8939145 |
| 84,0737119 | 80,9089987 |
| 84,0939434 | 80,9296917 |
| 84,0979443 | 80,9953085 |
| 84,1163327 | 81,0015639 |
| 84,1271061 | 81,0134638 |
| 84,1515164 | 81,1357129 |
| 84,1624367 | 81,2200357 |
| 84,1645399 | 81,2323906 |
| 84,1685212 | 81,2500635 |
| 84,1727949 | 81,2512397 |
| 84,1891622 | 81,2688083 |
| 84,22079 | 81,2695229 |
| 84,2233861 | 81,365623 |
| 84,2301696 | 81,4573048 |
| 84,2464265 | 81,5249551 |
| 84,2638697 | 81,5288623 |
| 84,2979235 | 81,5289633 |
| 84,3189439 | 81,5405456 |
| 84,3220639 | 81,5727294 |
| 84,3297405 | 81,5808379 |
| 84,3413534 | 81,6235096 |
| 84,3569677 | 81,6702558 |
| 84,3639029 | 81,7101664 |
| 84,3749833 | 81,7338296 |
| 84,3864922 | 81,7803222 |
| 84,3929961 | 81,7892336 |
| 84,4074071 | 81,8024703 |
| 84,4442442 | 81,8117648 |
| 84,4537058 | 81,8455203 |
| 84,5189109 | 81,8469639 |
| 84,5277492 | 81,8540409 |

| | |
|---|---|
| 84,5761456 | 81,9911958 |
| 84,6038535 | 82,0080328 |
| 84,6049299 | 82,0284122 |
| 84,607859 | 82,1890123 |
| 84,6154998 | 82,2017429 |
| 84,6439579 | 82,3745671 |
| 84,6758028 | 82,3924212 |
| 84,6787465 | 82,4450109 |
| 84,7144237 | 82,4584506 |
| 84,7291522 | 82,5352791 |
| 84,7391476 | 82,5490354 |
| 84,7429997 | 82,5999411 |
| 84,7627142 | 82,6523223 |
| 84,8462028 | 82,7039988 |
| 84,8581732 | 82,71007 |
| 84,8765361 | 82,7488861 |
| 84,9036902 | 82,7659409 |
| 84,9202278 | 82,8187733 |
| 84,9229526 | 82,8276708 |
| 84,926606 | 82,8639118 |
| 84,9700305 | 82,8682288 |
| 84,9726281 | 82,8691989 |
| 84,9909472 | 82,8831761 |
| 84,9931609 | 82,9431375 |
| 85,0185805 | 82,979654 |
| 85,0211188 | 82,9971462 |
| 85,0269966 | 83,0462304 |
| 85,036278 | 83,0779394 |
| 85,037934 | 83,0938373 |
| 85,0441799 | 83,112374 |
| 85,0539757 | 83,1205616 |
| 85,0875317 | 83,126183 |
| 85,0984712 | 83,2111365 |
| 85,1303668 | 83,2917346 |
| 85,136463 | 83,3975138 |
| 85,1513305 | 83,4691284 |
| 85,1537473 | 83,5027958 |
| 85,1925597 | 83,5159479 |
| 85,1997983 | 83,5418254 |
| 85,2103067 | 83,6732194 |
| 85,2230181 | 83,7416223 |
| 85,2427091 | 83,7886732 |
| 85,279751 | 83,8647051 |
| 85,2858662 | 83,901717 |
| 85,2975531 | 83,9399137 |
| 85,3268435 | 83,9665513 |
| 85,330803 | 84,0543163 |

| | |
|---|---|
| 85,3775715 | 84,0717565 |
| 85,3790745 | 84,0811148 |
| 85,3877128 | 84,125374 |
| 85,3899151 | 84,241177 |
| 85,3960916 | 84,2609636 |
| 85,4525814 | 84,2667831 |
| 85,4900386 | 84,3139715 |
| 85,4908636 | 84,3202105 |
| 85,5040398 | 84,3900194 |
| 85,5247141 | 84,4020168 |
| 85,5429926 | 84,402672 |
| 85,5562801 | 84,4036443 |
| 85,5675048 | 84,4262406 |
| 85,6055731 | 84,4547481 |
| 85,6293015 | 84,4598973 |
| 85,6307037 | 84,4909671 |
| 85,6353268 | 84,5061579 |
| 85,6740861 | 84,5292375 |
| 85,7012525 | 84,5952777 |
| 85,7483739 | 84,6287276 |
| 85,7492231 | 84,7039877 |
| 85,7642277 | 84,7041998 |
| 85,7659455 | 84,7051994 |
| 85,7960519 | 84,7190371 |
| 85,8015541 | 84,7434454 |
| 85,8132059 | 84,756408 |
| 85,826961 | 84,7676556 |
| 85,8531933 | 84,8448455 |
| 85,8681831 | 84,850007 |
| 85,8686003 | 84,8660453 |
| 85,8718952 | 84,8856386 |
| 85,8831811 | 84,9993477 |
| 85,8979871 | 85,0236208 |
| 85,9064191 | 85,0251656 |
| 85,9163511 | 85,0296561 |
| 85,9396081 | 85,0597074 |
| 85,9408934 | 85,1140482 |
| 85,9617171 | 85,1151801 |
| 86,0053418 | 85,173666 |
| 86,0667616 | 85,1895075 |
| 86,068088 | 85,224684 |
| 86,0716524 | 85,2278312 |
| 86,0808037 | 85,2340026 |
| 86,1052789 | 85,2725246 |
| 86,1141142 | 85,3566187 |
| 86,2400867 | 85,3639047 |
| 86,2463969 | 85,3905519 |

| | |
|---|---|
| 86,2486712 | 85,4225387 |
| 86,2500476 | 85,4434211 |
| 86,3178287 | 85,4509361 |
| 86,3243544 | 85,5010055 |
| 86,4097139 | 85,5312147 |
| 86,4112744 | 85,5418695 |
| 86,4570732 | 85,5511799 |
| 86,5009372 | 85,5821013 |
| 86,5488024 | 85,611872 |
| 86,5641307 | 85,6654975 |
| 86,5840604 | 85,6898384 |
| 86,599915 | 85,7002434 |
| 86,6023425 | 85,7033384 |
| 86,61199 | 85,703859 |
| 86,6185536 | 85,7556227 |
| 86,6258159 | 85,7768095 |
| 86,6306199 | 85,7794312 |
| 86,6531545 | 85,8439959 |
| 86,6550334 | 85,8762924 |
| 86,689708 | 85,9004675 |
| 86,6904737 | 85,9216177 |
| 86,6948423 | 85,9347459 |
| 86,7140883 | 85,9444356 |
| 86,717063 | 85,9647714 |
| 86,8276109 | 85,9728435 |
| 86,8395467 | 85,9797719 |
| 86,8675641 | 85,9990864 |
| 86,8708681 | 86,0376219 |
| 86,9167903 | 86,0463867 |
| 86,9432934 | 86,0511092 |
| 86,9842285 | 86,0682372 |
| 86,9865376 | 86,0832933 |
| 86,9925026 | 86,1149747 |
| 87,0059696 | 86,1172001 |
| 87,0354925 | 86,1254371 |
| 87,0936273 | 86,1323211 |
| 87,1274289 | 86,1565712 |
| 87,1356008 | 86,1890211 |
| 87,1375979 | 86,2123825 |
| 87,1653326 | 86,2952919 |
| 87,1782903 | 86,3589684 |
| 87,1873637 | 86,4719639 |
| 87,1984941 | 86,4728399 |
| 87,231745 | 86,4821734 |
| 87,2349104 | 86,579207 |
| 87,255126 | 86,5821453 |
| 87,2651401 | 86,5981733 |

| | |
|---|---|
| 87,2824766 | 86,5996443 |
| 87,2899379 | 86,6615792 |
| 87,2918163 | 86,6792915 |
| 87,325393 | 86,6806403 |
| 87,3429578 | 86,6831373 |
| 87,3842191 | 86,7036848 |
| 87,390382 | 86,7348855 |
| 87,3904416 | 86,779154 |
| 87,3960993 | 86,7867032 |
| 87,4097864 | 86,8743054 |
| 87,4194307 | 86,8938311 |
| 87,4577012 | 86,9333124 |
| 87,462005 | 86,9389077 |
| 87,46471 | 86,9717255 |
| 87,4895201 | 87,0499927 |
| 87,5032452 | 87,0637013 |
| 87,5529772 | 87,0670697 |
| 87,584677 | 87,0780358 |
| 87,5923089 | 87,1096671 |
| 87,5980093 | 87,120164 |
| 87,6268499 | 87,1455733 |
| 87,6290186 | 87,1591267 |
| 87,63251 | 87,324139 |
| 87,6713647 | 87,32923 |
| 87,6745158 | 87,3381655 |
| 87,6821612 | 87,3601709 |
| 87,6986996 | 87,3788411 |
| 87,7030862 | 87,392577 |
| 87,7122569 | 87,4034413 |
| 87,7139434 | 87,4206725 |
| 87,7196057 | 87,4587384 |
| 87,7220948 | 87,4791941 |
| 87,7684388 | 87,5427434 |
| 87,7837061 | 87,6096102 |
| 87,8080844 | 87,6182516 |
| 87,8117816 | 87,6257827 |
| 87,8258229 | 87,6440819 |
| 87,8436815 | 87,6495409 |
| 87,8722143 | 87,6609152 |
| 87,8796493 | 87,6981599 |
| 87,8948502 | 87,7080681 |
| 87,9003324 | 87,7777886 |
| 87,9093337 | 87,871769 |
| 87,9155903 | 87,8910913 |
| 87,9273333 | 87,8936605 |
| 87,93048 | 87,9003357 |
| 87,9352008 | 87,9138526 |

| | |
|---|---|
| 87,9356153 | 87,9365431 |
| 87,9772514 | 88,0046662 |
| 87,9835376 | 88,0267703 |
| 87,9970415 | 88,0643011 |
| 87,9982285 | 88,0795793 |
| 87,9988983 | 88,0996742 |
| 88,0013757 | 88,1231748 |
| 88,0453805 | 88,1934617 |
| 88,0733693 | 88,2018276 |
| 88,0861262 | 88,2091504 |
| 88,0914742 | 88,2205427 |
| 88,106521 | 88,2562096 |
| 88,1185266 | 88,2568383 |
| 88,1484112 | 88,2632694 |
| 88,1653775 | 88,2993805 |
| 88,1760661 | 88,3048865 |
| 88,1986819 | 88,3237098 |
| 88,2159964 | 88,3271985 |
| 88,2199539 | 88,3300906 |
| 88,2333301 | 88,3331668 |
| 88,2687636 | 88,3442617 |
| 88,2940206 | 88,3487507 |
| 88,2993339 | 88,4117978 |
| 88,3183986 | 88,482074 |
| 88,332052 | 88,4876107 |
| 88,384409 | 88,4879559 |
| 88,3873184 | 88,4881319 |
| 88,3887325 | 88,4902464 |
| 88,4395472 | 88,5080911 |
| 88,4485068 | 88,5107254 |
| 88,4774524 | 88,5277676 |
| 88,506099 | 88,5555442 |
| 88,5308482 | 88,5825715 |
| 88,5352015 | 88,6041822 |
| 88,549122 | 88,6129181 |
| 88,550531 | 88,6196892 |
| 88,5538974 | 88,6820665 |
| 88,5547919 | 88,6841929 |
| 88,5953659 | 88,6877182 |
| 88,5987738 | 88,6925273 |
| 88,6002291 | 88,7185792 |
| 88,6256165 | 88,7385847 |
| 88,6333844 | 88,7404486 |
| 88,6357847 | 88,7675878 |
| 88,6451087 | 88,7690162 |
| 88,6473617 | 88,7796426 |
| 88,649392 | 88,7905765 |

| | |
|---|---|
| 88,6505536 | 88,8120074 |
| 88,7324009 | 88,8198682 |
| 88,7581341 | 88,833427 |
| 88,7678215 | 88,8370382 |
| 88,7838462 | 88,8394664 |
| 88,7991401 | 88,8729985 |
| 88,8002964 | 88,9334665 |
| 88,8100051 | 88,9442286 |
| 88,8108665 | 88,9603038 |
| 88,8198354 | 88,9680032 |
| 88,8597007 | 88,9797625 |
| 88,8781976 | 89,0072395 |
| 88,8979425 | 89,0074849 |
| 88,9422413 | 89,0150482 |
| 88,9489771 | 89,0184224 |
| 88,9691647 | 89,028879 |
| 88,9700584 | 89,0508538 |
| 88,9857343 | 89,0654005 |
| 89,0008134 | 89,0732085 |
| 89,0029643 | 89,1413823 |
| 89,0299777 | 89,1427563 |
| 89,0711217 | 89,1804252 |
| 89,0759345 | 89,194703 |
| 89,0965928 | 89,2707912 |
| 89,1005516 | 89,2840837 |
| 89,1166864 | 89,3071415 |
| 89,146499 | 89,3083059 |
| 89,1541887 | 89,3333213 |
| 89,1713523 | 89,3361335 |
| 89,2141741 | 89,3606707 |
| 89,2290483 | 89,3627986 |
| 89,2318927 | 89,3708589 |
| 89,2331769 | 89,3782735 |
| 89,2424607 | 89,429321 |
| 89,2673952 | 89,4413187 |
| 89,3167325 | 89,4514764 |
| 89,3288343 | 89,4542898 |
| 89,3377528 | 89,4720571 |
| 89,3887426 | 89,4777197 |
| 89,3949725 | 89,4892159 |
| 89,4020793 | 89,4964469 |
| 89,4394081 | 89,5089558 |
| 89,4569053 | 89,5368213 |
| 89,4679841 | 89,5490105 |
| 89,4860755 | 89,5641821 |
| 89,4873706 | 89,5685305 |
| 89,5005665 | 89,5933286 |

| | |
|---|---|
| 89,5024134 | 89,60187 |
| 89,5385231 | 89,6247351 |
| 89,5557727 | 89,6308898 |
| 89,5574699 | 89,6321339 |
| 89,5780231 | 89,6457315 |
| 89,5959175 | 89,6602478 |
| 89,6336713 | 89,6869413 |
| 89,6477122 | 89,6918099 |
| 89,6995961 | 89,7095191 |
| 89,726307 | 89,7567241 |
| 89,7307728 | 89,765261 |
| 89,7546125 | 89,7845486 |
| 89,7679632 | 89,7860589 |
| 89,7828413 | 89,7913993 |
| 89,8011471 | 89,7917712 |
| 89,8056492 | 89,8189506 |
| 89,8111249 | 89,822263 |
| 89,8117292 | 89,823514 |
| 89,8130959 | 89,8267783 |
| 89,8148587 | 89,8499839 |
| 89,8268873 | 89,8559748 |
| 89,8546954 | 89,8613142 |
| 89,8676088 | 89,8693955 |
| 89,8687826 | 89,8946684 |
| 89,8798855 | 89,9207057 |
| 89,9213271 | 89,963658 |
| 89,9302059 | 90 |
| 89,9379007 | 90 |
| 89,9604271 | 90 |
| 89,9608714 | 90 |
| 89,9775895 | 90 |
| 89,9778545 | 90 |
| 89,982745 | 90 |
| 89,9917969 | 90 |
| 90 | 90 |
| 90 | 90 |
| 90 | 90 |
| 90 | 90 |
| 90 | 90 |
| 90 | 90 |
| 90 | 90 |
| 90 | 90 |
| 90 | 90 |
| 90 | 90 |
| 90 | 90 |
| 90 | 90 |
| 90 | 90 |

| | |
|---|---|
| 90 | 90 |
| 90 | |
| 90 | |
| 90 | |
| 90 | |
| 90 | |
| 90 | |
| 90 | |
| 90 | |
| 90 | |
| 90 | |
| 90 | |
| 90 | |
| 90 | |
| 90 | |
| 90 | |
| 90 | |
| 90 | |
| 90 | |
| 90 | |
| 90 | |
