# Supplementary material for: Cyclic stretch induces autophagy-mediated focal adhesion remodeling and activates mitochondria
Source: Life Sci Alliance. 2026 Feb 5;9(4):e202503347. doi: 10.26508/lsa.202503347 (PMC12877405; doi:10.26508/lsa.202503347)
Supplement: Supplementary file 4 [file LSA-2025-03347_SdataF4.pdf]

Source data Figure 4B-C

Main orientation of actin fibers per cell equivalent in [°]

unstr. WT (a)	unstr. T285D-T289D (b)	30 min str. WT (c)	30 min str. T285D-T289D (d)	1 h str WT (e)
0,5131714	0,30072457	0,43965095	0,38543898	12,5399187
0,95961194	0,41667331	2,23444031	1,52062742	39,6384023
1,00785451	0,58526815	6,16778375	2,22616519	44,0935938
1,13954668	1,60344684	11,9166645	4,93748578	45,6641399
2,15566902	1,95119232	11,9870019	5,26326492	45,9858379
2,48709925	4,46271624	21,4607868	6,45313814	46,480432
2,50862208	4,47994758	23,7487009	9,55285773	47,7431223
3,29296613	5,05258998	26,8683027	10,3250305	48,679233
3,48015371	5,30076953	27,0058753	10,4727479	48,7617718
3,55320662	5,68632436	27,3157869	11,0724762	49,1507653
4,06708678	6,25207581	27,4925152	11,1585003	49,8737232
4,34902364	6,51558982	29,3774905	11,7881998	49,9034942
6,02880875	7,01969154	32,7754954	12,1138273	50,1934951
6,03552565	10,0525207	35,983838	15,5871027	50,2621734
6,3875508	10,1466491	37,72586	18,1419385	50,4827501
7,16771518	11,4356597	38,6644776	23,0716171	50,9069308
8,81248945	12,2614191	38,6851333	23,9885645	51,2091047
9,50619903	14,9973051	38,7183467	24,8410089	51,7959332
9,51310596	15,0550652	39,0768281	27,5891199	51,8097397
9,63813036	15,1628863	39,521816	28,0155255	52,2004347
9,71423367	15,8917154	39,652891	28,4761735	52,3297635
9,76275304	16,5765463	39,7526393	30,2230182	52,3815983
11,1360616	16,8981269	40,7828802	30,2566201	52,5731754
11,9860239	17,4385098	41,432657	30,8619698	52,6507397
12,1232857	17,753043	42,3103404	34,9327718	53,0172458
12,201833	18,1443999	43,0792509	35,779701	53,1595051
12,3926462	18,2839886	43,1956631	36,7108158	53,4234695
12,4423642	18,8057948	43,8863204	37,420962	54,3050505
12,7637436	20,069219	44,3671904	38,0545046	54,440692
13,452702	20,6075576	44,5038825	38,2905624	54,7101556
13,8673223	20,8810497	44,6806897	38,6257048	55,4163496
14,9596437	21,4656556	44,7793449	39,00489	55,497884
14,9812732	21,9074903	45,1723826	39,0828271	55,7633472
15,3418648	22,5142776	45,82106	39,9255213	55,854431
16,1297022	22,8405893	45,8754578	39,9348548	56,0997533
16,4834462	23,7867327	46,6286015	40,2963733	56,3495055
17,0283202	23,972496	47,1683543	40,5256673	57,2163202
18,235316	24,2534772	47,2811036	40,792132	57,5875365
19,0321857	26,454303	47,5550226	42,7205299	58,5330117
19,3011447	26,6196088	47,8157711	43,5819015	58,552167
20,0058957	26,6929006	47,8311663	43,8168629	58,5742857
20,5963812	26,7487773	48,2714684	44,3140599	58,5874693
20,912625	26,864976	48,4212768	44,3629473	59,7922898
23,248317	26,8732951	48,4939805	45,7906468	59,8327051

24,2758822	26,8884078	48,5664007	45,8133742	59,9495844
24,6492212	26,9517691	48,8835634	45,9443516	60,4236173
25,7589615	27,4646595	48,9904137	46,3247518	60,5165694
25,9362762	27,8112758	49,2806214	46,4115957	60,526267
26,2273209	29,0396717	49,5523931	46,9307517	60,6274899
26,3340471	29,5464543	50,1345856	47,0257478	60,6652725
26,645812	29,6993819	50,4181609	47,225452	60,7494204
27,3761798	29,8532341	50,4626568	47,5756423	60,7600099
27,6292717	30,754263	50,5814452	47,7044698	60,8953931
28,5724357	31,2253038	50,7396661	47,9377901	61,1087788
28,7732202	31,4082803	50,9368525	47,9991464	61,7600133
31,1073118	32,2889766	50,9641485	48,0087363	61,8791335
31,1776673	32,6725755	51,0468127	48,1664835	61,977075
31,3092395	34,7307519	51,6539123	49,1103592	61,9940715
31,8699999	34,8028041	51,8693563	49,1295215	62,1938938
31,8873021	36,8875541	51,8887843	49,836567	62,2406087
31,9425029	37,14036	52,1954102	49,8749638	62,2952557
32,0260739	37,636441	52,2866485	50,1025236	62,372621
32,5390797	37,7807955	52,4185865	51,1895763	62,8179397
34,0736617	38,0371814	52,5239541	51,3098398	62,8853407
34,3767669	38,3001359	52,6031938	51,8706647	62,9145381
34,9367886	38,6296184	52,9063871	51,8958331	63,2090502
35,353004	38,6616218	52,9839561	52,5832028	63,3151097
35,7647525	38,7903566	53,0788696	52,9832404	63,396237
36,3541631	38,8021929	53,1291497	52,984725	63,4204255
36,4100672	39,1096901	53,964208	53,2661876	63,5537917
36,5976417	39,3515509	54,1478654	54,1505117	63,6825557
36,9955294	39,81489	54,155809	54,7415118	63,7732985
37,8093159	40,0364512	54,162038	54,8458547	63,7895691
38,8066008	40,8681723	54,2968361	55,1411888	63,884576
38,931949	40,9113286	54,8475914	55,2494173	63,9925111
39,3050861	41,0469428	55,029967	55,8285983	63,9992264
40,2558406	41,7334519	55,2338296	55,8962609	64,0957965
40,9933049	42,8559904	55,2868133	56,2155439	64,2838621
41,069761	42,8717856	55,7121493	56,4425554	64,6583252
41,1069378	43,0034727	55,7688483	56,7352097	64,7393632
41,5797785	43,1313004	56,0108614	56,8211625	64,8379329
42,621181	43,3617054	56,1070797	57,0836897	64,8405471
42,901745	43,5682749	56,1192792	57,1037742	64,8719687
43,6802407	43,8475663	56,3741089	57,127543	64,981517
44,526208	44,0537791	56,4144651	57,1645421	65,1068259
44,7274868	44,3883445	56,5657181	57,2068008	65,164538
45,5531296	44,4821434	56,6110134	57,5869182	65,1793064
45,5715877	44,9718339	56,8371797	57,923358	65,1910155
46,3597308	46,2191528	56,8767247	58,0451488	65,630745
46,8347632	48,282894	57,248791	58,2851599	65,7912077
48,0999336	48,4692952	57,2839007	58,4093622	66,4016284

48,1860866	49,0000384	57,5648226	58,7656277	66,4493643
48,3185009	49,6416399	57,6054149	58,8341561	67,2790293
48,4295582	49,8114983	57,681766	58,9039207	67,2798908
48,4489357	49,9918616	57,8373568	59,0236193	67,3638834
48,9746246	52,5494613	58,3322089	59,147057	67,6901683
49,0330322	52,7199423	58,4084507	59,3549236	67,8501975
49,5730012	54,4920568	58,4674761	59,3647311	67,8728066
49,7664605	54,7090258	58,7188702	59,424429	68,0077912
49,8416199	55,6379296	58,7599667	59,6012279	68,0651603
50,1266583	56,1643264	58,9026491	59,6617624	68,074046
50,4197684	56,8376573	59,005485	60,0332305	68,1018559
50,9873936	57,7037953	59,0426676	60,152415	68,4543615
51,0632226	57,7115356	59,0789427	60,4540075	68,487676
51,6846156	57,8054416	59,2253915	60,6524385	68,5774826
52,2548813	58,1614692	59,4761141	61,2231813	68,5938613
52,4716596	58,3806782	59,8067574	61,299359	68,770824
52,8259472	58,7213617	59,8534158	61,3126531	68,8668369
52,8699396	58,7698263	59,9314561	61,5868117	69,3141059
53,0877462	59,4737709	60,0488909	62,244437	69,8844436
53,1237677	59,9718889	60,156609	62,3612756	69,892404
53,5767331	61,4898214	60,2969859	62,5166249	70,2220712
54,9380159	62,6252844	61,0634704	62,6111921	70,3537156
54,9782265	63,1982803	61,0723975	63,2743762	70,5181163
55,1923533	63,3123939	61,2324825	63,5400568	70,583779
55,2103072	63,6850667	61,5305896	63,5797873	70,6737306
55,3184694	63,7668411	62,6208465	64,0307287	70,8183502
55,3241294	63,8647543	62,8898878	64,0406773	71,3327761
55,4968234	64,3689197	63,0689842	65,0123489	71,3400672
55,8403255	64,5928821	63,1362525	65,1976625	71,4957716
56,1665728	65,4235618	63,4339362	65,3413558	71,6213929
57,6942847	65,4926657	64,2274831	66,2273553	72,1330052
57,6980013	65,7844077	64,4265248	66,4816375	72,5705351
58,3101703	66,2610539	64,4869482	67,438918	72,5850489
58,3943525	66,735057	64,7497844	67,5733644	72,9985891
58,5302788	66,9755641	65,0125018	68,3236949	73,1199631
59,0375471	67,4421469	65,1136634	68,8836152	73,1226364
59,0786501	67,752125	65,1873904	69,30603	73,5727896
59,4094828	68,1977616	65,9483112	69,4897967	73,777467
59,4487471	68,3389679	66,0928751	69,8756732	73,8842573
60,040126	68,6178904	66,1962073	70,1468148	74,115696
60,4937256	69,1612003	66,2959902	70,3058648	74,3195187
60,5931087	70,2270518	66,4275343	71,4209225	74,6073541
60,6388542	70,4183385	66,6384741	71,4393299	75,7636274
60,7883484	70,69174	67,4699741	71,8534041	75,9157991
61,306085	70,7172309	67,6398761	71,8913317	76,0679968
61,7305172	71,1270775	67,672462	72,5146623	76,120879
61,7687544	71,2515143	68,5624254	73,6261279	76,415702

62,7229201	71,6273966	69,0578307	75,8573959	76,5779219
63,1278698	72,3439675	69,3546015	76,4268866	76,6839524
63,6953098	72,8803212	69,6636683	77,3478195	76,7436625
64,1092989	73,0743829	69,8693896	77,5135981	76,9827125
64,1366948	73,2340365	70,4982674	77,8354469	77,344631
64,2208885	74,3992552	70,8368988	78,111582	77,7057749
64,5520471	74,5464069	71,0882411	79,3372648	78,0019704
64,6644515	74,5566845	71,2543934	79,5752656	78,1244965
64,9590141	75,2105656	71,2551709	80,7433925	78,6511486
65,5301051	75,6143588	71,2821869	81,8010213	79,078593
65,6496394	75,7884473	71,3645682	81,837728	79,501867
65,7639713	75,8800901	71,511169	82,2548334	80,3810668
65,869064	75,9264554	71,5469076	82,5943002	80,4568092
65,8864183	76,8033521	71,5697677	83,1168835	80,6277257
66,3042373	76,9731268	71,6300569	83,3187376	80,7478745
67,3325969	78,0351349	71,9613422	83,5590849	81,6425126
67,5705913	79,1987063	71,9966959	84,1142037	83,1557516
68,1014651	80,1539217	72,1763515	84,976132	83,3366609
68,3909876	80,6204662	72,5701307	85,1467406	83,380492
68,4207241	80,9645373	72,8297248	85,2814228	83,5532269
68,734156	80,9998872	73,1187015	85,5874917	83,8600369
68,794928	81,5838302	73,4466546	85,8014829	84,072521
68,9531517	82,7961314	74,2201561	87,4234013	84,2553005
69,8185547	82,8778348	74,2238441	87,4818303	84,7765899
69,8626659	83,5461419	74,2448462	88,5687066	85,3773615
69,9683979	84,4768078	74,2803044	88,9538937	86,1717308
70,160979	85,1986868	74,5114785		86,6953797
70,4852878	85,2157916	75,524432		86,8765727
71,1359306	85,8299017	75,5702671		87,3171421
71,9074893	86,1334829	76,4199144		87,9836032
72,0360688	86,8834914	76,4353574		88,5406107
72,0998323	87,2877002	76,4790893		88,5406613
72,1713479	87,480546	77,650301		88,5626505
72,2146128	87,7908073	78,0827127		88,56665
72,4471137	87,8577597	78,5617767		88,6732015
73,0802193	88,6294509	78,7634321		88,7653554
73,1910926	89,048659	78,9036018		89,0093972
73,5839176	89,1955729	79,1599988		89,0628471
73,749116	89,3687635	79,3516121		89,2837296
73,9906859	89,5089023	79,8338357		89,7251883
74,6687491	90	79,9191966		89,8895327
75,6113433		79,9529985		90
75,7625038		80,2854992		90
75,8183342		80,3409756		90
76,1971912		80,4628806		90
76,2281211		80,7521593		
76,2971523		80,9246945		

76,569159	81,0702799
77,0217112	81,6462884
77,1642983	81,7161686
77,2030583	82,158447
77,219678	82,2897357
77,7293369	82,4659034
77,9007451	83,3450888
78,0611699	83,9750206
78,0874891	84,371396
78,389286	84,7135981
78,6206239	86,0626341
78,6617248	86,4500593
79,1509313	86,4665063
79,2987247	87,9316152
79,3222334	88,2311664
79,3838303	88,6832422
80,5032799	88,7367296
80,7781811	89,0865453
80,8636848	89,6478426
80,9024412	89,6749198
80,9313172	90
81,0088476	
81,3323924	
81,8927944	
82,2744692	
82,2957663	
82,580198	
82,7057049	
83,6035554	
84,1384722	
84,8014619	
85,4152078	
87,1481671	
87,7272446	
88,0736748	
88,2393413	
88,6488506	
88,9647589	
89,0381219	
89,1839954	

1 h str. T285D-T289D (f)	4 h str. WT (g)	4 h str. T285D-T289D (h)
0,37755699	47,7388368	44,7011978
0,75663071	50,9207125	51,6437406
1,92049312	51,0131621	54,1771029
7,01929434	52,8153177	54,4375798
8,31229487	53,2462978	55,1615313
9,15481905	54,3595235	57,7176337
11,6779834	55,2528988	62,7009013
12,7077414	55,8636929	63,0737487
20,0601978	58,9836807	63,2179522
23,8241204	60,0753221	63,5776655
25,4630487	60,1407737	63,712098
32,156036	60,8209502	64,4430991
32,3834268	61,1278785	64,9513344
33,1765277	61,2889761	65,0317682
33,6619297	61,335894	65,2065605
35,9335092	61,4987672	65,273246
36,1550077	61,5546367	65,8792927
36,981285	61,8722442	66,0650351
39,0630507	62,1502249	66,0769021
42,8890136	62,2872776	66,3709713
43,1822124	62,4432499	66,6793238
43,5841992	62,5714778	66,8352621
43,8136867	62,8478795	67,0968955
44,5894171	63,1895198	67,2118398
44,6392262	63,3515238	67,4733273
44,6528076	63,481621	67,6707745
44,9714329	63,8205811	67,9303446
45,0984521	63,8431065	67,9669825
46,4142955	63,9885499	68,1425822
46,5038516	64,1399364	68,9666929
48,0122144	64,8258199	69,5096157
48,8205582	65,1694917	69,7648301
48,869179	65,3598644	69,9626533
49,5204336	65,6160195	70,5496316
49,5692538	65,7149784	70,5736579
49,6727683	66,1168945	70,7324796
49,7244884	66,1484507	70,9029329
49,7771648	66,2233006	71,0451931
50,0119633	66,2505331	71,3393124
50,1146951	66,4168643	71,4076823
50,1253239	66,4540351	71,6934546
50,2401457	66,4744898	72,1954999
50,5041474	66,6605236	72,3413108
51,0264839	66,7900218	72,3573686

52,4607627	66,9508656	72,8290793
52,4923553	67,00879	73,1038196
52,7176885	67,230891	73,5886748
52,7552291	67,2553745	73,83466
52,9776585	67,275975	74,3417002
53,013537	67,3234595	74,7027304
53,8514341	67,4015944	75,0504547
54,1072288	67,4414704	75,1969427
54,1202607	67,4612911	75,9560634
54,3792607	67,6086721	76,0191031
54,3865316	67,8080217	76,3994234
54,4869933	67,9227974	76,6788456
54,5044592	68,3015075	77,0179335
54,6168851	68,3638069	77,1403837
54,812027	68,3945124	78,3906227
55,0301783	68,4021601	79,2031452
55,7514321	68,4397489	79,775092
55,9770885	68,4888344	79,9825326
56,1945261	68,6259272	80,0854109
56,529291	68,7386807	81,3094395
56,560145	69,0615683	81,9616037
56,634001	69,2333158	81,9903717
56,6595609	69,2658599	82,4673349
56,6810889	69,3484582	83,4662889
56,7048841	69,4312698	83,7594864
56,9236172	69,4886185	84,0352967
57,0230389	69,5183304	85,4045731
57,5814284	69,7986547	86,8507637
57,6507259	69,9451505	87,32443
57,7961867	70,5025412	89,6226953
58,0681295	70,8233993	
58,3651195	70,8761915	
58,5307112	70,9527017	
58,9395423	71,3984052	
59,1032419	71,5155199	
59,1395689	71,5311536	
59,8231978	71,5458165	
59,8972472	71,5592144	
59,9340033	71,7878258	
60,1157479	71,7936588	
60,1749165	71,9557027	
60,3012008	72,1328549	
60,5243384	72,1887381	
60,8240941	72,3543568	
61,0584913	72,3593696	
61,197626	72,5686583	
61,5929657	72,5764696	

61,608749	72,5822202
62,0883956	72,7407806
62,306845	72,8433641
62,34591	73,0777723
62,4875286	73,1549812
62,8057601	73,157985
62,8129161	73,2471239
62,850288	73,4005013
63,2226047	73,4548265
63,5773839	73,5201642
63,7623093	73,5435083
64,3551221	73,6907993
64,4448973	73,6928804
64,4493856	73,6992216
64,4708003	73,722918
64,8213239	73,8628205
65,1782397	73,9363994
65,4144769	73,9859177
66,3109286	73,9893343
66,6167571	74,1466483
66,735652	74,1871549
67,2639042	74,3033527
67,5939269	74,3206511
67,8650919	74,4020328
69,7667949	74,4438007
70,2251261	74,5094499
70,3348287	74,5797997
70,5972553	74,5810373
70,9709908	74,6142984
71,3140956	74,6594122
71,5032454	74,8275976
71,6366626	74,8283101
73,5952575	74,8309676
73,6738144	74,8769588
74,4464008	74,9141264
74,5852573	75,0704248
75,6585213	75,1257049
75,8718832	75,2066977
76,3561938	75,2788658
76,4009026	75,4741537
77,122536	75,6426136
77,228588	75,7912575
77,6877226	75,8334295
78,2613677	75,8742232
78,7728775	75,9156282
79,6169186	76,0620772
80,0870103	76,0655834

80,0871314	76,1102755
80,1644185	76,1195799
80,3160186	76,419202
81,4951222	76,6915016
82,313272	76,7094658
82,4801193	76,7710539
83,6612458	76,8657781
83,772766	76,9213822
84,7484437	76,948548
85,7772286	77,0184331
88,89772	77,2291703
	77,2369854
	77,4374403
	77,5195254
	77,5482777
	77,6599863
	77,7128593
	77,7493638
	77,7702479
	77,8752789
	78,0321614
	78,045734
	78,0786396
	78,1052119
	78,1976942
	78,2501947
	78,3399741
	78,3904692
	78,5178209
	78,6935581
	78,7642501
	78,8557554
	79,221285
	79,2383771
	79,3993687
	79,4299633
	79,5263931
	80,2336361
	80,3435824
	80,3699357
	80,3883385
	80,513804
	80,5546221
	80,5856969
	80,7034953
	80,7633681
	80,8045722

81,0904811
81,1062613
81,1268749
81,3131878
81,3154152
81,8947066
82,0590976
82,073311
82,0807934
82,1003371
82,1153192
82,2701215
82,3303336
82,3686525
82,6589691
82,6620827
82,7715157
82,8861907
83,0248976
83,1490456
83,4009349
83,5228653
83,6129317
83,6889835
83,7521419
84,0473595
84,3631626
84,4576851
84,5153584
84,5978595
84,6063172
84,671215
84,7247855
84,7756541
84,8384182
84,862367
84,9835575
85,0880487
85,1943843
85,286882
85,6040784
85,7717799
85,8708943
85,9436124
85,9723879
86,2288992
86,284054

86,2903189
86,333143
86,4053223
86,5630047
86,6328092
86,7700377
86,7861321
86,7999973
86,9283485
87,0141251
87,2380142
87,3642342
87,3701737
87,4249348
87,5082141
87,6023527
87,7838107
87,9511317
87,9681513
87,9759395
87,9895616
88,089611
88,4179132
88,4951183
88,5372545
88,5487026
88,5934719
88,635595
88,7091839
88,8429406
89,1220728
89,1504644
89,1672593
89,466248
89,7461565
89,8482993
90
90
90
90
90
90
90
