# Supplementary material for: Cyclic stretch induces autophagy-mediated focal adhesion remodeling and activates mitochondria
Source: Life Sci Alliance. 2026 Feb 5;9(4):e202503347. doi: 10.26508/lsa.202503347 (PMC12877405; doi:10.26508/lsa.202503347)
Supplement: Supplementary file 6 [file LSA-2025-03347_SdataF6.pdf]

Source data Figure 6B-G

Fig. 6B paxillin area

unstr.	1h str.	4h str.
2,91	2,76	2,30
2,98	2,91	2,63
2,84	2,22	3,15
4,26	2,71	2,37
3,11	2,52	2,74
2,64	2,97	2,39
3,23	2,08	2,65
2,66	2,65	2,64
2,90	2,43	2,70
3,09	2,75	2,98
3,33	2,82	2,64
3,14	2,84	
	2,77	
	2,84	

Fig. 6C vinuclin area

unstr.	1h str.	4h str.
2,44	2,75	2,05
2,81	2,53	2,47
2,58	2,10	2,60
3,77	2,57	2,25
2,92	2,42	2,64
2,41	2,83	2,44
2,71	2,74	2,55
2,62	2,58	2,59
2,33	2,34	2,60
3,44	2,75	2,80
3,71	2,71	2,59
3,34	2,41	
	2,34	
	2,45	

Fig. 6D paxillin coverage

unstr.	1h str.	4h str.
76	73	71
75	72	73
75	72	71
73	72	71
72	71	74
73	74	67
77	60	70
72	71	75
76	73	74
68	70	75
68	73	75
67	77	
	76	
	77	

Fig. 6E vinculin coverage

unstr.	1h str.	1h str.
67	71	64
71	68	67
70	67	62
65	69	67
67	71	70
67	71	66
67	70	66
69	68	73
65	68	70
71	68	70
73	69	73
70	66	
	67	
	67	

Fig. 6F FA area

unstr.	1h str.	4h str.
4,55	4,49	3,73
4,90	4,72	4,38
4,37	3,64	5,20
6,73	4,47	3,89
5,35	4,25	4,49
4,25	4,83	4,58
4,89	4,80	4,58
4,55	4,66	4,12
4,35	4,06	4,26
5,92	4,95	4,64
6,49	4,57	4,12
6,19	4,20	

Fig. 6G FA number

unstr.	1h str.	4h str.
564	533	637
673	569	542
472	549	508
580	520	567
436	602	591
632	512	579
398	623	669
643	573	662
430	552	546
632	477	515
635	570	662
720	641	

4,19	474
4,28	526

Source data figure 6I

FA reorientation

unstr.orientation [°]	1h str. orientation [°]	4h str. orientation [°]
0,215715981	0,239987402	0,013532163
0,348201517	0,271667285	0,05259107
0,365118494	0,346983311	0,093255617
0,37906735	0,35505072	0,17367299
0,400066296	0,380314858	0,234091722
0,66896897	0,428227974	0,310489593
0,717415577	0,452021924	0,310489593
0,739270704	0,523269952	0,479918681
0,761933238	0,573038162	0,566264268
0,818172698	0,606382297	0,590090786
0,975998622	0,616424114	0,758094015
1,269774408	0,628198078	0,758094015
1,348342448	0,637102627	0,887379554
1,40266922	0,735091043	0,901578745
1,466145189	0,760004527	0,94628309
1,475573675	0,807900313	1,298413645
1,519370167	0,912609314	1,310646989
1,612552925	0,944704023	1,3975069
1,79921571	1,088891207	1,417506355
2,054403141	1,13115231	1,417506355
2,124595143	1,143038077	1,610834919
2,322313158	1,46926276	1,78955492
2,344153067	1,501055267	1,848545455
2,372315495	1,512478635	1,876573265
2,39854552	1,517784563	1,893742309
2,672132382	1,517972759	2,061163113
2,835412579	1,637272357	2,108508027
2,887467214	1,705302766	2,127166658
2,911686586	1,727071659	2,141457942
3,163102097	1,815183649	2,186663654
3,455097763	1,818412663	2,455312505
3,58791151	1,876348366	2,557786447
3,605061262	1,899257933	2,603493256
3,700011665	1,955174557	2,610030985
3,780082259	1,960425149	2,610030985
4,065774617	2,168001467	2,664857868
4,175997383	2,192876149	2,927745967
4,416741325	2,355080059	2,946684069
4,512823748	2,404779287	2,946684069
4,550427038	2,542274086	3,015891657
4,584187744	2,753497305	3,109703616
4,645054953	2,769549088	3,222426116
4,767298143	2,804901799	3,274323614
4,768857386	2,833472287	3,369944049
4,803418386	2,846069149	3,420692742
4,858617151	2,846251315	3,42705518
4,966126813	2,857565193	3,543665615

5,013831394	2,896860562	3,609887052
5,149161636	2,969795205	3,645844701
5,349823945	3,00384706	3,852938423
5,502494101	3,07201982	3,852938423
5,56388292	3,096740828	3,887412274
5,664860886	3,148874351	3,930328526
5,66981982	3,176568699	4,089301864
5,910728955	3,190331608	4,112758414
5,939681998	3,256327634	4,129006419
5,976705593	3,415305401	4,186788848
6,247365854	3,42492761	4,238883893
6,344533885	3,439649312	4,26915431
6,363541068	3,450751734	4,28208366
6,384291338	3,4568103	4,289957959
6,439702155	3,47621351	4,379637463
6,532167349	3,523314511	4,419925156
6,536376797	3,54477096	4,440209724
6,604372325	3,584853412	4,452305462
6,620471958	3,717442526	4,452305462
6,742259991	3,768030526	4,506571412
6,744512054	3,82458902	4,506571412
6,944093584	3,90209801	4,529351205
7,107823205	3,976855836	4,668014257
7,26867475	4,010632411	4,720690067
7,532646027	4,134992974	4,765340259
7,610547541	4,171971068	4,879511062
7,620757977	4,190373801	4,94466496
7,887082957	4,209161667	5,093288447
7,895454075	4,291953113	5,426523818
7,932876225	4,294093682	5,468486331
8,02762159	4,621598347	5,483764229
8,199275514	4,627065065	5,572894306
8,232157797	4,730126891	5,5766075
8,269275621	4,730749466	5,5766075
8,303612649	4,78711872	5,693232574
8,343219269	4,793010133	5,693232574
8,658325991	4,807900973	5,820109638
8,661352822	4,849284967	5,822559421
8,748746868	4,861171477	5,92539475
8,824835234	4,867902537	6,113554929
8,848952268	4,876534026	6,260148541
9,008306437	4,916663066	6,296944962
9,134309021	5,041823862	6,326904013
9,3080888	5,06918967	6,383086458
9,380228884	5,130947657	6,428758778
9,416575754	5,239674117	6,428832213
9,861906253	5,370628568	6,537512195
9,863696961	5,417287027	6,604201882
9,948329576	5,49733012	6,643665593
9,955657095	5,524639753	6,722133525

10,09840137	5,5513998	6,726394169
10,26729422	5,708830566	6,87133017
10,445343	5,74551077	6,943433292
10,47028606	5,876055416	6,977480874
10,60332996	5,877695965	6,978630011
10,67755935	5,879077222	7,230593459
10,7928566	6,007572533	7,244891989
10,84012428	6,069369419	7,334902172
11,29488783	6,077833613	7,428998904
11,47950135	6,150252088	7,464576577
11,5431648	6,185630615	7,53463463
11,65275061	6,192863052	7,557275157
11,70015251	6,246537459	7,633013381
11,72349306	6,304898738	7,633013381
11,77677854	6,485237575	7,638344937
11,99385298	6,511335637	7,638344937
12,00269301	6,57094309	7,686756254
12,04503849	6,576462689	7,686756254
12,05107048	6,592222256	7,761502181
12,23991647	6,712788653	7,793437707
12,29952006	6,748348021	7,793437707
12,48321044	6,781655873	7,808134212
12,5081983	6,841023692	7,816800237
12,50941595	6,845552873	7,828513426
12,68243637	6,857950466	7,828513426
12,68363697	6,950263274	7,830022864
12,77345422	6,985103026	7,834675067
12,7787248	7,090367878	7,905340147
12,95423324	7,135219162	7,999274802
13,01222242	7,160579879	8,155316897
13,14693875	7,217752849	8,203878861
13,2036639	7,238757216	8,207559843
13,59763609	7,294818146	8,207559843
13,74806453	7,296102561	8,275010393
13,76873254	7,35260092	8,393136332
14,07573758	7,495452962	8,393136332
14,11602502	7,630653499	8,402207015
14,194222	7,727674987	8,402207015
14,27711125	7,817374431	8,460704627
14,56971646	7,910161665	8,460704627
14,57487897	7,969520162	8,463129157
14,59099729	7,969979547	8,463129157
14,6648572	8,041051562	8,485540642
14,74989834	8,051239407	8,642682344
14,76931463	8,054934126	8,687394926
14,79244601	8,067573563	8,687394926
14,83822499	8,164184917	8,699406704
14,85480151	8,199099069	8,781240053
14,89707619	8,270380264	8,863073881
15,02483844	8,302176293	8,884738265

15,17589455	8,357607175	8,938552343
15,19897139	8,482906252	8,94328172
15,32353447	8,53349597	9,018737363
15,45816277	8,567596378	9,070198677
15,54051985	8,666548046	9,138526733
15,84776202	8,696898883	9,154017156
16,12371962	8,756487382	9,233103537
16,15456406	8,775387415	9,352852762
16,36013492	8,817736526	9,357299583
16,56085658	8,825207519	9,397227135
16,90941236	8,838809519	9,475209013
16,91501548	8,867320821	9,475209013
16,92095681	9,019962537	9,475498087
16,93952079	9,069025002	9,481608143
17,0259078	9,075651659	9,58031523
17,06677093	9,11202173	9,586042814
17,30237036	9,338891346	9,69682194
17,31438926	9,348713677	9,69682194
17,34718158	9,36294427	9,703427661
17,46122171	9,365202738	9,846343312
17,75053232	9,371589768	9,87579382
17,94475262	9,444890137	9,941430017
18,12948074	9,466610725	9,966022961
18,1721355	9,492638865	10,02490913
18,18529316	9,538500656	10,06936643
18,21126141	9,69796483	10,11965419
18,42149297	9,709455889	10,12243071
18,55237873	9,889022141	10,26523125
18,55302866	9,983803401	10,26523125
18,59636549	10,08982672	10,33764556
19,00003293	10,11754957	10,33764556
19,02838982	10,19739101	10,41861694
19,04772741	10,29518998	10,45595521
19,14519133	10,44998616	10,5125206
19,24152827	10,45636179	10,5125206
19,94197035	10,49467744	10,53557705
20,064112	10,50855024	10,53743702
20,22852231	10,52740271	10,58642195
20,3717247	10,52977862	10,63414837
20,46138715	10,55140769	10,66650622
20,52937142	10,60289231	11,29890952
20,54770485	10,61751432	11,30261177
20,69250824	10,62006359	11,33820272
20,80173675	10,68546721	11,33820272
20,80641868	10,70906271	11,41303997
20,85361022	10,73648867	11,4740975
20,85795942	10,79145613	11,51793021
20,89597737	10,80701843	11,66575457
20,89835183	10,90168109	11,93491743
20,98234814	10,95738994	12,02518406

21,11373791	11,00329566	12,02518406
21,13164086	11,01976846	12,18447952
21,36709605	11,0308342	12,19849212
21,42599577	11,0701782	12,19849212
21,6646797	11,20343155	12,33294583
21,67431414	11,25894261	12,33743267
21,78820371	11,30155275	12,33743267
21,84679045	11,30526268	12,41316157
22,00074262	11,33614973	12,41316157
22,13114444	11,34431679	12,43207601
22,3218923	11,43476384	12,51594073
22,82358843	11,43735945	12,72328996
22,8624639	11,44280132	12,76149343
23,38238123	11,52593884	12,90470638
23,41627387	11,73717273	12,99745588
23,43375115	11,79894045	13,0053488
23,43949931	11,80148332	13,0053488
23,49726091	11,87679877	13,02538429
23,53127075	11,90202335	13,08086157
23,71297916	11,93117515	13,08086157
23,90892487	11,96477635	13,13223281
23,92802048	11,98514676	13,14429738
23,93346805	11,99254647	13,23100632
24,00749351	11,99718067	13,25728818
24,04138156	12,0108369	13,33234772
24,07124744	12,05856037	13,36724935
24,109243	12,12720708	13,38943961
24,19115436	12,18518304	13,63053113
24,26527107	12,28086905	13,63238352
24,32947461	12,43966145	13,70531617
24,34820675	12,44724675	13,82620399
24,39455896	12,46327561	13,98928438
24,42375103	12,52169301	14,07492746
24,57097325	12,54171948	14,09112941
24,63646632	12,5440405	14,33784926
24,6910584	12,6038004	14,34983091
24,73938996	12,61032167	14,98174891
24,8196266	12,64658088	14,98174891
24,90338303	12,75048036	15,00169494
25,01529647	13,00680561	15,10266413
25,06756382	13,01159294	15,11871933
25,13345478	13,04772673	15,11871933
25,16135886	13,06632465	15,2844928
25,37227755	13,12876556	15,33425974
25,53900047	13,1368435	15,45800581
25,61243194	13,18333273	15,48657936
25,69668945	13,18435131	15,48657936
25,81995087	13,19698332	15,63144137
25,94799071	13,20867667	15,63144137
25,99309834	13,34694131	15,77483234

26,01733115	13,42997523	15,77483234
26,1101009	13,47013398	15,8210747
26,11591916	13,56761984	15,83043522
26,25796167	13,60268497	15,88301332
26,365665	13,64991545	15,89285604
26,44315897	13,65482259	15,93313187
26,4638824	13,68042179	15,95263577
26,55670814	13,68941429	15,95701673
26,56050839	13,80720411	16,00274609
26,65763424	13,81046959	16,01991233
26,68653018	13,81815313	16,01991233
26,72823276	13,95982014	16,04789411
26,7500369	13,97278152	16,09233231
26,82076651	13,97833411	16,12217019
26,92064331	14,00620715	16,25293574
26,93350208	14,12211278	16,25293574
27,12472838	14,23430056	16,32029356
27,28286355	14,23460673	16,45867903
27,32754522	14,5703016	16,5543013
27,38758661	14,66771333	16,5543013
27,49934296	14,67027986	16,61769681
27,52250376	14,71390698	17,03477162
27,79918795	14,74423478	17,10887034
27,91850165	14,80606588	17,21822148
28,0200037	14,81216059	17,27060705
28,03122797	14,82210083	17,42973361
28,22134848	14,83473023	17,44102953
28,40691838	14,92268335	17,47696567
28,49369226	14,96720098	17,49882051
28,55562574	14,98030959	17,5396051
28,6503339	15,00056788	17,5396051
28,73116658	15,0471817	17,55760532
28,92519786	15,04884027	17,61470997
29,15550274	15,07180957	17,62378035
29,16818364	15,07306919	17,64570054
29,19923223	15,11988516	17,65744426
29,49718417	15,12602847	17,72418072
29,62649265	15,22857445	17,72418072
29,97218077	15,30952414	17,80718792
30,10816697	15,38895531	17,82241831
30,11622733	15,4028625	17,85368098
30,11655811	15,51035465	17,85368098
30,13495714	15,52547188	17,86758666
30,15976237	15,53878858	17,89803982
30,39328932	15,54600425	17,94048798
30,55271433	15,58951677	17,94048798
30,64425996	15,64026448	17,96502154
30,80043703	15,64689848	18,04589073
30,84983684	15,77250067	18,04589073
30,86255218	15,78574052	18,05623527

30,86674164	15,7858166	18,17006918
31,00835901	15,83716182	18,21117248
31,2511926	15,84419579	18,21160407
31,26128356	15,84944142	18,32103825
31,28588018	15,86608096	18,32103825
31,31627138	15,88026942	18,38619145
31,53141457	15,92091231	18,51745389
31,59459548	15,93921814	18,51745389
31,7175254	15,98298977	18,5710683
31,94304182	16,04648653	18,60149698
31,95216141	16,06114277	18,77962873
32,04503302	16,24752188	18,85821581
32,07191459	16,29077234	18,95054681
32,12122796	16,29960783	19,02276979
32,1267918	16,30151216	19,0712222
32,13737159	16,3116588	19,12942675
32,14481542	16,31211094	19,12942675
32,19337012	16,33787794	19,14049666
32,41792899	16,35708266	19,20189255
32,41970779	16,36565526	19,22735979
32,48914718	16,42009683	19,28463834
32,69516199	16,42388404	19,40530993
32,77104784	16,48030839	19,43220224
32,85151736	16,56606361	19,44154679
32,87280667	16,57356046	19,44154679
32,89931737	16,59982073	19,45016834
33,04761401	16,65817397	19,52889648
33,05249917	16,73287912	19,52889648
33,12233391	16,83421385	19,536241
33,13299932	16,86048546	19,536241
33,17700615	16,92491823	19,59405389
33,1782843	16,92552593	19,62062875
33,54270885	17,06378078	19,62062875
33,68523611	17,11158642	19,65109633
33,71968486	17,11232874	19,67482109
33,80768824	17,28198315	19,93945899
33,87206285	17,32325956	19,95635563
33,91334408	17,39138934	20,06717526
33,98548298	17,44222838	20,06930568
34,13265104	17,44501288	20,06930568
34,14017318	17,47165364	20,1661859
34,29655735	17,49145838	20,24303752
34,30948957	17,69291692	20,29390057
34,33904515	17,78859306	20,30883775
34,38118263	17,81588769	20,434023
34,63262486	17,83510957	20,434023
34,72250063	17,84223547	20,5116218
34,80920055	17,85646356	20,60448332
34,92084165	18,08614847	20,65332686
34,94967515	18,10648631	20,71640166

34,96336491	18,18483923	20,71998949
34,99463356	18,19847152	20,71998949
34,99847318	18,27248965	20,8883494
35,01688187	18,38794553	20,88856485
35,14113118	18,40288634	20,9776971
35,22397652	18,41038943	21,06044105
35,40208561	18,43299968	21,0847519
35,67795844	18,44601075	21,32207436
35,68643846	18,45850044	21,34061974
35,80099555	18,53785001	21,35424315
36,04558156	18,57798902	21,35424315
36,06404604	18,77812088	21,37672495
36,07663982	18,79757443	21,37672495
36,37343467	18,83051308	21,384696
36,39370516	18,85689644	21,384696
36,44832152	18,89847367	21,41588812
36,53188471	18,95141802	21,45612543
36,56294139	18,99626359	21,52502636
36,68995292	19,10611555	21,53789748
36,69532762	19,12897269	21,53789748
36,7775332	19,16485714	21,56387573
36,79015656	19,29835012	21,70366495
36,8315176	19,33538089	21,70366495
36,90856906	19,33728281	21,88321748
36,91950589	19,36954406	21,99808946
36,92418772	19,37208009	22,04827909
36,99998939	19,37911463	22,07608393
37,04845358	19,38769446	22,13559743
37,08952935	19,39448999	22,23936549
37,12414795	19,43302298	22,63283078
37,20597707	19,50823884	22,65592341
37,22244094	19,53071817	22,69493196
37,25688044	19,5543314	22,76593572
37,36383141	19,59926024	22,78791778
37,5237398	19,61993536	22,93507499
37,58023651	19,74073877	23,05558067
37,58877542	19,77516901	23,16174573
37,67220128	19,78145578	23,2849428
37,67504097	19,78381097	23,28926593
37,69271625	19,79949147	23,30264461
37,72952068	19,85812489	23,37624917
37,82799189	20,05198528	23,44105112
38,00803826	20,08987236	23,44105112
38,04997947	20,14294941	23,47699801
38,05737917	20,18426746	23,48785679
38,11396044	20,19798899	23,48785679
38,24825694	20,20241835	23,70701605
38,2981323	20,23878238	23,70701605
38,4591773	20,24886731	23,71761796
38,52249703	20,25250529	23,81621777

38,68755331	20,27301414	23,82053905
38,87626782	20,32824697	23,82053905
38,9695677	20,33538468	23,98618249
39,09980589	20,34900626	24,01508003
39,11057549	20,47753491	24,02083455
39,1731637	20,57521058	24,02083455
39,24033598	20,60319437	24,11329176
39,25344336	20,6034761	24,11329176
39,39113584	20,62178491	24,1464408
39,40832655	20,63068308	24,27671811
39,44453592	20,64111295	24,39159658
39,49409143	20,67018954	24,49716909
39,51166761	20,67187525	24,55660267
39,651639	20,88128319	24,57822989
39,71006447	20,92677301	24,6133385
39,71486262	20,97836237	24,69325246
39,8071586	21,00624529	24,73694729
39,90552654	21,03051711	24,80067111
40,06825976	21,09514632	24,80067111
40,20687025	21,17246136	24,80914331
40,27240339	21,17681684	24,82449454
40,2865361	21,2317734	24,82449454
40,31413283	21,25802008	24,99031241
40,3293302	21,2720576	25,01952596
40,43059336	21,28058363	25,03850333
40,50923932	21,34921371	25,13537926
40,53680282	21,40375837	25,17922126
40,78598881	21,43421051	25,24362971
40,80166233	21,44192351	25,26042833
40,83831579	21,45137725	25,32507284
40,88177801	21,48940301	25,4368588
40,9747056	21,530384	25,46742884
41,29998006	21,56835904	25,4860445
41,34396764	21,59520743	25,48677903
41,48882164	21,60874994	25,52533811
41,58651948	21,63425368	25,59966626
41,83528444	21,66386301	25,61985109
41,98751771	21,67161627	25,61985109
42,10668085	21,69833319	25,63402896
42,22323271	21,70159881	25,6602511
42,22949318	21,7066023	25,66122321
42,23093515	21,71869576	25,67957294
42,26902991	21,7442547	25,67957294
42,36544875	21,76405231	25,71154351
42,46220423	21,79706398	25,7328889
42,50345918	21,87087135	25,73415265
42,57379393	21,95198105	25,76244666
42,60657521	21,97317212	25,77075955
42,84193051	21,97842171	25,79431405
42,9425936	21,9906732	25,7990197

43,05006929	22,00586953	25,7990197
43,07483515	22,0419161	25,82704432
43,17655551	22,06855358	25,87923034
43,21097802	22,10466549	25,87962248
43,215249	22,11485517	25,87962248
43,29173386	22,12878483	25,93269367
43,29245318	22,14892804	25,93798887
43,35311208	22,19032631	25,93798887
43,42039247	22,25040954	25,99443609
43,44331832	22,29608361	26,00566913
43,47376883	22,34564198	26,03367059
43,50979543	22,3556496	26,26696952
43,56204904	22,37002081	26,2984435
43,70951713	22,37744669	26,31816945
43,72825632	22,3825203	26,33915258
43,73708673	22,43076924	26,42529437
43,75863431	22,44384026	26,42529437
43,79472489	22,46692586	26,47981015
43,8727998	22,54168286	26,48563175
43,88748784	22,5629603	26,53272123
43,9403745	22,69853703	26,5972911
43,96191425	22,77554143	26,89080397
43,99430406	22,82991736	27,00354734
44,01164745	22,87819993	27,05135977
44,20548042	22,89470879	27,08560834
44,52326793	22,96490854	27,08560834
44,59985153	23,04684047	27,10218911
44,65499951	23,08775424	27,10695741
44,76221124	23,11410135	27,16615957
44,85325951	23,17945643	27,18413215
44,90235117	23,27958258	27,24045185
44,97430837	23,35665437	27,3009637
45,16773805	23,388219	27,43708713
45,20386659	23,43162046	27,49366473
45,44486103	23,44467539	27,49395762
45,5468484	23,52450769	27,57671575
45,57348814	23,57396703	27,58016646
45,59997875	23,59417248	27,60154637
45,62203874	23,62389826	27,66390101
45,76728881	23,69178126	27,71489031
45,80991432	23,70834202	27,72936352
45,87910317	23,71219043	27,73963193
45,90675365	23,76218682	27,87024001
45,92273025	23,76564841	28,01725493
46,01221022	23,85909853	28,01725493
46,22728847	23,86709615	28,13941207
46,24894211	23,91111296	28,14821123
46,32391049	23,95611777	28,1906543
46,35517709	23,96348522	28,1906543
46,44670948	24,00359647	28,21581197

46,49600187	24,00841212	28,23242726
46,51054353	24,01185611	28,23242726
46,52942931	24,09231347	28,28162403
46,6000008	24,14611291	28,30652787
46,67001084	24,17377778	28,31468354
46,85140289	24,24540252	28,32847893
46,89915825	24,27500177	28,37337321
46,95090966	24,31810902	28,39794874
47,02826256	24,31994336	28,39794874
47,24132592	24,35450663	28,39890238
47,333689	24,42978693	28,42370078
47,37193668	24,45584372	28,43750767
47,48057453	24,46198021	28,67356524
47,48259688	24,51428291	28,77633618
47,64148306	24,56752453	28,82258353
47,83157423	24,57996584	28,90630901
47,85660515	24,59403368	29,07053975
47,88052174	24,59836576	29,08141986
47,88259674	24,60310208	29,16502132
48,04095523	24,60469768	29,16502132
48,18941774	24,60486594	29,20063515
48,22416963	24,66192212	29,20439875
48,28118346	24,67117306	29,24148016
48,28138234	24,69260678	29,3140084
48,46111182	24,70487276	29,35909833
49,00320894	24,71006688	29,38230182
49,17380091	24,73420842	29,43200203
49,21574769	24,74398919	29,43496567
49,57792446	24,74402072	29,46316869
49,65927876	24,75250649	29,50329045
49,70990943	24,79956567	29,52553802
49,74128651	24,83345359	29,52553802
49,75697485	24,8360789	29,63129324
49,77310068	24,85443723	29,66549891
49,89061683	24,9561497	29,66704531
49,89076824	24,97179502	29,68316776
49,90699155	24,99647589	29,69981963
49,95683683	25,04951387	29,81296829
49,97075634	25,08064519	29,88099928
49,98344366	25,08318611	29,89463073
49,99034057	25,09706498	29,91085375
49,99104645	25,15719426	29,91085375
50,01507897	25,20739413	29,96679494
50,02729576	25,23944216	30,15410397
50,29598728	25,25625813	30,15410397
50,41682675	25,34821625	30,23111819
50,46430991	25,37574493	30,23111819
50,54514113	25,39138651	30,28399587
50,60202435	25,40694031	30,28399587
50,61572814	25,47611803	30,36892336

50,61648349	25,48469992	30,38264426
50,67478449	25,49543954	30,43722151
50,67533864	25,52669844	30,43722151
50,68784882	25,56473177	30,49427745
50,7105064	25,63141175	30,51224691
50,73052713	25,64065792	30,59130196
50,88541287	25,68485792	30,6460129
51,15152175	25,69728908	30,69163665
51,17879681	25,70406403	30,75180513
51,29236833	25,76609459	30,7670481
51,43941613	25,79909887	30,82566906
51,57870477	25,8390121	30,85883466
51,63763884	25,89563194	30,87998696
51,66394848	25,93476609	30,87998696
51,69123371	26,00476393	30,9254818
51,71922076	26,05949661	30,95676479
51,76840868	26,10595274	30,96106754
51,80390582	26,10889995	30,96106754
51,91996512	26,15144539	30,98897746
51,92188209	26,18340101	30,99393441
51,95880752	26,18982375	31,05934001
52,00800006	26,2351524	31,11038572
52,04452066	26,24336662	31,13016826
52,09789861	26,2989458	31,23295411
52,14499404	26,32023688	31,27763516
52,1497707	26,37649229	31,31454553
52,15799444	26,41931485	31,35028049
52,18189603	26,44566561	31,35610083
52,19174543	26,48537655	31,42222836
52,21687987	26,50820245	31,42222836
52,34718864	26,53035921	31,42250518
52,49025656	26,59180864	31,4251745
52,55746879	26,70874274	31,43419525
52,65972146	26,71499476	31,49735509
52,71353531	26,77669111	31,5362961
52,77603673	26,78539936	31,53701566
52,88773125	26,82445857	31,53701566
53,0051742	26,84945675	31,5886341
53,21723223	26,96496554	31,74431144
53,25439788	27,01773925	31,7478985
53,36925692	27,02799994	31,77681967
53,37266715	27,03368801	31,782866
53,37384734	27,03730587	31,8977389
53,39535184	27,0824026	32,07899009
53,55855945	27,09961149	32,0831796
53,55921306	27,10969622	32,0831796
53,59824112	27,22357123	32,08777418
53,61502851	27,23834327	32,08777418
53,64362817	27,25079051	32,12897973
53,67102905	27,25692007	32,1773901

53,67328939	27,37241003	32,1773901
53,88106781	27,42398438	32,18441912
53,88751848	27,59811428	32,20610113
54,10661294	27,60656368	32,21888947
54,20048124	27,63301281	32,21888947
54,2722277	27,6684819	32,31973588
54,32291517	27,7928817	32,41836015
54,37740733	27,81736209	32,44469561
54,44134112	27,8761878	32,67269292
54,59195328	27,92143766	32,67269292
54,60322512	27,95937255	32,72169384
54,69456249	28,02782871	32,74912743
54,70353898	28,03796923	32,86132359
55,05218592	28,04217538	32,90832751
55,39847392	28,06237478	33,06065159
55,45073983	28,11296289	33,0648537
55,46255673	28,1338915	33,30284962
55,49686574	28,19265233	33,31482685
55,56034085	28,21008412	33,38092425
55,64824865	28,21208146	33,42221192
55,6922879	28,21267818	33,44185694
55,76327767	28,27170983	33,44185694
55,81158924	28,27459616	33,48705698
55,85042248	28,30720122	33,56543416
55,94695828	28,36724532	33,56929944
56,03701309	28,38715377	33,61334793
56,04337771	28,40092546	33,67752378
56,05857463	28,40814599	33,67752378
56,18736051	28,44488693	33,6801948
56,21977292	28,48003936	33,77816567
56,42742166	28,50754671	33,80063693
56,45118435	28,52103918	33,9249628
56,45120009	28,69384672	34,00640038
56,55217295	28,73515861	34,01084199
56,55582771	28,75453431	34,01084199
56,74505338	28,76025554	34,08099925
56,76798664	28,78880932	34,08099925
56,79288975	28,81004295	34,08140148
56,83748718	28,87079066	34,12231399
56,92399746	28,87250501	34,20651573
57,04618941	28,90531543	34,25855353
57,06859099	29,00051777	34,27416672
57,07560421	29,09127329	34,41195921
57,12887146	29,17769164	34,48723656
57,17486689	29,19912022	34,55151712
57,35154789	29,23978638	34,59682922
57,35554984	29,25956703	34,59781644
57,39813993	29,27541659	34,62771123
57,49752272	29,35161356	34,63478308
57,53487999	29,39695686	34,69002417

57,57808732	29,43929377	34,76958847
57,6312217	29,46828119	34,80529623
57,79890233	29,47148901	34,81574855
57,8333254	29,49386613	34,85910556
57,84222148	29,58387518	34,89239642
57,9324303	29,60188137	34,94308037
57,96977306	29,63693032	35,05323894
57,98000422	29,66054487	35,08783017
58,02305616	29,6712287	35,17355598
58,0329483	29,67458775	35,19501796
58,04302759	29,6973651	35,23845293
58,08708816	29,75266662	35,23943571
58,15132764	29,89796741	35,34007364
58,3466897	29,9051345	35,37177024
58,50887523	29,96019596	35,45199775
58,77654314	30,0154719	35,47403326
58,84258149	30,0287101	35,55494398
59,00090379	30,11276064	35,55760223
59,08575136	30,15834044	35,55760223
59,09345702	30,15953703	35,5581561
59,09779887	30,26204212	35,59812508
59,13969983	30,29918071	35,59812508
59,17013879	30,35651837	35,62183915
59,18040776	30,40471448	35,70909566
59,25971371	30,44295667	35,85036642
59,2607261	30,44800307	35,90645309
59,2866692	30,47410545	35,93548071
59,29266152	30,53085475	35,95862431
59,37287026	30,55177427	35,982095
59,4254615	30,60012995	35,982095
59,60483989	30,63037768	36,15485471
59,60945635	30,64218415	36,1560159
59,72433869	30,68773728	36,1560159
59,72879387	30,68854166	36,17784879
59,78455001	30,74484295	36,21848608
59,80698775	30,78529406	36,32585825
59,88638495	30,83127864	36,35858569
60,05672881	30,87889573	36,36837227
60,19660958	30,88075073	36,36837227
60,22835155	30,92585269	36,39521012
60,27352072	30,94488144	36,39691502
60,35162722	31,02688525	36,39691502
60,35448324	31,07625711	36,41428756
60,42802578	31,08883908	36,42438123
60,61136048	31,08957634	36,4470417
60,61667949	31,09551478	36,52196094
60,63282701	31,09821764	36,52196094
60,7646564	31,12489138	36,59691023
60,80888198	31,15550137	36,61273944
60,85699308	31,18080038	36,7163497

60,91218361	31,20089233	36,73746819
60,9435489	31,22966816	36,86576996
61,00022938	31,23286243	36,88408361
61,09346618	31,24411848	36,96236467
61,17477082	31,25354809	36,98815534
61,20465467	31,35326653	36,99899016
61,23591639	31,35699912	37,04541073
61,24212388	31,39181083	37,05861069
61,29619226	31,40547743	37,06287215
61,30032684	31,43325736	37,19208602
61,47540147	31,43462062	37,23025354
61,50760923	31,4625086	37,25620309
61,60075753	31,56667216	37,25709689
61,71008639	31,6680872	37,26320854
61,75107696	31,67575227	37,2909687
61,82080383	31,67597141	37,34823501
61,86018508	31,67664351	37,35654699
61,91487061	31,68115569	37,48391354
61,97507995	31,74837871	37,57553137
62,01790406	31,76992431	37,71489939
62,0410051	31,80970124	37,71723334
62,14046794	31,82153351	37,73568571
62,16821867	31,86965048	37,80274929
62,23226528	31,92694973	37,88365438
62,24632638	31,94977217	37,9222834
62,38041707	31,97926141	37,99234664
62,46976549	32,02682325	38,02292726
62,50634464	32,04498316	38,04979947
62,72490603	32,05245674	38,09722098
62,79066389	32,07637926	38,13919595
62,92555612	32,08086113	38,17496367
62,98998768	32,09092671	38,24150658
63,03956881	32,18023468	38,24703233
63,07268141	32,29246938	38,27060273
63,22694762	32,31407579	38,27440312
63,2983462	32,34161511	38,27816771
63,36146189	32,40335157	38,29998689
63,40919096	32,55726197	38,32958478
63,52164268	32,57311577	38,34699591
63,52274917	32,58477841	38,44234698
63,63717517	32,58997019	38,50485219
63,66459672	32,65079499	38,52241361
63,72662535	32,72541905	38,53271472
63,88987765	32,74984282	38,53271472
63,96103908	32,75570264	38,54761137
64,01038592	32,80082993	38,54761137
64,04943788	32,85361401	38,58836665
64,07645223	32,96183411	38,66463772
64,10611592	32,98612547	38,75789453
64,22668952	33,00318713	38,80821483

64,28748319	33,01075477	38,80821483
64,40469155	33,02461764	38,82460809
64,62234487	33,02782818	38,93207179
64,65258527	33,03849759	38,96503389
64,72654738	33,06402167	39,12594151
64,76265624	33,07487942	39,14605024
64,86697099	33,08531403	39,2537761
64,96162221	33,15362084	39,28336867
64,98345239	33,18061035	39,32033955
65,00725693	33,1848683	39,394247
65,1090272	33,31202344	39,45645858
65,18337943	33,35511862	39,45645858
65,20654909	33,395834	39,71015662
65,32532577	33,40737206	39,73494692
65,32791531	33,44092546	39,73494692
65,47695217	33,46605906	39,74734182
65,57386539	33,61708078	39,83186725
65,6016609	33,61953658	39,87704182
65,63273797	33,63031095	39,93786366
65,7638099	33,69375791	39,98903956
65,77986777	33,69746835	39,99332563
65,83390209	33,69953327	39,99587841
65,98539678	33,70128943	40,15109665
66,03063958	33,72128115	40,22718604
66,08605231	33,81301192	40,24684647
66,14327251	33,89547456	40,33046998
66,3187545	33,91431755	40,41657667
66,32265696	33,92452643	40,44266432
66,38319299	33,93979072	40,50073273
66,42409932	33,9891551	40,50073273
66,47732822	34,0654712	40,53358903
66,55885877	34,06981936	40,53814226
66,65319657	34,1035017	40,55349191
66,76838988	34,10591958	40,90280259
66,91802264	34,13178915	40,92085795
66,9477011	34,16196391	40,9275041
67,01126491	34,1682417	40,93090311
67,28415313	34,21099141	40,94516308
67,30337008	34,21214675	40,9899193
67,32355662	34,22188215	40,99208113
67,6315388	34,24705567	41,01184856
67,6344346	34,2493706	41,0545102
67,90925601	34,25659888	41,0545102
68,05842541	34,26213817	41,17980486
68,08858735	34,27753631	41,1924792
68,10080617	34,32029358	41,22727358
68,10475849	34,32063162	41,33435756
68,10864428	34,37943237	41,34896468
68,30335503	34,41645366	41,34896468
68,4441403	34,44150826	41,3518608

68,45104651	34,44272838	41,39858953
68,55601749	34,51024646	41,43604778
68,56821092	34,51623744	41,51200028
68,61859094	34,52716108	41,53428034
68,66803117	34,56351832	41,57318013
68,75714212	34,62019637	41,5945664
68,86405247	34,681972	41,86197852
68,94497715	34,68884302	41,87503605
68,96131562	34,70430561	42,03419727
68,9694299	34,71613385	42,06655914
68,97105295	34,73894309	42,07775159
69,00521229	34,88384015	42,09413053
69,02112147	34,92949531	42,09413053
69,0627574	34,9608434	42,09847973
69,12062615	34,96793927	42,13567083
69,18637834	34,98771302	42,13567083
69,23372992	35,05914478	42,14737178
69,26768615	35,08945811	42,14998923
69,35110489	35,1149959	42,16354108
69,49791174	35,17331496	42,22944145
69,60143236	35,27909994	42,22944145
69,74457808	35,30518474	42,2383668
69,78543936	35,3174102	42,2383668
69,82234723	35,32546689	42,26326195
69,86777775	35,35949373	42,35379631
69,94581968	35,436989	42,38828483
70,04277849	35,46840442	42,39819293
70,0439172	35,47373385	42,39917332
70,33198006	35,51106384	42,44649617
70,41666781	35,52016822	42,51125987
70,51822781	35,73159015	42,56366244
70,52234509	35,81539084	42,59529289
70,71564315	35,85841671	42,63582801
70,94056459	35,93734029	42,79395147
71,05557246	35,95274969	42,79395147
71,06218926	36,03774404	42,80037832
71,06242752	36,0621366	42,80037832
71,07259911	36,10679225	42,82785689
71,1032107	36,12322357	42,8311257
71,26925205	36,12387935	42,97339505
71,28789303	36,16444967	42,97772478
71,37882711	36,16564157	43,01092535
71,50521169	36,18863549	43,01122596
71,57577792	36,20920007	43,03243055
71,59571847	36,28642456	43,03264396
71,61525463	36,31485539	43,04242567
71,77693212	36,32370805	43,04865075
71,79469129	36,33120299	43,09814413
71,97912444	36,3870564	43,09814413
71,9794863	36,43337442	43,13157965

71,99636202	36,46185027	43,22043927
72,00930484	36,62335464	43,34849307
72,01384325	36,65162939	43,34849307
72,09459411	36,70350545	43,35049456
72,09747205	36,71609121	43,40619383
72,34173504	36,74797192	43,40770936
72,34807366	36,76323932	43,44427487
72,42197677	36,77121802	43,62734392
72,47778877	36,78823765	43,63040548
72,56399555	36,82985306	43,63040548
72,61015353	36,84605172	43,6415645
72,68671899	36,85432539	43,67444172
72,73487584	36,85962232	43,73207051
72,7490113	36,88603584	43,73207051
72,76199298	36,93010367	43,78153078
72,777829	36,94427804	43,87582226
72,81556338	36,97663859	43,88013367
72,89420812	37,00731245	43,88297028
72,96829064	37,06025567	43,88297028
73,06503732	37,06182759	43,91124323
73,20474	37,0758791	43,91124323
73,21313228	37,16403146	43,92752442
73,2644502	37,17414015	43,96688123
73,28720908	37,20282261	43,96772187
73,43799636	37,24826267	43,97148102
73,49357467	37,28324149	44,00830823
73,57355176	37,31644956	44,03538369
73,72331344	37,36532262	44,08575868
73,74660947	37,38702415	44,13777356
73,78451508	37,40200083	44,13809593
73,87598977	37,41802614	44,20061342
73,94496681	37,41926477	44,2524279
73,9532334	37,44205243	44,25455908
74,01058806	37,45123162	44,27084794
74,02726723	37,48790876	44,28130121
74,03822784	37,53691646	44,37605431
74,26134505	37,56028314	44,40789541
74,34661718	37,56304897	44,4129095
74,37488275	37,57487398	44,47306432
74,38158819	37,73895275	44,48009588
74,48138774	37,75367276	44,4916642
74,49376402	37,75381202	44,59873236
74,60959092	37,76891051	44,62983909
74,62076971	37,81866288	44,66094957
74,67363794	37,81968821	44,66094957
74,67961681	37,83064504	44,68167205
74,72117603	37,83479667	44,7494027
74,87092391	37,83494489	44,85671142
74,92393972	37,83509171	44,94623025
74,94237589	37,91179992	44,94623025

75,00717099	37,9495371	45,10125173
75,01984877	37,98188999	45,10125173
75,05621623	38,02431047	45,12134502
75,1856314	38,0885587	45,12565272
75,21391333	38,10749279	45,14565968
75,26968588	38,12369492	45,14752423
75,35561555	38,12847564	45,14752423
75,379184	38,13709332	45,20910576
75,43149726	38,1553602	45,21417787
75,47198117	38,15751595	45,35305437
75,50275854	38,19524157	45,3538199
75,59665173	38,24658472	45,37877795
75,62487137	38,27649479	45,39566883
75,66681475	38,2835355	45,4581766
75,71494469	38,35285971	45,4581766
75,77920239	38,36991453	45,46976565
75,79829839	38,38857325	45,4817622
75,87616209	38,40090722	45,58570514
75,88596491	38,40368295	45,7466549
75,96090028	38,50914328	45,78709128
76,01684914	38,51546714	45,78759386
76,15267783	38,52311518	45,8258461
76,18895696	38,60375836	45,8258461
76,25776403	38,62563756	45,89851083
76,25934081	38,69545743	45,91428376
76,31045243	38,70589196	45,93549904
76,31819307	38,73827484	45,95990517
76,46756443	38,75752237	45,99116885
76,51901022	38,77299452	46,04130201
76,61856963	38,83825235	46,04167444
76,688427	38,87734116	46,04167444
76,77904977	38,89359496	46,04185915
76,82426865	38,91166679	46,06078947
76,91798523	38,92132454	46,06078947
76,94074422	38,93400715	46,06509273
77,01405342	38,98900372	46,07532746
77,14482802	38,99167178	46,09725087
77,16567892	39,00860017	46,22543261
77,22323067	39,02179197	46,22543261
77,32666238	39,03391256	46,25768337
77,3273584	39,12789567	46,2636075
77,45305771	39,15119771	46,40531526
77,48291925	39,20315121	46,40531526
77,78572316	39,22769388	46,41806097
77,82216926	39,24668163	46,45439131
77,92829092	39,26803838	46,47888648
77,93809316	39,30772016	46,61245175
78,05973677	39,3275787	46,62850142
78,07984762	39,33223875	46,62850142
78,31886653	39,36753755	46,68089752

78,35937044	39,37807188	46,69949556
78,36630326	39,38870166	46,73390485
78,48715803	39,39331184	46,77015846
78,49472657	39,40139772	46,77059049
78,53722396	39,46400336	46,80899002
78,56179345	39,48111103	46,81217621
78,63661566	39,51261393	46,82154491
78,65387968	39,53213053	46,83503532
78,81980497	39,54613688	46,83598909
78,89826073	39,54888203	46,83598909
78,94305998	39,57715544	46,83938025
79,05496078	39,61283568	46,83938025
79,09997547	39,6185676	46,86484358
79,12650719	39,6397904	46,91210368
79,15651387	39,64561499	46,91559401
79,19757046	39,650293	46,93252633
79,22376524	39,73083035	46,9552384
79,24256033	39,73332317	46,99228104
79,28067173	39,75833807	47,00592939
79,34932666	39,90742497	47,00592939
79,36447376	39,97537855	47,08133644
79,76146794	39,97921953	47,09057442
79,89964232	40,04043996	47,09057442
80,13756684	40,06250044	47,09830954
80,16763508	40,07376094	47,17088557
80,19941233	40,09379394	47,220769
80,22295747	40,16711277	47,24585481
80,23506973	40,19121544	47,24585481
80,2836238	40,19514118	47,25353406
80,35378734	40,22880905	47,26541085
80,3851243	40,23060594	47,30430329
80,43896909	40,28222302	47,38908317
80,51079714	40,28422855	47,38908317
80,54980592	40,36632945	47,52802455
80,60751491	40,37630185	47,57129605
80,61539115	40,38704903	47,60335269
80,61915223	40,46847642	47,61830501
80,75624582	40,49429219	47,61830501
80,99599218	40,52380869	47,66661126
81,05587892	40,54594739	47,66661126
81,11650896	40,55474208	47,67656575
81,12131827	40,57930593	47,71280905
81,21620717	40,59145051	47,74623382
81,28439982	40,59234153	47,7575668
81,32447798	40,59973498	47,81312094
81,32844549	40,60124592	47,99847681
81,37386544	40,65747275	48,05247114
81,41133927	40,6941279	48,0532129
81,53383022	40,75334541	48,0532129
81,68717821	40,76684843	48,08795857

81,8730212	40,7728907	48,09745532
81,88709982	40,77531573	48,09745532
81,94885609	40,77939429	48,10969098
81,98569049	40,79185657	48,14544855
81,99624436	40,80779993	48,20524998
82,11215925	40,81117173	48,24449748
82,1385884	40,82300991	48,4095358
82,14030132	40,85901354	48,4817893
82,22701487	40,86525947	48,53306245
82,26323228	40,9477965	48,59735299
82,45325406	40,97494725	48,59735299
82,4743352	40,98538182	48,60994073
82,6497605	40,99001704	48,62322117
82,65183226	40,99447199	48,66602469
82,706681	41,09676511	48,67905985
82,71123215	41,15435882	48,70897799
82,90684995	41,15905774	48,84731153
82,913489	41,17173591	48,84731153
82,92350808	41,17399303	48,85299822
83,03876117	41,24333952	48,88512974
83,04182206	41,26507267	48,89115717
83,08330604	41,26901058	48,94638874
83,12626894	41,36570842	49,01150359
83,21991911	41,38416186	49,02695453
83,28695637	41,40799548	49,04153449
83,38388814	41,41844511	49,0654251
83,4156328	41,44778438	49,0654251
83,41763451	41,4650066	49,06647014
83,43330681	41,48419249	49,29206668
83,4825357	41,49486781	49,33125703
83,77714982	41,54702232	49,3321538
84,09917433	41,5546721	49,35083329
84,17587732	41,60702129	49,36259933
84,21450037	41,61668988	49,42211255
84,26479458	41,62290245	49,42211255
84,31748527	41,65361092	49,43253035
84,3180408	41,65423735	49,4814564
84,44992667	41,66418626	49,54646882
84,58070991	41,67237435	49,56385275
84,62574792	41,73330844	49,57868088
84,68665381	41,77179933	49,57868088
84,70041276	41,78448034	49,61356883
84,70991972	41,81150086	49,62437513
84,77091584	41,87120805	49,62557571
85,07586609	41,87938202	49,63000632
85,08809456	41,89552117	49,63000632
85,10954665	41,91182755	49,65336765
85,16423033	41,91382193	49,73046978
85,16516194	41,94130701	49,79655414
85,33348735	41,95994934	49,79655414

85,3502742	42,00830697	49,80524457
85,4684869	42,03845066	49,80806635
85,56127466	42,05046644	49,82258565
85,56189905	42,06361779	49,82258565
85,57968288	42,08318103	49,86700214
85,5978967	42,09499382	49,86700214
85,64246348	42,15854191	49,88363353
85,67261729	42,16176833	49,88777761
85,67573426	42,18190829	49,9052908
85,73840922	42,19263437	49,90914085
85,77921903	42,21575689	49,9124559
85,90340407	42,22533543	49,93648821
86,04846749	42,30893298	49,95515519
86,05164713	42,31940255	49,97341846
86,1168951	42,32348847	49,98836171
86,15312075	42,40046346	50,02765145
86,24890792	42,4370341	50,03203948
86,2702214	42,44258236	50,17931802
86,45766416	42,44282796	50,17931802
86,51581399	42,48879851	50,18142609
86,51685368	42,50198654	50,18189072
86,55599157	42,50577942	50,18189072
86,63926197	42,51547053	50,2135026
86,68118363	42,53539751	50,2159548
86,82285221	42,55197376	50,21998902
86,93570162	42,56789014	50,26516922
86,95170206	42,5849863	50,28039799
87,23397247	42,59220399	50,31921958
87,62555212	42,62333259	50,34770617
87,72752618	42,66378998	50,34770617
87,76351952	42,75884454	50,35622761
87,81971301	42,78905905	50,36615974
87,87297824	42,8330147	50,39398747
87,95954235	42,85576537	50,40081859
87,99138595	42,86830592	50,43553809
88,17136755	42,86914033	50,44822899
88,20120259	42,90469132	50,45666889
88,20928397	42,93808931	50,47161746
88,29627386	42,95345327	50,47161746
88,56924427	42,985278	50,47584955
88,67194915	42,99537483	50,48484732
88,69918272	43,02459944	50,48921084
88,75966492	43,08298313	50,51853088
88,77629212	43,1349534	50,60945043
88,82973148	43,15110903	50,64628439
88,83733719	43,15815548	50,69467153
88,89744446	43,16301339	50,69467153
88,94398071	43,17427113	50,72719322
88,96480267	43,18009225	50,75452757
89,0327235	43,18659976	50,7776456

89,17069195	43,19053959	50,7776456
89,30022798	43,19742397	50,79670277
89,30290637	43,21201063	50,79670277
89,30587596	43,21603176	50,84793613
89,33273693	43,25745147	50,87966481
89,39334739	43,25768702	50,89227032
89,40559964	43,32602624	50,89227032
89,54883418	43,37524914	50,90252612
89,56428444	43,38182894	50,97965538
89,58036451	43,42203638	50,99148828
89,69567036	43,43324857	50,99148828
89,69639564	43,4991787	51,03046833
89,79724618	43,50042774	51,03046833
89,84335651	43,51308931	51,03956129
89,92624334	43,52506218	51,05259153
	43,53636611	51,10151224
	43,55413501	51,11228942
	43,59977789	51,18493288
	43,60240369	51,19822334
	43,63672402	51,19822334
	43,64792761	51,2150047
	43,65048493	51,23181932
	43,66046307	51,26598053
	43,67091936	51,26598053
	43,70677321	51,29006736
	43,71267247	51,29153429
	43,75947091	51,29153429
	43,76438729	51,35628204
	43,7698636	51,4260426
	43,85698598	51,45319571
	43,88311545	51,45319571
	43,9608221	51,47034001
	43,97988202	51,51632183
	44,00343533	51,52569618
	44,01942579	51,57339617
	44,08028451	51,63158369
	44,08511578	51,65169825
	44,10576307	51,66758707
	44,13767329	51,68462249
	44,14479552	51,6922428
	44,17642051	51,76835254
	44,19194899	51,77548823
	44,21982623	51,84385513
	44,22751192	51,87466578
	44,23252717	51,90497039
	44,24249768	51,90497039
	44,24440718	51,95907483
	44,25498787	51,9873324
	44,29874851	51,99331201
	44,31942758	52,14631312

44,35343341	52,18852293
44,35597631	52,20309544
44,3587296	52,2062313
44,41223029	52,20891081
44,42440971	52,24872507
44,42469942	52,26624685
44,42749422	52,26624685
44,46284859	52,27178362
44,48979126	52,27178362
44,49081565	52,3199462
44,49083721	52,36386722
44,49358442	52,36386722
44,49445694	52,36642995
44,50012813	52,36642995
44,52556671	52,37575418
44,54443173	52,41204011
44,55432049	52,43695025
44,63363706	52,46566735
44,64689024	52,47901068
44,65750037	52,47901068
44,68976623	52,49341218
44,70375074	52,51362022
44,78309752	52,52185279
44,79765844	52,52185279
44,81488033	52,52557424
44,82569501	52,54166187
44,8378122	52,54503682
44,84111885	52,66528262
44,89649268	52,68216773
44,89792061	52,71739355
44,93065629	52,71739355
44,93305959	52,72185236
44,96758261	52,73748022
44,98207107	52,77705125
45,0135132	52,78182353
45,01388324	52,78182353
45,03171788	52,84817551
45,05189245	52,85799481
45,07366439	52,85799481
45,0887598	52,8621614
45,09704925	52,9084095
45,1748941	52,91068925
45,18619167	52,91201091
45,19860343	52,9211846
45,20319464	52,93009986
45,22845351	52,93932304
45,22848714	52,94989841
45,23188875	52,94989841
45,24034891	52,95437932
45,25907604	52,95437932

45,29862809	53,10937501
45,31220009	53,11889451
45,35339199	53,14000408
45,35914597	53,18472367
45,35924264	53,25597556
45,37428275	53,26537854
45,38878125	53,27213798
45,38894226	53,27213798
45,42573331	53,29329229
45,44221341	53,366878
45,44311402	53,37538182
45,46581827	53,39668817
45,47622573	53,41105139
45,49860316	53,44196568
45,54227737	53,46255322
45,5472063	53,46255322
45,54851721	53,47046668
45,59263021	53,49126587
45,59605944	53,51461945
45,61131647	53,52230039
45,62361741	53,53170933
45,73000243	53,54483064
45,77130758	53,5885046
45,80297194	53,59807532
45,8433411	53,59807532
45,88781156	53,62922055
45,89343337	53,63057878
45,90271416	53,71315881
45,99566609	53,85047833
46,05373784	53,90784456
46,05929785	53,90784456
46,06639999	53,9358172
46,11962378	53,9358172
46,12600487	53,97707549
46,17035751	54,00317004
46,18404708	54,00317004
46,18626332	54,01292328
46,1949787	54,01292328
46,19729126	54,0457095
46,20994215	54,09339446
46,21546515	54,09339446
46,22124799	54,11271115
46,22268277	54,13237494
46,22393555	54,16491188
46,25852309	54,17143392
46,26764098	54,18421086
46,3775693	54,1981284
46,39748742	54,1981284
46,39935315	54,20620453
46,40236489	54,21481391

46,46209938	54,21481391
46,48857417	54,21875217
46,4961983	54,2334785
46,51720873	54,2438884
46,53720815	54,24442402
46,53909813	54,24635088
46,53920955	54,2725364
46,54302505	54,33038553
46,57631218	54,33038553
46,58967454	54,33163008
46,63784499	54,35059075
46,65412793	54,35188666
46,65436905	54,47494921
46,67524465	54,56342001
46,67683762	54,58657864
46,68687537	54,58657864
46,69717392	54,60113175
46,69799613	54,64527427
46,72785851	54,64622438
46,73059856	54,65139404
46,78205673	54,68676303
46,78622056	54,7007145
46,79076555	54,7007145
46,79137735	54,72248279
46,80689944	54,73231668
46,82595825	54,81044063
46,84307383	54,81536235
46,84579299	54,84982278
46,8585161	54,84982278
46,86584186	54,86089975
46,87140691	54,87088285
46,89182909	54,87344794
46,91717564	55,11412089
46,92648073	55,11606829
46,98423725	55,11606829
47,03462061	55,15549463
47,07788078	55,16388242
47,08893174	55,16388242
47,09627505	55,16618495
47,11213615	55,18453083
47,15690049	55,27401976
47,17442739	55,27465472
47,19348995	55,27788732
47,19459687	55,295468
47,19825611	55,41753385
47,254782	55,4394875
47,31405083	55,4394875
47,33426623	55,45906371
47,36649891	55,46046778
47,40810201	55,46046778

47,42655432	55,47440534
47,45295966	55,48757766
47,4626519	55,49927255
47,47517376	55,49927255
47,47556631	55,52379225
47,50630456	55,55656173
47,51683185	55,59637809
47,5382217	55,59727768
47,54462569	55,62125097
47,55059355	55,62125097
47,55582418	55,64113888
47,61016342	55,67746916
47,61341615	55,68937984
47,61485328	55,68937984
47,63836127	55,72325385
47,66219904	55,72325385
47,67376608	55,73102536
47,67717493	55,74687556
47,67993679	55,77231063
47,727386	55,80673471
47,76844379	55,8254284
47,7770869	55,84363777
47,78621385	55,90664761
47,8150156	55,91645134
47,81647141	55,94266047
47,84455916	55,96614098
47,85045306	56,05585765
47,85889704	56,0573035
47,86670081	56,07281018
47,87046149	56,07638087
47,89953249	56,07804094
47,9259772	56,08146134
47,94343371	56,09090666
47,94390547	56,11469592
47,9516117	56,12356795
47,96944407	56,13313786
47,98430062	56,13378565
47,99520619	56,13506187
48,00665957	56,18523595
48,05438397	56,21006167
48,05700133	56,24401287
48,05948728	56,29099095
48,06301848	56,29567414
48,07957962	56,3055311
48,09504954	56,36102192
48,12963228	56,36226484
48,14813317	56,37810811
48,15617809	56,38087786
48,17722686	56,38824172
48,19393049	56,39970466

48,20241437	56,40079021
48,21333338	56,40743082
48,21945208	56,42188922
48,22046079	56,42565536
48,24255643	56,42801133
48,25309069	56,44316897
48,27906484	56,45786327
48,3223624	56,57065323
48,32683887	56,57065323
48,37786582	56,58804431
48,42321709	56,58804431
48,44143277	56,60043557
48,4552623	56,70003119
48,46901006	56,71056747
48,47629758	56,71056747
48,47776167	56,71503679
48,48441666	56,76553102
48,49473236	56,77958409
48,50103291	56,83424245
48,50737645	56,83636325
48,51883922	56,89261847
48,53459144	56,89261847
48,54707106	56,96274282
48,55363585	56,96464768
48,55626423	56,97053129
48,55859591	57,02055457
48,6039079	57,03741979
48,60495122	57,04475295
48,61553718	57,08125886
48,64024301	57,0857404
48,65087952	57,10469216
48,6524036	57,20061422
48,65760178	57,20637092
48,68991292	57,20637092
48,69726706	57,22789234
48,72147971	57,24067128
48,73388452	57,24067128
48,73622005	57,24141855
48,74876413	57,25028076
48,75893874	57,35541692
48,77171482	57,35579751
48,78289537	57,38389332
48,79025552	57,39896229
48,7906434	57,40188697
48,82404654	57,41009236
48,82717775	57,41009236
48,84525244	57,43985953
48,86784772	57,43985953
48,87989071	57,45408608
48,88761199	57,45408608

48,92425002	57,46162067
48,93279274	57,48923933
48,95506462	57,49549904
48,96783538	57,49549904
48,98104414	57,51759303
48,98689397	57,52924888
49,00366381	57,53664402
49,01324792	57,54668561
49,03271867	57,56935083
49,08092423	57,61168246
49,10001396	57,64161413
49,12130151	57,73296893
49,12146921	57,75660742
49,1405008	57,78117935
49,15897271	57,78117935
49,18099771	57,78869512
49,21860774	57,81932872
49,24978806	57,82970628
49,25363729	57,82970628
49,27261567	57,84032678
49,31874917	57,8631322
49,36755036	57,88148644
49,37184562	57,89089805
49,40040193	57,92846503
49,41065146	57,92868828
49,41326086	57,92868828
49,42188622	57,95525843
49,45248681	57,98569184
49,45358845	58,01262144
49,48690623	58,0153735
49,49176922	58,02366073
49,50243863	58,02366073
49,50923804	58,03540905
49,51087245	58,03862592
49,54011696	58,03862592
49,55817796	58,07075136
49,61667471	58,07075136
49,62096828	58,15186735
49,63138847	58,17291842
49,69632156	58,1734594
49,69952079	58,21561079
49,72402008	58,21561079
49,73382179	58,25104892
49,74811161	58,27199416
49,75564564	58,30239261
49,75782352	58,31926902
49,76327477	58,32111948
49,76765692	58,32111948
49,77151649	58,3323638
49,77651202	58,33262422

49,81438795	58,33262422
49,85723764	58,34984734
49,8846518	58,3752153
49,88472186	58,37828196
49,88591956	58,39009291
49,92728265	58,41090971
49,92732151	58,42674964
49,94788545	58,45299487
49,95578885	58,49512059
50,03447052	58,49648729
50,05305227	58,52946906
50,07873269	58,61102777
50,12908231	58,61102777
50,13436949	58,62064764
50,15569945	58,62064764
50,19023209	58,62846286
50,24542426	58,66814391
50,24548377	58,66814391
50,27895671	58,67650143
50,28938335	58,72058271
50,29290072	58,72382189
50,30747101	58,72705267
50,3287442	58,73709727
50,337503	58,73952231
50,37264335	58,73952231
50,38005633	58,74329663
50,38801786	58,74598956
50,41476853	58,7465587
50,43230713	58,77870209
50,46341295	58,81055378
50,50906623	58,8186375
50,54223055	58,8186375
50,59811371	58,82070045
50,62749034	58,83083344
50,62856245	58,83083344
50,64743435	58,84528622
50,64892607	58,86137479
50,71648249	58,86137479
50,73762536	58,87888203
50,7530515	58,87888203
50,76873489	58,87947195
50,7785234	58,91558446
50,81304323	58,91558446
50,82114594	58,92321565
50,88121555	58,93388841
50,9034307	58,95576416
50,94559231	58,97054558
50,95127455	58,99233759
50,95844465	58,99233759
50,97656778	58,99474604

51,01027075	58,99474604
51,01729218	59,00817988
51,02862165	59,06710039
51,04597406	59,06774092
51,06124314	59,15810781
51,06639696	59,15810781
51,08722888	59,16247615
51,10166246	59,16646863
51,10248953	59,16646863
51,1100326	59,16819031
51,12159412	59,17550173
51,12268465	59,21162329
51,13803089	59,23612826
51,15319129	59,24000649
51,1535228	59,24523248
51,21008086	59,26544817
51,21223343	59,30376611
51,22617004	59,30539565
51,24065177	59,30984423
51,24134425	59,32908174
51,24843644	59,32908174
51,25867643	59,33537028
51,27966134	59,33537028
51,36444963	59,3662058
51,41386023	59,38736239
51,44207207	59,38736239
51,45913837	59,4018071
51,46688747	59,4138939
51,49323631	59,45075555
51,49830695	59,46643664
51,49880794	59,49692948
51,50879396	59,49692948
51,51088235	59,56050959
51,63853539	59,56050959
51,67081308	59,56055516
51,67106709	59,58358183
51,69069486	59,58830122
51,71630809	59,61473428
51,71637806	59,61473428
51,7172334	59,64950262
51,72207194	59,67142855
51,72467527	59,69563765
51,7320827	59,70249821
51,78293494	59,80320825
51,7979549	59,80586964
51,83654546	59,82106182
51,8540848	59,82224955
51,87491901	59,82643756
51,87501649	59,82873696
51,892537	59,82919168

51,9356243	59,83110177
51,93750011	59,85273435
51,95204285	59,89342628
51,96372832	59,89342628
51,97152668	59,91318955
51,98953288	59,91318955
52,00656513	59,91967784
52,0299813	59,92296597
52,04164795	59,92884872
52,05134444	59,93880807
52,06462308	59,96690037
52,06519269	59,99637368
52,06593355	60,00267196
52,07949056	60,00710773
52,14347125	60,00849785
52,14746908	60,03524221
52,15379006	60,0479735
52,15382182	60,05082609
52,16361543	60,13000553
52,1848404	60,13847933
52,2024073	60,15600538
52,20648297	60,1567235
52,22409462	60,16388954
52,22804154	60,17359012
52,22878584	60,18109551
52,23902216	60,20706944
52,25383125	60,2605742
52,2943919	60,28341473
52,29761637	60,30306143
52,31261194	60,30615385
52,34186408	60,30923028
52,34200954	60,32023987
52,36124563	60,3411814
52,36217487	60,34698515
52,37135934	60,36104836
52,39237769	60,36345968
52,40563663	60,36345968
52,42430833	60,37722699
52,45401812	60,37839076
52,47585045	60,39322178
52,50328853	60,39601672
52,51738529	60,4102131
52,52965908	60,46908467
52,5438611	60,46908467
52,54396342	60,50279943
52,56146458	60,50279943
52,57533248	60,51418225
52,60420354	60,52890304
52,60850571	60,52988527
52,65832876	60,52988527

52,70020485	60,56841744
52,71544658	60,56841744
52,72245491	60,57449823
52,72805997	60,58889006
52,74198322	60,60319296
52,74581496	60,60776928
52,78196107	60,6137898
52,8358566	60,63961009
52,89000172	60,64030742
52,901919	60,64229973
52,92543748	60,65422147
52,92798286	60,66590014
52,94388557	60,66786048
52,95091195	60,67088978
52,95242431	60,67522381
52,96293343	60,68535438
52,96689827	60,70667924
52,97735323	60,73138869
52,99734962	60,73803275
53,00169106	60,74073124
53,04493845	60,74073124
53,07031633	60,7505539
53,08296128	60,75352252
53,08598315	60,77046218
53,12104147	60,77046218
53,12935162	60,7762728
53,1464469	60,7762728
53,15025484	60,77878289
53,16071867	60,80582127
53,18769053	60,80582127
53,20037341	60,8067904
53,21199625	60,8067904
53,22384301	60,82951442
53,23259885	60,85902767
53,24124623	60,86534623
53,24504226	60,87114129
53,24652909	60,87114129
53,2675579	60,94796531
53,30051134	60,95288174
53,31613832	60,95965053
53,31705222	60,95965053
53,32625042	60,98853649
53,3349612	60,99066641
53,35608028	60,99725588
53,39795483	61,03160084
53,41034046	61,08045017
53,44687325	61,08045017
53,44738967	61,11402638
53,45424248	61,15423177
53,47967341	61,2587432

53,48647488	61,28513166
53,49939113	61,28988223
53,51665886	61,28988223
53,52332823	61,29221245
53,58290836	61,3133811
53,59619753	61,31769422
53,61152488	61,32353931
53,62802106	61,32707607
53,62989604	61,33160697
53,63024449	61,36192941
53,63189457	61,38601182
53,63881	61,39395513
53,65424044	61,41933706
53,65830656	61,4258899
53,66040337	61,46054808
53,66379305	61,47522033
53,6640605	61,48313014
53,6651291	61,48988345
53,6835904	61,50231957
53,6949283	61,50231957
53,74604283	61,51668563
53,75708043	61,52177443
53,79791874	61,52579977
53,79850364	61,52667139
53,83089968	61,52917273
53,86440133	61,52917273
53,87319696	61,57340405
53,89650882	61,61257058
53,91139258	61,61274223
53,91813782	61,61274223
53,93635696	61,62102702
53,98858118	61,62872367
53,99380355	61,64717996
54,00436946	61,66448979
54,01313123	61,66448979
54,02498993	61,66669127
54,03425664	61,66895151
54,03617506	61,67444098
54,05176877	61,67845815
54,10430519	61,68445848
54,11186179	61,68695187
54,12444805	61,70460564
54,16303112	61,70478507
54,19709787	61,70478507
54,19910907	61,70845911
54,21498009	61,71203855
54,22399805	61,71381412
54,25208496	61,73526898
54,27600962	61,7422211
54,27919204	61,75447163

54,27949471	61,75447163
54,2828896	61,81013713
54,2894617	61,81088084
54,29695907	61,81088084
54,30138708	61,81363311
54,32787267	61,81635015
54,35313819	61,82018352
54,38908776	61,84562671
54,39159773	61,84562671
54,41574132	61,84827288
54,42709573	61,85557327
54,45177087	61,86644706
54,45577927	61,91516983
54,46414061	61,91516983
54,49504894	61,9324711
54,52745179	61,93957708
54,53842612	61,94977376
54,54280911	61,94977376
54,56536471	61,9536344
54,56949792	61,95574674
54,57545417	61,96679296
54,58213004	61,98607449
54,61159642	61,99670462
54,62364215	62,02210163
54,62436619	62,03943576
54,6269652	62,06148931
54,64403257	62,0688364
54,6515016	62,11322245
54,65717272	62,1200853
54,6762708	62,13424335
54,69330547	62,15195992
54,69729113	62,15226925
54,70118244	62,15226925
54,70859339	62,16289649
54,71682532	62,17014608
54,72145819	62,21395177
54,73345998	62,21653701
54,79562547	62,21678202
54,82444731	62,27544612
54,85155787	62,33072671
54,86407422	62,34459003
54,86595634	62,35340133
54,88790733	62,40455104
54,88797842	62,41434465
54,90040934	62,42462381
54,93749705	62,42531741
54,96977797	62,44364929
54,98271878	62,44364929
55,00344185	62,45837077
55,06656877	62,45837077

55,06735802	62,49096022
55,0701148	62,5081724
55,08012606	62,52492151
55,08832121	62,55215351
55,0920543	62,56837842
55,09607482	62,59312824
55,0975687	62,59976478
55,10191626	62,64082712
55,11682928	62,64092245
55,12228718	62,66250932
55,13199182	62,66250932
55,13804352	62,66800539
55,14625427	62,70224773
55,15471701	62,72944687
55,22341097	62,73322231
55,2305926	62,76405984
55,23848135	62,76405984
55,25112203	62,79472759
55,25955814	62,79581024
55,26856331	62,81394054
55,29049719	62,8304609
55,29598242	62,8304609
55,30009185	62,83625074
55,30787932	62,8422931
55,33244556	62,8422931
55,33574867	62,84317777
55,34730177	62,84317777
55,34947391	62,84988228
55,36473822	62,85454867
55,37598151	62,90120238
55,39377857	62,90969191
55,40219122	62,91858187
55,40227586	62,94901313
55,44701106	62,9540168
55,44792592	62,97035537
55,46157516	63,00591269
55,48567863	63,0073239
55,50906061	63,02311221
55,52574462	63,02311221
55,54147759	63,06560243
55,57624395	63,06724477
55,61836272	63,06724477
55,62107187	63,06840155
55,65611976	63,07397062
55,65825877	63,07922297
55,66098015	63,07922297
55,6671104	63,08550471
55,71361893	63,0911456
55,71719955	63,09508002
55,72022866	63,11046968

55,74551035	63,11063329
55,75073447	63,13033056
55,75260251	63,14886141
55,75983622	63,15143431
55,76961936	63,16700334
55,78757221	63,18702131
55,81053461	63,19331412
55,83771576	63,19331412
55,84621688	63,20817623
55,85047634	63,20817623
55,88804543	63,21987582
55,90885309	63,23893513
55,92159695	63,24748044
55,93582461	63,24835438
55,94727953	63,24835438
55,95417311	63,25146588
55,97096771	63,26194282
55,9929222	63,27320795
56,01999393	63,27320795
56,0222854	63,31366968
56,02648227	63,33354687
56,02931818	63,34656377
56,06834953	63,34823633
56,07188809	63,35568347
56,08350425	63,36097762
56,08883568	63,37153383
56,09180778	63,37888053
56,09261855	63,39475537
56,12053724	63,41275231
56,13002712	63,41275231
56,13037807	63,42178589
56,17328601	63,43522302
56,18919536	63,43896391
56,21071168	63,43896391
56,25844706	63,45481136
56,26392653	63,46634806
56,27386366	63,50286811
56,29874358	63,5339687
56,30810467	63,54192136
56,33025515	63,5629438
56,35144073	63,57803866
56,36054486	63,59489827
56,39028802	63,61944275
56,42525657	63,63522642
56,42940143	63,64039148
56,43660811	63,64039148
56,4419292	63,64121148
56,47236351	63,64519971
56,47287181	63,64519971
56,47484903	63,64941129

56,48582996	63,66675281
56,50346555	63,66675281
56,50566005	63,68696803
56,55144866	63,68696803
56,55954874	63,70661982
56,56589129	63,7405296
56,58182649	63,74103237
56,59670768	63,76805169
56,60645138	63,76805169
56,6103964	63,77002706
56,613655	63,78598271
56,61855477	63,79635614
56,63126559	63,79921596
56,65060778	63,79921596
56,687881	63,80568009
56,73094471	63,82791428
56,73519836	63,84881356
56,76158476	63,88317719
56,76546796	63,89634577
56,77352257	63,91340806
56,80729994	63,91340806
56,81331855	63,91768521
56,81516562	63,94183595
56,81585853	63,96312166
56,84871028	63,96598223
56,84933871	63,96873289
56,86510098	63,96873289
56,86684703	63,97290374
56,88411519	63,98246996
56,8922058	63,98361159
56,91266518	63,99293667
56,92772369	64,01494642
56,93150144	64,06508549
56,95477236	64,0659534
56,95802366	64,08386974
57,02191876	64,08981778
57,02735039	64,1034998
57,05146972	64,10544588
57,05764521	64,1105166
57,09842921	64,12950586
57,12097796	64,12950586
57,12824155	64,13797002
57,14838639	64,14859796
57,1554707	64,16355607
57,18703498	64,17598075
57,19366462	64,20412407
57,21258469	64,21432273
57,22682664	64,23020228
57,22891361	64,2366797
57,23953567	64,24416051

57,26459686	64,24416051
57,26798047	64,25259435
57,2694111	64,25259435
57,34491678	64,25587632
57,34558154	64,26615415
57,34914899	64,32308778
57,35747797	64,35041749
57,37504016	64,35041749
57,37576835	64,36466009
57,38800679	64,37593051
57,39756652	64,37593051
57,39865463	64,41536282
57,40262718	64,42244416
57,40761832	64,43125221
57,41921411	64,43393679
57,43827127	64,43393679
57,46186266	64,44438064
57,47780547	64,44438064
57,47828623	64,45389857
57,49340685	64,45649808
57,51757847	64,50489095
57,52331554	64,5114597
57,52626246	64,53204556
57,53353194	64,53454939
57,5402367	64,55457217
57,54767515	64,5554182
57,54790902	64,5630748
57,58358051	64,56743981
57,5957639	64,56743981
57,5964027	64,57971359
57,60080719	64,59956408
57,61811259	64,61819421
57,63904908	64,61819421
57,65492698	64,62991848
57,67354119	64,63480804
57,67681307	64,65795888
57,67773998	64,66077069
57,68114197	64,66077069
57,69677828	64,68756626
57,74221083	64,69680421
57,75953501	64,6970965
57,77696797	64,70075585
57,80730663	64,70075585
57,81515581	64,71196244
57,82090661	64,72325076
57,85808865	64,72689199
57,87792475	64,72689199
57,90851584	64,72913399
57,9146467	64,7442709
57,92388214	64,74937025

57,94321036	64,76091954
57,94676936	64,76230069
57,95003435	64,76260704
57,95466694	64,81242628
57,96426489	64,8341931
57,98268585	64,85090463
57,98940446	64,88463878
58,0091765	64,90046151
58,00944653	64,90046151
58,01312496	64,93404144
58,01487999	64,95400593
58,08467397	64,96363989
58,11507079	64,97449176
58,11559573	65,00009123
58,15078966	65,00009123
58,15524516	65,01510976
58,16921451	65,02190728
58,17081819	65,03026227
58,19124931	65,04150893
58,19482298	65,05664003
58,19865365	65,05900953
58,20816772	65,0854344
58,24062695	65,0854344
58,25372427	65,09325545
58,29200454	65,11713311
58,30261208	65,11713311
58,31026947	65,12694007
58,32429197	65,12694007
58,32964905	65,12936907
58,3681084	65,14020485
58,36839775	65,14321071
58,41607588	65,18021658
58,41663429	65,18129823
58,43383974	65,18149399
58,4663801	65,19464034
58,49766272	65,20054067
58,51624517	65,20337603
58,5693587	65,20337603
58,57955948	65,25096184
58,59658782	65,26596198
58,60320841	65,27079144
58,62001088	65,31192913
58,68410313	65,32153783
58,68518287	65,33682224
58,69280594	65,33749258
58,71720417	65,33749258
58,72789106	65,34490938
58,7318104	65,34657866
58,74210829	65,35914077
58,74390794	65,37711347

58,75085909	65,42507014
58,75243812	65,42955761
58,79033717	65,43128865
58,82663416	65,43128865
58,83116269	65,43704426
58,83513807	65,43704426
58,83821948	65,45413484
58,85053112	65,46957143
58,86156694	65,49942154
58,87147485	65,49942154
58,8725494	65,51054461
58,89595055	65,51950393
58,90568426	65,5280448
58,91073513	65,52926694
58,92481764	65,52926694
58,93889766	65,53178864
58,9477466	65,53929568
58,95757666	65,54573052
58,96652415	65,54573052
58,98893048	65,55189305
59,03856415	65,55665458
59,04647313	65,55665458
59,07526829	65,57585484
59,09221952	65,58084079
59,09754673	65,58626288
59,12257865	65,58985219
59,12917276	65,61059195
59,13968005	65,62413248
59,18156342	65,62413248
59,218527	65,62860992
59,23676524	65,64466006
59,27224693	65,65055646
59,27847315	65,66112575
59,28318074	65,66112575
59,30492828	65,66204299
59,34575719	65,68604405
59,3634632	65,68604405
59,38879199	65,72065951
59,39955386	65,7260076
59,41239439	65,72707588
59,4295127	65,72707588
59,43678615	65,74012556
59,4374871	65,75737148
59,4546245	65,75737148
59,45588216	65,76248454
59,46798956	65,7745589
59,48346086	65,8033445
59,51136668	65,83079668
59,51169561	65,84237822
59,53044651	65,87579589

59,53088156	65,87579589
59,5524471	65,87682306
59,59241204	65,87682306
59,61051087	65,88062284
59,62833564	65,89101858
59,63355402	65,90439155
59,63726334	65,90612579
59,64631912	65,91469978
59,65197645	65,91470907
59,65814934	65,94281012
59,68930048	65,95579968
59,69296614	65,95579968
59,71066867	65,96074203
59,71482838	65,96810117
59,72023606	65,98423077
59,76961761	65,99442363
59,78441381	65,99654422
59,7873505	65,99813468
59,80076806	66,00931593
59,81433011	66,01731339
59,82463763	66,02109521
59,85818561	66,04290615
59,89310539	66,04439539
59,89941718	66,05212439
59,91178692	66,07510712
59,94837884	66,10655192
59,96435288	66,11620221
59,98860071	66,12396936
59,99386845	66,13223532
59,99453161	66,15469224
60,01206357	66,19394508
60,02195052	66,19394508
60,05206196	66,20453104
60,05817757	66,20453104
60,08068495	66,22101598
60,08119042	66,2242501
60,08518972	66,24157785
60,08936107	66,24244938
60,10073948	66,24244938
60,10550442	66,2578539
60,1724886	66,26632756
60,17306032	66,30600963
60,18914162	66,30780191
60,22068535	66,31388578
60,23324291	66,34631034
60,23338186	66,37074153
60,24041931	66,3989485
60,24205468	66,41399118
60,25411368	66,41808037
60,27384994	66,41808037

60,27493864	66,46445641
60,30966817	66,49451668
60,31111438	66,49511117
60,31150252	66,51893148
60,33457761	66,51946359
60,34178826	66,52722066
60,35311819	66,54842409
60,37214247	66,54998588
60,45346412	66,55072193
60,45363491	66,57373515
60,49034432	66,59096387
60,49973672	66,6090097
60,51250264	66,62688289
60,52551727	66,63222744
60,52794445	66,64019872
60,57058935	66,64371181
60,59888557	66,64371181
60,64999893	66,68005941
60,66308074	66,71949474
60,70596159	66,72783523
60,70928288	66,73371257
60,71326187	66,73371257
60,71837338	66,73438335
60,73106618	66,73500978
60,73449873	66,75364675
60,75513028	66,7719464
60,77197251	66,7719464
60,78088659	66,77905995
60,78639938	66,78561248
60,83277088	66,789405
60,85658569	66,79976964
60,89351321	66,79976964
60,8981893	66,81031253
60,9328259	66,82118482
60,94657178	66,82671828
60,96791551	66,84389096
61,00617924	66,85474675
61,01774755	66,86552564
61,01806284	66,86560654
61,03701763	66,94403697
61,03824731	66,94403697
61,07088039	66,94789188
61,07383721	66,95953038
61,10110309	66,95953038
61,10660735	67,00690987
61,12383216	67,01011815
61,15071142	67,02268272
61,1550292	67,02412401
61,16564786	67,03183367
61,21281296	67,03768887

61,22355923	67,04086591
61,23993158	67,04978984
61,24430108	67,06961584
61,25625926	67,08012923
61,25833595	67,08461903
61,26192473	67,08569442
61,29486359	67,08654605
61,33890475	67,08768337
61,35218516	67,08768337
61,36780088	67,12414484
61,41979711	67,13277259
61,44016537	67,13277259
61,4620431	67,14218547
61,47108783	67,14454785
61,47114272	67,15591232
61,48326629	67,1665495
61,49467208	67,17861481
61,50153588	67,19399449
61,51443222	67,21647194
61,52156756	67,21803543
61,53650973	67,22185746
61,56862073	67,22794401
61,56894089	67,2398829
61,57645655	67,2398829
61,59142446	67,26767926
61,61218396	67,27210012
61,62117092	67,27791457
61,65092029	67,29072763
61,68087099	67,30219078
61,69212634	67,30489789
61,69864118	67,30489789
61,7051226	67,31655459
61,72289048	67,33548033
61,73168447	67,344476
61,74393809	67,344476
61,74994962	67,35793734
61,75432856	67,37284116
61,76288289	67,38000304
61,76922339	67,3908931
61,77324211	67,3908931
61,77521044	67,40095581
61,78403559	67,40239225
61,79555281	67,41196857
61,83789055	67,42804998
61,83859002	67,43338236
61,86829376	67,44974332
61,87012495	67,45663295
61,91783364	67,47417657
61,92216513	67,49568649
61,96110971	67,50395861

61,96112992	67,51761092
61,97465419	67,52357548
61,98652596	67,5268637
62,0048938	67,53187364
62,01168323	67,53855436
62,03401573	67,53855436
62,03410117	67,54237501
62,03981278	67,54446654
62,04267831	67,54499788
62,04758575	67,55412547
62,05714839	67,56669211
62,06529862	67,56912995
62,09121804	67,56912995
62,09287619	67,57444762
62,10276888	67,57970156
62,17101744	67,58082942
62,17371988	67,59217474
62,175521	67,59381461
62,17632383	67,60771128
62,19922914	67,6143238
62,20101332	67,61718951
62,21668385	67,62967116
62,21717789	67,62967116
62,21872795	67,63249117
62,22388541	67,63310833
62,23129896	67,63349192
62,25553196	67,63358732
62,27106918	67,63723291
62,33587989	67,64842154
62,41083418	67,65262613
62,41089383	67,66699947
62,41712667	67,6746787
62,44347165	67,6875305
62,44917481	67,69220583
62,45126474	67,70135607
62,45643113	67,7083769
62,46066905	67,72020652
62,46323807	67,72392155
62,46928862	67,72392155
62,4783897	67,73897879
62,47955294	67,75505537
62,48066152	67,76554746
62,48959853	67,76554746
62,51931286	67,80408698
62,52442936	67,80783812
62,52506767	67,81070595
62,52918673	67,81070595
62,56150236	67,81678873
62,57349214	67,8444797
62,57546721	67,8444797

62,57697307	67,84564862
62,60555466	67,85260595
62,60899994	67,86349986
62,63376911	67,86356673
62,63939402	67,87235224
62,64393731	67,87235224
62,65048789	67,89168474
62,65570689	67,90382421
62,65650165	67,91619777
62,66682563	67,91619777
62,67021778	67,91853243
62,67549187	67,92409997
62,70851194	67,92409997
62,71021485	67,94151666
62,71689981	67,94342193
62,72109676	67,94978927
62,73117839	67,97065513
62,7379061	67,97904304
62,75202213	67,98047425
62,77812252	68,00457933
62,80497377	68,00705735
62,80596299	68,00977513
62,81832981	68,01262071
62,82046266	68,01546395
62,82154941	68,01736541
62,82259014	68,02083605
62,82370593	68,04170907
62,83295963	68,0761617
62,84999646	68,0761617
62,87668579	68,07879032
62,89917073	68,08990672
62,91740273	68,09011077
62,92509043	68,1010743
62,93617554	68,10230823
62,94150629	68,12865467
62,95190564	68,13467989
62,95529438	68,13467989
63,0075678	68,1351945
63,00851832	68,14001098
63,00866158	68,15720948
63,01517371	68,16056371
63,04089235	68,16141149
63,05196351	68,16768383
63,09895312	68,17572105
63,11344852	68,17925552
63,11912623	68,1970266
63,13903198	68,24354256
63,17186795	68,24578604
63,17517042	68,26329724
63,18106541	68,26972908

63,18461706	68,27002927
63,18513457	68,29316267
63,20098195	68,29687961
63,2054129	68,31157393
63,22299593	68,31521876
63,24473948	68,31521876
63,24858185	68,34594795
63,25003265	68,35292798
63,2571631	68,37462941
63,26449955	68,37462941
63,26821398	68,38462961
63,27191041	68,40227001
63,30695896	68,40469198
63,31462258	68,40469198
63,33659645	68,40555903
63,33907821	68,42767576
63,35932833	68,42767576
63,36117042	68,45293287
63,39389863	68,46250055
63,40825602	68,47617527
63,42219034	68,47617527
63,43415319	68,47994629
63,4378494	68,50503561
63,46282839	68,51034148
63,49820427	68,51034148
63,50214802	68,53214518
63,52898075	68,53262901
63,53798016	68,54316715
63,5475051	68,58610046
63,54866553	68,59564101
63,58854617	68,597489
63,58941299	68,62198209
63,62641468	68,6232041
63,62848548	68,62798559
63,64550334	68,62859769
63,65620475	68,63055587
63,67871046	68,65736339
63,68779268	68,65736339
63,69699412	68,66258287
63,70490727	68,66258287
63,71262863	68,67499803
63,7135481	68,68602119
63,73483587	68,69015948
63,73602394	68,69664109
63,79275685	68,69671547
63,79903463	68,70306984
63,8232074	68,70431561
63,83291294	68,71558819
63,8829029	68,73760247
63,90023251	68,74488978

63,90579568	68,74696344
63,90738048	68,76209142
63,9178876	68,77458617
63,92240507	68,77554933
63,92634397	68,77566552
63,9356641	68,78438297
63,96383489	68,78438297
64,00278595	68,79143574
64,03388357	68,79143574
64,03404462	68,8009889
64,03612178	68,8022524
64,03905776	68,81464237
64,04112452	68,85030041
64,04435246	68,8504607
64,05271105	68,86064985
64,05545747	68,86195768
64,06199345	68,86501565
64,06408929	68,87501864
64,06978873	68,87501864
64,07768923	68,87785924
64,07943668	68,88701009
64,08148013	68,90634813
64,08645089	68,91101394
64,08897203	68,91120277
64,09752679	68,94656207
64,10408864	68,94752834
64,12642918	68,95111677
64,12687438	68,95111677
64,13427159	68,95258975
64,1504374	68,97566351
64,15146995	69,00166055
64,15320176	69,00209559
64,17505026	69,0154606
64,17815049	69,04186145
64,18767634	69,04402987
64,2090461	69,04506482
64,21689419	69,04590009
64,25039611	69,05547404
64,26229197	69,06027106
64,26276116	69,06170091
64,27154885	69,06873035
64,27211373	69,08653363
64,27457648	69,09371931
64,29966577	69,09371931
64,30808544	69,10678614
64,33574461	69,11467663
64,33588731	69,13103908
64,35734137	69,1330716
64,36104484	69,15409881
64,362066	69,15864245

64,42972851	69,15864245
64,43632233	69,16598205
64,45428029	69,17185166
64,48046455	69,17452033
64,5162359	69,20794915
64,52912714	69,21154003
64,53643556	69,22111123
64,55229975	69,22652862
64,55473453	69,22741822
64,59053365	69,23102872
64,60209392	69,23982604
64,61028938	69,24970024
64,62853809	69,24970024
64,63294267	69,25507482
64,63371422	69,25638184
64,64178297	69,26324164
64,64853296	69,26324164
64,66151593	69,27980059
64,66934136	69,28314447
64,67296702	69,28314447
64,68728239	69,29253285
64,70241817	69,29253285
64,76938443	69,30161265
64,7730535	69,30161265
64,78215305	69,32778401
64,7919406	69,32943888
64,82143432	69,33233858
64,83960626	69,34179524
64,84162066	69,34707788
64,8442241	69,35632549
64,84975565	69,35707566
64,89499256	69,36059556
64,90684763	69,39566639
64,91642079	69,39783052
64,9205267	69,40326899
64,93634269	69,40500187
64,96441575	69,42356754
64,97854669	69,45752261
64,97906479	69,46837961
64,9974	69,47056922
65,01373794	69,47517551
65,01395032	69,48890948
65,02412188	69,49063318
65,03794757	69,49063318
65,04063325	69,5046762
65,04835327	69,52350613
65,08754099	69,52350613
65,10338041	69,52519772
65,11157956	69,52519772
65,12279139	69,52914822

65,15001006	69,54422961
65,20202114	69,54422961
65,20776892	69,55329299
65,22485214	69,55525307
65,24015886	69,56953722
65,25603967	69,57138591
65,26790488	69,57566869
65,2814721	69,58046471
65,29091116	69,59909157
65,30249153	69,61635051
65,30997769	69,61635051
65,31614315	69,64119793
65,31654665	69,64119793
65,33896963	69,64440887
65,34232176	69,66231187
65,34513504	69,68424971
65,34877115	69,6887776
65,35287202	69,69516413
65,36041541	69,71090899
65,36627315	69,72341559
65,38824433	69,72341559
65,38963092	69,77372825
65,39202742	69,77795148
65,41152727	69,77795148
65,42966059	69,78246043
65,43310434	69,79732912
65,49109629	69,80035116
65,49773133	69,80311008
65,50183158	69,8063452
65,50847827	69,80853165
65,55665278	69,81406631
65,56566814	69,82511363
65,56915919	69,82566621
65,59608858	69,82566621
65,62596426	69,83559074
65,65354302	69,83733918
65,66904938	69,839933
65,6842163	69,839933
65,69853079	69,84065907
65,7053223	69,84995274
65,7102686	69,85686682
65,71702381	69,85895
65,74186767	69,86653072
65,74453481	69,86687447
65,74539692	69,87296143
65,7455221	69,87311358
65,75667838	69,88559544
65,76046131	69,90017502
65,76101588	69,90962271
65,77153915	69,90962271

65,77299719	69,91049987
65,78372778	69,91056563
65,82412892	69,9227348
65,84300105	69,92863055
65,89039691	69,93968621
65,90597242	69,94022593
65,95440579	69,94022593
65,95793883	69,96434079
65,95921341	69,97185019
65,98362067	69,97817337
65,99631353	69,99193099
66,00545397	69,99955829
66,01995483	70,00288629
66,0330296	70,00530928
66,03866363	70,00692345
66,0420257	70,01995502
66,08216824	70,03613069
66,08282576	70,03613069
66,08892118	70,06135953
66,09354326	70,06135953
66,09863727	70,06542258
66,09946377	70,06659571
66,12304952	70,07454387
66,1539603	70,07454387
66,16009401	70,08259249
66,16426285	70,09049278
66,19154935	70,10086366
66,198766	70,111547
66,20256221	70,13696509
66,20496924	70,1501358
66,23396177	70,15672031
66,23707788	70,16270831
66,24648269	70,16311641
66,25084662	70,17875592
66,25314267	70,20176572
66,25362742	70,20176572
66,26438614	70,20388282
66,28710512	70,20589465
66,29164878	70,20667253
66,29861187	70,2096916
66,30136645	70,21479343
66,3153118	70,21639713
66,31591553	70,23260365
66,33496854	70,23260365
66,37212188	70,2354435
66,37215861	70,23819081
66,40050594	70,23819081
66,41738688	70,24839858
66,41976273	70,26553184
66,44798906	70,27068125

66,4552428	70,27401836
66,48517284	70,27549333
66,489791	70,28823744
66,5089251	70,28823744
66,52836512	70,29362907
66,53608589	70,29362907
66,55385455	70,29748107
66,56474158	70,31685238
66,5679625	70,32454111
66,61066829	70,32602896
66,67363761	70,34080508
66,69005647	70,34166296
66,69329835	70,35089948
66,71311271	70,35910449
66,71718684	70,36292023
66,73302388	70,36292023
66,73586342	70,37130207
66,73625937	70,38572203
66,7701741	70,38893346
66,77494926	70,39799518
66,8381349	70,39812332
66,85264512	70,41759592
66,8792109	70,42170229
66,88583483	70,42392388
66,88973774	70,43261882
66,90846025	70,43711049
66,91366372	70,43805241
66,93734836	70,44341636
66,94159192	70,44341636
66,95278719	70,44822788
66,97736213	70,45627831
66,98523033	70,46539274
67,01134207	70,46931062
67,02998803	70,49501854
67,03084045	70,49890819
67,03424462	70,56304091
67,06521421	70,56356514
67,07169301	70,56544827
67,08576108	70,56544827
67,08896677	70,58088613
67,09088944	70,58283717
67,12966982	70,61012491
67,15364827	70,61012491
67,18018343	70,6101272
67,19125578	70,62795194
67,20291533	70,63050244
67,23385587	70,63189059
67,25244419	70,6360773
67,25382594	70,6360773
67,25677551	70,66565718

67,26557468	70,6728223
67,27300469	70,67654022
67,28379278	70,68674868
67,28826809	70,7029727
67,29042379	70,70307312
67,29462684	70,71913513
67,29651691	70,72527025
67,30561926	70,72868975
67,32220897	70,7293613
67,33073499	70,73538516
67,33445222	70,76408801
67,38830359	70,76652072
67,38879775	70,76666017
67,41024211	70,7708129
67,41649947	70,77417015
67,41667808	70,77729935
67,48311533	70,78403253
67,48374868	70,78403253
67,53537926	70,78750819
67,5759491	70,79008111
67,5773615	70,79526128
67,61254628	70,79526128
67,6137317	70,79987362
67,62128417	70,81132653
67,62379579	70,81208059
67,62647948	70,83733687
67,65676767	70,88654922
67,69392931	70,89739156
67,69526841	70,90584278
67,69565111	70,91448853
67,70130379	70,91550512
67,71904295	70,91838907
67,72417897	70,92523586
67,74954998	70,93341357
67,76092895	70,93750815
67,76455464	70,93890853
67,77046493	70,94307789
67,78587214	70,94752237
67,80578409	70,95893542
67,81331371	70,97424782
67,83760675	70,97816168
67,83799499	70,99576209
67,84011155	71,01024513
67,84316933	71,02444961
67,86780158	71,02482039
67,87030699	71,02482039
67,8935069	71,02484019
67,89524661	71,04404026
67,90419238	71,04797197
67,91155379	71,04797197

67,91802851	71,09375343
67,9204211	71,09544365
67,92096717	71,14270078
67,94491715	71,14797465
67,94518518	71,15513259
67,94731719	71,15513259
67,95704555	71,15788331
67,96494978	71,15788331
67,99077024	71,16145982
67,99447029	71,16628438
68,04269043	71,16633444
68,04660572	71,17894253
68,04685023	71,19213649
68,04915302	71,20748687
68,06519702	71,22269242
68,08873692	71,23435269
68,11111554	71,24797259
68,11640467	71,2692976
68,13178117	71,27237088
68,14243439	71,28287982
68,15362048	71,28743929
68,1552476	71,28743929
68,15695391	71,29334501
68,15885798	71,30065766
68,16697443	71,30975643
68,1902194	71,30975643
68,20557773	71,31092196
68,22249203	71,31391797
68,23859212	71,31402179
68,24401506	71,31732919
68,25109861	71,31987702
68,27321046	71,3263375
68,27483342	71,32753112
68,27608729	71,33390074
68,28330826	71,34689809
68,29268605	71,36144533
68,2978046	71,36144533
68,32548907	71,37580238
68,32557751	71,38358558
68,33081401	71,40884366
68,33133702	71,40961505
68,33157188	71,40961505
68,33980718	71,41087068
68,33987964	71,43656801
68,34111889	71,43656801
68,3637626	71,44917128
68,36696744	71,45976832
68,37858526	71,46841496
68,38594224	71,47072735
68,41377719	71,47365052

68,41892897	71,48132193
68,43197523	71,51348332
68,46490792	71,51459642
68,48557618	71,51501621
68,49251991	71,51609655
68,50361312	71,51650507
68,5112742	71,52846257
68,51671007	71,52973527
68,51875775	71,53076624
68,52178715	71,54139532
68,55031233	71,55946078
68,55895218	71,57224224
68,56519915	71,5777923
68,58191994	71,57830465
68,60105344	71,58350969
68,60947761	71,58882026
68,62283256	71,59326541
68,62932541	71,6071828
68,6313534	71,60799111
68,6627931	71,61531864
68,66910137	71,64968919
68,68928661	71,66560492
68,68972413	71,67658467
68,70108132	71,67658467
68,73420536	71,68431537
68,75290766	71,69430253
68,75972512	71,69444896
68,76196834	71,70052966
68,76833474	71,70686794
68,76936775	71,70791381
68,77017978	71,71033636
68,78013339	71,71141464
68,82044605	71,71182973
68,82083445	71,71182973
68,82665137	71,71573534
68,82798613	71,7280983
68,84655186	71,7280983
68,84770918	71,73509934
68,85175836	71,74473058
68,92965969	71,74473058
68,94785505	71,77573736
68,96432423	71,77778255
68,9644236	71,78601412
68,97749094	71,78754649
68,99766812	71,79532969
69,00475625	71,80797007
69,01536673	71,81050794
69,03276341	71,81050794
69,0441633	71,83062527
69,04629032	71,83062527

69,05551217	71,84158074
69,08406454	71,85702288
69,09674182	71,8636146
69,12837582	71,86489785
69,14585607	71,8734836
69,14855161	71,88684225
69,16432159	71,88684225
69,20099258	71,88848011
69,21118323	71,88848011
69,21840812	71,89912155
69,25203672	71,90032308
69,2692623	71,90557959
69,26931749	71,92421541
69,29443393	71,93568865
69,33833535	71,93929351
69,35386777	71,94698021
69,35760371	71,95136916
69,36117806	71,9594361
69,3695355	71,95990527
69,37111117	71,96287474
69,37598562	71,97504492
69,37879823	71,99004654
69,391937	71,99400005
69,39244537	71,99792118
69,39357762	71,99792118
69,40812347	72,00031675
69,42969109	72,01352496
69,43021092	72,01352496
69,43088512	72,02154815
69,49416913	72,02444228
69,50165109	72,03467363
69,5065911	72,0440696
69,51595871	72,06663549
69,54288556	72,06663549
69,54630563	72,06955761
69,56912658	72,06955761
69,59575053	72,08705411
69,59869557	72,13465146
69,60711751	72,14505404
69,61517984	72,14505404
69,62462933	72,15193996
69,62532198	72,16363322
69,6475953	72,16478974
69,65665121	72,16478974
69,68040111	72,18711077
69,68159712	72,1913091
69,69367659	72,21815893
69,74629476	72,23082968
69,75752629	72,23082968
69,80682378	72,24464213

69,81664093	72,25132071
69,81942824	72,251683
69,82376181	72,27576817
69,82422185	72,29359516
69,8354706	72,29520289
69,84062049	72,29758521
69,86231452	72,32023492
69,87630916	72,32023492
69,87734285	72,32278864
69,89693877	72,32278864
69,91781059	72,32337452
69,96778192	72,33728372
69,98694616	72,34575237
70,00039615	72,34575237
70,01900409	72,35003685
70,05910109	72,35118363
70,06043964	72,35118363
70,06245148	72,35431269
70,06701606	72,35442346
70,09511872	72,35541051
70,09927437	72,35541051
70,11054243	72,35667976
70,11368605	72,36075132
70,13479143	72,36295675
70,14728261	72,36456754
70,1637731	72,37744752
70,17523357	72,41077862
70,20720975	72,41512584
70,25456088	72,43428831
70,26159178	72,43428831
70,26708461	72,43589607
70,28161695	72,43589607
70,28817409	72,43972385
70,29586567	72,44028948
70,29710349	72,47409067
70,30645137	72,47567036
70,34977192	72,47777594
70,35286884	72,47810074
70,35745538	72,47881046
70,36929931	72,49710061
70,37836886	72,49710061
70,38607395	72,49762451
70,49396894	72,49762451
70,50726517	72,52971702
70,51111343	72,54435496
70,52004563	72,55875448
70,53230817	72,56441959
70,57137636	72,56441959
70,59370819	72,56661108
70,59749746	72,56794608

70,6069998	72,5764251
70,63316194	72,58766549
70,6544038	72,59043031
70,66275493	72,59752867
70,67342673	72,59752867
70,68140395	72,59898044
70,68209632	72,59898044
70,68485266	72,60538741
70,7112973	72,6067908
70,7164569	72,61268131
70,73687756	72,61568303
70,74033225	72,63418542
70,74100303	72,63798628
70,78760623	72,63827514
70,79198031	72,63827514
70,79433506	72,63942918
70,79840189	72,6745992
70,80685536	72,67586163
70,82665578	72,67811677
70,83388869	72,67811677
70,83523222	72,68995511
70,87740755	72,69719553
70,88246714	72,70021636
70,88749355	72,71853334
70,89996897	72,72746044
70,9015626	72,72939721
70,9271293	72,73270931
70,93347335	72,73298418
70,93610252	72,73816332
70,93761713	72,74223062
70,95633416	72,74405716
70,97919402	72,75480106
70,9861832	72,75480106
71,0015977	72,76264105
71,00426979	72,76536902
71,0441916	72,76749699
71,04596608	72,7683852
71,0903728	72,77961137
71,09215652	72,79872296
71,10165374	72,79992105
71,11360707	72,81566802
71,11517121	72,81765269
71,14120375	72,81765269
71,15470113	72,83202839
71,20231152	72,83412296
71,22019554	72,84563543
71,22586385	72,8484588
71,24243054	72,85666919
71,26178901	72,85717817
71,26291901	72,85822769

71,27641181	72,86605004
71,32746453	72,86784276
71,3411592	72,87011453
71,40030031	72,87569818
71,40186579	72,89007486
71,40663473	72,91648745
71,41856565	72,92875712
71,42038795	72,93103117
71,4537628	72,94023851
71,5023268	72,94870656
71,50271852	72,94870656
71,50394065	72,9500201
71,50815301	72,95726119
71,54057115	72,95726119
71,5437831	72,95729055
71,55575942	72,9766445
71,56746555	72,98325244
71,60353568	72,98325244
71,60884911	72,9923538
71,61699106	73,01350111
71,62560939	73,01350111
71,63220508	73,03323599
71,63250605	73,03819217
71,63566479	73,03819217
71,63652596	73,03935879
71,63822539	73,04397807
71,649018	73,0513594
71,66471756	73,05748615
71,70628238	73,06332294
71,7301759	73,06990289
71,73440823	73,0748674
71,73923478	73,07786568
71,74448425	73,08299885
71,76055851	73,08307945
71,76674486	73,09431482
71,76906188	73,10087973
71,77386444	73,10793274
71,80210569	73,10793274
71,84104355	73,11523074
71,84982923	73,11523074
71,85028032	73,13102827
71,86900045	73,13347591
71,91984152	73,13561781
71,93294258	73,14357443
71,93585569	73,14357443
71,94022087	73,14763364
71,9455251	73,15472243
71,95877565	73,15489228
71,96126272	73,16447965
71,96220116	73,17820289

71,96634347	73,17820289
71,96972001	73,18809694
71,9737049	73,18876879
71,97515226	73,19198744
72,01650196	73,19729045
72,02301102	73,19810456
72,0487646	73,19810456
72,07360694	73,19827945
72,08128373	73,20206083
72,10013146	73,20568678
72,11505917	73,21422118
72,12904853	73,22323315
72,142434	73,22378183
72,14568306	73,23512101
72,19439383	73,23512101
72,21700288	73,25078944
72,2192566	73,25078944
72,23341048	73,26733565
72,251504	73,26733565
72,25726365	73,27366593
72,27237952	73,27575954
72,29336909	73,28137178
72,30837473	73,29302191
72,32795513	73,29455056
72,33131296	73,29939469
72,33727395	73,30314201
72,36278266	73,30314201
72,37786044	73,30388435
72,39006886	73,30388435
72,4094354	73,30527687
72,41335473	73,31329152
72,43220766	73,31442542
72,48152145	73,31520689
72,48967512	73,31611844
72,4962391	73,31806643
72,52932661	73,32133655
72,53001848	73,34089331
72,56478958	73,35454984
72,57938052	73,35454984
72,58388577	73,36119402
72,59311236	73,37463019
72,60709341	73,37946995
72,61936701	73,38044799
72,65904829	73,38044799
72,69192781	73,38361646
72,69260347	73,39490928
72,70015166	73,39569111
72,71114162	73,40085644
72,7426326	73,40183417
72,75613264	73,40183417

72,76399484	73,40889112
72,76764034	73,41428033
72,78475761	73,42253793
72,79550511	73,42350878
72,81227165	73,42350878
72,81978764	73,43368694
72,82197586	73,43973216
72,82583346	73,4768294
72,83516821	73,49097723
72,83880843	73,49097723
72,87067647	73,507927
72,88080876	73,52830661
72,92436677	73,53489274
73,00463415	73,53489274
73,00702743	73,53944106
73,03444347	73,54538185
73,12355545	73,54672144
73,14724075	73,54932689
73,15427916	73,55287187
73,15588157	73,55287187
73,16119943	73,557318
73,17513071	73,557318
73,17714109	73,56441628
73,23542361	73,56441628
73,27557814	73,56667836
73,29043256	73,57287196
73,29368873	73,57645907
73,31863953	73,58432903
73,33956632	73,58676233
73,34004126	73,58676233
73,35452529	73,59296423
73,36580284	73,60412141
73,39637737	73,60595205
73,40115763	73,60595205
73,40652951	73,60673489
73,46347092	73,60814338
73,5151331	73,60901625
73,52641746	73,61291762
73,55706512	73,61463616
73,56192074	73,61795259
73,57369762	73,62275458
73,59874195	73,62336633
73,65301461	73,62336633
73,65698481	73,62565275
73,6695824	73,64374138
73,67258741	73,65392166
73,67998863	73,65717573
73,69110062	73,67830238
73,69382338	73,68572815
73,69567861	73,69162298

73,69761107	73,71233637
73,70483676	73,71233637
73,70673179	73,71424898
73,70706869	73,71913286
73,73668264	73,7253875
73,75889268	73,7301613
73,76509523	73,73577554
73,7905622	73,73922628
73,79884544	73,74162916
73,79981265	73,74422115
73,83299463	73,74643214
73,8422915	73,74643214
73,87243867	73,75621387
73,87697643	73,7752485
73,90110878	73,78138257
73,9026933	73,78210713
73,90360221	73,78405637
73,90892904	73,7847213
73,91498036	73,81396568
73,91840663	73,82615814
73,92356118	73,83304654
73,9541707	73,86061818
73,96548069	73,86787379
74,02615918	73,86787379
74,04545405	73,86804828
74,07819839	73,88364991
74,09276698	73,89198956
74,0931911	73,92062306
74,09698548	73,93921309
74,11105948	73,9406789
74,11774771	73,9406789
74,17599166	73,94215265
74,18561125	73,95744442
74,18844653	73,96461431
74,1971036	73,96689006
74,20006291	73,96689006
74,20944384	73,97199262
74,22946041	73,97791766
74,23646879	73,97791766
74,26649809	74,00738382
74,2994812	74,02358552
74,30386554	74,02987224
74,30726942	74,03493419
74,32840028	74,03495911
74,33547379	74,0360813
74,36197215	74,0379749
74,36258655	74,03801686
74,37788489	74,03801686
74,39983709	74,05283243
74,41873631	74,05731992

74,43200006	74,05964674
74,45727394	74,07915545
74,46533285	74,08777602
74,47030853	74,09042338
74,4839313	74,09345923
74,4903339	74,10079535
74,4920174	74,10733003
74,49501321	74,10733003
74,49606155	74,1190097
74,5091965	74,13376348
74,51268711	74,13376348
74,60277677	74,13667234
74,64175993	74,14246133
74,66792458	74,14246133
74,67878227	74,16944389
74,68174568	74,16944389
74,69967237	74,18226957
74,71694587	74,18368359
74,72061212	74,18368359
74,72079669	74,20321392
74,74048802	74,20321392
74,74241173	74,20520836
74,78582009	74,21047575
74,78700255	74,21047575
74,79604104	74,21219399
74,79645821	74,21814624
74,79870106	74,22711776
74,80659311	74,22983364
74,80723884	74,23654674
74,8128318	74,23675358
74,82401851	74,24754937
74,86843405	74,24805091
74,92334135	74,25615767
74,92718335	74,26178506
74,92836811	74,2930998
74,93462666	74,310889
74,96372476	74,310889
74,97068968	74,31920856
74,9736687	74,31920856
74,97910661	74,33558562
75,0114535	74,33558562
75,02517455	74,33708443
75,05648478	74,36154475
75,06512251	74,36154475
75,09209551	74,36180354
75,11736303	74,36180354
75,15195758	74,36720677
75,16007047	74,36720677
75,16460175	74,37386258
75,16749941	74,37551094

75,1760056	74,38297587
75,19475619	74,38677379
75,20878589	74,38985508
75,22106109	74,39939331
75,22282711	74,4077509
75,25734172	74,4077509
75,26392207	74,41933259
75,2640967	74,44205298
75,27325633	74,44402318
75,29508654	74,475017
75,30398612	74,4768722
75,30910779	74,49796677
75,31203822	74,49796677
75,3542814	74,50060953
75,3685779	74,50304439
75,37847956	74,50370926
75,39438745	74,50793101
75,41565952	74,51554078
75,42756696	74,52963533
75,43153419	74,5343664
75,44626051	74,53464828
75,46595117	74,54859698
75,46643481	74,55387594
75,50840297	74,56208738
75,51865732	74,58181437
75,52029393	74,58272272
75,54977102	74,5844401
75,54999048	74,59405773
75,5805847	74,61137994
75,58492377	74,63071915
75,62723617	74,64078109
75,62960931	74,64078109
75,64534495	74,64290106
75,65040832	74,65368456
75,66667331	74,66251107
75,73301938	74,66251107
75,73408016	74,66264923
75,73419724	74,66264923
75,73674606	74,66287777
75,78044991	74,66544333
75,81958485	74,67075018
75,82665882	74,67075018
75,8507182	74,68177352
75,85685691	74,68868572
75,86627134	74,69258807
75,8957674	74,69430223
75,89853459	74,6965268
75,91710533	74,69766522
75,91829518	74,70212706
75,91859325	74,7183978

75,92677075	74,71946385
76,00104382	74,73168934
76,00639354	74,74324696
76,01058381	74,74579086
76,02277072	74,75188148
76,02496379	74,75636265
76,07777815	74,75638457
76,08107043	74,75876884
76,08551627	74,75876884
76,09510185	74,77373156
76,14619799	74,78559093
76,15071799	74,79722716
76,15499636	74,81595051
76,16138406	74,8200245
76,1730355	74,82399085
76,18917974	74,82399085
76,21570971	74,82810238
76,22933893	74,83316022
76,24268411	74,84350184
76,25690149	74,85621759
76,29112102	74,85628252
76,29727981	74,87296327
76,33880414	74,87296327
76,3625746	74,87704739
76,38029094	74,87704739
76,38858707	74,88105632
76,39657991	74,88393609
76,39706991	74,88525927
76,41701745	74,90670269
76,44384623	74,9171811
76,46101889	74,91981835
76,52054012	74,91981835
76,53509389	74,93021443
76,53668699	74,93021443
76,54865835	74,9496851
76,56014683	74,9496851
76,56258497	74,95186376
76,56895647	74,96940639
76,57953797	74,97095511
76,58542814	74,97179874
76,59356632	74,97179874
76,59741651	74,9761967
76,59949607	74,98449851
76,60886	74,98631372
76,61720371	75,00240955
76,63235294	75,01396382
76,63489938	75,01396382
76,63815333	75,02874172
76,65885818	75,02874172
76,6676144	75,03453557

76,73659652	75,04821685
76,75416484	75,04821685
76,77760209	75,05255458
76,80120188	75,05949047
76,83683326	75,05949047
76,84131672	75,06636107
76,85031833	75,08185527
76,85641297	75,08784361
76,86213042	75,10451391
76,89468088	75,10451391
76,91520408	75,12346826
76,92253513	75,12617293
76,93405849	75,12936201
76,94112686	75,13147659
76,97435695	75,13677438
76,99991747	75,14280652
77,00247373	75,14869989
77,02706017	75,14869989
77,03686028	75,15249414
77,04760683	75,15844173
77,05202453	75,16592405
77,07855605	75,16747379
77,08323343	75,17877167
77,08901122	75,1972715
77,13628659	75,1972948
77,17551152	75,1972948
77,18276362	75,20961543
77,19289784	75,21491466
77,22876124	75,21491466
77,2514272	75,22909111
77,26924248	75,2329769
77,38186339	75,2364615
77,39100866	75,24759477
77,39257764	75,24759477
77,40066026	75,25141346
77,41288371	75,25141346
77,42335502	75,26955079
77,43537815	75,27965249
77,44595453	75,27965249
77,45396018	75,29688044
77,45520025	75,31324188
77,45676255	75,31324188
77,48914492	75,3233735
77,50148558	75,32713373
77,5320127	75,33428472
77,56367373	75,33428472
77,58216759	75,33471466
77,59367506	75,33471466
77,60724988	75,34730784
77,61615921	75,35950972

77,64182195	75,36013213
77,64703303	75,36471949
77,66858327	75,36471949
77,67187639	75,36483279
77,69819391	75,36497947
77,70526246	75,37624573
77,7115223	75,37681649
77,7378488	75,37800452
77,75208202	75,3794865
77,76457598	75,40735992
77,78721987	75,41172131
77,80477983	75,44052035
77,80673693	75,44930328
77,80891692	75,45951675
77,81518516	75,45951675
77,8394648	75,47255127
77,84649172	75,47255127
77,90400027	75,48051749
77,90959807	75,48553832
77,9293421	75,49068195
77,9310213	75,49069069
77,95659059	75,50034074
77,96403276	75,50751223
77,97177558	75,50751223
77,99797916	75,50785039
78,00208916	75,50785039
78,00262189	75,50864995
78,00736203	75,50864995
78,02363605	75,50965753
78,02968517	75,52889885
78,08475705	75,52889885
78,08684761	75,53397433
78,10899921	75,55794192
78,12553797	75,56558958
78,13287638	75,56901393
78,16177478	75,57456477
78,17408397	75,58731184
78,17999673	75,58731184
78,18151726	75,60067525
78,21787392	75,61407013
78,23310568	75,62646443
78,2563743	75,63681596
78,26005214	75,64552461
78,26643638	75,65242023
78,31379294	75,6586459
78,31433066	75,66354352
78,33515608	75,67148027
78,34466308	75,69409983
78,37005369	75,69494556
78,40060809	75,71648556

78,40132135	75,71901484
78,40479974	75,71910407
78,40959648	75,73583859
78,47818732	75,73583859
78,49146149	75,73679366
78,49554749	75,74232127
78,51503227	75,75263116
78,5174344	75,75480479
78,54058106	75,75660644
78,56078896	75,76217522
78,57423549	75,76217522
78,61057326	75,76286105
78,61842246	75,76615398
78,65367884	75,7781176
78,68907819	75,78928087
78,70410665	75,78962253
78,74998431	75,78962253
78,75171376	75,82420113
78,75334894	75,82810789
78,75623163	75,83189909
78,76266825	75,84165792
78,78327364	75,84892278
78,80338538	75,84892278
78,82024059	75,85198926
78,82223785	75,85676079
78,83396078	75,85887389
78,84204009	75,86007333
78,85282358	75,86007333
78,861878	75,86970404
78,86998015	75,86970404
78,89522102	75,88272636
78,91368376	75,89511483
78,91958822	75,90162394
78,92424401	75,90378042
78,93309579	75,91131535
78,93404555	75,91557386
78,93494202	75,9174106
78,99152136	75,92535224
78,99302461	75,92723623
78,99547702	75,92723623
79,03272768	75,92901008
79,04573223	75,92947936
79,094987	75,93147354
79,09786878	75,93279234
79,10352424	75,93554455
79,10649087	75,94235661
79,10669929	75,94235661
79,1318258	75,95954531
79,13610226	75,96119284
79,14028045	75,96119284

79,15960064	75,9842856
79,16358822	75,9842856
79,18201351	76,00668335
79,20504281	76,01418019
79,24692224	76,02848281
79,25079463	76,04558977
79,26670654	76,067512
79,26782077	76,07329208
79,30485506	76,08332765
79,31574932	76,09545022
79,32447165	76,11160445
79,33181712	76,13316517
79,34227663	76,13341227
79,36291375	76,13871632
79,38692152	76,13871632
79,424872	76,14461031
79,44050994	76,14525899
79,44446643	76,1461953
79,52056719	76,15056163
79,52716861	76,15056163
79,54460583	76,16292892
79,58221625	76,16657609
79,64057461	76,1744127
79,65250455	76,18219232
79,66521279	76,21269271
79,67106793	76,2199892
79,67288375	76,22256101
79,67560367	76,22256101
79,6888003	76,22479011
79,73939402	76,22479011
79,75293693	76,22873445
79,76540044	76,24116985
79,76770366	76,25380745
79,77927803	76,26183098
79,78298717	76,26599817
79,79421716	76,27172304
79,79430724	76,27437166
79,80577433	76,27437166
79,81078146	76,30107062
79,83257018	76,31110745
79,87559028	76,31284565
79,89315456	76,31284565
79,90415673	76,32807198
79,9631896	76,33107933
79,96736922	76,34983964
79,97762593	76,35399278
79,98704123	76,37446122
80,00111057	76,38782059
80,02373344	76,38847784
80,03117006	76,39200483

80,03338763	76,39281921
80,05440738	76,39965284
80,08017555	76,40991022
80,08215989	76,41238867
80,09103574	76,41238867
80,09225333	76,42282222
80,14803006	76,43218201
80,24119432	76,43898299
80,24835838	76,46318515
80,26277364	76,48442724
80,27399827	76,48973429
80,29775343	76,49898358
80,29976955	76,49898733
80,32196301	76,49898733
80,42901156	76,50715812
80,43320548	76,51408112
80,43907819	76,52153584
80,4459133	76,52445807
80,46587514	76,52445807
80,49276362	76,53207391
80,51135255	76,53207391
80,51424692	76,53562802
80,51784783	76,53766809
80,57454713	76,54168935
80,58796708	76,54168935
80,59462537	76,54533333
80,63456645	76,54533333
80,63763517	76,54748816
80,6680809	76,5696357
80,67467607	76,57389744
80,67885259	76,60045305
80,68081141	76,60655349
80,68109801	76,60724886
80,69776151	76,62970215
80,70575389	76,63909311
80,75026762	76,63996462
80,75587883	76,64077657
80,78514397	76,6422473
80,79165368	76,64864573
80,79241831	76,64864573
80,79453775	76,65034338
80,81221074	76,6628707
80,82832462	76,66457104
80,83950438	76,68059254
80,85177363	76,70371076
80,85420883	76,71788068
80,9039524	76,72207306
80,93206369	76,72207306
80,94866951	76,73299616
80,95721964	76,75405645

80,970433	76,75405645
80,9784416	76,7630958
81,00186548	76,76793325
81,01278413	76,76793325
81,02678105	76,77325444
81,0342355	76,79129098
81,05497975	76,79981225
81,06420777	76,80312345
81,07794928	76,81234989
81,07924307	76,81626929
81,08688512	76,81768307
81,11188079	76,81768307
81,11748173	76,81850672
81,14741618	76,83802101
81,15964521	76,84796435
81,17041587	76,84796435
81,21095995	76,84935437
81,23020655	76,84935437
81,28904788	76,85626561
81,30314819	76,85626561
81,31084097	76,8624321
81,32176704	76,86506877
81,3571996	76,87244824
81,35932932	76,87347874
81,3631981	76,87432205
81,36388555	76,88035525
81,40148709	76,88040604
81,40218504	76,88866195
81,41369415	76,88866195
81,48997217	76,88970828
81,53071452	76,88970828
81,54054166	76,89086607
81,54433661	76,89202032
81,5523155	76,89527444
81,61873198	76,90583716
81,62351876	76,90749284
81,63908602	76,90835397
81,65675089	76,91071331
81,66014351	76,91366538
81,70064226	76,92215775
81,7150001	76,92603369
81,7911817	76,94453238
81,80264803	76,95393754
81,82804678	76,95541312
81,85778566	76,96297701
81,87385982	76,96794586
81,88815601	76,97796096
81,90623123	76,99436591
81,91669257	76,99584425
81,95686466	77,00505628

81,99283015	77,00602822
82,0181689	77,00774863
82,03088897	77,0081126
82,03092282	77,00849951
82,0332429	77,00849951
82,04175653	77,02527169
82,04883816	77,04528864
82,05933914	77,05640048
82,09529177	77,05811293
82,09938143	77,06772432
82,10548405	77,06828198
82,11613089	77,07173851
82,13999014	77,074597
82,14418662	77,0748711
82,15001261	77,0748711
82,15024819	77,09519253
82,15557586	77,09968288
82,16547322	77,10748107
82,17428572	77,10748107
82,19015084	77,10766118
82,22299589	77,11070419
82,22559373	77,11146124
82,23856268	77,11526485
82,24365791	77,11951676
82,25643461	77,12190094
82,27639475	77,15039027
82,27969743	77,15172752
82,28040887	77,15463561
82,33456424	77,15483542
82,34771155	77,15486976
82,3498157	77,15865519
82,35799534	77,15963182
82,36447161	77,16577704
82,36723394	77,17759152
82,37578996	77,21292447
82,40536429	77,22216451
82,40953181	77,25609169
82,41391212	77,2709324
82,42213623	77,27985852
82,43025047	77,28345771
82,44427425	77,28345771
82,47742675	77,28492395
82,52209727	77,29308352
82,53491595	77,29557822
82,56116482	77,29605948
82,57402871	77,29825734
82,57713518	77,30009255
82,57844764	77,30341837
82,60244668	77,30954563
82,61552264	77,3200222

82,62871051	77,32252861
82,66613153	77,32654899
82,67877481	77,32654899
82,68332652	77,34149347
82,68888347	77,34159224
82,70077496	77,35441861
82,72095847	77,35547814
82,7259077	77,37460191
82,74870615	77,37806263
82,75557287	77,3875765
82,76622315	77,40109738
82,8226594	77,41284354
82,85134271	77,4236815
82,86464037	77,43042961
82,87064048	77,43305464
82,88464231	77,45355642
82,88618508	77,45355642
82,89118996	77,45403008
82,89453404	77,47134452
82,90711029	77,48794987
82,9324955	77,48837921
82,93509528	77,4969058
82,94332729	77,51139955
82,99140369	77,52013603
83,00113031	77,52013603
83,0067339	77,52117568
83,01616475	77,53571147
83,02155414	77,54874297
83,02303568	77,54874297
83,04432257	77,55832658
83,04830825	77,55962443
83,05911237	77,57185063
83,06023985	77,57207272
83,07324874	77,57794417
83,0744129	77,59092441
83,08964265	77,59140005
83,09042081	77,59361143
83,09189149	77,59456383
83,09379673	77,60312094
83,09683708	77,60849645
83,09870597	77,62654072
83,14233284	77,63493815
83,16724247	77,64013208
83,18284959	77,64013208
83,18489164	77,64137741
83,19252655	77,64614758
83,23125881	77,64993336
83,24201453	77,65734687
83,25098701	77,66577641
83,27068434	77,66779001

83,31947609	77,68120325
83,3361017	77,6836306
83,35815922	77,68534647
83,39164328	77,69522837
83,39777341	77,71135875
83,41817392	77,71149134
83,42237803	77,7138135
83,42573341	77,72539708
83,4573019	77,74017277
83,47211355	77,75023353
83,47596557	77,75023353
83,47996012	77,75024965
83,49500785	77,75268588
83,4987468	77,77432479
83,53034312	77,79675769
83,53561064	77,79675769
83,56148321	77,79825479
83,56762245	77,79850382
83,58201938	77,79850382
83,58262023	77,82168873
83,60095089	77,84254695
83,63428749	77,84513341
83,6544857	77,85200028
83,66630621	77,8654469
83,67970939	77,87051017
83,700315	77,88004091
83,71783666	77,88004091
83,75257464	77,89338534
83,75743061	77,90644855
83,79548634	77,9100184
83,82590692	77,9340149
83,83128759	77,94055231
83,83550732	77,94379683
83,83614854	77,95145771
83,87984849	77,95145771
83,89222805	77,9697254
83,8926838	77,9697254
83,93271887	77,97138222
83,93567506	77,97138222
83,93914275	77,97269443
83,99397864	77,98085236
84,00558671	77,98147259
84,05460284	77,98147259
84,0824966	77,98339465
84,11017412	77,99885331
84,11986612	78,00578031
84,14228515	78,0088589
84,22724068	78,01429953
84,24801849	78,02612394
84,27667611	78,02852775

84,31652876	78,03153131
84,33336087	78,03153131
84,36394602	78,05396053
84,39074861	78,05396053
84,39812616	78,08248037
84,41776511	78,08248037
84,4422722	78,08249726
84,4431282	78,08783969
84,47053631	78,08868061
84,4716919	78,0935461
84,48498046	78,10834548
84,49356765	78,10834548
84,49899616	78,11156401
84,50328626	78,11444921
84,52280714	78,11671659
84,52282949	78,11786732
84,52563513	78,12973095
84,54677591	78,12973095
84,5482084	78,13062919
84,54925951	78,13680345
84,5732785	78,13680345
84,58314114	78,14643071
84,59315754	78,14643071
84,6301012	78,15950694
84,68943354	78,16921267
84,69477226	78,17263734
84,7124445	78,172841
84,71920465	78,17500641
84,7509586	78,1989032
84,77382952	78,2014432
84,77727743	78,20339992
84,7806229	78,20381553
84,78363804	78,20890382
84,79376273	78,20914633
84,83544134	78,21605719
84,87542886	78,21605719
84,89675376	78,21797711
84,90442986	78,22673004
84,90469935	78,2299626
84,96093794	78,23566688
85,02620419	78,24381365
85,03898055	78,26178196
85,04815414	78,26301383
85,08269396	78,26301383
85,09241721	78,27220937
85,09295347	78,28662095
85,10526006	78,28662095
85,11938177	78,3119803
85,12381838	78,31609428
85,15186331	78,31609428

85,15514382	78,33116682
85,17859184	78,33171721
85,18164277	78,33429433
85,23198118	78,33429433
85,2326485	78,33494952
85,23562241	78,34157911
85,24004338	78,35730449
85,26056961	78,36606885
85,2918812	78,36690294
85,35529273	78,36859072
85,37046229	78,37026361
85,42861984	78,38370762
85,43129553	78,38490812
85,48496218	78,3854681
85,48573294	78,40080442
85,49623109	78,40515488
85,52106281	78,41080477
85,54195671	78,42222814
85,58361608	78,42222814
85,59239565	78,42311577
85,593244	78,42701214
85,6005393	78,43250568
85,60865684	78,43822005
85,61868247	78,44166736
85,65085246	78,44166736
85,6571587	78,44254752
85,66545923	78,4771224
85,66689231	78,47764446
85,73671219	78,4783631
85,75214719	78,4783631
85,76038539	78,49098791
85,76957767	78,49119044
85,78682458	78,50461131
85,80522689	78,5284675
85,81129808	78,54349086
85,83880607	78,54772742
85,84600857	78,55739567
85,87674985	78,55739567
85,92314853	78,56049711
85,99695203	78,56049711
86,01614856	78,56475603
86,05059886	78,56475603
86,05911449	78,57001457
86,06675478	78,57706144
86,08075792	78,59721741
86,12670553	78,59763255
86,15530321	78,61125908
86,17467037	78,6242
86,18977738	78,63207252
86,22210968	78,6535173

86,24358981	78,66170056
86,2685205	78,66806171
86,28096975	78,6954055
86,38210746	78,70024927
86,39187063	78,70024927
86,39810069	78,71344524
86,43296686	78,71344524
86,44049795	78,72011433
86,44648032	78,72746246
86,44935022	78,72879997
86,44971725	78,73302942
86,46344434	78,74856499
86,49972556	78,74856499
86,51015165	78,74866142
86,53218251	78,7537602
86,54539012	78,7670629
86,54929968	78,77753016
86,56644748	78,77933927
86,57503502	78,77973627
86,66835009	78,78064672
86,67988629	78,7981687
86,69731832	78,80092848
86,70109415	78,81338145
86,70508933	78,81338145
86,71540547	78,82127386
86,75950603	78,82127386
86,78479272	78,82230964
86,79779375	78,84260133
86,79800202	78,84460399
86,82567387	78,84816877
86,83803359	78,84854869
86,84505864	78,84854869
86,84800769	78,84957806
86,86181662	78,84957806
86,8640328	78,85244101
86,86925302	78,86416976
86,8738871	78,88179929
86,88525585	78,88612715
86,89059389	78,88612715
86,90952532	78,88753297
86,91761165	78,88753297
86,92790796	78,89851275
86,92813321	78,92626446
86,97756721	78,92626446
86,99013403	78,95155868
86,99578033	78,95445644
87,01398836	78,95445644
87,06053165	78,96912556
87,07030341	78,98259404
87,08582305	78,98895735

87,13172711	78,99298031
87,13601439	79,01468723
87,20777945	79,01674178
87,21034617	79,01874086
87,21630728	79,01874086
87,24575484	79,0231542
87,31283568	79,02850209
87,317242	79,02850209
87,32742712	79,03399115
87,35729435	79,03642864
87,38203143	79,03648985
87,3983427	79,05002434
87,43030253	79,05002434
87,45071055	79,0549683
87,46550845	79,05576929
87,50791837	79,06074429
87,51185964	79,06074429
87,52095955	79,06153519
87,52165306	79,06153519
87,53216237	79,06211378
87,53378139	79,06490672
87,54368368	79,07366498
87,55964364	79,07501602
87,57686304	79,09024836
87,57873086	79,09277861
87,58491302	79,09650884
87,59884732	79,11236266
87,62119612	79,13800794
87,68746972	79,16106918
87,69524649	79,16934242
87,71038228	79,18228329
87,71528798	79,18506223
87,72380345	79,18704833
87,7261313	79,18881095
87,73372598	79,18992924
87,75529943	79,19053737
87,80953342	79,19053737
87,81244376	79,19648641
87,84520011	79,19726728
87,88877286	79,20416591
87,91958976	79,21786262
87,94329132	79,2251775
87,9583805	79,23445216
87,96599962	79,23861738
87,99422338	79,24081249
88,02078796	79,24081249
88,03790607	79,24384695
88,04370591	79,25423741
88,05388554	79,2622094
88,07742856	79,26552561

88,09299681	79,27231875
88,10801253	79,27231875
88,12236822	79,27543595
88,12815798	79,27881808
88,13226219	79,2838188
88,14804104	79,2874898
88,16959172	79,28815748
88,1885603	79,30045242
88,18875543	79,30477173
88,21543569	79,30959226
88,26799987	79,31212561
88,28184432	79,31532194
88,29126192	79,32401105
88,29680978	79,32847658
88,31260468	79,33660971
88,31349019	79,33660971
88,3157436	79,33915531
88,32445458	79,34344369
88,33042732	79,34480382
88,34917126	79,34549927
88,35838487	79,35560864
88,39909066	79,35560864
88,43296716	79,35676825
88,49134026	79,35740474
88,53226034	79,37043674
88,53786924	79,37043674
88,54973705	79,37183092
88,56434324	79,37183092
88,61754532	79,37648636
88,62774204	79,38303661
88,63654621	79,39828906
88,65276341	79,39828906
88,69116877	79,40695814
88,69322713	79,41966083
88,70450374	79,43776381
88,70808271	79,44334344
88,75861266	79,45364029
88,77985484	79,47349624
88,78400268	79,49409348
88,78862454	79,50429031
88,80725456	79,51227994
88,83402909	79,51462756
88,87679542	79,51833957
88,88404562	79,51833957
88,8891424	79,52644616
88,93684851	79,52711881
88,97368745	79,53358058
89,01799036	79,54362908
89,0330006	79,54806056
89,059452	79,55527335

89,06754168	79,55567531
89,09068845	79,55856573
89,11577484	79,56636421
89,18584698	79,57277902
89,22237051	79,58701358
89,24169808	79,60035974
89,24202728	79,60279011
89,25395383	79,61645081
89,25959887	79,62619088
89,2703746	79,6376866
89,27163367	79,65492808
89,27501547	79,66171428
89,29958397	79,66331245
89,31173413	79,66331245
89,31913697	79,66434184
89,32638826	79,68140021
89,33236718	79,68636334
89,34886181	79,69748857
89,37563253	79,70285782
89,37838188	79,71276595
89,40731319	79,73099597
89,42420979	79,73586344
89,46721081	79,73586344
89,47081749	79,73894278
89,49846025	79,75023506
89,49953511	79,75709435
89,5048938	79,77522769
89,52331871	79,77585408
89,53182373	79,78561188
89,53881283	79,78651073
89,57835866	79,7900076
89,60774973	79,7900076
89,61192755	79,80165505
89,64754547	79,8052288
89,67687707	79,82762047
89,67824939	79,84554258
89,69398908	79,85736376
89,70259325	79,85972977
89,70741634	79,8635435
89,72175326	79,86492326
89,74866595	79,86492326
89,76999007	79,87345434
89,82526826	79,87551381
89,83423103	79,87551381
89,84472277	79,88401164
89,84720831	79,89767386
89,86402616	79,89767386
89,86878138	79,9023636
89,87041999	79,9148168
89,88123383	79,92227298

89,89489171	79,94067052
89,8985812	79,95597202
89,9083872	79,970996
89,91900648	79,97540163
89,92104022	79,99570558
89,92137469	79,9966072
89,94499422	80,01960847
89,9479217	80,02055365
89,94793625	80,02180007
89,95705881	80,02619799
89,96045999	80,04076881
89,97610014	80,04272083
89,98089883	80,05590856
89,9954849	80,06371059
	80,0731853
	80,08759762
	80,08759762
	80,08920128
	80,0930577
	80,0930577
	80,11430785
	80,11825954
	80,1191035
	80,12786287
	80,13061409
	80,1341176
	80,1387291
	80,14862335
	80,15345941
	80,16382487
	80,16743877
	80,16960692
	80,19171886
	80,19171886
	80,1965806
	80,20014853
	80,20356833
	80,22776377
	80,24125631
	80,24295118
	80,24374292
	80,24374292
	80,24418839
	80,25006271
	80,25474387
	80,25762529
	80,25769367
	80,26558662
	80,26856041
	80,29030349

80,30251434
80,30281671
80,30907593
80,31756589
80,32094464
80,3240831
80,32615499
80,34105263
80,34502275
80,35203106
80,35203106
80,36963912
80,44336453
80,44754089
80,4505951
80,46340565
80,48063607
80,48147744
80,48147744
80,48281591
80,49607311
80,49607311
80,50102587
80,50524014
80,51094528
80,52254731
80,52653122
80,53002787
80,53330018
80,54966483
80,578413
80,57871587
80,58418791
80,58418791
80,59949779
80,60313246
80,60373487
80,60599953
80,60776768
80,60914465
80,61789576
80,62820526
80,63815711
80,63815711
80,64674006
80,65193476
80,65193476
80,65363023
80,65363023
80,65390486

80,65390486
80,66161865
80,66171524
80,66450691
80,67816525
80,6815546
80,68481309
80,68647055
80,68960739
80,69240339
80,69265571
80,69382371
80,70992849
80,71175356
80,71243579
80,71951175
80,72027403
80,72503522
80,73323303
80,73568752
80,73568752
80,73590055
80,73590055
80,74346029
80,7495879
80,74986104
80,75281649
80,75355199
80,75476601
80,7556578
80,77494507
80,77654162
80,79525268
80,79525268
80,80693259
80,80846759
80,81025615
80,81196071
80,81262552
80,81262552
80,81558937
80,81742781
80,81854041
80,81981456
80,81989802
80,82290196
80,83724141
80,83878947
80,84513703
80,84513703

80,8715932
80,8715932
80,8728716
80,90889817
80,91059839
80,91061208
80,91165776
80,91563971
80,92052596
80,92052596
80,92642854
80,9272993
80,92733945
80,92997784
80,9461072
80,94795578
80,94795578
80,97156161
80,97156161
81,00149434
81,00149434
81,00484855
81,00523734
81,00901417
81,01731054
81,01731054
81,0253236
81,0253236
81,02605802
81,02605802
81,02650423
81,02650423
81,03643841
81,03643841
81,03758023
81,04322779
81,0483763
81,0494849
81,05925122
81,05925122
81,09953111
81,09953111
81,10356668
81,10363086
81,11654349
81,11654349
81,12845275
81,1324329
81,1354808
81,13884013

81,15523744
81,16076599
81,17924702
81,18712058
81,18714169
81,19570314
81,19802033
81,20406308
81,2077049
81,20866972
81,21403952
81,21896276
81,23132379
81,255392
81,26305388
81,2667778
81,2722008
81,27381666
81,31711259
81,31974431
81,3277425
81,34118005
81,34118005
81,35584131
81,37023526
81,37096912
81,37655834
81,37655834
81,39261168
81,40436512
81,40740654
81,41319904
81,42518291
81,43497483
81,43497483
81,43627958
81,43627958
81,4465593
81,45432572
81,45548036
81,45575099
81,4571273
81,4614566
81,46193542
81,46240631
81,46240631
81,47462628
81,51255211
81,51255211
81,51825098

81,52209194
81,53361569
81,53361569
81,53603935
81,54439666
81,54900849
81,54900849
81,55967346
81,5626905
81,57110021
81,57849645
81,58761955
81,59198921
81,60077187
81,61050537
81,61050537
81,61167055
81,61238438
81,6158475
81,62038865
81,62038865
81,63300652
81,63300652
81,63722977
81,6475699
81,65527954
81,65804131
81,65804131
81,66616863
81,69060351
81,69635434
81,70472043
81,70507001
81,72138428
81,72376013
81,72587125
81,72717191
81,72717191
81,7298531
81,75328173
81,75898051
81,76280848
81,7652983
81,7749432
81,7777956
81,78628376
81,79252311
81,80320914
81,80320914
81,80910624

81,80910624
81,81496789
81,82517135
81,84757478
81,85035707
81,85931578
81,86744893
81,89641664
81,90446105
81,9277561
81,93733495
81,94216909
81,94633523
81,94633523
81,9492767
81,9492767
81,9545516
81,96595098
81,96595098
81,97002234
81,97482198
81,98339714
81,98339714
81,98622626
81,99581119
81,99581119
81,99762845
82,00006787
82,00006787
82,00261629
82,00444569
82,01532769
82,01735143
82,02392877
82,02392877
82,03551423
82,03687263
82,03797953
82,05811538
82,05811538
82,06553718
82,06787677
82,09311282
82,09311282
82,10244521
82,11106017
82,12168106
82,1287223
82,13379521
82,13379521

82,1394462
82,1404704
82,14848144
82,15056393
82,15975412
82,1599208
82,1599208
82,16785439
82,20121313
82,20216702
82,22361439
82,22452227
82,23644718
82,24205818
82,24205818
82,24223373
82,2438744
82,24404346
82,24708187
82,2720418
82,2748258
82,27865573
82,27865573
82,27891251
82,27891251
82,27922901
82,28577235
82,29051903
82,29601703
82,3023925
82,3055736
82,3055736
82,3129168
82,31765977
82,3319282
82,34931833
82,35094841
82,35892734
82,37200115
82,37212258
82,37511093
82,39739066
82,398782
82,398782
82,40315263
82,40583267
82,40583267
82,41672446
82,41672446
82,42400553

82,42513693
82,42513693
82,44428521
82,44455944
82,44455944
82,4482727
82,4482727
82,45475981
82,45668876
82,46563671
82,4672343
82,50228265
82,50228265
82,50856464
82,52409977
82,541981
82,54367417
82,54546007
82,5658943
82,5658943
82,5684824
82,57499639
82,57530968
82,59121825
82,59389502
82,60016167
82,60016167
82,60530728
82,60787789
82,60787789
82,61188915
82,61188915
82,61393794
82,61913472
82,62996143
82,63664409
82,63788817
82,64225265
82,64376454
82,64497372
82,6456852
82,64842367
82,64842367
82,67100497
82,67482242
82,67551099
82,6821546
82,71670482
82,71795981
82,71795981

82,72376271
82,72506874
82,73180196
82,75559897
82,7707705
82,77187793
82,77187793
82,77431574
82,77436175
82,77727739
82,77953124
82,78009818
82,78688132
82,81894091
82,81894091
82,82577186
82,84494804
82,85116104
82,85116104
82,85565235
82,86578854
82,87918717
82,88444266
82,90317314
82,90844804
82,90844804
82,91232639
82,91327836
82,92519944
82,93719666
82,96204576
82,96732303
82,96843372
82,97199412
82,97348559
82,97348559
82,97454045
82,9956107
82,9956107
82,99761664
82,99761664
82,99780987
83,00690494
83,00777949
83,0159247
83,01854849
83,02060203
83,03393591
83,05543821
83,06723683

83,07394456
83,08214989
83,10444271
83,11693508
83,11693508
83,12005912
83,12005912
83,1256725
83,12639086
83,12820093
83,13231016
83,13459307
83,13459307
83,15641988
83,16320397
83,17297835
83,17297835
83,17370099
83,18161967
83,21095895
83,21451276
83,23317728
83,23497299
83,24949551
83,24949551
83,25285581
83,25285581
83,2562072
83,2562072
83,27301431
83,27712899
83,28700678
83,28710791
83,2933069
83,2933069
83,31163439
83,31869433
83,318945
83,31973513
83,32539889
83,34563089
83,34600518
83,3515752
83,36085301
83,36281018
83,36281018
83,37855097
83,38702711
83,39832359
83,3987336

83,3987336
83,39951359
83,4287233
83,4320671
83,43358843
83,43552518
83,43947588
83,43966188
83,44753544
83,45034273
83,45034273
83,45606664
83,45606664
83,46617884
83,46858677
83,47160261
83,48357446
83,48963666
83,49941825
83,49951727
83,50877485
83,50939037
83,50939037
83,51595206
83,51967769
83,52161529
83,52161529
83,52371118
83,52371118
83,53120703
83,5402375
83,54110824
83,54267647
83,54448389
83,55650296
83,55650296
83,55791308
83,56361662
83,56361662
83,572617
83,5800173
83,59686488
83,59686488
83,59786657
83,62247792
83,62274568
83,63305725
83,639447
83,64645306
83,65141555

83,65141555
83,65213101
83,65213101
83,65796008
83,66408552
83,6865804
83,68766055
83,68766055
83,69846442
83,70943954
83,70943954
83,71045684
83,71090293
83,71425528
83,71641503
83,72853128
83,72961258
83,73903567
83,7453207
83,7453207
83,74826468
83,74852465
83,74852465
83,77515732
83,77515732
83,781912
83,78557106
83,79444895
83,79444895
83,80257983
83,8072599
83,8072599
83,80969437
83,82696419
83,84812766
83,86180118
83,86180118
83,86537962
83,88525423
83,88814211
83,8912858
83,89416634
83,89485397
83,89877015
83,90813089
83,92348966
83,92540381
83,92716189
83,92892851
83,93305802

83,93653727
83,94138503
83,94175302
83,96274265
83,96384884
83,96384884
83,96565647
83,96681531
83,97399046
83,97399046
83,98160071
84,01020296
84,01020296
84,01127392
84,01127392
84,01263816
84,01313999
84,01353938
84,01923759
84,01948159
84,02272232
84,02274977
84,02768428
84,03493984
84,03493984
84,03775949
84,0481544
84,0481544
84,05571134
84,05966724
84,06231081
84,06231081
84,06357848
84,09530493
84,09530493
84,09856443
84,10319122
84,10600243
84,1079268
84,10977369
84,12557237
84,12557237
84,12851297
84,13066814
84,13607073
84,13607073
84,14186466
84,14268638
84,1578656
84,1578656

84,15998644
84,16212591
84,16311261
84,18064956
84,18064956
84,18374685
84,18765979
84,18835427
84,19347496
84,19347496
84,19527401
84,19642892
84,20722112
84,21280219
84,21349645
84,21452174
84,22183743
84,22183743
84,23360583
84,23360583
84,23912771
84,23961399
84,24132944
84,24132944
84,2454299
84,25829873
84,25829873
84,25863104
84,26173124
84,26473428
84,27515339
84,28716495
84,29607893
84,30611418
84,32830668
84,33788965
84,34930758
84,35495738
84,36394444
84,3822043
84,38293506
84,38293506
84,3897509
84,39186327
84,39248479
84,39461144
84,39641763
84,3974305
84,41715063
84,41715063

84,42144339
84,42144339
84,43528391
84,43885706
84,43885706
84,4494005
84,47531821
84,47816763
84,48275711
84,4983231
84,51299764
84,53379965
84,54697
84,54934057
84,55111939
84,55918592
84,55992845
84,56764984
84,56764984
84,60263194
84,61101454
84,63341854
84,64685529
84,64943694
84,64990409
84,65980238
84,68648085
84,72170419
84,72774118
84,74474004
84,74822516
84,75237197
84,75373003
84,75577681
84,75729413
84,75729413
84,75924174
84,75924174
84,76234372
84,77276124
84,77817023
84,80132579
84,80132579
84,80416454
84,81429711
84,82068401
84,83990752
84,86621539
84,86632796
84,87683106

84,88153994
84,88227758
84,89180451
84,90759725
84,9148935
84,9148935
84,92928443
84,93157481
84,94783725
84,94783725
84,9591731
84,96306165
84,97119965
84,98975695
84,99194845
84,99852256
85,00345215
85,00562938
85,02178194
85,02654667
85,03002935
85,03656141
85,05166034
85,05166034
85,05899007
85,06147392
85,06552315
85,06742969
85,07722688
85,07722688
85,08265193
85,09951821
85,09951821
85,11366256
85,11750663
85,12049625
85,12178423
85,1239827
85,13633892
85,13754571
85,15899558
85,16203077
85,17925725
85,18588623
85,1995182
85,20802376
85,22029554
85,22159612
85,22322212
85,224499

85,2319733
85,23519417
85,23837759
85,23873566
85,24099923
85,24099923
85,25134226
85,25451617
85,25578111
85,2762698
85,2762698
85,27998002
85,28434016
85,28763134
85,29041265
85,31461917
85,32158734
85,32158734
85,33072389
85,35239865
85,35747099
85,35747099
85,36426107
85,36426107
85,37020005
85,37374562
85,37512166
85,37876953
85,38524905
85,39703008
85,40226418
85,42119161
85,4315766
85,45224185
85,45518799
85,45585383
85,46273003
85,48649318
85,48649318
85,49153278
85,50935675
85,51156455
85,51803441
85,52067302
85,52335422
85,52335422
85,53631929
85,5583413
85,55985965
85,57300904

85,57300904
85,59101238
85,59942983
85,59942983
85,60060397
85,60727053
85,60769651
85,60769651
85,61055568
85,6128982
85,6128982
85,61475172
85,61475172
85,61827754
85,62454038
85,62454038
85,62462733
85,62517672
85,62797249
85,64549226
85,65424592
85,65854047
85,66330942
85,66578755
85,66979664
85,67140185
85,67140185
85,67434697
85,67797822
85,67971468
85,68033708
85,68344614
85,71032152
85,71506749
85,73722255
85,73722255
85,73765383
85,73881682
85,74334892
85,74334892
85,7537923
85,7537923
85,76039999
85,76515339
85,77249797
85,77647863
85,77647863
85,77680366
85,7788603
85,79352239

85,79829708
85,79887132
85,82036627
85,82690803
85,82690803
85,82885327
85,83862926
85,8549396
85,86325607
85,86325607
85,87665326
85,8810263
85,88592494
85,88662905
85,88869723
85,89337556
85,90643664
85,9076437
85,91383216
85,91621864
85,91676637
85,91676637
85,92581859
85,93416314
85,95779392
85,96090042
85,96090042
85,96202283
85,965176
86,00405756
86,01584431
86,04601967
86,04781727
86,05774612
86,06223132
86,06287449
86,06287449
86,08052425
86,08774601
86,10447712
86,10447712
86,11187003
86,12132396
86,12319415
86,13571602
86,14022297
86,14261953
86,15647764
86,15647764
86,16610943

86,16666985
86,17202001
86,19089976
86,19089976
86,19452969
86,20983333
86,21788881
86,22209544
86,22821146
86,23650163
86,23650163
86,24899918
86,26678445
86,26989663
86,27145821
86,28182406
86,29420995
86,29810566
86,31199676
86,31199676
86,33099869
86,33099869
86,34361557
86,35692183
86,36021572
86,37359672
86,3781985
86,39084881
86,39103859
86,3941346
86,3941346
86,40307751
86,40375817
86,40375817
86,40412219
86,41323013
86,41425175
86,41425175
86,42997896
86,44064321
86,44346288
86,45049773
86,4539514
86,45729993
86,47511133
86,47511133
86,48496776
86,4946781
86,49957124
86,49957124

86,50568132
86,50568132
86,51031734
86,5227998
86,52451624
86,53593209
86,53593209
86,53868456
86,54207114
86,57289435
86,57860301
86,58238915
86,58682639
86,59094329
86,59984358
86,59984358
86,60249197
86,61327574
86,62046568
86,6228458
86,62689358
86,63529518
86,63541515
86,64994034
86,64994034
86,68786409
86,68786409
86,70358652
86,70358652
86,7130272
86,73629396
86,73629396
86,73674302
86,74017456
86,74093195
86,74169744
86,74169744
86,75312858
86,75787641
86,76094453
86,78041021
86,78247051
86,78315376
86,78576884
86,81575894
86,81575894
86,83552384
86,84240073
86,86811312
86,88131555

86,89128489
86,89128489
86,8917821
86,89966831
86,90597084
86,90902052
86,92433401
86,92433401
86,93530805
86,9434952
86,95538984
86,95538984
86,97113729
86,97344089
86,97507047
86,9870501
86,9870501
87,0000845
87,00769729
87,00968093
87,03544765
87,04783008
87,04783008
87,05604467
87,06008437
87,0634078
87,06487058
87,0707829
87,07184435
87,07207395
87,10562088
87,10562088
87,10684331
87,10684331
87,10833263
87,10833263
87,11720054
87,11736696
87,13323928
87,13575758
87,14612944
87,14830124
87,15639428
87,15840399
87,15840399
87,16589758
87,1732608
87,17874044
87,17975711
87,18330439

87,19316535
87,19612241
87,21437402
87,21437402
87,23320577
87,25021109
87,25116253
87,25130068
87,26000515
87,28675576
87,29124488
87,29585496
87,31634759
87,3219334
87,3219334
87,32493664
87,3332831
87,33597868
87,34871409
87,35595792
87,36104475
87,36262654
87,36429809
87,36498883
87,36933839
87,36933839
87,37357654
87,37357654
87,37581919
87,37581919
87,37938046
87,38559144
87,38918438
87,39006874
87,39506798
87,3954249
87,3954249
87,42392716
87,43657178
87,4422142
87,4422142
87,44987435
87,45936864
87,46166952
87,46490124
87,49279126
87,50465963
87,51644782
87,52066129
87,52066129

87,52746121
87,53473716
87,53474805
87,53559732
87,54551126
87,55095367
87,551892
87,56040906
87,56760275
87,5695359
87,58584921
87,58584921
87,59146309
87,59146309
87,59234448
87,59642461
87,59642461
87,60653338
87,60653338
87,61615509
87,61615509
87,63600227
87,63877241
87,63877241
87,63935729
87,64382403
87,67160423
87,67160423
87,67410238
87,67410238
87,68403917
87,68779977
87,69361665
87,69404001
87,69766825
87,70640758
87,7094116
87,71682185
87,71968066
87,71968066
87,72048715
87,73267866
87,73267866
87,73827433
87,74684811
87,74913632
87,77703727
87,77703727
87,77907312
87,77956341

87,77991109
87,77991109
87,79331353
87,79348151
87,79712537
87,79893999
87,79893999
87,81206623
87,81899152
87,81899152
87,82668225
87,82668225
87,83646698
87,83654509
87,84342035
87,84342035
87,84448857
87,84448857
87,84977952
87,85159802
87,85977024
87,85977024
87,86165615
87,86473318
87,87835477
87,87835477
87,88423307
87,88614856
87,88614856
87,89077106
87,89948118
87,89948118
87,90038907
87,91626033
87,92310977
87,92913138
87,94187308
87,94187308
87,94391764
87,95748924
87,95775511
87,97463658
87,97465519
87,97465519
87,97539017
87,97539017
87,98848811
87,98848811
87,99234597
87,99814679

88,01375303
88,02192216
88,02515896
88,03754934
88,04225182
88,04441286
88,04538655
88,05707252
88,06152106
88,07459097
88,08521793
88,08956477
88,09408323
88,09424688
88,09452707
88,09569844
88,10492849
88,10492849
88,10716776
88,10716776
88,11372466
88,11684379
88,11684379
88,12501242
88,13430905
88,13488616
88,15261683
88,15546968
88,15546968
88,16555914
88,16555914
88,18371615
88,18946569
88,19380895
88,19874163
88,20112209
88,20112209
88,20267336
88,2120355
88,21835172
88,23204481
88,23618915
88,24933954
88,26408184
88,26816271
88,27306054
88,28861967
88,31009436
88,31458327
88,31458327

88,31888415
88,33056671
88,33834406
88,35329313
88,36989688
88,3925041
88,3925041
88,39636568
88,39939934
88,40146412
88,40341316
88,40873888
88,41146706
88,42563921
88,43744116
88,43744116
88,44389563
88,44393099
88,4478267
88,44804994
88,45589547
88,46296978
88,46546774
88,46598141
88,48322211
88,49918895
88,50024028
88,50148144
88,50265072
88,50504309
88,50520719
88,51854085
88,52381205
88,52531824
88,52531824
88,52664844
88,54200093
88,54624942
88,54712279
88,56029764
88,56609224
88,5685617
88,5687314
88,57137947
88,57282886
88,57368091
88,57541282
88,58085886
88,58483602
88,58601973

88,59661886
88,59661886
88,59802581
88,59853712
88,60722623
88,6115463
88,6277265
88,6290648
88,63051399
88,63737999
88,64041533
88,65323212
88,65540922
88,65540922
88,65902327
88,69290951
88,69290951
88,7045861
88,70674544
88,70674544
88,7225415
88,7226314
88,7226314
88,72579855
88,73072817
88,73230409
88,73635029
88,7383178
88,73831944
88,77167516
88,78129813
88,78129813
88,78303737
88,78303737
88,78945583
88,79794269
88,80524753
88,80893987
88,8097076
88,8097076
88,83156525
88,83817491
88,84176174
88,85904995
88,87363802
88,87363802
88,87507444
88,87543124
88,88420323
88,89931799

88,89970768
88,89970768
88,93494647
88,94325401
88,94662277
88,95275545
88,96629435
88,96887366
88,96954502
88,97963937
88,97963937
88,99379478
88,99379478
89,00262451
89,00462777
89,00636313
89,00636313
89,01054541
89,01540778
89,0158436
89,02164559
89,03270154
89,03296505
89,03474842
89,04168683
89,07030823
89,07030823
89,07790447
89,07975549
89,09306786
89,10184468
89,10373204
89,10973829
89,11768122
89,12210975
89,13112138
89,15330125
89,15330125
89,16728782
89,17277372
89,17927658
89,17927658
89,18730451
89,18730451
89,20535669
89,2178102
89,22220697
89,23124593
89,23124593
89,23391686

89,2365319
89,25097467
89,26894716
89,29965485
89,30604388
89,33315974
89,34321688
89,35078442
89,37336867
89,3819661
89,3819661
89,38514482
89,40102575
89,41075377
89,41230914
89,42765778
89,43926757
89,43926757
89,43946041
89,45690917
89,45690917
89,45952743
89,46110387
89,46589158
89,46700192
89,46700192
89,46977667
89,47031275
89,47031275
89,47338805
89,47481708
89,47481708
89,48854228
89,48869767
89,49864787
89,52191673
89,52191673
89,52300983
89,52982764
89,52982764
89,53190787
89,53572819
89,53890987
89,54500999
89,54500999
89,54721096
89,54953299
89,54953299
89,55885534
89,56061224

89,59347305
89,6145681
89,6277238
89,63277006
89,64529394
89,64529394
89,64865086
89,64865086
89,65151149
89,65893848
89,67201947
89,67521638
89,6783538
89,68741824
89,69163755
89,70464745
89,70464745
89,70508363
89,7086009
89,70920825
89,71048012
89,71048012
89,74891753
89,75020637
89,75099289
89,76968443
89,78444338
89,78996053
89,78996053
89,80863388
89,81320895
89,81320895
89,81840164
89,81954098
89,8215482
89,82946873
89,84674362
89,85266929
89,86304301
89,86811782
89,86811782
89,87557074
89,87822528
89,87822528
89,87915502
89,87924963
89,88266907
89,8830718
89,8830718
89,88325443

89,88387801
89,88849198
89,91833808
89,91915168
89,9268979
89,9268979
89,92862886
89,92930145
89,92930145
89,9337055
89,94244519
89,94244519
89,94849943
89,95271616
89,95271616
89,95793
89,96003416
89,9661931
89,98457874
89,98457874
89,98472696
89,99069374
