# Supplementary material for: Cyclic stretch induces autophagy-mediated focal adhesion remodeling and activates mitochondria
Source: Life Sci Alliance. 2026 Feb 5;9(4):e202503347. doi: 10.26508/lsa.202503347 (PMC12877405; doi:10.26508/lsa.202503347)
Supplement: Supplementary file 7 [file LSA-2025-03347_SdataF7.pdf]

Source data Figure 7C

JC-1 [red/green fl.]

cccp	unstr.	4h str.
0,111295	0,794005	0,641026
0,153955	1,301702	0,485211
0,155073	0,570581	0,561842
0,131949	0,600291	0,455677
0,126474	0,563484	0,633185
0,158419	0,691046	0,599387
0,112842	0,69727	0,709691
0,125491	0,660465	0,533528
0,121332	0,655107	0,614238
0,117008	0,910875	0,756151
0,328344	0,869824	0,834812
0,320912	0,492889	0,657963
0,406087	0,738679	0,770875
0,369845	0,613944	0,777737
0,339869	0,804987	0,632302
0,463757	0,62843	0,804995
0,456342	0,594933	0,741622
0,420554	0,733478	0,725
0,457938	0,766606	0,77551
0,413194	0,50387	0,712601
0,477672	0,334413	0,972488
0,484586	0,432842	0,939729
0,472228	0,419592	0,752591
0,407535	0,446541	1,130641
0,346645	0,278555	1,219057
0,370603	0,385533	1,133378
0,459021	0,439495	1,041871
0,36966	0,574285	1,026447
0,466898	0,692635	
	0,551507	
	0,65045	
	0,617391	
	1,002183	
	0,594463	
	0,596889	
	0,589674	
	0,681894	

Source data Figure 7E
actin orientation [°]

unstr. (a)	unstr. +oligomycin (b)	1h str. (c)	1h str. + oligomycin (d)	4h str. (e)
0,056400441	0,012167	8,88794695	0,147588	4,734639
0,087793421	0,020399	20,08913075	0,467315	11,01564
0,113486584	0,148682	44,67285082	0,702951	23,543
0,158488091	0,355316	45,98882527	1,217355	26,40953
0,199576425	0,829318	49,27748102	1,309067	34,90258
0,316868033	0,964484	50,03834492	1,751282	35,77027
0,371875677	1,004315	50,11798178	2,129308	36,78029
0,483876747	1,006279	51,36788225	2,645586	44,93881
0,52100722	1,029605	51,97090658	6,533798	45,05081
0,653010777	1,19789	52,51705223	7,723703	47,48032
0,724349348	1,198467	52,54160216	8,004984	47,52014
0,747312258	1,246995	54,61975244	14,01391	48,53097
0,774064069	1,644213	55,15272609	15,76492	51,97891
0,870741313	1,676192	55,62470763	20,62151	52,49985
0,934966221	1,696155	56,48436693	23,77635	53,77923
0,964549171	1,773705	56,59641699	24,04665	54,93483
1,035268309	1,912134	57,00852203	36,13459	54,93675
1,119365651	2,108288	57,1653197	38,39454	55,15839
1,168488453	2,153926	57,98324274	39,68569	55,53506
1,288377704	2,226959	58,25922907	40,92775	56,67897
1,419491327	2,254715	58,29618039	41,25052	56,68009
1,518758429	2,285509	58,50937477	43,53109	58,26967
1,559373642	2,314613	58,57527464	44,00512	58,47229
1,598442904	2,35812	58,71840346	44,24453	59,62981
1,600619307	2,436982	59,03223475	45,39656	59,908
1,623618539	2,459849	59,0677786	46,29728	60,06405
1,654495625	2,465041	59,0998455	46,40044	60,59396
1,782454017	2,599089	59,16466726	46,52051	61,39686
1,789586607	2,621038	59,23708414	46,5648	62,64945
1,799547797	2,716935	59,35259541	47,81733	63,05878
1,823256989	2,766237	59,80637374	47,83964	63,76238
1,873175942	3,020062	60,27422419	48,57033	63,96001
1,88656503	3,100384	60,32114441	49,496	64,20643
1,906333186	3,30336	60,73525043	50,01754	64,52089
1,960445468	3,384555	60,93099074	50,75346	64,71199
2,067792215	3,578594	60,94196681	51,9355	65,1821
2,069538803	3,645954	61,24891529	52,13261	65,33017
2,21615157	3,676723	62,10162161	52,40124	66,70961
2,273576416	3,783791	62,18366732	52,8842	66,71045
2,309130895	3,814633	62,20232167	52,93374	67,20213
2,346621623	4,495504	62,88105251	54,18607	67,20251
2,371126303	4,593518	63,14992463	54,40987	67,70498
2,466726315	4,600912	63,24808102	54,49719	67,78505
2,512870812	4,684551	63,43309772	54,75627	67,93524
2,528192752	4,740492	63,57085039	55,01778	67,94129
2,702254111	4,777226	63,69942899	55,04379	68,41674
2,752685672	4,793917	64,00558548	55,14615	68,42275

2,813167398	4,855408	64,16941883	55,15193	68,83304
2,826664036	5,019321	64,53629995	55,38842	69,07567
2,851138173	5,044916	64,93058505	55,69867	69,19892
2,926350057	5,203143	64,9561969	55,86471	69,21234
2,943853138	5,209729	65,00772327	55,96779	69,3418
3,095357244	5,260293	65,0664482	56,16375	69,64668
3,096467107	5,265814	65,1384869	56,2005	69,95361
3,273464264	5,57998	65,66795525	56,33103	70,07607
3,275459987	5,809052	65,68283084	56,83608	70,22625
3,463199874	6,117687	65,68652626	57,31431	70,74302
3,506142294	6,160376	65,76063281	57,41536	70,76207
3,592159862	6,164645	65,86418577	57,68234	71,33471
3,735993445	6,312206	65,91560356	57,81264	71,34884
3,755887842	6,553793	65,94200159	58,03408	71,54596
3,756583235	6,580377	66,16830097	58,05851	71,92771
3,943285113	6,832648	66,19708278	58,08602	72,40793
3,985605648	7,050216	66,22346437	58,32053	72,43191
4,15399922	7,052438	66,26360586	58,78465	72,43403
4,273203036	7,058746	66,60025582	59,13039	72,56871
4,29918053	7,267729	66,79225219	59,47469	72,71604
4,304837668	7,352895	66,98548236	59,58502	72,92998
4,334591849	7,480062	67,19911963	59,70158	73,45708
4,357815849	7,552906	67,38631216	59,83424	73,49175
4,622416403	7,577398	67,49658825	59,84867	73,6322
4,976542469	7,653009	67,52012094	60,45313	73,6344
4,994149281	7,741527	67,52195798	60,68726	73,75169
5,159384822	7,951412	67,59595366	60,74764	73,98894
5,182230662	8,097074	67,66355645	61,26114	74,0946
5,235520239	8,221804	67,97915715	61,51702	74,30583
5,256860903	8,526656	68,01934938	61,88582	74,4844
5,296302561	8,742289	68,0478483	62,06452	74,58399
5,656009662	9,12864	68,14008069	62,17574	74,78466
5,712628102	9,214387	68,14694814	62,29588	74,79922
5,749984379	9,543788	68,39389521	62,47372	75,00662
5,967070434	9,555758	68,5914945	62,83851	75,06532
5,99091686	9,684861	68,6593198	62,97283	75,07143
6,072046248	9,697187	68,7483361	63,18057	75,15833
6,104527796	9,71492	68,94745618	63,37169	75,31737
6,404757237	9,877498	69,10721697	63,47259	75,37189
6,45420908	10,15939	69,21113864	63,69948	75,65732
6,690858269	10,35691	69,27291693	63,72425	75,65826
6,76464669	10,38704	69,47430193	64,04836	75,66691
6,842725807	10,40506	69,50082864	64,11427	75,69513
6,926530491	10,60698	69,54519279	64,12878	75,7678
7,518310805	10,6165	69,62543247	64,21012	75,99396
7,541429538	10,75209	69,65164375	64,33668	76,00279
7,582613846	10,7819	69,93900429	64,43703	76,23194
7,614477848	10,7914	70,08692265	64,47646	76,25041
7,638520497	10,84087	70,11800823	64,66283	76,26587
7,749813227	11,20683	70,16977029	64,80977	76,33554

8,891068499	11,51071	70,21093494	64,95229	76,35875
9,037510257	11,89417	70,24756259	65,07933	76,39487
9,256162823	11,91995	70,35562065	65,09529	76,43566
9,388322786	12,16848	70,35855853	65,18673	76,44577
9,425038105	12,26291	70,42236644	65,25231	76,44855
9,465264671	12,32251	70,45358555	65,2834	76,48318
9,497526472	12,5581	70,61330339	65,36318	76,65728
9,527198374	12,61822	70,65959932	65,38702	76,70981
9,653554064	12,66125	70,80460409	65,48269	76,73683
10,05548189	12,71488	70,87622248	65,69709	76,74835
10,13430865	12,95217	70,94183778	65,73649	76,79575
10,21267827	12,9722	70,9621411	65,90388	76,93878
10,3556703	13,22111	71,06117782	66,24293	76,97103
10,36032318	13,26963	71,071245	66,35608	76,98597
10,6738797	13,28822	71,09854169	66,49848	77,0629
10,69447462	13,92652	71,11297118	66,81027	77,09592
10,78098994	14,1085	71,12914137	66,85912	77,2055
10,79733633	14,20944	71,17071833	67,06534	77,2267
10,89634017	14,30286	71,19719014	67,15097	77,29638
10,91530912	14,34628	71,20696841	67,17371	77,33129
11,05716971	14,39906	71,29797161	67,26046	77,3505
11,10579103	14,41306	71,33584284	67,33617	77,53056
11,23280363	14,43464	71,41217796	67,7438	77,58784
11,36806028	14,47849	71,43165046	67,88614	77,58946
11,59815555	14,53195	71,43302219	68,0315	77,62399
11,63978114	14,64642	71,58831824	68,09417	77,78701
11,68936815	14,80793	71,59945744	68,14018	77,81935
11,6960631	14,89455	71,62981319	68,36642	77,83897
11,89796743	14,93241	71,77387728	68,41881	77,84767
11,92554869	14,95917	71,79703287	68,49305	77,86807
11,94900499	15,04885	71,91687568	68,65835	77,90722
12,03042801	15,17084	72,09680707	68,77526	77,91249
12,42355333	15,18695	72,12355928	68,89111	77,94086
12,54662259	15,39586	72,30061021	69,05624	77,98782
13,19706035	15,66037	72,42511281	69,22855	78,09376
13,42824536	15,86976	72,42979242	69,25048	78,12482
13,48457081	15,87379	72,43135016	69,43785	78,12789
13,56565246	16,20714	72,48947791	69,62072	78,12915
13,67283065	16,30088	72,69163435	69,64875	78,13225
13,79773843	16,54546	72,7224704	70,16461	78,16128
13,96661891	16,57017	72,8365515	70,23825	78,18376
14,11970994	16,596	72,9136401	70,41256	78,27585
14,12965292	16,72228	73,08629915	70,54506	78,38122
14,23788184	16,83625	73,11026572	70,70654	78,39122
14,29718184	17,35446	73,26734036	70,75396	78,4401
14,541809	17,3725	73,33056618	70,83369	78,45007
14,58473084	17,79491	73,45593653	70,92029	78,45452
14,6994285	17,81685	73,54052469	71,11072	78,48532
14,79605842	18,19827	73,6292581	71,40757	78,60371
14,85288337	18,31727	73,75035426	71,66376	78,66987

15,04173198	18,35017	73,76574092	71,73323	78,70331
15,17664529	18,59065	73,87532898	71,93759	78,75496
15,41643203	18,60299	73,97266913	71,95311	78,8597
15,47985025	19,17736	73,99099682	72,00065	78,88899
15,53703675	19,39329	74,07321381	72,0728	78,90926
15,71928423	19,41801	74,17889993	72,12288	78,98322
15,84722231	19,45027	74,27723085	72,721	79,23413
16,06285812	19,50288	74,32134345	73,19404	79,26173
16,12654479	19,5657	74,42263507	73,28311	79,28406
16,24736437	19,83771	74,50442291	73,35894	79,37772
16,37165173	20,21758	74,5493207	73,8003	79,54721
16,39819552	20,25797	74,62061419	73,88765	79,56186
16,67933829	20,29148	74,72538128	74,42004	79,59444
16,95448815	20,63294	74,80408996	74,78159	79,61227
17,1822591	20,86339	74,81864233	74,82872	79,61466
17,76721462	20,89218	74,85934914	74,93591	79,73497
17,80404476	21,04746	75,00335791	75,00619	79,74451
17,97542312	21,50002	75,03892186	75,06273	79,75855
18,04649298	21,7711	75,05649733	75,19671	79,88079
18,08872613	21,93898	75,07191966	75,28698	80
18,12165589	21,99235	75,1284589	75,5054	80,0365
18,60617373	22,06839	75,14883639	75,6308	80,14874
18,84101666	22,07171	75,15291613	75,78024	80,16801
18,85270419	22,12556	75,16788146	76,09054	80,19374
18,88066128	22,27196	75,38265252	76,14723	80,31025
19,02538513	22,28669	75,45314797	76,34609	80,31234
19,27923975	22,31562	75,45421184	76,45612	80,32499
19,40399055	22,36663	75,67008295	76,67136	80,37783
19,52537805	22,73067	75,85516294	76,81278	80,42917
19,71931969	22,74427	75,87329122	76,85211	80,4321
19,75766537	23,01057	75,87890294	76,85731	80,48425
19,79311385	23,05536	75,90826933	77,18396	80,51005
19,82148077	23,1607	75,9723009	77,50219	80,57498
20,40934552	23,29834	76,02761613	77,51418	80,57578
20,52390844	23,44923	76,10719765	77,53187	80,64108
20,59199143	23,58403	76,20890595	77,62381	80,6584
20,5930495	23,58447	76,2328865	77,70604	80,66637
20,77959341	23,70219	76,26033334	77,74646	80,71803
20,83270744	23,72285	76,422694	77,79907	80,76551
20,94812952	23,92237	76,42806343	77,99106	80,80319
21,355462	23,95654	76,43904502	78,26542	80,81068
21,58448077	24,27543	76,65539204	79,07995	80,84536
21,58869458	24,29724	76,66579745	79,14745	80,89003
21,68170205	24,30196	76,75361715	79,15024	80,91302
21,77613455	24,4234	76,80982114	79,19675	80,94802
21,7961385	25,10075	76,92339427	79,2831	80,97186
21,85453141	25,1268	76,93213594	79,32879	80,97207
22,11290453	25,20817	77,01413219	79,3652	81,00239
22,15599444	25,30748	77,05344582	79,39663	81,07576
22,1653744	25,46478	77,0642311	79,54697	81,15378

22,40978176	25,51187	77,06450699	79,57148	81,17664
22,9333723	25,76284	77,11449453	79,70509	81,19061
23,05070219	25,81723	77,13901194	79,77595	81,27747
23,1496297	25,89335	77,1886051	79,81612	81,31897
23,38418119	25,90371	77,21298162	79,87975	81,36674
23,62696294	25,93794	77,21782699	79,95815	81,39466
23,83780303	26,11893	77,30339926	80,06886	81,4608
23,90700278	26,13392	77,35937806	80,08463	81,50739
24,25090673	26,19108	77,37290076	80,31688	81,63628
24,26813879	26,69408	77,37293658	80,37555	81,63901
24,4012325	27,10469	77,4138886	80,39303	81,6452
24,52148325	27,13547	77,43260581	80,4735	81,65691
24,52383282	27,15745	77,51429904	80,49576	81,66873
24,55953959	27,5119	77,54876177	80,63118	81,74258
24,60662745	27,61707	77,56757769	80,73953	81,84971
24,64583585	27,65372	77,57002756	80,87283	81,88479
24,91452941	27,78996	77,57793557	80,87639	81,95356
24,9754008	27,80522	77,58046126	80,88228	82,08055
25,07135447	27,88333	77,58412817	81,03228	82,0901
25,15653768	28,11811	77,64775033	81,03543	82,10191
25,23895137	28,18932	77,68573968	81,05924	82,16291
25,47607219	28,41371	77,72582025	81,48138	82,17165
26,00212844	28,47115	77,89522483	81,51696	82,18214
26,20084673	28,51154	77,91898245	81,60767	82,21084
26,21006823	28,54823	77,96973681	81,61832	82,21291
26,21498755	28,63973	77,97338128	81,75233	82,28399
26,2852087	28,66272	77,97991185	81,77539	82,29886
26,34164016	29,09186	77,99761692	81,79172	82,30692
26,38936869	29,10225	78,01162069	81,94983	82,35704
26,41242591	29,25945	78,03466989	82,05817	82,40877
26,73805828	29,56935	78,05396328	82,18539	82,42154
26,8263924	29,58128	78,11610797	82,29219	82,47963
26,83368687	29,64434	78,14944123	82,31343	82,5019
26,87268726	29,77752	78,18740268	82,38667	82,51567
26,91863781	29,9659	78,29096818	82,48719	82,53196
26,94335042	30,15665	78,29186327	82,48853	82,56913
27,03259626	30,31123	78,31067101	82,57381	82,57127
27,10506862	30,33506	78,38522266	82,68597	82,58336
27,47613683	30,4966	78,39991326	82,73981	82,58438
27,48792526	30,64864	78,41962239	82,75911	82,60205
27,55355295	30,69494	78,45208781	82,78395	82,60949
27,56697739	30,82331	78,47917346	82,844	82,84391
27,89800008	30,85239	78,52355316	83,01023	82,88071
27,93104317	30,98808	78,52920687	83,02785	82,95831
28,14494764	31,05611	78,55974583	83,04162	82,96606
28,19927058	31,06561	78,59133246	83,05696	82,99731
28,23391315	31,08785	78,62405485	83,12159	83,00715
28,81198258	31,09148	78,62487702	83,20764	83,02413
28,9064184	31,10005	78,68721405	83,28496	83,06857
29,07764826	31,21517	78,70036858	83,38563	83,0958

29,3071846	31,32332	78,74565387	83,38995	83,09908
29,32959676	31,37209	78,76193985	83,39476	83,12576
29,493379	31,87184	78,91580445	83,45479	83,22723
29,65367826	31,87549	78,99407003	83,45723	83,24428
29,9259311	31,98264	79,026561	83,51395	83,27308
30,16278512	31,99097	79,06737859	83,64244	83,29796
30,34036616	32,22114	79,09826038	83,66234	83,31424
30,37687951	32,36028	79,11928188	83,71737	83,31472
30,5101289	32,36315	79,23269693	83,75629	83,36026
30,62897273	32,50245	79,25905309	83,8084	83,38587
30,64758112	32,81881	79,32352852	83,85551	83,4581
30,98835801	32,92835	79,32431992	83,8745	83,47833
31,09424559	32,94761	79,47846808	83,98115	83,64102
31,37913287	33,09506	79,48332404	83,98339	83,70392
31,43662942	33,25148	79,51675067	84,03643	83,70571
31,67762084	33,26792	79,56009537	84,26374	83,73788
31,6978902	33,34026	79,63724829	84,32271	83,77784
31,77352983	33,3569	79,86187537	84,47094	83,80799
31,86094877	33,56685	79,96265995	84,49128	83,91468
31,86831596	33,71361	80,07201927	84,51202	83,91745
31,97249156	33,94487	80,1141778	84,536	83,94619
32,07884487	33,99369	80,17096719	84,58418	83,97718
32,1373161	34,20053	80,19537403	84,64583	84,12652
32,30166702	34,20466	80,20243859	84,72035	84,14509
32,47809759	34,82353	80,22118732	84,73251	84,14651
32,60731316	34,86452	80,23412574	84,73883	84,18138
32,85549824	35,16315	80,25975449	84,75278	84,19664
33,07370319	35,23389	80,28118256	84,75656	84,22296
33,09134327	35,29781	80,29528463	84,81946	84,32017
33,4089655	35,405	80,30460335	84,83145	84,3311
33,47634143	35,40756	80,30665297	84,87071	84,33265
33,48062449	35,63621	80,37880606	84,89876	84,3636
33,54683221	35,6418	80,42495867	85,01249	84,37452
33,67419477	35,69626	80,42568581	85,03795	84,44791
33,68065718	35,71638	80,42611739	85,04224	84,54096
33,71670051	35,87862	80,54090422	85,12795	84,57763
33,84465956	35,92636	80,56337965	85,17735	84,57822
33,84734093	36,24972	80,61895326	85,33441	84,5865
33,87470496	36,35731	80,69042132	85,39976	84,63819
33,91148457	36,59711	80,70533666	85,52384	84,64735
34,0653815	36,61747	80,85332344	85,52429	84,70924
34,15031399	36,85068	80,89186603	85,56266	84,7412
34,41375553	36,96369	80,93181043	85,57016	84,74794
34,79528959	37,20448	80,95001834	85,59682	84,78171
34,82480803	37,21867	80,99739774	85,67362	84,79729
34,83939496	37,22672	81,02271853	85,68307	84,80505
35,00536576	37,72641	81,0615697	85,70908	84,8543
35,06556127	37,89982	81,07395555	85,71127	84,87073
35,22693207	37,94232	81,19944588	85,73151	84,89937
35,27411255	38,19354	81,2096027	85,73172	84,91926

35,4262852	38,26458	81,33608379		85,8082	84,93815
35,56928459	38,35663	81,3691548		85,83076	84,96102
35,597965	38,93832	81,41868125		85,83613	84,96751
35,79928651	38,95731	81,43795505		85,88805	84,97829
35,83470561	39,09903	81,46215266		85,93018	84,98489
36,12667162	39,30337	81,47556476		86,01029	85,05074
36,31367574	39,379	81,51077875		86,01293	85,07152
36,37894599	39,7849	81,6201239		86,11355	85,07401
36,42595819	39,81626	81,63349532		86,11707	85,1182
36,46786902	39,86754	81,66043951		86,15036	85,14871
36,54334866	39,98536	81,78541701		86,16717	85,15477
36,831049	40,07713	81,80784139		86,1801	85,15802
36,85108816	40,18157	81,81774689		86,24389	85,15996
36,90485018	40,2121	81,90177		86,31675	85,16319
37,17595973	40,42039	81,94645322		86,38887	85,19846
37,46670467	40,48161	81,96549816		86,47971	85,21247
37,47156248	40,51047	81,98056542		86,53651	85,21596
37,65699059	40,81777	82,0053271		86,56848	85,26017
37,66827702	40,84113	82,07492259		86,5863	85,27414
37,70525874	40,9821	82,08351162		86,61041	85,28118
37,78879529	41,00646	82,08801406		86,73498	85,29205
38,1424417	41,19415	82,10246295		86,7501	85,29268
38,15381287	41,27841	82,12326542		86,85728	85,30633
38,52737855	41,28698	82,16642941		86,9832	85,31214
38,85991467	41,29895	82,2487496		87,00934	85,33118
38,93558729	41,30273	82,36670572		87,09011	85,33269
39,15686711	41,37551	82,42500867		87,09623	85,35825
39,23816735	41,46726	82,44266546		87,12856	85,35891
39,29664768	41,73577	82,45061201		87,13291	85,42836
39,54301681	41,81889	82,45223576		87,1579	85,53233
39,61898426	42,01738	82,46779456		87,16559	85,53538
39,78978548	42,24983	82,48439466		87,19947	85,57078
39,93210059	42,31309	82,55598375		87,20331	85,57121
39,98159606	42,3586	82,59166553		87,43705	85,58166
40,00357507	42,36929	82,59312213		87,4742	85,61248
40,07526026	42,43855	82,60782336		87,47611	85,63446
40,10188649	42,46606	82,62372572		87,48474	85,6683
40,36008244	42,64112	82,70774269		87,52227	85,68304
40,36565294	42,655	82,77279958		87,52754	85,71026
40,43440989	42,70283	82,78450444		87,56163	85,72081
40,49994753	42,76407	82,87614686		87,57711	85,73753
40,54517524	42,76669	82,8805563		87,57736	85,74957
40,58950241	42,8208	82,93398336		87,60256	85,78738
40,61496698	42,82231	82,95593416		87,61092	85,79369
40,92451547	42,95024	83,03702481		87,66551	85,8035
40,96729178	43,0312	83,13510113		87,66648	85,82849
41,12083608	43,08635	83,24499588		87,67387	85,83956
41,15434506	43,15114	83,24918925		87,69606	85,84722
41,15874065	43,34493	83,27180804		87,71731	85,88547
41,24977264	43,5019	83,28247864		87,71936	85,94112

41,36041297	43,56748	83,36478532	87,77512	85,94284
41,40371993	43,75618	83,38122513	87,77866	85,99333
41,42158907	43,80274	83,38657328	87,78719	86,00964
41,49780278	43,92335	83,45431092	87,79459	86,01215
41,50509169	44,01244	83,46282329	87,81085	86,03407
41,8388804	44,30475	83,53804947	87,82244	86,06463
41,85004987	44,31741	83,57952113	87,85509	86,0986
42,20568442	44,34291	83,58503342	87,8832	86,10626
42,24760372	44,41812	83,58751225	87,90189	86,11256
42,35842917	44,43668	83,60079283	87,93517	86,14073
42,55347707	44,48003	83,65381809	87,93929	86,15293
42,56673537	44,59446	83,67398241	87,99339	86,15547
42,57267186	44,63434	83,67759982	88	86,18017
42,58297953	44,6476	83,72568809	88,01564	86,18907
42,63042874	44,67618	83,74893931	88,08002	86,20436
42,92912524	45,04522	83,79422197	88,08051	86,21536
42,93070648	45,34772	83,80090271	88,10402	86,22392
43,08686777	45,42751	83,89672683	88,13477	86,2259
43,1481751	45,75716	83,90584852	88,14438	86,24251
43,17330402	45,79631	83,92175543	88,14806	86,24256
43,36439663	45,87062	83,92240419	88,15155	86,2726
43,36719139	46,02211	83,94640946	88,15533	86,28364
43,63372235	46,26727	83,9588541	88,18628	86,28894
44,16234726	46,54631	84,00387658	88,20171	86,2962
44,25751767	46,59751	84,03066622	88,25953	86,30424
44,26173061	46,61117	84,04306796	88,28526	86,3099
44,31000646	46,62386	84,07646028	88,31147	86,3699
44,36346882	46,80751	84,07718671	88,32003	86,39681
44,62769881	47,08511	84,07732886	88,32886	86,40524
44,66772479	47,17517	84,12509638	88,32888	86,40712
44,77402898	47,2234	84,20027562	88,35657	86,40821
45,02535961	47,34985	84,21631761	88,38877	86,41411
45,17208987	47,40125	84,22786838	88,45765	86,44461
45,24936967	47,43708	84,31764904	88,46678	86,44721
45,34335591	47,67501	84,3180527	88,51918	86,50459
45,51356259	47,73329	84,31885251	88,52113	86,51454
45,52316686	47,76657	84,4029092	88,5346	86,54679
45,54997853	47,77442	84,41492726	88,55905	86,63746
45,63147886	48,16449	84,49066161	88,59592	86,66282
45,73389197	48,2347	84,5098397	88,60478	86,68467
45,8461412	48,26221	84,52213657	88,63789	86,69462
45,88379469	48,2663	84,54879172	88,64929	86,70814
45,92322909	48,38992	84,62138072	88,67754	86,72343
45,93089546	48,50535	84,73530597	88,70315	86,73978
45,95929583	48,55016	84,7602137	88,72645	86,75103
46,02364136	48,7886	84,77738692	88,73418	86,76977
46,10522598	48,97227	84,82096521	88,75085	86,78632
46,13403457	49,29449	84,86516389	88,75824	86,80923
46,17461274	49,31893	84,90141546	88,80848	86,82251
46,17835027	49,57118	84,93700058	88,88543	86,84058

46,37339133	49,68576	84,97021603	88,90352	86,86
46,41782428	50,0181	84,99818098	88,90668	86,88557
46,46225478	50,07981	84,9989878	88,91437	86,90198
46,59967007	50,08837	85,09667666	88,97217	86,90475
46,71814893	50,09006	85,11936116	88,98983	86,90785
46,83251748	50,10212	85,12830372	89,02658	86,95752
46,86274343	50,19698	85,19036368	89,0539	86,98772
46,90982987	50,31623	85,28020704	89,11055	86,99242
46,91609097	50,34912	85,33388521	89,11377	87,0071
47,00507291	50,45072	85,38450418	89,11616	87,02777
47,04237173	50,85804	85,38760574	89,16821	87,03865
47,13907288	50,86806	85,39273075	89,24269	87,05614
47,14209649	50,98328	85,41750317	89,33511	87,0834
47,14553383	51,08342	85,45673747	89,42917	87,11193
47,23683276	51,22056	85,46635254	89,45002	87,11475
47,36283638	51,36047	85,46896153	89,48909	87,11914
47,44477789	51,40013	85,4994448	89,50662	87,13516
47,45197207	51,42944	85,54579965	89,51042	87,14033
47,68272199	51,51608	85,69991123	89,51864	87,15797
47,68495431	51,59706	85,73776689	89,53314	87,17415
47,73280771	51,61209	85,75351843	89,56734	87,17539
47,96655079	51,81282	85,7798648	89,57796	87,22091
48,16492988	51,8982	85,88981099	89,66134	87,23263
48,45935756	52,08366	85,90192326	89,69722	87,23722
48,53573531	52,16888	85,91235637	89,71674	87,29041
48,58573844	52,60458	85,95391819	89,73597	87,30508
48,67481649	52,78272	85,96225882	89,78307	87,31521
48,67922041	52,87474	85,97049096	89,78314	87,32057
48,83353453	53,08342	86,0138508	89,79001	87,33657
48,91012632	53,22385	86,03860779	89,8291	87,35399
48,94074567	53,34029	86,06052196	89,85958	87,35579
49,09093221	53,37165	86,08809747	89,86266	87,35661
49,10481219	53,44795	86,1525346	89,86729	87,36198
49,16110246	53,45667	86,1718969	89,90061	87,37074
49,28036815	53,59208	86,22154883	89,92129	87,39271
49,55948001	53,73543	86,26837269	89,9357	87,39361
49,67745956	53,8882	86,29163029	89,95961	87,43493
49,75689909	54,04886	86,29949163	89,98702	87,4404
49,90027194	54,13334	86,30737096	90	87,45617
49,90789431	54,21387	86,32457769	90	87,48814
49,95180911	54,28459	86,35120157	90	87,49003
49,96156524	54,32255	86,35579092	90	87,54138
50,02859634	54,35429	86,43597329	90	87,5762
50,40321887	54,4513	86,47193849	90	87,57837
50,41259027	54,48923	86,50060659	90	87,57857
50,7027922	54,71626	86,54096031	90	87,59495
50,80026524	54,78267	86,59054949	90	87,62355
50,82956905	55,0236	86,62478998	90	87,63001
50,99155381	55,05958	86,63865621	90	87,63228
51,1033774	55,13867	86,65746089	90	87,66572

51,31619505	55,3235	86,66960714	90	87,66599
51,39977617	55,59139	86,68095955	90	87,70109
51,41946003	55,59834	86,6878774	90	87,70395
51,60929188	55,94686	86,71357621	90	87,72049
51,75462784	55,98286	86,73609936	90	87,72619
51,88120146	56,02315	86,76220063	90	87,73244
52,26484692	56,38722	86,79417375	90	87,74467
52,30751812	56,51481	86,83777176	90	87,74826
52,41848866	56,53772	86,84319279	90	87,75392
52,48800642	56,71053	86,85828674	90	87,77973
52,59903518	56,73608	86,88771093	90	87,7805
52,60571906	56,85167	86,88778455	90	87,78592
52,77276316	56,98316	86,9013608	90	87,81155
53,15598637	56,98633	86,90461224	90	87,81329
53,18560328	57,04311	86,92892984	90	87,81408
53,55301685	57,19966	86,93040676	90	87,83474
53,56628409	57,24767	86,94692982	90	87,83689
53,60217802	57,39098	86,96549771	90	87,84117
53,61282636	57,52243	87,00947301		87,87048
53,75174342	57,5449	87,08604937		87,88065
53,92340864	57,65121	87,10008239		87,89493
54,04699968	57,82741	87,1454668		87,89795
54,12000884	57,92244	87,15556858		87,90532
54,45446596	57,99987	87,1977236		87,90958
54,75690537	58,1437	87,20860823		87,91484
54,7621676	58,25901	87,2475555		87,94315
54,87026109	58,67127	87,2713538		87,94412
54,87779257	58,67217	87,27917518		87,96564
54,9924352	58,83051	87,28101934		87,97606
55,09845294	58,88339	87,28830131		87,99616
55,18089025	58,90175	87,30521021		88,07354
55,40260893	59,12378	87,30617634		88,10758
55,42986591	59,24288	87,35687268		88,12329
55,53620746	59,47112	87,39210421		88,12557
55,60431113	59,49772	87,41946992		88,13549
55,81897924	59,53561	87,43290041		88,23642
55,93918854	59,54021	87,51131561		88,23982
55,97839851	59,54184	87,53082604		88,24188
55,98507464	59,69899	87,5770775		88,24199
56,07090451	59,8242	87,61261763		88,26315
56,10411892	59,95535	87,62466922		88,31465
56,1623879	60,09586	87,69793166		88,31686
56,35607033	60,18816	87,70244958		88,32907
56,36072597	60,39285	87,75129062		88,33354
56,36319982	60,53437	87,76094493		88,34776
56,59329543	60,55735	87,76655291		88,36307
56,67512564	60,58701	87,7857126		88,36563
57,03822569	60,74841	87,81577213		88,39347
57,25038975	60,79889	87,81660644		88,39673
57,26991267	60,86209	87,82223287		88,41158

57,35747256	60,88813	87,82307782	88,43123
57,58925415	60,89996	87,83143428	88,45226
57,66166603	61,08159	87,83560213	88,46011
57,66661194	61,25057	87,83631842	88,49013
58,17247126	61,46439	87,90296836	88,49487
58,2487251	61,50059	87,91751747	88,49775
58,56777451	61,50447	87,92887791	88,51331
58,57174785	61,56877	87,9443724	88,52164
58,62320151	61,71585	87,9482875	88,52628
58,66647184	61,71761	88,00175179	88,56385
58,67586137	61,73057	88,02986957	88,56523
58,93399943	62,10834	88,04300095	88,56874
58,96721034	62,17847	88,0646818	88,58587
58,97844808	62,21022	88,08017903	88,60662
59,04124639	62,29793	88,09210135	88,62356
59,04211188	62,30636	88,16961482	88,63884
59,1589318	62,32424	88,17455628	88,6419
59,52943881	62,4432	88,20227563	88,64234
59,60171499	62,51344	88,246072	88,65182
59,95041629	62,60255	88,26923528	88,65771
59,97405629	62,63757	88,28702509	88,65838
60,29955228	62,73405	88,29296411	88,67235
60,44116146	62,92351	88,30716458	88,67593
60,49201563	62,95272	88,36290003	88,67914
60,49309059	63,06321	88,36711695	88,68654
60,5325688	63,11923	88,38031708	88,70156
60,70315956	63,16829	88,39025248	88,73078
60,71664311	63,34362	88,42463963	88,7466
60,83527472	63,40388	88,42608867	88,7555
60,8802495	63,59754	88,42744236	88,75926
61,05342518	63,90165	88,44707759	88,76094
61,12621056	63,95506	88,45658485	88,76781
61,23845886	64,02699	88,52235694	88,7711
61,84728975	64,03115	88,54327228	88,78794
61,91306033	64,07463	88,56295037	88,81691
62,00136625	64,11029	88,57174757	88,81798
62,01407725	64,15733	88,58973989	88,82142
62,4654461	64,31697	88,6073201	88,83257
62,51742512	64,52771	88,67843989	88,84898
62,65770286	64,72515	88,71292906	88,85694
62,78833043	64,78738	88,73002167	88,86674
62,9808186	64,80515	88,73936296	88,88254
63,19616621	64,99472	88,74638756	88,88836
63,20232489	65,02334	88,76124556	88,89571
63,58880668	65,44935	88,76621979	88,89618
63,60392434	65,5561	88,78922515	88,90698
63,67884581	65,58478	88,80029483	88,92332
63,76791735	65,65706	88,81600469	88,92414
63,81668849	65,74836	88,84053247	88,93525
64,00151321	65,77146	88,86089593	88,97642

64,08320322	65,97483	88,86339323	88,98818
64,13356903	66,19885	88,86594148	88,99683
64,20286812	66,398	88,88475982	89,01729
64,37290633	66,52389	88,89800699	89,02423
64,53530612	66,55026	88,90632097	89,03508
64,74583594	66,75283	88,92439152	89,03527
65,07620628	66,76645	88,97254929	89,04558
65,328842	66,78818	89,02049283	89,04724
65,34857157	66,85225	89,02731328	89,05809
65,41150766	66,8723	89,03885182	89,07116
65,44011752	66,87705	89,07817404	89,07464
65,72638569	67,16033	89,08255235	89,09276
65,79115147	67,16543	89,10184622	89,10001
65,94581522	67,19055	89,11967006	89,10663
66,03085244	67,19102	89,12439933	89,10668
66,248752	67,23185	89,12453687	89,12188
66,47685489	67,38304	89,13826114	89,12528
66,61812576	67,44105	89,1410705	89,13254
66,63815548	67,64692	89,16827001	89,14981
66,66720184	67,71441	89,21191245	89,15075
66,70695149	67,81669	89,22799515	89,17392
67,06702987	67,98019	89,23624731	89,18448
67,15347504	67,98805	89,24008422	89,19644
67,3009795	68,0917	89,2410383	89,2018
67,54707578	68,23448	89,2410748	89,20955
67,87342639	68,31336	89,25519406	89,21301
67,93204512	68,37513	89,38223819	89,21496
68,14773974	68,79463	89,38788656	89,2161
68,18662303	69,14163	89,38875699	89,22574
68,3265246	69,29446	89,39232323	89,24534
68,42038753	69,48801	89,39273586	89,24646
68,48535008	69,52694	89,40103432	89,24739
68,75098308	69,54214	89,44054294	89,25233
68,77617666	69,59588	89,4803332	89,25409
68,8544925	69,9307	89,49561494	89,26353
69,13845726	69,98406	89,52518006	89,31054
69,21117949	70,05973	89,53066506	89,31311
69,2326986	70,1089	89,56874633	89,32107
69,30400545	70,12364	89,58455844	89,32198
69,41781539	70,13937	89,59786241	89,32994
69,7067454	70,15687	89,6048702	89,35539
69,9424749	70,21579	89,60771867	89,36026
70,05172643	70,29862	89,60796927	89,3616
70,2596321	70,36165	89,63704552	89,37484
70,27043674	70,40724	89,64793498	89,38584
70,29661365	70,4914	89,64884055	89,39425
70,31902873	70,65345	89,65279783	89,39481
70,34679509	70,78318	89,65790896	89,40265
70,47112933	71,01519	89,66296545	89,41302
70,71915411	71,21392	89,67388465	89,42111

71,39105237	71,24361	89,68754899	89,44573
71,55217661	71,33664	89,70779145	89,45109
71,6431355	71,67195	89,73067524	89,46258
71,67579255	72,03395	89,73620956	89,47189
71,72098135	72,05643	89,75240566	89,50004
72,51357351	72,14661	89,78512162	89,50663
72,51620389	72,25216	89,88125678	89,50737
72,90379308	72,28094	89,88836886	89,52131
73,22343138	72,3091	89,91564151	89,53873
73,42006197	72,4595	89,92244497	89,54775
73,44959241	72,53086	89,95657028	89,55599
74,23459221	72,64538	89,9649437	89,57078
74,35540105	72,83171	89,97566769	89,57472
74,66427419	72,88215	89,98221803	89,57484
74,70018254	72,99524	89,98656707	89,58363
74,71718529	73,08523	89,98916305	89,58448
74,74745774	73,13221	90	89,58815
74,90892587	73,38474	90	89,61154
74,91571286	73,57591	90	89,61899
75,13496646	73,70083	90	89,63706
75,3270026	73,71177	90	89,63709
75,48944775	73,87365	90	89,65699
75,59079044	74,11706	90	89,6613
75,73261713	74,48709	90	89,67349
75,75081664	74,56717	90	89,69025
76,02959708	74,60654	90	89,69607
76,19496676	74,60709	90	89,7416
76,19805505	74,6716	90	89,74432
76,43643569	74,7595	90	89,74854
76,87746845	74,82661	90	89,75841
77,36439466	74,93148	90	89,76614
77,49866349	74,9431	90	89,76708
77,75915818	75,01326	90	89,76709
77,77572824	75,24856	90	89,78507
77,78907512	75,33072	90	89,78598
77,82199843	75,38473	90	89,78706
77,9213199	75,50461	90	89,81684
78,11108998	75,53341	90	89,82943
78,17335353	75,99299	90	89,83575
78,18177376	76,03092	90	89,86603
78,38934026	76,20896		89,88815
78,4067963	76,29551		89,89863
78,76216322	76,34339		89,927
78,89510843	76,36469		89,93117
78,93612902	76,46195		89,93451
78,9638301	76,4722		89,94812
79,29336343	76,56336		89,95792
79,29683043	76,70451		89,97146
79,41302803	77,56593		89,98876
79,93882828	77,59745		90

80,03770457	77,70401	90
80,19636496	77,76775	90
80,33922404	77,79081	90
80,50530395	78,13851	90
80,68726362	78,34575	90
80,69452642	78,37565	90
80,81982455	78,40127	90
80,97542938	78,43137	90
81,08479542	78,52681	90
81,21323103	78,6339	90
81,45908925	78,92331	90
81,96413134	79,14121	90
82,14045266	79,15352	90
82,31956266	79,18734	90
82,33395098	79,67687	90
82,36789339	79,72861	90
82,41720093	79,87298	90
82,51935647	79,97655	90
83,02554431	80,12467	90
83,17954932	80,19761	90
83,28533642	80,28029	90
83,57453379	80,54409	90
83,59345662	80,76718	90
83,78642535	81,05637	90
83,89626576	81,06921	90
83,98762327	81,16788	90
84,12461506	81,19148	90
84,71459521	81,40126	90
84,77830481	81,41335	90
84,83828129	81,61966	90
84,85878221	81,82658	90
84,96025385	81,86105	90
85,07187952	81,87266	90
85,47825157	82,10925	90
85,77201313	82,12243	90
85,89640747	82,14885	90
85,91764065	82,20631	90
85,97651379	82,46403	90
85,97890714	82,47599	90
86,29028236	82,53001	90
86,46991992	82,5542	90
86,69345111	82,71791	90
86,75061894	82,75915	
86,79958409	82,89277	
87,30890063	83,14801	
87,43854394	83,17743	
87,48780833	83,24983	
87,5383265	83,33287	
87,56201572	83,40825	
87,5692259	83,50321	

87,7769626	84,25248
87,85239779	84,26399
87,89550748	84,30925
87,97480733	84,3759
88,00672173	84,40236
88,02146136	84,46098
88,06220248	84,53205
88,17816759	84,56657
88,31814685	84,6788
88,43430987	84,732
88,53978478	84,83613
88,5536029	84,89807
88,58151011	84,97125
88,73356701	85,16443
89,05181159	85,22507
89,05368472	85,45012
89,09342847	85,46759
89,21171505	85,53447
89,3931811	85,75307
89,61572109	85,8645
89,93945658	85,87013
90	86,07176
90	86,13337
90	86,1742
90	86,33612
90	86,33953
90	86,36816
90	86,46498
	86,58581
	86,64226
	86,70706
	86,74347
	86,78765
	86,79732
	86,80865
	86,85751
	86,91527
	87,01073
	87,01808
	87,19474
	87,26371
	87,26489
	87,27948
	87,34334
	87,53291
	87,61529
	87,82144
	87,8375
	87,85834
	87,86427

87,96396
87,98223
88,00012
88,1836
88,309
88,4025
88,43368
88,47518
88,51782
88,54008
88,56798
88,71899
88,72677
88,73266
88,79859
88,82057
88,82809
88,92241
88,93817
88,98003
89,08628
89,20277
89,20405
89,21622
89,237
89,2993
89,36798
89,56285
89,7262
89,78729
89,8691
89,88838
89,90658
89,9236
90
90
90
90
90
90
90

4h str. + oligomycin (f)

0,060598
0,151727
0,201665
0,343056
0,885022
0,938786
1,014288
1,104306
1,124767
1,141494
1,647428
2,01434
2,177413
2,573874
2,8555
2,904149
3,015462
3,180792
3,433222
4,602971
5,972179
6,490393
6,493603
7,680345
8,377699
8,516619
9,138392
9,507682
9,526611
9,586045
9,792295
10,24984
10,59876
10,89839
12,3916
13,15918
13,31264
14,34206
14,60288
15,12513
16,81269
17,17516
18,32228
19,44889
19,86462
20,23786
20,40794

21,03489
21,36689
21,68196
22,14344
24,25728
25,73411
25,89265
26,33537
26,48002
27,19028
28,98418
29,96892
30,18978
30,9577
32,20065
33,13979
35,12023
36,26217
36,31228
36,86996
36,92737
37,98949
38,29034
38,66028
38,88289
39,24506
39,73698
40,23384
40,81053
41,29323
41,99632
42,03374
42,06562
43,19962
43,20173
43,90685
44,43056
44,57629
45,15126
45,32991
45,78014
45,82668
46,92796
47,55659
47,69844
47,80064
47,91266
48,48996
48,75967
48,86916

48,90365
48,91259
49,14415
49,14433
49,15172
49,33841
50,08937
50,93295
51,33798
51,9382
52,01867
52,06584
52,44061
52,44243
52,54815
52,64636
52,71271
52,71602
52,94874
53,02202
53,1179
53,24499
53,60891
53,71438
54,04676
54,60905
54,94179
55,24081
55,43202
56,09458
56,13265
56,18624
56,32851
56,48722
56,66072
56,79865
56,92514
57,14996
57,45878
57,55678
57,91037
57,9959
58,17677
58,23772
58,78617
59,0882
59,15839
59,29202
59,4388
59,59251

59,75432
60,21504
60,2941
60,58671
61,10271
61,41662
61,45441
61,4942
61,50281
61,58662
61,58707
61,62069
61,69584
61,72644
61,7842
61,8807
62,04315
62,11867
62,19413
62,31848
62,44991
62,49786
62,54931
62,68925
62,75612
62,84302
62,98121
62,9978
63,16569
63,19582
63,26025
63,27054
63,40919
63,79076
63,82111
63,88743
63,9084
63,94861
64,02822
64,1558
64,17582
64,19949
64,40384
64,44992
64,57767
64,64341
64,7999
64,80013
64,86879
64,93938

64,96209
64,97997
64,98802
65,00301
65,08612
65,15637
65,16632
65,23777
65,35875
65,3799
65,41445
65,51124
65,6567
65,71875
65,83017
65,87616
66,21164
66,35138
66,39232
66,40599
66,50296
66,50512
66,55604
66,58366
66,62418
66,66658
66,76482
66,84799
66,86282
66,97048
66,98835
67,02191
67,13566
67,14151
67,48081
67,51483
67,53843
67,63533
67,63566
67,7402
67,84206
67,86801
67,8687
67,89213
67,90883
67,94524
67,98199
68,05533
68,113
68,25273

68,27567
68,29441
68,31351
68,39221
68,4451
68,53154
68,76014
68,84437
68,97632
69,02466
69,06468
69,16243
69,22127
69,23686
69,23841
69,24001
69,28192
69,4169
69,46832
69,58384
69,64298
69,74277
69,91003
69,91115
70,00044
70,00878
70,14929
70,18653
70,30653
70,33263
70,48141
70,48427
70,49674
70,57913
70,76494
70,77387
70,81235
70,82283
70,87494
70,9529
71,10244
71,19154
71,29443
71,43013
71,47151
71,54317
71,63616
71,73858
71,76925
71,99097

71,99569
72,03867
72,04004
72,05249
72,1381
72,30701
72,32358
72,37362
72,3826
72,50167
72,52891
72,69694
72,74562
72,76232
72,81281
72,98443
73,00464
73,03093
73,31014
73,38858
73,40068
73,48678
73,53467
73,65336
73,66484
73,68993
73,7425
73,90112
73,92215
74,04651
74,08785
74,11799
74,26838
74,28633
74,30634
74,30997
74,49659
74,59326
74,68763
74,70795
74,7382
74,96844
75,25668
75,30786
75,45352
75,47757
75,50545
75,56316
75,57764
75,61744

75,69856
75,82334
75,89065
75,95159
75,99873
76,01796
76,30556
76,31862
76,32921
76,41828
76,4714
76,47384
76,54795
76,66987
76,69066
76,84832
76,93402
77,12802
77,14539
77,23207
77,31994
77,34186
77,38759
77,45205
77,46239
77,50624
77,51052
77,51371
77,52798
77,63332
77,76387
77,8595
77,87858
77,9509
78,0047
78,16612
78,1682
78,18217
78,18405
78,25812
78,26365
78,26551
78,30154
78,4651
78,4921
78,55537
78,63038
78,72901
78,84744
79,0919

79,11041
79,1391
79,33096
79,72524
79,77424
79,79209
79,83001
80,07368
80,21658
80,25941
80,34229
80,34882
80,37444
80,56217
80,59794
80,6082
80,67815
80,68792
80,79576
80,80859
80,92695
80,97496
80,98735
81,06185
81,16271
81,18927
81,23836
81,3122
81,49544
81,5463
81,64103
81,64671
81,72769
81,78294
81,89193
82,15872
82,28138
82,29686
82,30544
82,33958
82,48499
82,70233
82,78399
82,8242
82,86947
82,94881
83,0544
83,0821
83,121
83,14823

83,16359
83,25487
83,38321
83,44331
83,52161
83,53393
83,57154
83,62229
83,66877
83,71315
83,74652
83,7838
84,04308
84,05737
84,09785
84,1546
84,19503
84,26427
84,28866
84,29268
84,32357
84,34671
84,38613
84,43013
84,44648
84,46506
84,5975
84,61724
84,68845
84,72003
84,74876
84,78448
84,78584
84,78688
84,80929
84,88606
84,99933
85,02522
85,04931
85,15653
85,25663
85,27531
85,30573
85,41102
85,49794
85,55257
85,60604
85,61704
85,61991
85,65045

85,80608
85,86657
85,89765
85,90485
85,91296
85,93805
86,05674
86,12643
86,21752
86,22036
86,24165
86,26238
86,26371
86,29634
86,29832
86,32522
86,39156
86,47663
86,49931
86,55871
86,62114
86,64915
86,70808
86,72775
86,73301
86,76911
86,78157
86,87595
86,88274
86,92756
86,96559
87,07099
87,12013
87,18689
87,26577
87,40438
87,43504
87,50408
87,61297
87,6275
87,65944
87,71554
87,73472
87,74795
87,79123
87,81796
87,82512
87,87667
87,88706
87,91327

87,91419
87,9546
88,05276
88,11809
88,2283
88,23108
88,35421
88,35976
88,37179
88,38076
88,40653
88,42344
88,46294
88,48425
88,51199
88,60408
88,62365
88,65324
88,65711
88,6655
88,6826
88,70391
88,71774
88,89334
88,9077
89,00307
89,00394
89,01434
89,02427
89,02664
89,07773
89,12138
89,24237
89,24986
89,27112
89,27472
89,36995
89,37327
89,37547
89,37754
89,44343
89,45037
89,45274
89,54094
89,63403
89,71409
89,78057
89,78142
89,82803
89,86407

89,87114
89,89507
89,92268
89,98971
90
90
90
90
90
90
90
90
90
90
90
90
90
90
